# N‐Insertion of Diazonium Salts Into Ketone Derivatives

**DOI:** 10.1002/anie.202505341

**Published:** 2025-05-06

**Authors:** Mohammed Anif Pasha, Jiwon Jang, Youngjin Bae, Seunghoon Shin

**Affiliations:** ^1^ Department of Chemistry and Research Institute for Convergence of Basic Science Hanyang University Seoul 04763 South Korea; ^2^ Syngene International Ltd. Biocon Park SEZ Bommasandra Industrial Area – Phase IV, Jigani Link Road Bangalore Karnataka 560 099 India

**Keywords:** Azepinones, Diazonium salts, Isoquinolinone, Ring expansion, Skeletal editing

## Abstract

We report a new oxidative nitrogen insertion into cyclic ketones using readily available diazonium salts. In the case of indanones, the reaction is promoted by auto‐catalytically generated Brønsted acid and proceeds via sequential α‐diazenylation and ring expansion through an N‐iminoaziridinium intermediate. For less α‐acidic ketones, a complementary strategy employing silyl enol ethers was developed: catalyzed by HNTf_2_, efficient nitrogen insertion occurred into silyl enol ethers derived from a broad range of four‐ to seven‐membered cyclic ketones. The resulting N‐aminoamides exhibit broad synthetic utility in various downstream transformations. Notably, a ring expansion, followed by N─N bond cleavage offers a powerful tool for skeletal editing, converting indanones into isoquinolinones, as demonstrated by the scaffold modification of donepezil.

## Graphical abstract

Diazonium salts insert into ketones and their silyl enol ethers through a sequence of α‐azenylation and ring expansion via an N‐iminoaziridinium species. Coupled with an efficient N‐N bond cleavage, this protocol offers a mild alternative to Beckmann rearrangements and a powerful tool for skeletal editing of ketones.

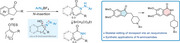

Arene diazonium salts,^[^ ^1^, ^2^ ^]^ readily prepared from anilines, are well‐established carbon electrophiles, functioning as aryl radical precursors (Scheme 1a, left).^[^ ^3^, ^4^, ^5^, ^6^, ^7^ ^]^ Although significant advances have been made in their activation using transition metal,^[^ ^8^, ^9^, ^10^, ^11^ ^]^ photoredox,^[^ ^12^, ^13^ ^]^ and electrochemical methods,^[^ ^14^ ^]^ their use as nitrogen sources with dinitrogen retention remains relatively underexplored. Reported examples include nucleophilic attack by carbon nucleophiles, such as arenes^[^ ^15^ ^]^ and C–H acids,^[^ ^16^, ^17^, ^18^, ^19^ ^]^ as well as interception of radical intermediates to form diazo adducts (Scheme 1a, right).^[^ ^20^, ^21^, ^22^ ^]^

**Scheme 1:**
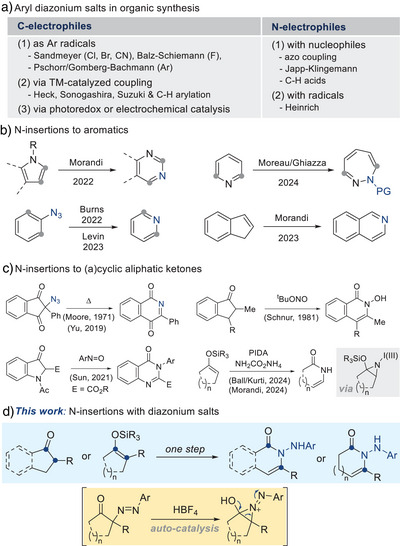
Backgrounds of N‐insertion of diazonium salts into ketones.

Skeletal editing, an emerging strategy for medicinal chemistry, enables precise modification of molecular cores, streamlining structure–activity relationship studies.^[^ ^23^, ^24^, ^25^, ^26^ ^]^ Within this context, oxidative nitrogen insertion into (hetero)aromatic compounds has attracted considerable interest (Scheme 1b).^[^ ^27^, ^28^, ^29^, ^30^, ^31^, ^32^, ^33^, ^34^ ^]^ Of particular interest are nitrogen insertions into aliphatic scaffolds derived from common feedstocks such as ketones. Classical transformations such as Beckmann, Schmidt, and related rearrangements exemplify this approach, converting ketones into amides.^[^ ^35^, ^36^, ^37^, ^38^ ^]^ Ring expansions of α‐azido^[^ ^39^, ^40^ ^]^ α‐nitroso,^[^ ^41^ ^]^ and α‐hydroxyamino^[^ ^42^ ^]^ ketones have also been shown to yield amides (Scheme 1c). Recently, Ball/Kürti and Morandi independently reported electrophilic ammonia equivalents for related transformations, proceeding through a common aziridinium intermediate bearing I(III) moiety as a leaving group.^[^ ^43^, ^44^ ^]^

Building on these advances, we report our discovery that diazonium salts induce one‐pot N‐insertion into ketones and their silyl enol ethers. This transformation, utilizing readily available diazonium salts, efficiently effects ring expansions of a broad range of ketones to afford the corresponding N‐aminoamides.

We began by investigating the reaction of 2‐methyl‐1‐indanone **1a** with 4‐fluorophenyl diazonium salt **2a** (Table 1). Heating **1a** and **2a** in acetonitrile at 50 °C for 8 h yielded the N‐insertion product **3aa** in 90% yield (Method 1), confirmed by X‐ray crystallography.^[^ ^45^ ^]^ The reaction required heating, as no conversion occurred at room temperature (entry 2). Intriguingly, the reaction exhibited a distinct induction period of ∼4 h, when a trace amount of **I‐aa** (<5%) started to appear and then a rapid conversion to **3aa** occurred, suggesting autocatalysis (Figure 1a). Inspection of a mixture of **2a** in CH_3_CN heated at 50 °C revealed a small amount of 4‐F‐C_6_H_4_NHAc, presumably formed from the S_N_Ar reaction of **2a** with acetonitrile and extraneous water,^[^ ^46^ ^]^ generating HBF_4_. Once generated, HBF_4_ may catalyze further α‐diazenylation to **I‐aa**, generating more HBF_4_ in an autocatalytic manner. Supporting this, adding 10 mol% of HBF_4_ eliminated the induction period (Figure 1b) and facilitated the formation of **3aa** at room temperature (entry 3). The reaction was completely inhibited by 2,6‐lutidine (entry 4) or 4 Å molecular sieves (entry 5). Coordinating solvents hindered the conversion (entry 6), and chlorinated solvents slowed the reaction, likely due to the limited solubility of **2a** (entry 7). Using one equivalent of **2a** required a longer reaction time and resulted in lower yield (entry 8). The minimal accumulation of **I‐aa** (Figure 1) during the reaction suggests that slow acid‐catalyzed α‐diazenylation to **I‐aa**, followed by rapid ring expansion to **3aa**.

Simple treatment of 1‐indanones with arene diazonium salts in the absence of additives or catalysts emerged as an appealing approach, prompting us to explore its generality (Method 1, Table 2). Our initial focus was on the influence of the electronic properties of the arene diazonium salts on the reaction outcome. Diazonium salts bearing electron‐withdrawing substituents (**2b–e** and **2g–2k**) facilitated efficient ring expansion. Conversely, the presence of electron‐donating groups at the para‐position (**2f**) led to significantly diminished conversion, presumably due to a sluggish autocatalytic generation of the catalytically active HBF_4_ and due also to a sluggish ring expansion (vide infra in Scheme 4). In this case, the addition of Brønsted acid catalyst failed to improve the yield further, because of a competing Baeyer–Villiger type oxidation of ketones (Table S2).^[^ ^47^ ^]^

**Table 1:** Examination of Conditions for N‐Insertion to **1a**.^a)^

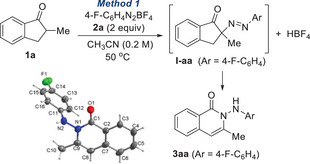				
Entry	Deviation from above	Time (h)	Conv^b)^ (%)	3aa^c)^ (%)
1	None (***Method 1***)	8	>99	90
2	Rt	24	0	0
3	HBF_4_ (10 mol%), rt	8	>99	85
4	2,6‐lutidine (10 mol%)	24	0	0
5	MS 4 Å (40 mg)	24	0	0
6	in 1,4‐dioxane, DMSO or DMF	24	0	0
7	In CH_2_Cl_2_	24	>99	80
8	**2a** (1.0 equiv)	18	>99	86

^a)^ Conditions: **1a** (0.1 mmol), **2a** (0.2 mmol) in CH_3_CN (0.5 mL).

^b)^ Determined from the crude ^1^H NMR spectra with CH_2_Br_2_ as an internal standard.

^c)^ Isolated yield after flash chromatography.

**Figure 1:**
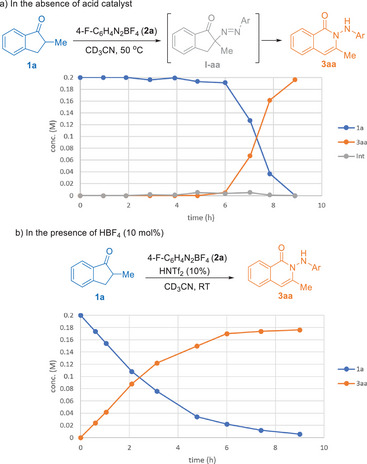
Reaction progress in the presence and absence of Brønsted acid catalyst.

**Table 2:** Reactions of Indanones with Arene Diazonium Salts.^a)^

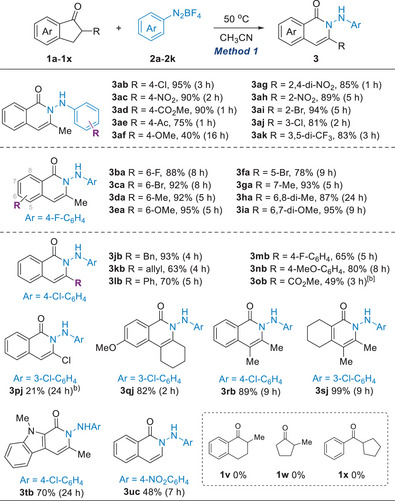

^a)^ Conditions: **1a** (0.2 mmol) and **2** (0.4 mmol) in CH_3_CN (1 mL) at 50 °C; isolated yield after flash chromatography.

^b)^ In the presence of HBF_4_ (1 equiv.).

We then explored the scope of substituted indanones. Isoquinolinones (**3ba**–**3ia**) with C5–C8 substituents were obtained efficiently. C3‐Substituted products with benzyl (**3jb**), allyl (**3** **kb**), and aryl groups (**3lb**‐**3 nb**) were also readily prepared. However, C3 electron‐withdrawing groups, such as esters (**3ob**) and halogens (**3pj**), required the addition of 1 equiv. of HBF_4_ for conversion, albeit in modest yields. The reaction was further extended to synthesize 3,4‐dialkyl isoquinolinones (**3qj–3rb**). Notably, non‐benzenoid pyridin‐2‐one **3sj** and indole‐fused **3tb** were obtained in good to excellent yields. Indan‐1‐ones lacking α‐substituents were poor substrates with **2a** or **2b**; In this case, a strongly electrophilic *p‐*NO_2_C_6_H_4_N_2_BF_4_ **2c** provided **3uc** in 48% yield. Unfortunately, the one‐step protocol was ineffective with six‐membered ketone **1v**, non‐benzenoid ketone **1w**, and acyclic ketone **1x**, presumably due to the lower acidity of α‐hydrogen.

To address the limitations in substrate scope, we developed a complementary protocol employing silyl enol ethers, which are conveniently accessible from the corresponding ketones (Table 3).^[^ ^44^ ^]^ Treatment of silyl enol ether **4e** (2 equiv) with **2c** (1 equiv) in the presence of HNTf_2_ (10 mol%) at room temperature produced the ring‐expanded product **5ec** in 75% yield (entry 1: Method 2). Without HNTf_2_ catalyst, the reaction stalled at the α‐diazenylated **I‐ec** (entry 2), indicating that the ring expansion is not catalyzed by silylium salt Et_3_SiBF_4_ generated during the reaction.^[^ ^48^ ^]^ Use of HBF_4_ or in situ HBF_4_ generation via water addition was less effective (entries 3 and 4). By using an excess of diazonium salts, an inferior yield was obtained (entry 5, Table S3). Coordinating solvents (THF, DMF, DMSO, and acetone) halted the reaction at **I‐ec** (entry 6; Table S4), and higher concentrations led to decomposition and lower yield (entry 7; Table S5). Less reactive arene diazonium salts (**2a–2b** and **2l**) yielded mostly the respective α‐diazenylated intermediates **I‐ea**, **I‐eb**, and **I‐el** (entries 8–10). Change to TBDMS ether had negligible effects (entry 11).

**Table 3:** Conditions for N‐Insertion to Silyl Enol Ether **4e**.^a)^

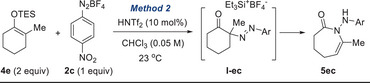
Entry	Deviation from above	Time (h)	5ec (%)^b)^
1	None (***Method 2***)	1	75
2	no HNTf_2_	24	0 (**I‐ec**, 44)
3	HBF_4_ instead of HNTf_2_	6	41
4	H_2_O (1 equiv)	1	23
5	**4e** (1 equiv), **2c** (2 equiv)	1	69
6	THF as solvent	1	0 (**I‐ec**, 64)
7	CHCl_3_ (0.1 M)	0.25	52
8	**2a** (4‐F‐C_6_H_6_N_2_BF_4_)	7	0 (**I‐ea**, 72)
9	**2b** (4‐Cl‐C_6_H_6_N_2_BF_4_)	7	18 (**I‐eb**, 45)
10	**2l** (C_6_H_5_N_2_BF_4_)	7	0 (**I‐el**, 34)
11	TBDMS instead of TES	1	75

^a)^ Conditions: **4e** (0.2 mmol), **2c** (0.1 mmol) in CHCl_3_ (2 mL);

^b)^ Determined from the crude ^1^H NMR spectra with CH_2_Br_2_ as an internal standard.

With optimized conditions in hand (Method 2), we investigated the reaction of silyl enol ethers derived from various ketones (Table 4). Cyclobutanone‐derived silyl enol ether **4a** underwent ring expansion to give **5ac** with an exocyclic double bond in 78% yield. A series of silyl enol ethers derived from cyclopentanones (**4b–4d**) were found to be suitable substrates, affording the corresponding products **5bc–5dc** in reasonable to good yields. Furthermore, the reaction proved applicable to cyclohexanone‐derived silyl enol ethers (**4e–4j**), providing access to the corresponding azepin‐2‐ones **5ec–5jc**. Other electron‐deficient arene diazonium salts (**2e** and **2f**) were able to promote the ring expansion to afford **5ee** and **5dg**, respectively. Encouragingly, we were also able to synthesize an eight‐membered **5kc** using this protocol.

**Table 4:** Scope of Silyl Enol Ethers.^a)^

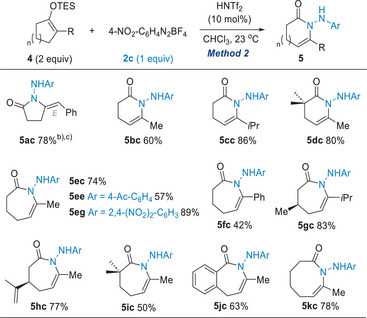

^a)^ Conditions: **4a** (0.4 mmol), **2c** (0.2 mmol) in CHCl_3_ (4 mL); isolated yield after flash chromatography.

^b)^ Silyl enol ether **4a** was used as a mixture of regioisomers (6.7:1).

^c)^ NOE experiments confirmed the *E*‐geometry of the double bond.



(1)

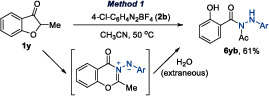



(2)

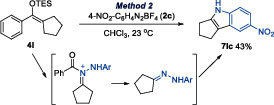




Notably, some ketones or silyl enol ethers yielded unexpected products, though their formation can be rationalized within the proposed mechanism (Equations 1 and 2). For example, benzofuran‐3‐one derivative **1y** afforded **6yb**, likely via hydrolysis of the intermediate (Equation 1). Similarly, **4l** from an acyclic ketone reacted to give indole **7lc**, which likely arises from hydrolysis of the N‐acyl hydrazone intermediate, followed by a subsequent Fischer indolization (Equation 2).

We then explored functional group interconversion of the products. The N─N bond in N‐aminoamide (**3ab**) was efficiently cleaved into an isoquinolinone **8ab** in 93% yield, through N_β_‐alkylation and subsequent elimination. Treating **3ab** with NBS under Lewis base catalysis furnished brominated **9ab**. Chemoselective reduction of the olefin in **3ab** in the presence of the N─N bond gave **10ab**. Additionally, Pd‐catalyzed oxidative cyclization of the Ph‐substituted derivative **3lb** produced the fused cycle **11lb**. The N‐aminoamide products exhibited Ritter‐type reactivity in acetonitrile (Scheme 2b), involving trapping of the N‐aminoiminium intermediate by acetonitrile and intramolecular ring closure. For example, treating five to seven‐membered lactam derivatives **5ac**, **5bc,** and **5ec** with HNTf_2_ in acetonitrile at room temperature afforded 1,2,4‐triazol‐fused cycles **12ac**, **12bc**, and **12ec**, respectively.

**Scheme 2:**
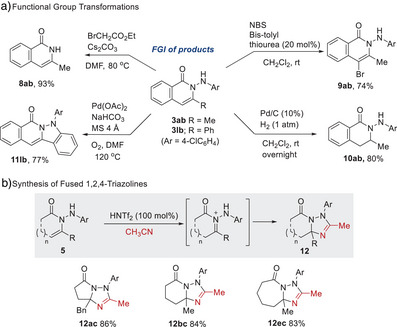
Synthetic applications.

A sequence of ring expansion and N─N bond cleavage provides an efficient tool for skeletal editing, transforming indanones into Isoquinolinones, which are privileged structural motifs in many pharmacological agents.^[^ ^49^ ^]^ To demonstrate this, we investigated skeletal editing of donepezil, a drug used to treat Alzheimer's disease by increasing acetylcholine levels (Scheme 3). The indanone core of donepezil was efficiently transformed into an isoquinolinone **13** in 78% yield over two steps.

**Scheme 3:**
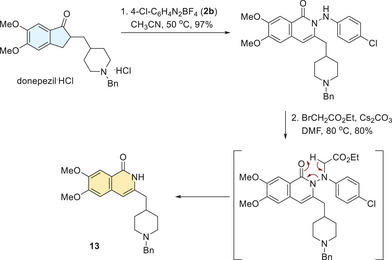
Skeletal Editing of Donepezil into an Isoquinolin‐2‐one.

To interrogate the mechanism of ring expansion, we conducted a Hammett plot study (Scheme 4). We prepared several derivatives of α‐diazenyl ketone intermediates having varying substituents (X) at the C5‐position of indanone. In this case, electron‐releasing groups that can stabilize the benzylic cation (i.e., C1) by resonance accelerated the ring expansion, with a linear free energy relation with σ^+^ (ρ = −1.3). In addition, the *p‐*position of the aryl groups (Y) on the diazonium salts was varied. In the latter case, the observed rate had a linear correlation with σ_P_, suggesting electron‐withdrawing substituents stabilize the developing negative charge at the N2 atom in the transition state. Together, these experiments are consistent with a transition state having a partial positive charge developing at C1 and a negative charge at N2.

**Scheme 4:**
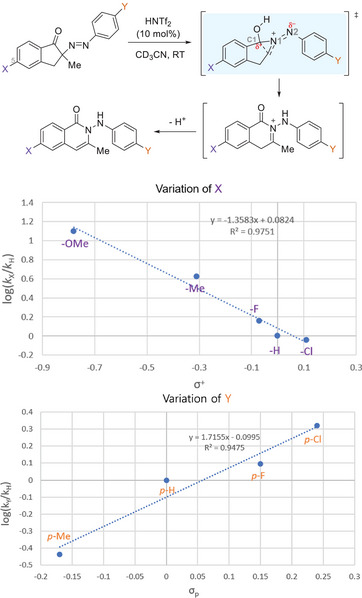
Hammett plots of the ring expansion step and the transition state charge distribution.

In summary, we developed a mild, one‐step N‐insertion into ketones and silyl enol ethers using readily accessible diazonium salts as nitrogen sources. This method is effective for a wide range of four‐ to seven‐membered cyclic ketones, encompassing both aromatic and aliphatic systems, and provides a valuable alternative to traditional approaches such as Beckmann rearrangement. Furthermore, we have showcased the versatility of the resulting products through various transformations, including the modification of the drug donepezil.

## Conflict of Interests

The authors declare no conflict of interest.

## Supporting information



Supporting Information

Supporting Information

## Data Availability

The data that support the findings of this study are available in the supplementary material of this article.
