# Supplementary material for: N‐Insertion of Diazonium Salts Into Ketone Derivatives
Source: Angew Chem Int Ed Engl. 2025 May 6;64(29):e202505341. doi: 10.1002/anie.202505341 (PMC12258690; doi:10.1002/anie.202505341)
Supplement: Supplementary file 1 — Supporting Information [file ANIE-64-e202505341-s001.pdf]

Supporting Information

N-Insertion of Diazonium Salts into Ketone Derivatives

Mohammed Anif Pasha, Jiwon Jang, Youngjin Bae and Seunghoon Shin*

Department of Chemistry, Research Institute for Convergence of Basic Science, Hanyang University, Seoul 04763, Korea.

sshin@hanyang.ac.kr

Table of Contents

1. General Information--S2

2. Further Optimization Data---S3

3. Synthesis of Substrates--S9

4. Representative Procedures--S13

5. Characterization of New Compounds--S14

6. Synthetic Applications---S34

7. Mechanistic Experiments--S41

8. X-ray Crystallographic Data of **3aa**--S43

9. References---S51

Appendix: Copy of ^{1}H and ^{13}C spectra

1. General Information

All materials (including substrates **1a**, **1b**, **1c**, **1f**, **1g**, **1i**, **1u**, **1v**, and **1w**) were purchased from TCI, Aldrich, or Alfa Aesar and were used as received. Dry THF, CH_3CN, toluene, CH_2Cl_2 and ether were obtained after passing the HPLC grade solvents through activated aluminum column. All other solvents were dried and distilled according to standard methods before use. TLC (thin-layer chromatography) analyses were carried out on Merck silica gel 60 F254 TLC plates and was visualized with UV lamp and $KMnO_4$ solution. Flash chromatography was performed on Kieselgel 60 (230-400 mesh). ^{1}H and ^{13}C NMR spectra were recorded on a Bruker (400 MHz) spectrometer with TMS as an internal standard. High resolution mass spectra (HRMS) were obtained from Organic Chemistry Research Center in Sogang University.

[Safety hazards associated with diazonium salts]

Many diazonium salts are thermally unstable and sensitive to friction and shock.[9] Most diazonium salts are known for their violent decomposition hazard in the solid state.[9b] For the experiments described herein, it is recommended that no more than 0.75 mmol of diazonium salts are prepared at one time.[9a]

2. Further Optimization Data

Table S1. Screening of solvent (Method 1)[a]

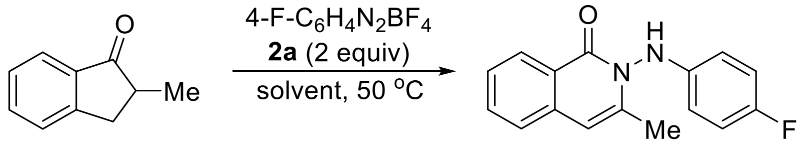

Entry	Solvent	Time (h)	Conv. (%)	Yield (%)
1	CH_3CN	8	>99	90
2	1,2-DCE	24	>99	90
3	Acetone	24	>99	86
4	CH_2Cl_2	24	>99	80
5	$CHCl_3$	24	48	22
6	1,4-dioxane	24	0	0
7	DMSO	24	0	0
8	DMF	24	0	0

[a]**1a** (0.1 mmol), **2a** (0.2 mmol) in CH_3CN (0.5 mL); isolated yield after flash chromatography.

[Monitoring Reaction Progress: Figure 1]

The following procedure was followed for the reaction monitoring in Figure 1.

Figure 1(a): in the absence of HBF_4 catalyst.

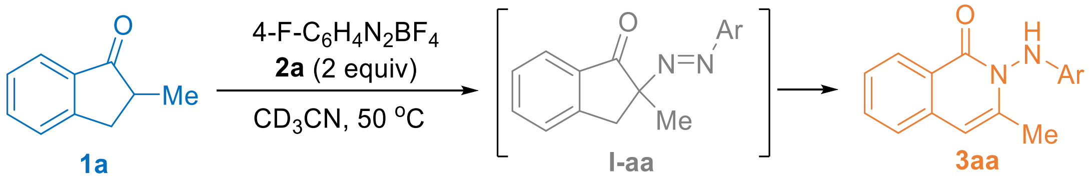

To a suspension of diazonium salt **2a** (0.2 mmol) in CD_3CN (0.5 mL) was added indanone derivative **1a** (0.1 mmol) and CH_2Br_2 (0.1 mmol) as an internal standard. The mixture was heated to 50 °C and the reaction progress was monitored by ^1H NMR spectrometry.

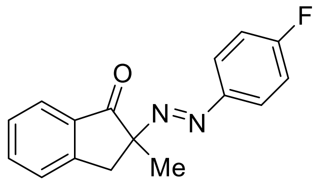

I-aa (EtOAc/nHex = 1:3, R_f = 0.6) as a yellow oil; ^{1}H NMR (400 MHz, $CDCl_3$): δ 7.81 (d, *J* = 7.7 Hz, 2H), 7.70 (dd, *J* = 9.0, 5.2 Hz, 2H), 7.66 (t, *J* = 7.3 Hz, 1H), 7.53 (d, *J* = 7.7 Hz, 1H), 7.42 (t, *J* = 7.6 Hz, 1H), 7.09 (t, *J* = 8.8 Hz, 2H), 3.91 (d of ABq, *J* = 17.3 Hz, 1H), 3.24 (d of ABq, *J* = 17.4 Hz, 1H), 1.67 (s, 3H); ^{13}C NMR (100 MHz, $CDCl_3$): δ 202.8, 164.2 (d, *J* = 250.1 Hz), 152.4, 148.2 (d, *J* = 2.8 Hz), 135.6, 135.3, 127.9, 126.7, 124.9, 124.6 (d, *J* = 9.1 Hz), 115.8 (d, *J* = 22.7 Hz), 81.4, 39.6, 20.9; IR (ATR): ṽ 3069, 2975, 2923, 1717, 1596, 1513, 1492, 1468, 1283, 1222, 1137, 1091, 960, 840, 797, 734 cm^{-1}; HRMS (EI) Calcd for $C_{16}H_{14}FN_2O^+$ $[M+H]^+$ 269.1085; found 269.1085.

Figure 1(b): in the presence of HBF_4 (10 mol%)

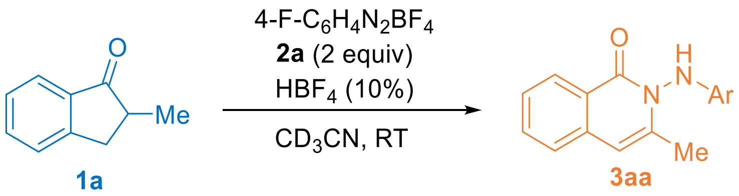

To a suspension of diazonium salt **2a** (0.2 mmol) in CD_3CN (0.5 mL) was added indanone derivative **1a** (0.1 mmol), CH_2Br_2 (0.1 mmol), and $HNTf_2$ (0.01 mmol). The mixture was heated to 50 °C and the reaction progress was monitored by ^{1}H NMR spectrometry.

Figure S1. Reaction progress using **1a** and **2c**

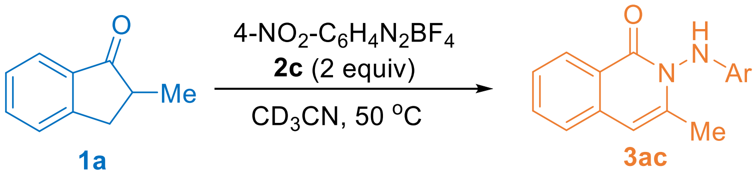

Following identical method to above, the reaction of **1a** with **2c** (4-NO_2-$C_6H_4N_2BF_4$) was compared with the reaction with **2a** (4-F-$C_6H_4N_2BF_4$).

Employing a more reactive **2c** (4-NO_2-$C_6H_4N_2BF_4$), the induction period was still observed,

but was shortened to ca. 1 h.

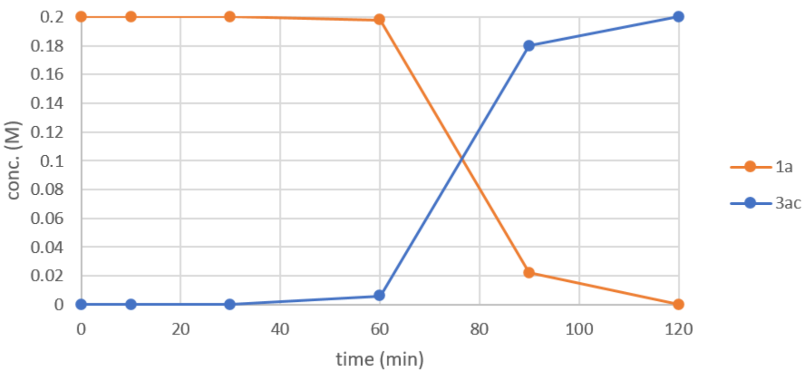

Figure S1. Reaction progress using **1a** and **2c**

Table S2. Reactions with 4-MeO-$C_6H_4N_2BF_4$ in the presence of acid catalyst (Method 1)[a]

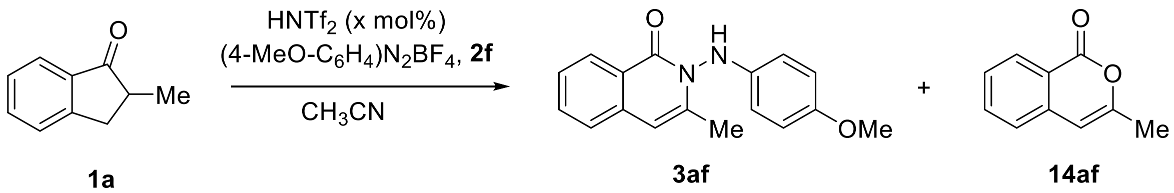

Entry	$HNTf_2$	Temp	Time	Yield (%)
1	none	50 °C	16 h	**3af** (40)
2	10 mol%	RT	24 h	**3af** (12)+**14af** (22%)
3	50 mol%	RT	24 h	**3af** (13)+**14af** (42%)
4	10 mol%	50 °C	24 h	**3af** (23)+**14af** (44%)

[a]A mixture of **1a** (0.1 mmol) and **2f** (0.2 mmol) in CH_3CN (0.5 mL) was placed in a vial and was capped under air; yields were determined from crude ^1H NMR spectra with CH_2Br_2 as an internal standard.

In the case of electron-rich arene diazonium salts (**2f**), we obtained only low yield of desired product **3af** (40%, entry 1, Table S2). To improve the yield of **3af**, addition of acid catalyst was tested (entry 2). In the presence of $HNTf_2$ (10 mol%) the reaction proceeded at RT, but a lower yield of **3af** (12%) and unexpected product **14af** (22%) was obtained, with the rest of the mass balance was unreacted starting material. Neither higher loading of acid catalyst (entry 3) nor higher temperature (entry 4) improved the yield of **3af**. The structure of **14af** was confirmed by comparison with literature.[10] It formed most likely from the reaction of its enol form with

air (O_2). The competitive formation of **14af** is due to the comparitively slower reaction with electron-rich arene diazonium salts with 4-MeO-C_6H_4 group.

Table S3. Screening of equivalent (Method 2)[a]

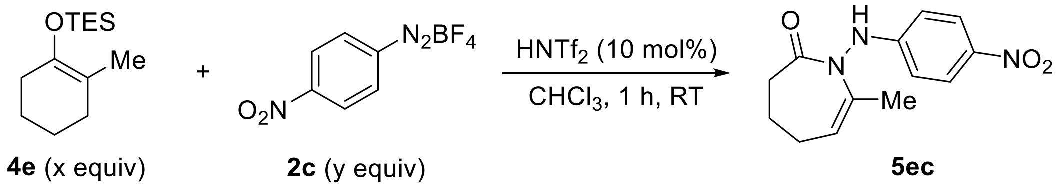

Entry	x	y	Yield (%)
1	1.0	2.0	69
2	1.0	1.0	55
3	1.5	1.0	70
4	2.0	1.0	75
5	3.0	1.0	50

[a]**2c**, **4e** (0.1 mmol was used as limiting reagent) in $CHCl_3$ (2 mL); yields were determined from crude ^1H NMR spectra; a vinyl peak of **5ec** (triplet at 5.61 ppm) was integrated relative to an internal standard, CH_2Br_2 (singlet at 4.92 ppm).

Table S4. Screening of solvent (Method 2)[a]

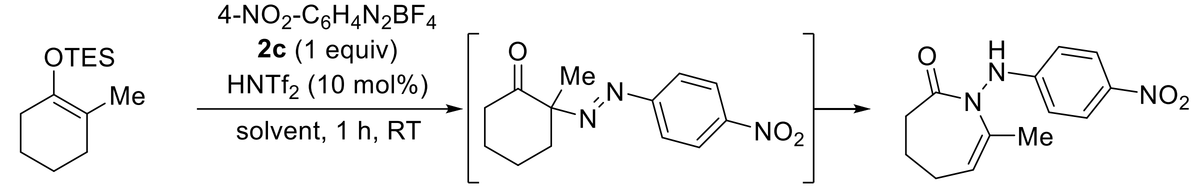

4e (2 equiv) **I-ec** **5ec**

Entry	solvent	Yield (%) (**I-ec**/**5ec**)
1	MeCN	0 / 53
2	$MeNO_2$	0 / 31
3	CH_2Cl_2	0 / 63
4	$CHCl_3$	0 / 75
5	DCE	0 / 62
6	Ph-Cl	0 / 72
7	THF	64 / 0
8	DMF	62 / 0
9	DMSO	64 / 0
10	acetone	58 / 0
11	HFIP	0 / 0

[a]**2c** (0.1 mmol), **4e** (0.2 mmol) in solvent (2 mL); yields were determined from crude ^{1}H NMR spectra; a vinyl peak of **5ec** (triplet at 5.61 ppm) was integrated relative to an internal standard, CH_2Br_2 (singlet at 4.92 ppm).

Table S5. Screening of concentration (Method 2)[a]

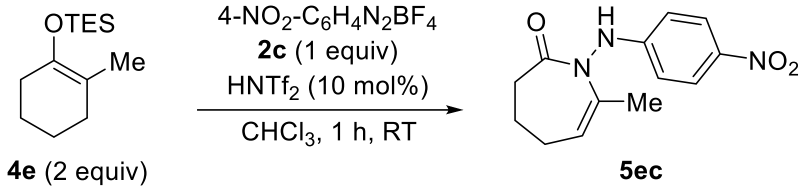

Entry	Concentration	Time	Yield (%)
1	0.5 M	15 min	43
2	0.1 M	15 min	52
3	0.05 M	1 h	75
4	0.01 M	3 h	0

[a]**2c** (0.1 mmol), **4e** (0.2 mmol) in $CHCl_3$; yields were determined from crude ^1H NMR spectra; a vinyl peak of **5ec** (triplet at 5.61 ppm) was integrated relative to an internal standard, CH_2Br_2 (singlet at 4.92 ppm).

Table S6. Screening of catalyst loading (Method 2)[a]

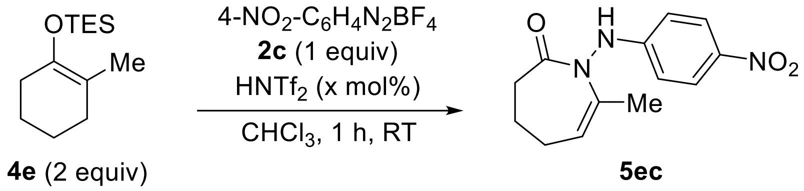

Entry	$HNTf_2$	Time	Yield (%)
1	none	24 h	0
2	10 mol%	1 h	75
3	50 mol%	15 min	74

[a]**2c** (0.1 mmol), **4e** (0.2 mmol) in $CHCl_3$ (2 mL); yields were determined from crude ^1H NMR spectra; a vinyl peak of **5ec** (triplet at 5.61 ppm) was integrated relative to an internal standard, CH_2Br_2 (singlet at 4.92 ppm).

3. Synthesis of Substrates

General Procedure A[1]

(Synthesis of **1d**,[2a] **1e**,[1b] **1g**,[2b] **1h**,[2c] **1j**[1a], **1k**[1b], **1o**[1a], **1r**[1a]; ketone precursors of **4b**[1c] and **4c**[1c])

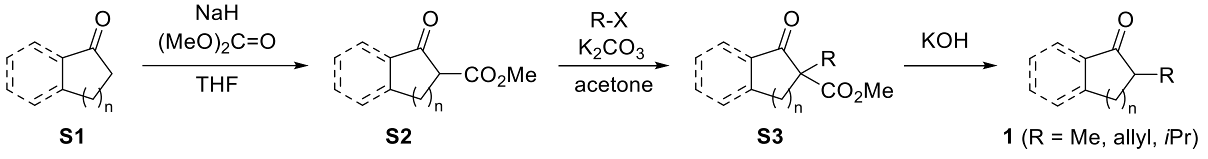

(**S2**) To a suspension of NaH (21 mmol) in THF (90 mL) was added dimethyl carbonate (50 mmol) at room temperature. The mixture was stirred for 5 min and then ketone **S1** (10 mmol) in THF (30 mL) was added dropwise. The mixture was stirred 4 h at room temperature, until the completion of the reaction by TLC. The mixture was then cooled to 0 °C and was quenched with 1 N aq. HCl until neutral pH. Water (100 mL) was added and the product was extracted with ether (80 mL x 3). The combined organic layers were washed with brine (50 mL), dried (Na_2CO_3), and concentrated. The residue was purified by flash chromatography (EtOAc/Hex) to afford 80-95% of **S2**.

(**S3**) To a mixture of K_2CO_3 (2.4 equiv) in acetone (0.2 M) was added β-keto ester **S2** cooled at 0 °C was added dropwise a solution of alkyl halide (1.2 equiv; MeI, BnBr, or allyl chloride) in acetone. The mixture was heated to reflux. When the TLC indicated complete reaction, the mixture was cooled to RT and was filtered through a short pad of celite, and was washed with copious amount of acetone. The filtrate was concentrated.

The crude **S3** was dissolved in ethanol-water (5:1, 30 mL) and was treated with KOH (2.5 equiv), and the reaction mixture was heated to reflux for 2 h. The residue was neutralized with saturated aq. NH_4Cl (20 mL) and the aqueous layer was extracted with EtOAc (20 mL x 3). The combined organic layers were washed with brine (20 mL), dried (Na_2SO_4), and concentrated in vacuo. The residue was purified by flash chromatography (EtOAc/Hex) to afford 50-80% of **1**.

General Procedure B[3a]

(Synthesis of **1l**,[3b] **1m**,[3b] **1n**[3b])

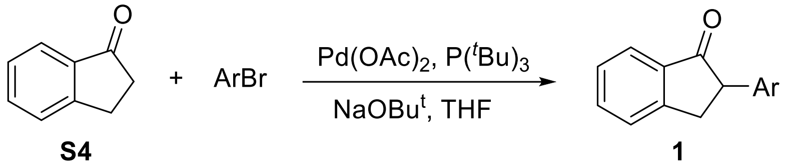

$Pd(OAc)_2$ (2 mol%), tri(tert-butyl)phosphine (2 mol%), and NaO^tBu (1.5 equiv.) were suspended in THF (15 mL) in a screw capped vial. The vial was sealed with a cap containing a PTFE septum and removed from the glove box. Bromobenzene (1.0 mmol) and 1-indanone (1.1 mmol) were added to the reaction mixture by syringe. The reaction mixture was stirred at 25 °C and was monitored by TLC. After completion of reaction, the crude reaction was diluted with ether (30 mL) and washed with 1 N HCl, water, and brine. The organic layer was dried over Na_2SO_4, filtered, and concentrated in vacuo. The residue was chromatographed on silica gel (EtOAc/hexane = 1:20) to give 85-92% of 2-aryl-1-indanones.

General Procedure C[4]

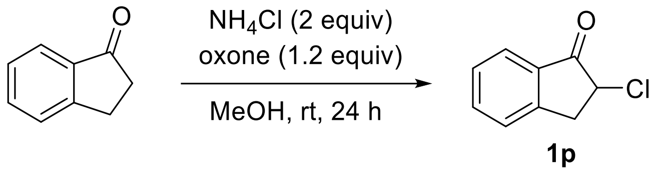

(**1p**)[4] Identical procedure to the literature was followed.

General Procedure D[5a]

(Synthesis of **1q**[5a], **1r**[5b], **1s**[5c], **1t**[5d])

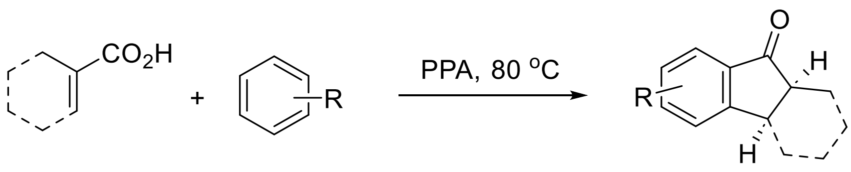

1-Cyclohexene carboxylic acid or corresponding acrylic acid (2.0 equiv.) was added to a stirred neat mixture of arene (1.0 equiv.) and polyphosphoric acid (PPA) (2.5 equiv.) maintained at 80 °C. After 2.5 h, dark-red reaction mixture was poured into ice, stirred until the resulting sticky mass had completely dissolved (*ca.* 1 h) and then extracted with ether. The combined organic phases were then dried ($MgSO_4$), filtered and concentrated under reduced pressure. The resulting dark red oil was subjected to flash chromatography on silica (EtOAc/hexane = 1:5). Concentration of the appropriate fractions and flash chromatography afforded title ketones (92-98%).

General Procedure E1[6]

(Synthesis of **4a**, **4b**[7a], **4c**, **4d**, **4g**, **4j**[7e], **4k**, **4l**[6])

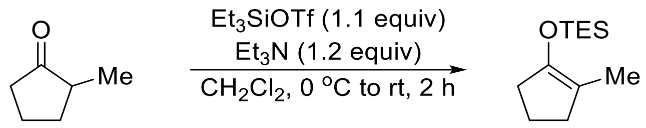

To a flame-dried flask was added ketone (5 mmol) and Et_3N (20 mmol) in CH_2Cl_2 (10 mL). The mixture was cooled to 0 °C and Et_3SiOTf (7.5 mmol) was added dropwise while stirring. The mixture was allowed to warm to RT over 2 h. Upon completion of the reaction (TLC), the mixture was diluted with hexane (50 mL) and was washed with sat. aq. $NaHCO_3$ (50 mL) and brine (30 mL). The organic layer was dried (Na_2SO_4), filtered, and was concentrated in vacuo. The residue was purified by flash chromatography with silica (SiO_2) that was neutralized by treatment with 2% Et_3N in acetone.

Synthesized **4b**[7a] and **4j**[7e] had matching spectral data with their respective literature data.

General Procedure E2[6]

(Synthesis of **4e**[7b], **4f**[7c], **4h**[7d], **4i**)

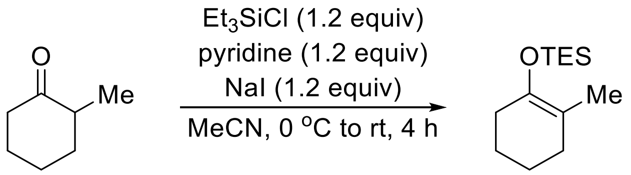

A dried round bottom flask containing a stir bar was charged with ketone (5.0 mmol). The round bottom flask was then evacuated/backfilled with argon three times. The round bottom flask was then cooled to 0 ºC in an ice bath and stirred while pyridine (6.0 mmol) and Et_3SiCl (6.0 mmol) were added. Separately, NaI (6.0 mmol) was dissolved in MeCN (10 mL) under argon. The NaI solution was then added dropwise at 0 ºC via syringe, and the reaction was allowed to gradually warm to room temperature. Upon completion of the reaction, the solution was diluted with hexane (50 mL) and washed sequentially with cold sat. aq. $NaHCO_3$, then brine. The combined organic layers were then dried (Na_2SO_4), filtered, and was concentrated in vacuo. The residue was purified by flash chromatography with silica (SiO_2) that was neutralized by treatment with 2% Et_3N in acetone.

Synthesized **4e**[7b], **4f**[7c], **4h**[7d] had matching spectral data with their respective literature data.

4. Representative Procedure

Method 1

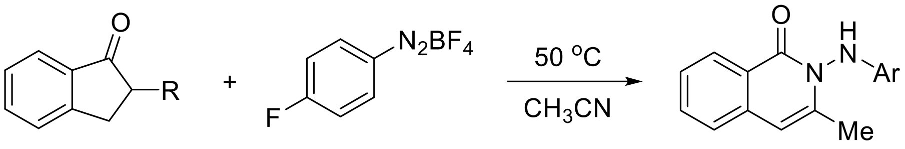

To a suspension of diazonium salt **2a** (0.2 mmol) in CH_3CN (0.5 mL) was added indanone derivative **1a** (0.1 mmol) at room temperature. The mixture was then heated to 50 °C until the completion of the reaction by TLC. Upon completion of the reaction, the mixture was cooled to RT and concentrated in vacuo. The residue was purified by flash chromatography (EtOAc/Hex).

Method 2

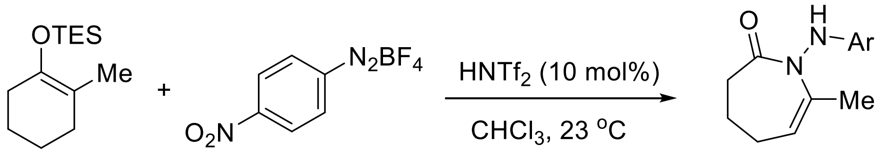

To a suspension of diazonium salt **2c** (0.2 mmol) and $HNTf_2$ (10 mol%) in $CHCl_3$ (2 mL) was added silyl enol ether **4e** (0.1 mmol) at room temperature. The reaction mixture was stirred at 25 °C and monitored by TLC. Upon completion of the reaction, the mixture was quenched with Et_3N (10 μl) and concentrated in vacuo. The residue was purified by flash chromatography (EtOAc/Hex).

5. Characterization of New Compounds

5.1. Characterization of substrates

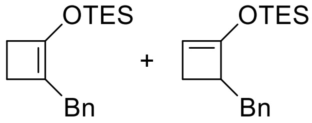

4a+**4a'** (obtained as an inseparable mixture of **4a**:**4a'** = 6.7:1)

4a (**GP-E1**, 30%, EtOAc/nHex = 1:20, R_f = 0.8) as a colorless oil; ^{1}H NMR (400 MHz, $CDCl_3$): δ 7.31-7.13 (m, 5H), 3.33 (s, 2H), 2.53-2.44 (m, 2H), 1.94-1.85 (m, 2H), 0.98 (t, 8.0 Hz, 9H), 0.66 (q, J = 7.7 Hz, 6H); ^{13}C NMR (100 MHz, $CDCl_3$): δ 141.4, 139.9, 128.7, 128.2, 125.6, 115.5, 32.8, 32.4, 21.8, 6.6, 5.2; IR (ATR): ṽ = 3035, 2963, 2875, 1779, 1712, 1454, 1239, 1177, 1063, 1005, 827, 740, 700 cm^{-1}; HRMS (EI) Calcd for $C_{17}H_{27}OSi^+$ $[M+H]^+$ 275.1826; found 275.1828.

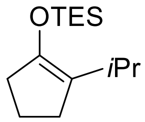

4c

4c (**GP-E1**, 30%, EtOAc/nHex = 1:20, R_f = 0.7) as a colorless oil; ^{1}H NMR (400 MHz, $CDCl_3$): δ 2.76 (septet, J = 6.8 Hz, 1H), 2.33-2.24 (m, 2H), 2.21-2.11 (m, 2H), 1.82-1.71 (m, 2H), 0.98 (t, 8.0 Hz, 9H), 0.94 (d, J = 6.9 Hz, 6H), 0.64 (q, J = 7.8 Hz, 6H); ^{13}C NMR (100 MHz, $CDCl_3$): δ 144.5, 122.3, 34.0, 26.8, 24.8, 21.0, 19.8, 6.7, 5.4; IR (ATR): ṽ = 2955, 2877, 2846, 1677, 1460, 1413, 1342, 1219, 1160, 1016, 973, 883, 843, 727 cm^{-1}; HRMS (EI) Calcd for $C_{14}H_{29}OSi^+$ $[M+H]^+$ 241.1982; found 241.1982.

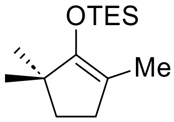

4d

4d (**GP-E1**, 24%, EtOAc/nHex = 1:20, R_f = 0.7) as a colorless oil; ^{1}H NMR (400 MHz, $CDCl_3$): δ 2.08 (t, J = 7.0 Hz, 2H), 1.62 (t, J = 7.2 Hz, 2H), 1.53 (s, 3H), 1.00 (s, 6H), 1.00 (t, 7.9 Hz, 9H), 0.69 (q, J = 7.9 Hz, 6H),; ^{13}C NMR (100 MHz, $CDCl_3$): δ 153.4, 109.0, 43.4, 36.9, 30.6, 26.4, 12.9, 6.6, 6.0; IR (ATR): ṽ = 2955, 8278, 1683, 1459, 1414, 1384, 1325, 1284, 1223, 1144, 1006, 902, 842, 728 cm^{-1}; HRMS (EI) Calcd for $C_{14}H_{29}OSi^+$ $[M+H]^+$ 241.1982; found 241.1984.

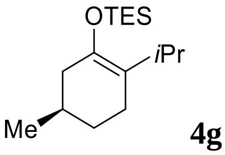

4g

4g (**GP-E1**, 79%, EtOAc/nHex = 1:20, R_f = 0.7) as a colorless oil; ^{1}H NMR (400 MHz, $CDCl_3$): δ 3.08 (septet, J = 7.0 Hz, 1H), 2.13-2.01 (m, 1H), 2.01-1.84 (m, 2H), 1.79-1.59 (m, 3H), 1.16-1.04 (m, 1H), 0.99 (t, J = 8.0 Hz, 9H), 0.95 (d, J = 6.2 Hz, 3H), 0.91 (dd, J = 6.9, 1.8 Hz, 6H), 0.65 (q, J = 7.7 Hz, 6H); ^{13}C NMR (100 MHz, $CDCl_3$): δ 140.9, 119.8, 38.9, 31.1, 29.6, 25.8, 21.6, 21.5, 20.5, 20.2, 6.9, 5.7; IR (ATR): ṽ = 2955, 2911, 2877, 1671, 1457, 1413, 1379, 1353, 1239, 1186, 1161, 1004, 959, 922, 883, 803, 726 cm^{-1}; HRMS (EI) Calcd for $C_{16}H_{33}OSi^+$ $[M+H]^+$ 269.2295; found 269.2296.

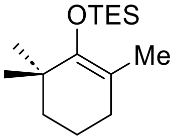

4i

4i (**GP-E2**, 61%, EtOAc/nHex = 1:20, R_f = 0.7) as a colorless oil; ^{1}H NMR (400 MHz, $CDCl_3$): δ 1.94 (t, J = 5.7 Hz, 1H), 1.53 (s, 3H), 1.59-1.44 (m, 4H), 1.02 (s, 6H), 1.00 (t, J = 7.9 Hz, 9H), 0.72 (q, J = 7.9 Hz, 6H); ^{13}C NMR (100 MHz, $CDCl_3$): δ 150.0, 109.6, 40.1, 35.2, 31.7, 27.6, 19.5, 17.7, 7.1, 6.0; IR (ATR): ṽ = 2955, 2913, 2877, 1668, 1458, 1382, 1299, 1239, 1168, 1006, 975, 915, 861, 824, 776, 728, 686 cm^{-1}; HRMS (EI) Calcd for $C_{15}H_{31}OSi^+$ $[M+H]^+$ 255.2139; found 255.2139.

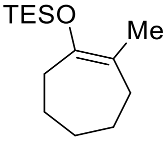

4k

4k (**GP-E2**, 52%, EtOAc/nHex = 1:20, R_f = 0.7) as a colorless oil; ^{1}H NMR (400 MHz, $CDCl_3$): δ 2.30-2.20 (m, 2H), 2.05-1.95 (m, 2H), 1.71-1.59 (m, 2H), 1.62 (s, 3H), 1.59-1.44 (m, 4H), 1.94 (t, J = 5.7 Hz, 1H), 1.53 (s, 3H), 1.59-1.44 (m, 4H), 0.99 (t, J = 8.0 Hz, 9H), 0.65 (q, J = 7.8 Hz, 6H); ^{13}C NMR (100 MHz, $CDCl_3$): δ 148.1, 116.2, 35.1, 32.7, 31.5, 26.5, 25.5, 18.6,

6.8, 5.5; IR (ATR): ṽ = 2916, 2877, 2849, 1677, 1455, 1376, 1347, 1243, 1170, 1070, 1005, 938, 880, 725 cm^{-1}; HRMS (EI) Calcd for $C_{14}H_{29}OSi^+$ $[M+H]^+$ 241.1982; found 241.1984.

5.2. Characterization of products

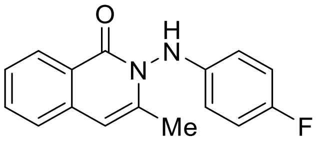

3aa

3aa (90%, EtOAc/nHex = 1:3, R_f = 0.4) as an off-white solid; mp: 162-166 °C; ^1H NMR (400 MHz, $CDCl_3$): δ 8.33 (d, *J* = 8.0 Hz, 1H), 7.65 (t, *J* = 8.1 Hz, 1H), 7.49 (d, *J* = 7.9 Hz, 1H), 7.43 (t, *J* = 8.0 Hz, 1H), 7.33 (s, 1H), 6.91 (t, *J* = 8.7 Hz, 2H), 6.65 (dd, *J* = 8.9, 4.4 Hz, 2H), 6.44 (s, 1H), 2.41 (s, 3H); ^{13}C NMR (100 MHz, $CDCl_3$): δ 162.2, 158.4 (d, *J* = 238.4 Hz), 143.2 (d, *J* = 2.2 Hz), 141.8, 136.7, 132.7, 127.9, 126.0, 125.4, 124.5, 116.0 (d, *J* = 22.7 Hz), 115.3 (d, *J* = 7.9 Hz), 105.0, 19.0; IR (ATR): ṽ = 3264, 2923, 1660, 1626, 1597, 1508, 1215, 874, 826, 755, 697 cm^{-1}; HRMS (EI) Calcd for $C_{16}H_{13}FN_2NaO^+$ $[M+Na]^+$ 291.0904; found 291.0906.

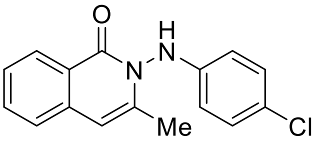

3ab

3aa (95%, EtOAc/nHex = 1:3, R_f = 0.4) as an off-white solid; mp: 192-198 °C; ^1H NMR (400 MHz, $CDCl_3$): δ 8.33 (d, *J* = 8.0 Hz, 1H), 7.65 (t, *J* = 8.4 Hz, 1H), 7.49 (d, *J* = 8.0 Hz, 1H), 7.46-7.39 (m, 2H), 7.16 (d, *J* = 8.8 Hz, 2H), 6.61 (d, *J* = 8.8 Hz, 2H), 6.44 (s, 1H), 2.40 (s, 3H); ^{13}C NMR (100 MHz, $CDCl_3$): δ 162.2, 145.9, 141.9, 136.8, 132.8, 129.2, 127.9, 126.8, 126.0, 125.4, 124.5, 115.1, 105.0, 18.9; IR (ATR): ṽ = 2981, 1660, 1632, 1595, 1377, 1240, 1105, 1043, 936, 846, 753 cm^{-1}; HRMS (EI) Calcd for $C_{16}H_{13}ClN_2NaO^+$ $[M+Na]^+$ 307.0609; found 307.0613.

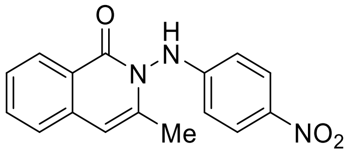

3ac

3ac (90%, EtOAc/nHex = 1:3, $R_f = 0.2$) as a yellow solid; mp: 198-204 °C; ^{1}H NMR (400 MHz, $CDCl_3$): δ 8.33 (d, *J* = 8.1 Hz, 1H), 8.10 (d, *J* = 9.1 Hz, 2H), 7.72-7.67 (m, 2H), 7.52 (d, *J* = 7.9 Hz, 1H), 7.47 (t, *J* = 8.0 Hz, 1H), 6.73 (d, *J* = 9.1 Hz, 2H), 6.49 (s, 1H), 2.40 (s, 3H); ^{13}C NMR (100 MHz, $CDCl_3$): δ 162.3, 153.0, 141.7, 141.6, 136.8, 133.3, 127.8, 126.4, 125.72, 125.66, 124.4, 112.5, 105.8, 18.9; IR (ATR): ṽ = 3231, 1657, 1629, 1594, 1507, 1330, 1274, 1180, 1110, 842, 765, 751, 696 cm^{-1}; HRMS (EI) Calcd for $C_{16}H_{14}N_3O_3^+$ $[M+H]^+$ 296.1030; found 296.1033.

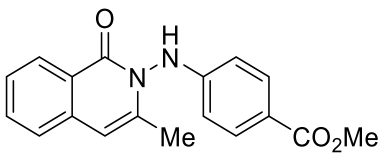

3ad

3ad (75%, EtOAc/nHex = 1:3, $R_f = 0.4$) as an off-white solid; mp: 171-174 °C; ^{1}H NMR (400 MHz, $CDCl_3$): δ 8.33 (d, *J* = 8.0 Hz, 1H), 7.90 (d, *J* = 8.9 Hz, 2H), 7.67 (t, *J* = 8.2 Hz, 1H), 7.47 (br, s, 1H), 7.50 (d, *J* = 7.9 Hz, 1H), 7.44 (d, *J* = 8.1 Hz, 1H), 6.70 (d, *J* = 8.9 Hz, 2H), 6.46 (s, 1H), 3.86 (s, 3H), 2.40 (s, 3H); ^{13}C NMR (100 MHz, $CDCl_3$): δ 166.7, 162.2, 151.5, 141.8, 136.8, 133.0, 131.4, 128.0, 126.2, 125.5, 124.6, 123.7, 113.0, 105.2, 51.9, 19.0; IR (ATR): ṽ = 3250, 3007, 2955, 1712, 1660, 1606, 1434, 1276, 1174, 1109, 769, 695 cm^{-1}; HRMS (EI) Calcd for $C_{18}H_{17}N_2O_2^+$ $[M+H]^+$ 293.1285; found 293.1287.

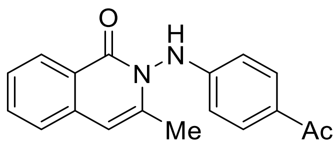

3ae

3ae (75%, EtOAc/nHex = 1:3, $R_f = 0.4$) as an off-white solid; mp: 181-184 °C; ^{1}H NMR (400 MHz, $CDCl_3$): δ 8.32 (d, *J* = 8.0 Hz, 1H), 7.85 (d, *J* = 8.7 Hz, 2H), 7.67 (t, *J* = 8.1 Hz, 1H), 7.51 (d, *J* = 8.0 Hz, 1H), 7.44 (t, *J* = 7.9 Hz, 1H), 7.41 (s, 1H), 6.72 (d, *J* = 8.7 Hz, 2H), 6.47 (s, 1H), 2.51 (s, 3H), 2.41 (s, 3H); ^{13}C NMR (100 MHz, $CDCl_3$): δ 196.6, 162.2, 151.6, 141.8, 136.8, 133.0, 131.4, 130.4, 128.0, 126.2, 125.5, 124.6, 113.0, 105.2, 26.3, 18.9; IR (ATR): ṽ = 3252, 3009, 1665, 1630, 1599, 1359, 1270, 1177, 960, 911, 836, 765, 734, 596 cm^{-1}; HRMS (EI) Calcd for $C_{18}H_{17}N_2O_2^+$ $[M+H]^+$ 293.1285; found 293.1287.

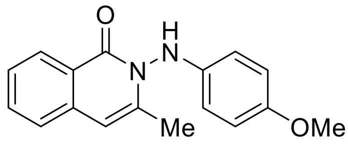

3af

3af (40%, EtOAc/nHex = 1:3, R_f = 0.3) as yellow oil; ^1H NMR (400 MHz, $CDCl_3$): δ 8.34 (d, *J* = 8.0 Hz, 1H), 7.64 (t, *J* = 8.0 Hz, 1H), 7.48 (d, *J* = 8.0 Hz, 1H), 7.42 (t, *J* = 8.0 Hz, 1H), 7.31 (s, 1H), 6.78 (d, *J* = 8.8 Hz, 2H), 6.67 (d, *J* = 8.8 Hz, 2H), 6.43 (s, 1H), 3.73 (s, 3H), 2.42 (s, 3H); ^{13}C NMR (100 MHz, $CDCl_3$): δ 162.3, 155.2, 142.0, 140.6, 136.8, 132.6, 127.9, 125.9, 125.4, 124.5, 115.6, 114.8, 104.8, 55.6, 19.1; IR (ATR): ṽ = 2925, 1664, 1608, 1570, 1479, 1350, 1326, 1263, 1162, 1068, 1027, 762, 689 cm^{-1}; HRMS (EI) Calcd for $C_{17}H_{16}N_2NaO_2^+$ $[M+Na]^+$ 303.1104; found 303.1108.

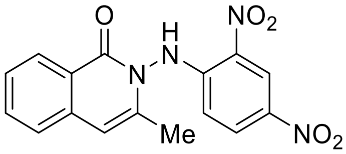

3ag

3ag (85%, EtOAc/nHex = 1:3, R_f = 0.2) as a yellow solid; mp: 162-164 °C; ^1H NMR (400 MHz, $CDCl_3$): δ 8.33 (d, *J* = 2.4 Hz, 1H), 8.30 (d, *J* = 8.1 Hz, 1H), 7.98 (dd, *J* = 9.0, 2.4 Hz, 1H), 7.69 (t, *J* = 8.1 Hz, 1H), 7.57 (s, 1H), 7.51 (d, *J* = 7.9 Hz, 1H), 7.46 (t, *J* = 8.2 Hz, 1H), 6.49 (s, 1H), 6.45 (d, *J* = 9.0 Hz, 1H), 2.41 (s, 3H); ^{13}C NMR (100 MHz, $CDCl_3$): δ 161.6, 148.9, 141.8, 141.1, 136.7, 133.3, 128.0, 126.5, 125.8, 125.6, 124.4, 124.0, 119.8, 111.8, 105.7, 18.8; IR (ATR): ṽ = 3245, 1671, 1633, 1587, 1504, 1333, 1291, 1124, 1047, 894, 828, 744, 692 cm^{-1}; HRMS (EI) Calcd for $C_{16}H_{13}N_4O_5^+$ 341.0880; found 341.0883.

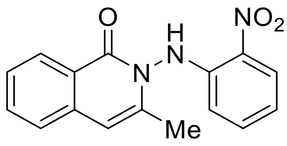

3ah

3ah (89%, EtOAc/nHex = 1:3, R_f = 0.3) as an off-white solid; mp: 162-164 °C; ^1H NMR (400 MHz, $CDCl_3$): δ 9.64 (s, 1H), 8.31 (d, *J* = 8.0 Hz, 1H), 8.27 (dd, *J* = 8.8, 1.6 Hz, 1H), 7.68 (t, *J* = 8.0 Hz, 1H), 7.50 (d, *J* = 7.6 Hz, 1H), 7.48-7.37 (m, 2H), 6.97 (t, *J* = 8.8 Hz, 1H), 6.55 (dd, *J* = 8.4, 0.8 Hz, 1H), 6.48 (s, 1H), 2.40 (s, 3H); ^{13}C NMR (100 MHz, $CDCl_3$): δ 161.7, 144.6, 141.7, 136.7, 136.2, 134.6, 133.1, 128.1, 126.7, 126.3, 125.5, 124.8, 120.4, 114.0, 105.4, 19.0;

IR (ATR): ṽ = 3314, 2991, 1674, 1630, 1661, 1527, 1492, 1421, 1337, 1279, 1261, 751 cm^{-1}; HRMS (EI) Calcd for $C_{16}H_{14}N_3O_3^+$ $[M+H]^+$ 296.1030; found 296.1031.

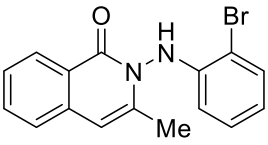

3ai

3ai (94%, EtOAc/nHex = 1:3, R_f = 0.4) as an off-white solid; mp: 174-178 °C; ^{1}H NMR (400 MHz, $CDCl_3$): δ 8.32 (d, *J* = 8.0 Hz, 1H), 7.65 (t, *J* = 8.1 Hz, 1H), 7.54 (d, *J* = 8.0 Hz, 1H), 7.49 (d, *J* = 7.9 Hz, 1H), 7.43 (t, *J* = 8.0 Hz, 1H), 7.39 (s, 1H), 7.09 (t, *J* = 8.2 Hz, 1H), 6.82 (t, *J* = 7.8 Hz, 1H), 6.46 (s, 1H), 6.33 (d, *J* = 8.1 Hz, 1H), 2.43 (s, 3H); ^{13}C NMR (100 MHz, $CDCl_3$): δ 161.8, 144.3, 142.0, 136.8, 132.9, 132.8, 128.4, 128.0, 126.0, 125.4, 124.6, 122.9, 113.2, 110.1, 104.9, 19.0; IR (ATR): ṽ = 3310, 2987, 1670, 1630, 1599, 1483, 1280, 1031, 751 cm^{-1}; HRMS (EI) Calcd for $C_{16}H_{14}BrN_2O^+$ $[M+H]^+$ 329.0284; found 329.0284.

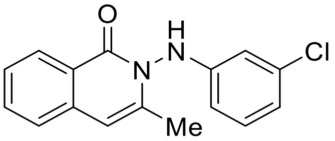

3aj

3aj (81%, EtOAc/nHex = 1:3, R_f = 0.4) as an off-white solid; mp: 176-179 °C; ^{1}H NMR (400 MHz, $CDCl_3$): δ 8.33 (d, *J* = 8.1 Hz, 1H), 7.66 (t, *J* = 8.1 Hz, 1H), 7.50 (d, *J* = 7.9 Hz, 1H), 7.44 (t, *J* = 7.9 Hz, 1H), 7.29-7.20 (m, 2H), 7.15 (t, *J* = 8.0 Hz, 1H), 6.92 (d, *J* = 7.9 Hz, 1H), 6.66 (t, *J* = 1.9 Hz, 1H), 6.61 (d, *J* = 8.2 Hz, 1H), 6.45 (s, 1H), 2.41 (s, 3H) ; ^{13}C NMR (100 MHz, $CDCl_3$): δ 162.2, 148.6, 141.8, 136.8, 135.3, 132.9, 130.5, 128.0, 126.1, 125.5, 124.5, 122.3, 114.0, 112.2, 105.1, 19.0; IR (ATR): ṽ = 3252, 2924, 1661, 1630, 1599, 1508, 1479, 1341, 1270, 765, 702 cm^{-1}; HRMS (EI) Calcd for $C_{16}H_{14}ClN_2O^+$ $[M+H]^+$ 285.0789; found 285.0791.

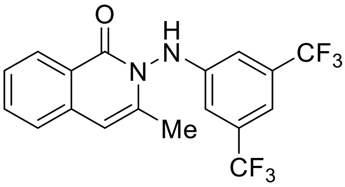

3ak

3ak (83%, EtOAc/nHex = 1:3, R_f = 0.3) as an off-white solid; mp: 221-224 °C; ^{1}H NMR (400 MHz, $CDCl_3$): δ 8.37 (d, *J* = 8.0 Hz, 1H), 7.99 (s, 1H), 7.71 (t, *J* = 8.0 Hz, 1H), 7.54 (d, *J* = 8.0 Hz, 1H), 7.49 (t, *J* = 8.0 Hz, 1H), 7.40 (s, 1H), 7.05 (s, 2H), 6.52 (s, 1H), 2.40 (s, 3H) ; ^{13}C NMR (100 MHz, $CDCl_3$): δ 162.3, 148.9, 141.3, 136.9, 133.3, 132.8 (q, *J* = 33.1 Hz), 127.9, 126.4, 125.7, 124.4, 123.0 (q, *J* = 271.3 Hz), 115.3 (m, *J* = 4.2 Hz), 113.1 (q, *J* = 4.4 Hz), 105.9, 18.9; IR (ATR): ṽ = 3230, 2991, 1665, 1629, 1386, 1284, 1266, 1173, 1129, 876, 765, 752 cm^{-1}; HRMS (EI) Calcd for $C_{18}H_{13}F_6N_2O^+$ $[M+H]^+$ 387.0927; found 387.0930.

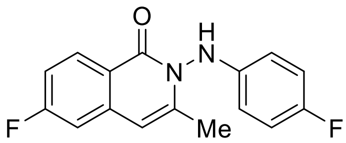

3ba

3ba (88%, EtOAc/nHex = 1:3, R_f = 0.4) as an off-white solid; mp: 193-195 °C; ^{1}H NMR (400 MHz, $CDCl_3$): δ 8.38-8.29 (m, 1H), 7.31 (br, s, 1H), 7.17-7.07 (m, 2H), 6.92 (t, *J* = 8.4 Hz, 2H), 6.65 (dd, *J* = 8.8, 4.4 Hz, 2H), 6.38 (s, 1H), 2.40 (s, 3H); ^{13}C NMR (100 MHz, $CDCl_3$): δ 165.5 (d, *J* = 251.5 Hz), 161.5, 158.4 (d, *J* = 238.5 Hz), 143.4, 143.0 (d, *J* = 2.2 Hz), 139.0 (d, *J* = 10.7 Hz), 131.1 (d, *J* = 10.1 Hz), 121.1 (d, *J* = 1.3 Hz), 115.9 (d, *J* = 22.7 Hz), 115.3 (d, *J* = 7.9 Hz), 114.9 (d, *J* = 23.6 Hz), 110.1 (d, *J* = 21.8 Hz), 104.3 (d, *J* = 3.1 Hz), 19.1; IR (ATR): ṽ = 3248, 2929, 1661, 1629, 1616, 1505, 1483, 1217, 1160, 880, 831, 752, 663 cm^{-1}; HRMS (EI) Calcd for $C_{16}H_{12}F_2N_2NaO^+$ $[M+Na]^+$ 309.0810; found 309.0813.

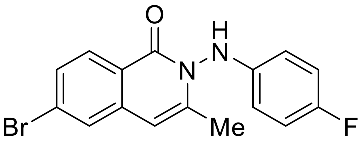

3ca

3ca (96%, EtOAc/nHex = 1:3, R_f = 0.4) as an off-white solid; mp: 178-181 °C; ^{1}H NMR (400 MHz, $CDCl_3$): δ 8.17 (d, *J* = 8.4 Hz, 1H), 7.65 (d, *J* = 1.6 Hz, 1H), 7.51 (dd, *J* = 8.4, 2.0 Hz, 1H), 7.30 (br, s, 1H), 6.92 (t, *J* = 8.4 Hz, 2H), 6.64 (dd, *J* = 9.2, 4.4 Hz, 2H), 6.34 (s, 1H), 2.40 (s, 3H); ^{13}C NMR (100 MHz, $CDCl_3$): δ 161.8, 158.5 (d, *J* = 238.7 Hz), 143.5, 142.8 (d, *J* = 2.5 Hz), 138.1, 129.7, 129.3, 127.9, 127.8, 123.1, 116.0 (d, *J* = 22.7 Hz), 115.3 (d, *J* = 7.8 Hz), 103.8, 19.1; IR (ATR): ṽ = 3266, 2924, 1661, 1625, 1594, 1510, 1226, 831, 765, 747, 685 cm^{-1}; HRMS (EI) Calcd for for $C_{16}H_{12}BrFN_2NaO^+$ $[M+Na]^+$ 369.0009; found 369.0010.

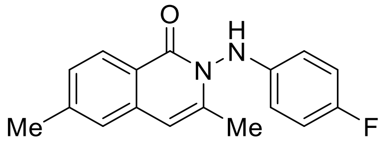

3da

3da (92%, EtOAc/nHex = 1:3, R_f = 0.5) as an off-white solid; mp: 178-180 °C; ^{1}H NMR (400 MHz, $CDCl_3$): δ 8.22 (d, *J* = 8.4 Hz, 1H), 7.31-7.21 (m, 2H), 6.91 (t, *J* = 8.4 Hz, 2H), 6.65 (dd, *J* = 9.2, 4.4 Hz, 2H), 6.37 (s, 1H), 2.49 (s, 3H), 2.39 (s, 3H); ^{13}C NMR (100 MHz, $CDCl_3$): δ 162.1, 158.4 (d, *J* = 238.2 Hz), 143.4, 143.3 (d, *J* = 2.2 Hz), 141.8, 136.9, 127.8, 127.7, 125.1, 122.3, 115.9 (d, *J* = 22.7 Hz), 115.3 (d, *J* = 7.8 Hz), 104.7, 21.9, 19.0; IR (ATR): ṽ = 3244, 2929, 1656, 1630, 1599, 1386, 1306, 1213, 1155, 885, 831, 734, 658 cm^{-1}; HRMS (EI) Calcd for $C_{17}H_{16}FN_2O^+$ $[M+H]^+$ 283.1241; found 283.1243.

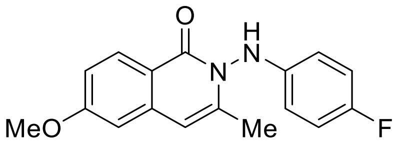

3ea

3ea (95%, EtOAc/nHex = 1:3, R_f = 0.3) as an off-white solid; mp: 204-208 °C; ^{1}H NMR (400 MHz, $CDCl_3$): δ 8.24 (d, *J* = 8.8 Hz, 1H), 7.35 (br, s, 1H), 7.00 (dd, *J* = 8.8, 2.4 Hz, 1H), 6.90 (t, *J* = 8.4 Hz, 2H), 6.83 (d, *J* = 2.4 Hz, 1H), 6.65 (dd, *J* = 8.8, 4.4 Hz, 2H), 6.35 (s, 1H), 3.91 (s, 3H), 2.38 (s, 3H); ^{13}C NMR (100 MHz, $CDCl_3$): δ 163.1, 161.8, 158.3 (d, *J* = 237.8 Hz), 143.3 (d, *J* = 2.2 Hz), 142.5, 138.9, 129.9, 118.3, 116.0, 115.7 (d, *J* = 13.3 Hz), 115.2 (d, *J* = 7.9 Hz), 106.1, 104.6, 55.4, 19.0; IR (ATR): ṽ = 3244, 2929, 1656, 1594, 1505, 1279, 1262, 1244, 1213, 1017, 831, 752 cm^{-1}; HRMS (EI) Calcd for $C_{17}H_{16}FN_2O_2^+$ $[M+H]^+$ 299.1190; found 299.1195.

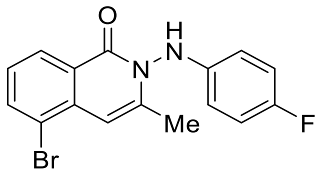

3fa

3af (78%, EtOAc/nHex = 1:3, R_f = 0.4) as an off-white solid; mp: 172-174 °C; ^{1}H NMR (400 MHz, $CDCl_3$): δ 8.31 (d, *J* = 8.0 Hz, 1H), 7.90 (d, *J* = 7.6 Hz, 1H), 7.32 (br, s, 1H), 7.27 (t, *J*

= 7.6 Hz, 1H), 6.93 (t, *J* = 8.4 Hz, 2H), 6.80 (s, 1H), 6.70-6.61 (m, 2H), 2.46 (s, 3H); ^{13}C NMR (100 MHz, $CDCl_3$): δ 161.4, 158.5 (d, *J* = 238.6 Hz), 143.3, 142.7 (d, *J* = 2.3 Hz), 136.5, 135.8, 127.6, 126.5, 125.9, 120.0, 116.0 (d, *J* = 22.7 Hz), 115.4 (d, *J* = 7.9 Hz), 103.7, 19.3; IR (ATR): ṽ = 3252, 2924, 1665, 1625, 1590, 1505, 1386, 1208, 880, 831, 800, 756, 729, 658 cm^{-1}; HRMS (EI) Calcd for $C_{16}H_{12}BrFN_2NaO^+$ $[M+Na]^+$ 369.0009; found 369.0013.

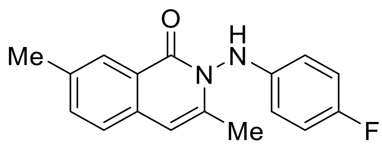

3ga

3ga (93%, EtOAc/nHex = 1:3, R_f = 0.5) as an off-white solid; mp: 196-198 ^{o}C; ^{1}H NMR (400 MHz, $CDCl_3$): δ 8.13 (d, *J* = 1.2 Hz, 1H), 7.48 (dd, *J* = 8.4, 2.0 Hz, 1H), 7.40 (d, *J* = 8.0 Hz, 1H), 6.91 (t, *J* = 8.8 Hz, 2H), 6.65 (d, *J* = 8.8, 4.4 Hz, 2H), 6.41 (s, 1H), 2.46 (s, 3H), 2.39 (s, 3H); ^{13}C NMR (100 MHz, $CDCl_3$): δ 162.1, 158.4 (d, *J* = 238.0 Hz), 143.2 (d, *J* = 2.3 Hz), 140.7, 136.0, 134.4, 134.3, 127.3, 125.3, 124.4, 115.9 (d, *J* = 22.8 Hz), 115.3 (d, *J* = 7.8 Hz), 104.8, 21.4, 18.9; IR (ATR): ṽ = 3239, 2920, 1652, 1630, 1603, 1505, 1346, 1221, 1155, 845, 823, 734, 671, 596 cm^{-1}; HRMS (EI) Calcd for $C_{17}H_{16}FN_2O^+$ $[M+H]^+$ 283.1241; found 283.1246.

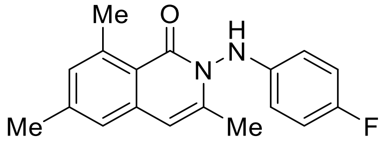

3ha

3ha (87%, EtOAc/nHex = 1:3, R_f = 0.6) as a colorless oil; ^{1}H NMR (400 MHz, $CDCl_3$): δ 7.25 (s, 1H), 7.09 (s, 1H), 7.00 (s, 1H), 6.92 (t, *J* = 8.8 Hz, 2H), 6.71-6.59 (m, 2H), 6.29 (s, 1H), 2.81 (s, 3H), 2.41 (s, 3H), 2.35 (s, 3H); ^{13}C NMR (100 MHz, $CDCl_3$): δ 162.7, 158.3 (d, *J* = 237.8 Hz), 143.6 (d, *J* = 2.2 Hz), 142.5, 141.7, 141.5, 138.6, 130.6, 123.5, 120.7, 115.9 (d, *J* = 22.8 Hz), 115.1 (d, *J* = 7.8 Hz), 105.0, 23.3, 21.5, 18.9; IR (ATR): ṽ = 3269, 2925, 1661, 1622, 1612, 1508, 1230, 1220, 859, 827 cm^{-1}; HRMS (EI) Calcd for $C_{18}H_{18}FN_2O^+$ $[M+H]^+$ 297.1398; found 297.1401

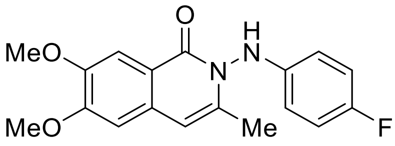

3ia

3ia (95%, EtOAc/nHex = 1:3, R_f = 0.2) as an off-white solid; mp: 247-250 °C; ^{1}H NMR (400 MHz, $CDCl_3$): δ 7.68 (s, 1H), 7.32 (br, s, 1H), 6.90 (t, *J* = 8.4 Hz, 2H), 6.83 (s, 1H), 6.69-6.58 (m, 2H), 6.35 (s, 1H), 3.99 (s, 3H), 3.95 (s, 3H), 2.38 (s, 3H); ^{13}C NMR (100 MHz, $CDCl_3$): δ 161.5, 158.4 (d, *J* = 238.2 Hz), 153.9, 148.6, 143.3 (d, *J* = 1.7 Hz), 140.2, 132.4, 118.2, 115.9 (d, *J* = 22.7 Hz), 115.3 (d, *J* = 7.7 Hz), 107.6, 105.4, 104.4, 56.1, 18.9; IR (ATR): ṽ = 3248, 2929, 1656, 1594, 1510, 1461, 1279, 1261, 1213, 1146, 1027, 853,769, 751, 720 cm^{-1}; HRMS (EI) Calcd for $C_{18}H_{18}FN_2O_3^+$ $[M+H]^+$ 329.1296; found 329.1297.

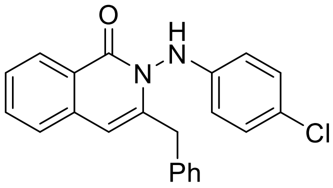

3jb

3jb (93%, EtOAc/nHex = 1:3, R_f = 0.6) as an off-white solid; mp: 164-166 °C; ^{1}H NMR (400 MHz, $CDCl_3$): δ 8.30 (d, *J* = 8.0 Hz, 1H), 7.64 (t, *J* = 8.0 Hz, 1H), 7.46 (d, *J* = 8.0 Hz, 1H), 7.43 (t, *J* = 8.0 Hz, 1H), 7.33-7.17 (m, 6H), 7.13 (d, *J* = 8.8 Hz, 2H), 6.57 (d, *J* = 8.8 Hz, 2H), 6.29 (s, 1H), 4.10 (d of ABq, *J* = 16.0 Hz, 1H), 3.89 (d of ABq, *J* = 16.0 Hz, 1H); ^{13}C NMR (100 MHz, $CDCl_3$): δ 162.3, 145.8, 144.6, 137.0, 136.5, 132.9, 129.23, 129.17, 128.5, 127.9, 126.8, 126.3, 125.9, 124.7, 115.0, 106.0, 38.2; IR (ATR): ṽ = 3260, 3030, 1661, 1626, 1601, 1490, 1340, 1263, 1096, 825, 755, 699 cm^{-1}; HRMS (EI) Calcd for $C_{22}H_{18}ClN_2O^+$ $[M+H]^+$ 361.1102; found 361.1104.

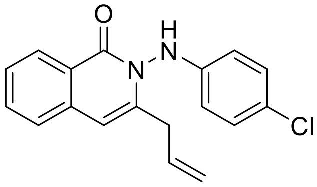

3kb

3kb (53%, EtOAc/nHex = 1:3, R_f = 0.6) as a yellow solid; mp: 130-132 °C; ^{1}H NMR (400 MHz, $CDCl_3$): δ 8.33 (d, *J* = 8.0 Hz, 1H), 7.66 (t, *J* = 8.1 Hz, 1H), 7.52 (d, *J* = 7.9 Hz, 1H), 7.44 (t, *J* = 8.0 Hz, 1H), 7.33 (s, 1H), 7.16 (d, *J* = 8.8 Hz, 2H), 6.62 (d, *J* = 8.8 Hz, 2H), 6.43

(s, 1H), 5.98 (ddt, J = 17.0, 10.2, 6.6 Hz, 1H), 5.21 (d, J = 10.1 Hz, 1H), 5.18 (d, J = 16.9 Hz, 1H), 3.61 (dd of ABq, J = 16.2, 5.7 Hz, 1H), 3.32 (dd of ABq, J = 16.6, 6.2 Hz, 1H); ^{13}C NMR (100 MHz, $CDCl_3$): δ 162.3, 145.9, 143.7, 136.6, 133.3, 132.9, 129.3, 127.9, 126.9, 126.3, 125.8, 124.7, 118.4, 115.1, 104.8, 35.8; IR (ATR): ṽ = 3253, 1659, 1626, 1600, 1490, 1264, 1091, 828, 758, 741 cm^{-1}; HRMS (EI) Calcd for $C_{18}H_{16}ClN_2O^+$ $[M+H]^+$ 311.0946; found 311.0948.

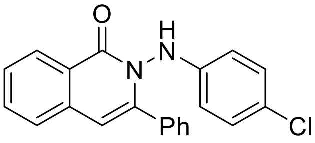

3lb

3lb (70%, EtOAc/nHex = 1:3, R_f = 0.6) as an off-white solid; mp: 196-200 °C; ^{1}H NMR (400 MHz, $CDCl_3$): δ 8.40 (d, J = 8.0 Hz, 1H), 7.71 (t, J = 8.0 Hz, 1H), 7.60 (d, J = 8.0 Hz, 1H), 7.56-7.46 (m, 3H), 7.42-7.31 (m, 3H), 7.20 (s, 1H), 7.09 (d, J = 8.8 Hz, 2H), 6.61 (s, 1H), 6.58 (d, J = 8.8 Hz, 2H); ^{13}C NMR (100 MHz, $CDCl_3$): δ 162.2, 145.9, 144.9, 136.5, 134.3, 133.1, 129.2, 129.1, 128.9, 128.1, 127.9, 126.87, 126.85, 126.3, 125.1, 115.5, 107.5; IR (ATR): ṽ = 3256, 3026, 1654, 1619, 1594, 1493, 1277, 1260, 825, 755, 699 cm^{-1}; HRMS (EI) Calcd for $C_{21}H_{16}ClN_2O^+$ $[M+H]^+$ 347.0946; found 347.0949.

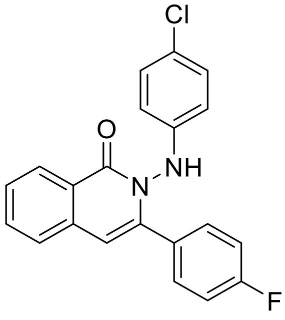

3mb

3mb (65%, EtOAc/nHex = 1:3, R_f = 0.5) as an off-white solid; mp: 180-184 °C; ^{1}H NMR (400 MHz, $CDCl_3$): δ 8.39 (d, J = 8.0 Hz, 1H), 7.71 (t, J = 8.4 Hz, 1H), 7.59 (d, J = 7.6 Hz, 1H), 7.56-7.46 (m, 3H), 7.24 (s, 1H), 7.09 (d, J = 9.2 Hz, 2H), 7.03 (t, J = 8.8 Hz, 2H), 6.59 (s, 1H), 6.55 (d, J = 8.8 Hz, 2H); ^{13}C NMR (100 MHz, $CDCl_3$): δ 163.0 (d, J = 248.0 Hz), 162.1, 145.7, 143.8, 136.3, 133.1, 131.1 (d, J = 8.4 Hz), 130.2 (d, J = 3.6 Hz), 129.1, 128.1, 127.04, 127.01, 126.3, 125.1, 115.5, 115.0 (d, J = 21.7 Hz), 107.5; IR (ATR): ṽ = 3267, 3022, 1657, 1619, 1598, 1511, 1490, 1260, 1232, 1159, 1096, 824, 751 cm^{-1}; HRMS (EI) Calcd for

$C_{21}H_{14}ClFN_2NaO^+$ $[M+Na]^+$ 387.0671; found 387.0681.

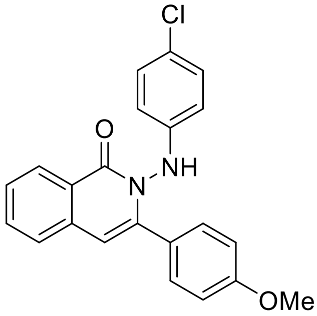

3nb

3nb (80%, EtOAc/nHex = 1:3, R_f = 0.3) as a yellow oil; ^{1}H NMR (400 MHz, $CDCl_3$): δ 8.38 (d, *J* = 8.0 Hz, 1H), 7.70 (t, *J* = 8.0 Hz, 1H), 7.58 (d, *J* = 7.6 Hz, 1H), 7.53-7.43 (m, 3H), 7.18 (s, 1H), 7.09 (d, *J* = 8.8 Hz, 2H), 6.86 (d, *J* = 8.8 Hz, 2H), 6.61-6.55 (m, 3H), 3.81 (s, 3H); ^{13}C NMR (100 MHz, $CDCl_3$): δ 162.3, 160.1, 146.0, 144.7, 136.6, 133.0, 130.6, 129.1, 128.1, 126.9, 126.7, 126.6, 126.2, 125.0, 115.6, 113.3, 107.1, 55.3; IR (ATR): ṽ = 3257, 2927, 2855, 1650, 1606, 1511, 1489, 1290, 1248, 1177, 1030, 822, 735 cm^{-1}; HRMS (EI) Calcd for $C_{22}H_{18}ClN_2O_2^+$ 377.1051; found 377.1054.

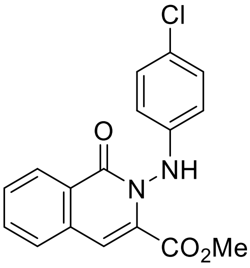

3ob

3ob (50%, EtOAc/nHex = 1:3, R_f = 0.3) as an off-white solid; mp: 152-156 ^{o}C; ^{1}H NMR (400 MHz, $CDCl_3$): δ 8.41 (d, *J* = 8.0 Hz, 1H), 7.75 (t, *J* = 8.1 Hz, 1H), 7.64 (d, *J* = 7.8 Hz, 1H), 7.61 (t, *J* = 8.1 Hz, 1H), 7.18 (d, *J* = 8.8 Hz, 3H), 7.13 (s, 1H), 7.05 (s, 1H), 6.72 (d, *J* = 8.8 Hz, 2H), 3.85 (s, 3H); ^{13}C NMR (100 MHz, $CDCl_3$): δ 162.3, 161.6, 145.7, 135.5, 134.7, 133.4, 129.1, 128.9, 128.4, 127.4, 127.1, 127.0, 115.9, 110.0, 53.1; IR (ATR): ṽ = 3253, 1743, 1669, 1596, 1476, 1433, 1313, 1264, 1217, 1170, 768, 748 cm^{-1}; HRMS (EI) Calcd for for $C_{17}H_{14}ClN_2O_3^+$ $[M+H]^+$ 329.0687; found 329.0690.

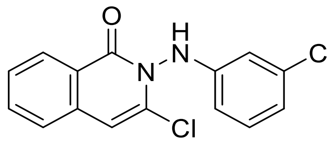

3pj

3pj (23%, EtOAc/nHex = 1:3, R_f = 0.45) as a yellow solid; mp: 187-190 °C; ^{1}H NMR (400 MHz, $CDCl_3$): δ 8.33 (d, *J* = 8.1 Hz, 1H), 7.71 (t, *J* = 8.2 Hz, 1H), 7.57-7.46 (m, 2H), 7.23-7.13 (m, 2H), 6.96 (d, *J* = 7.9 Hz, 1H), 6.77 (s, 1H), 6.74 (t, *J* = 2.1 Hz, 1H), 6.69 (dd, *J* = 8.2, 2.0 Hz, 1H); ^{13}C NMR (100 MHz, $CDCl_3$): δ 161.9, 147.9, 135.8, 135.2, 133.7, 133.6, 130.4, 128.4, 127.3, 125.6, 124.4, 122.6, 114.3, 112.5, 106.4; IR (ATR): ṽ = 3253, 1639, 1620, 1593, 1516, 1476, 1340, 1174, 928, 824, 801, 774, 755 cm^{-1}; HRMS (EI) Calcd for $C_{15}H_{11}Cl_2N_2O^+$ $[M+H]^+$ 305.0243; found 305.0245.

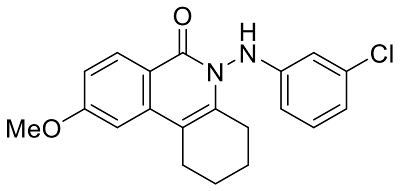

3qj

3qj (82%, EtOAc/nHex = 1:3, R_f = 0.4) as an off-white solid; mp: 190-192 °C; ^{1}H NMR (400 MHz, $CDCl_3$): δ 8.33 (d, *J* = 8.8 Hz, 1H), 7.31 (s, 1H), 7.11 (t, *J* = 8.0 Hz, 1H), 7.04 (dd, *J* = 9.2, 2.8 Hz, 1H), 6.99 (d, *J* = 2.4 Hz, 1H), 6.91-6.85 (m, 1H), 6.64 (t, *J* = 2.0 Hz, 1H), 6.58 (dd, *J* = 8.0, 2.4 Hz, 1H), 3.94 (s, 3H), 3.05-2.90 (m, 1H), 2.86-2.74 (m, 1H), 2.74-2.62 (m, 1H), 2.61-2.48 (m, 1H), 1.95-1.72 (m, 4H); ^{13}C NMR (100 MHz, $CDCl_3$): δ 163.2, 161.2, 149.0, 140.2, 139.1, 135.1, 130.31, 130.26, 121.7, 118.3, 114.7, 113.7, 112.0, 110.1, 103.7, 55.4, 25.5, 24.0, 22.3, 22.0; IR (ATR): ṽ = 3256, 2933, 1653, 1620, 1600, 1490, 1277, 1260, 1217, 765, 748 cm^{-1}; HRMS (EI) Calcd for $C_{20}H_{20}ClN_2O_2^+$ $[M+H]^+$ 355.1208; found 355.1210.

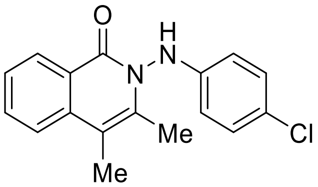

3rb

3rb (89%, EtOAc/nHex = 1:3, R_f = 0.5) as an off-white solid; mp: 224-226 °C; ^{1}H NMR (400 MHz, $CDCl_3$): δ 8.40 (d, *J* = 8.0 Hz, 1H), 7.73 (d, *J* = 4.3 Hz, 2H), 7.54-7.42 (m, 1H), 7.35 (s, 1H), 7.17 (d, *J* = 8.9 Hz, 2H), 6.63 (d, *J* = 8.9 Hz, 2H), 2.46 (s, 3H), 2.39 (s, 3H); ^{13}C NMR

(100 MHz, $CDCl_3$): δ 161.5, 146.1, 137.9, 137.2, 132.7, 129.3, 128.1, 126.9, 125.9, 124.5, 122.9, 115.3, 108.9, 15.2, 13.9; IR (ATR): ṽ = 3260, 3007, 1653, 1620, 1593, 1490, 1277, 1260, 765, 748 cm^{-1}; HRMS (EI) Calcd for $C_{17}H_{16}ClN_2O^+$ $[M+H]^+$ 299.0946; found 299.0949.

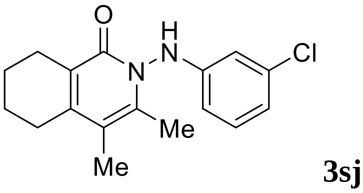

3sj

3sj (99%, EtOAc/nHex = 1:3, R_f = 0.5) as an off-white solid; mp: 238-240 °C; ^{1}H NMR (400 MHz, $CDCl_3$): δ 7.56 (br, s, 1H), 7.13 (t, *J* = 8.2 Hz, 1H), 6.90 (d, *J* = 7.2 Hz, 1H), 6.65-6.52 (m, 2H), 2.66-2.46 (m, 4H), 2.34 (s, 3H), 2.05 (s, 3H), 1.86-1.64 (s, 4H); ^{13}C NMR (100 MHz, $CDCl_3$): δ 160.9, 148.7, 147.7, 139.8, 135.0, 130.2, 124.7, 121.9, 114.1, 112.5, 112.4, 27.7, 24.0, 22.3, 21.6, 14.9, 13.7; IR (ATR): ṽ = 3220, 2934, 1643, 1603, 1580, 1543, 1480, 1277, 1260, 768, 748 cm^{-1}; HRMS (EI) Calcd for $C_{17}H_{20}ClN_2O^+$ $[M+H]^+$ 303.1259; found 303.1261.

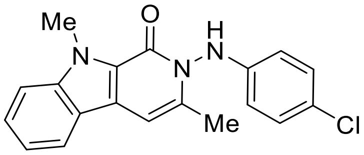

3tb

3tb (70%, EtOAc/nHex = 1:3, R_f = 0.4) as an off-white solid; mp: 201-203 °C; ^{1}H NMR (400 MHz, $CDCl_3$): δ 7.96 (d, *J* = 8.0 Hz, 1H), 7.53 (t, *J* = 8.4 Hz, 1H), 7.45 (d, *J* = 8.4 Hz, 1H), 7.38 (s, 1H), 7.27 (t, *J* = 8.0 Hz, 1H), 7.19 (d, *J* = 8.8 Hz, 2H), 6.85 (s, 1H), 6.62 (d, *J* = 8.8 Hz, 2H), 4.24 (s, 3H), 2.46 (s, 3H); ^{13}C NMR (100 MHz, $CDCl_3$): δ 156.2, 146.2, 141.3, 138.2, 129.4, 127.12, 127.05, 125.5124.8, 121.2, 121.1, 120.1, 115.1, 110.1, 99.2, 31.2, 19.2; IR (ATR): ṽ = 3246, 2920, 1663, 1590, 1570, 1493, 1330, 1267, 1237, 1094, 821, 807, 751 cm^{-1}; HRMS (EI) Calcd for $C_{19}H_{17}ClN_3O^+$ $[M+H]^+$ 338.1055; found 338.1057.

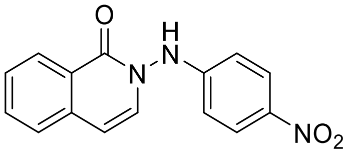

3uc

3uc (48%, EtOAc/nHex = 1:3, R_f = 0.2) as a yellow solid; mp: 224-227 °C; ^{1}H NMR (400 MHz, $CDCl_3$): δ 8.41 (d, *J* = 8.0 Hz, 1H), 8.11 (d, *J* = 9.1 Hz, 2H), 7.78 (s, 1H), 7.75 (t, *J* = 7.6 Hz, 1H), 7.63 (d, *J* = 7.8 Hz, 1H), 7.55 (t, *J* = 7.6 Hz, 1H), 7.31 (d, *J* = 7.5 Hz, 1H), 6.77 (d, *J* = Hz, 2H), 6.54 (d, *J* = 7.5 Hz, 1H); ^{13}C NMR (100 MHz, $CDCl_3$): δ 161.4, 152.9, 142.5, 137.0, 133.3, 133.2, 128.1, 127.5, 126.5, 126.3, 125.8, 113.3, 106.9; IR (ATR): ṽ = 3254, 3083, 2927, 2855, 1644, 1579, 1493, 1331, 1268, 836, 790, 750 cm^{-1}; HRMS (EI) Calcd for $C_{15}H_{12}N_3O_3^+$ $[M+H]^+$ 282.0873; found 282.0875.

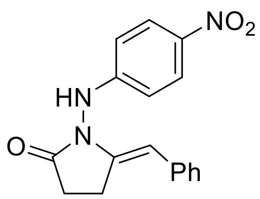

5ac

5cc (78%, EtOAc/nHex = 1:3, R_f = 0.3) as a yellow oil; ^{1}H NMR (400 MHz, $CDCl_3$): δ 8.13 (d, *J* = 9.1, 2H), 7.33 (t, *J* = 7.7 Hz , 2H), 7.24 (d, *J* = 7.4 Hz, 2H), 7.19 (d, *J* = 7.3 Hz, 1H), 6.83 (s, 1H), 6.79 (d, *J* = 9.1 Hz, 2H), 6.08 (s, 1H), 3.21 (t, *J* = 6.9 Hz, 2H), 2.88-2.57 (m, 2H); ^{13}C NMR (100 MHz, $CDCl_3$): δ 172.9, 150.9, 141.9, 137.8, 135.5, 128.7, 127.7, 126.2, 125.9, 112.6, 104.1, 27.1, 22.1; IR (ATR): ṽ = 2936, 2167, 1752, 1674, 1597, 1501, 1324, 1261, 1177, 1110, 911, 841, 752 cm^{-1}; HRMS (EI) Calcd for $C_{17}H_{16}N_3O_3^+$ $[M+H]^+$ 310.1186; found 310.1188; 1D-NOE experiments supported (E)-geometry of the double bond.

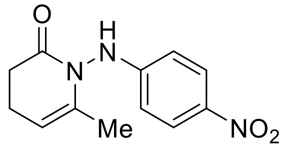

5bc

5bc (60%, EtOAc/nHex = 1:1, R_f = 0.4) as a yellow oil; ^{1}H NMR (400 MHz, $CDCl_3$): δ 8.15 (d, *J* = 9.1 Hz, 2H), 6.78 (d, *J* = 9.1 Hz, 2H), 6.72 (s, 1H), 5.13 (s, 1H), 2.75-2.67 (m, 2H), 2.49-2.25 (m, 2H), 1.89 (s, 3H); ^{13}C NMR (100 MHz, $CDCl_3$): δ 169.9, 153.7, 141.5, 137.9, 125.9, 112.0, 104.1, 31.8, 19.2, 17.7; IR (ATR): ṽ = 3279, 2931, 1683, 1595, 1503, 1329, 1276, 1183, 1111, 841, 752 cm^{-1}; HRMS (EI) Calcd for $C_{12}H_{14}N_3O_3^+$ $[M+H]^+$ 248.1030; found 248.1030.

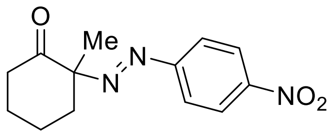

I-ec

I-ec (EtOAc/nHex = 1:5, R_f = 0.6) as a yellow solid; mp: 72-75 °C; ^{1}H NMR (400 MHz, $CDCl_3$): δ 8.34 (d, *J* = 9.0 Hz, 2H), 7.81 (d, *J* = 9.0 Hz, 2H), 2.82-2.73 (m, 1H), 2.56-2.46 (m, 2H), 2.15-2.03 (m, 1H), 2.01-1.91 (m, 1H), 1.91-1.73 (m, 3H), 1.36 (s, 3H); ^{13}C NMR (100 MHz, $CDCl_3$): δ 209.2, 154.8, 148.9, 124.7, 123.0, 82.0, 40.5, 39.9, 27.7, 21.8, 20.7; IR (ATR): ṽ 2933, 2868, 1717, 1614, 1526, 1448, 1345, 1316, 1120, 1090, 859, 753 cm^{-1}; HRMS (EI) Calcd for $C_{13}H_{16}N_3O_3^+$ $[M+H]^+$ 262.1186; found 262.1188.

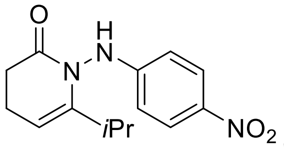

5cc

5cc (86%, EtOAc/nHex = 1:3, R_f = 0.3) as a yellow oil; ^{1}H NMR (400 MHz, $CDCl_3$): δ 8.12 (d, *J* = 9.1, 2H), 6.81 (s, 1H), 6.75 (d, *J* = 9.1 Hz, 2H), 5.24-5.15 (m, 1H), 2.71-2.37 (m, 4H), 2.36-2.23 (m, 1H), 1.07 (dd, *J* = 6.6, 2.0 Hz, 6H); ^{13}C NMR (100 MHz, $CDCl_3$): δ 170.5, 154.1, 147.9, 141.7, 125.9, 112.4, 101.8, 31.8, 27.9, 23.0, 20.3, 18.9; IR (ATR): ṽ = 3278, 2963, 1677, 1595, 1502, 1329, 1237, 1180, 1111, 841, 752 cm^{-1}; HRMS (EI) Calcd for $C_{14}H_{18}N_3O_3^+$ $[M+H]^+$ 276.1343; found 276.1345.

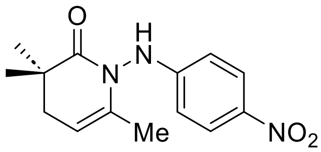

5dc

5cc (80%, EtOAc/nHex = 1:3, R_f = 0.3) as a yellow oil; ^{1}H NMR (400 MHz, $CDCl_3$): δ 8.13 (d, *J* = 9.2 Hz, 2H), 6.77 (s, 1H), 6.74 (d, *J* = 9.2 Hz, 2H), 5.04 (t, *J* = 5.5 Hz, 1H), 2.31-2-11 (m, 2H), 1.93-1.86 (m, 3H), 1.26 (s, 3H), 1.19 (s, 3H); ^{13}C NMR (100 MHz, $CDCl_3$): δ 175.2, 154.0, 141.6, 137.0, 125.9, 112.1, 102.2, 38.1, 34.4, 24.8, 24.4, 17.7; IR (ATR): ṽ = 2931, 1672, 1595, 1502, 1388, 1329, 1110, 840, 731 cm^{-1}; HRMS (EI) Calcd for $C_{14}H_{18}N_3O^+$ $[M+H]^+$ 276.1343; found 276.1344.

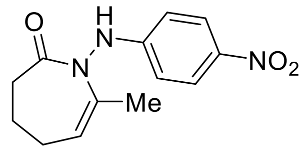

5ec

5ec (74%, EtOAc/nHex = 1:3, R_f = 0.3) as a yellow solid; mp: 169-172 °C; ^{1}H NMR (400 MHz, $CDCl_3$): δ 8.11 (d, *J* = 9.1, 2H), 6.93 (s, 1H), 6.76 (d, *J* = 9.1 Hz, 2H), 5.62 (td, *J* = 7.1, 1.1 Hz, 1H), 2.58 (t, *J* = 6.8 Hz, 2H), 2.37-2.14 (m, 4H), 1.93 (s, 3H); ^{13}C NMR (100 MHz, $CDCl_3$): δ 173.7, 153.7, 141.3, 138.9, 125.8, 118.0, 122.4, 33.9, 30.0, 24.1, 18.5; IR (ATR): ṽ 2948, 2868, 1665, 1595, 1502, 1323, 1275, 1254, 1177, 1109, 912, 837, 730 cm^{-1}; HRMS (EI) Calcd for $C_{13}H_{15}N_3O_3^+$ [M+H]$^+$ 261.1113; found 261.1115.

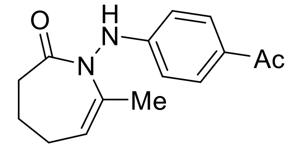

5ee

5ee (57%, EtOAc/nHex = 1:3, R_f = 0.2) as a yellow oil; ^{1}H NMR (400 MHz, $CDCl_3$): δ 7.85 (d, *J* = 8.7 Hz, 2H), 6.77 (d, *J* = 8.8 Hz, 2H), 6.75 (s, 1H), 5.59 (t, *J* = 7.2 Hz, 1H), 2.56 (t, *J* = 7.1 Hz, 2H), 2.51 (s, 3H), 2.34-2.13 (m, 4H), 1.94 (s, 3H); ^{13}C NMR (100 MHz, $CDCl_3$): δ 196.6, 173.7, 152.5, 139.3, 130.39, 130.36, 117.4, 112.7, 34.0, 30.1, 26.2, 24.1, 18.5; IR (ATR): ṽ 3280, 2943, 2858, 1666, 1600, 1432, 1358, 1319, 1273, 1176, 959, 831, 749 cm^{-1}; HRMS (EI) Calcd for $C_{15}H_{19}N_2O_2^+$ [M+H]$^+$ 259.1441; found 259.1443.

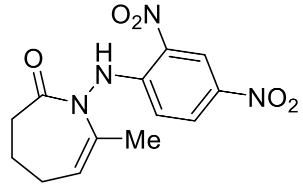

5eg

5eg (89%, EtOAc/nHex = 1:3, R_f = 0.2) as a yellow solid; mp: 54-57 °C; ^{1}H NMR (400 MHz, $CDCl_3$): δ 8.25 (d, *J* = 2.5 Hz, 1H), 8.06 (dd, *J* = 9.0, 2.4 Hz, 1H), 7.01 (s, 1H), 6.74 (d, *J* = 9.1 Hz, 1H), 5.65 (td, *J* = 7.2, 1.2 Hz, 1H), 2.59 (t, *J* = 7.1 Hz, 2H), 2.36-2.16 (m, 4H), 1.94 (s, 3H); ^{13}C NMR (100 MHz, $CDCl_3$): δ 173.3, 149.3, 140.7, 138.6, 125.7, 124.1, 118.42, 118.39, 111.7, 33.9, 29.9, 24.1, 18.4; IR (ATR): ṽ 3265, 2943, 2865, 1677, 1588, 1500, 1329, 1293,

1110, 894, 822, 744 cm^{-1}; HRMS (EI) Calcd for $C_{13}H_{15}N_4O_5^+$ $[M+H]^+$ 307.1037; found 307.1037.

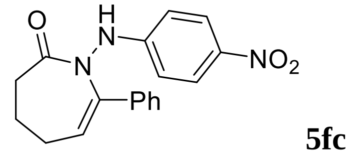

5fc

5fc (42%, EtOAc/nHex = 1:3, R_f = 0.3) as a yellow solid; mp: 171-174 °C; ^{1}H NMR (400 MHz, $CDCl_3$): δ 8.04 (d, *J* = 9.1, 2H), 7.40-7.26 (m, 5H), 6.85 (s, 1H), 6.72 (d, *J* = 9.1 Hz, 2H), 6.09 (t, *J* = 7.7 Hz, 1H), 2.69 (t, *J* = 7.1 Hz, 2H), 2.56 (q, *J* = 7.4 Hz, 2H), 2.35 (quintet, *J* = 7.0 Hz, 2H); ^{13}C NMR (100 MHz, $CDCl_3$): δ 174.1, 153.3, 143.8, 141.3, 135.0, 129.0, 128.6, 126.8, 125.7, 121.3, 112.9, 33.6, 30.6, 24.6; IR (ATR): ṽ 2949, 2863, 1669, 1596, 1501, 1446, 1323, 1265, 1152, 1110, 973, 838, 753, 697 cm^{-1}; HRMS (EI) Calcd for $C_{18}H_{17}N_3O_3^+$ $[M+H]^+$ 323.1270; found 323.1270.

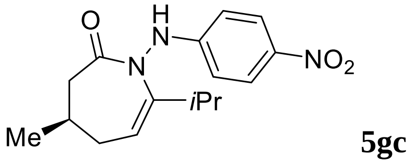

5gc

5gc (83%, EtOAc/nHex = 1:3, R_f = 0.3) as a yellow solid; mp: 67-70 °C; $[\alpha]^{20}_D$ = 72.5° (c 1.00, $CHCl_3$); ^{1}H NMR (400 MHz, $CDCl_3$): δ 8.10 (d, *J* = 9.1, 2H), 7.00 (s, 1H), 6.75 (d, *J* = 9.1 Hz, 2H), 5.69 (t, *J* = 7.6 Hz, 1H), 2.74-2.52 (m, 3H), 2.52-2.32 (m, 1H), 2.26-2.13 (m, 1H), 2.08-1.94 (m, 1H), 1.11 (d, *J* = 6.8 Hz, 3H), 1.08 (dd, *J* = 15.4, 6.4 Hz, 6H); ^{13}C NMR (100 MHz, $CDCl_3$): δ 173.0, 154.2, 149.2, 141.4, 125.8, 114.5 (br), 112.7, 41.1, 38.3, 31.9 (br), 28.9, 21.5, 21.1 (br); IR (ATR): ṽ 2965, 1659, 1595, 1491, 1361, 1292, 1093, 1010, 822, 763 cm^{-1}; HRMS (EI) Calcd for $C_{16}H_{22}N_3O_3^+$ $[M+H]^+$ 304.1656; found 304.1656.

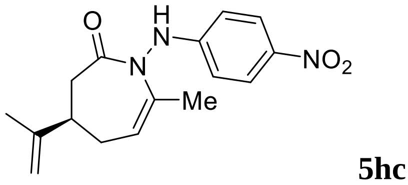

5hc

5hc (77%, EtOAc/nHex = 1:3, R_f = 0.3) as a yellow solid; mp: 67-70 °C; $[\alpha]^{20}_D$ = -659.1° (c 1.00, $CHCl_3$); ^{1}H NMR (400 MHz, $CDCl_3$): δ 8.11 (d, *J* = 9.1, 2H), 6.91 (s, 1H), 6.76 (d, *J* = 9.1 Hz, 2H), 5.63 (t, *J* = 7.6 Hz, 1H), 4.86 (s, 1H), 4.78 (s, 1H), 3.10 (quintet, *J* = 7.0 Hz, 1H), 2.74-2.56 (m, 2H), 2.54-2.35 (m, 1H), 2.32-2.21 (m, 1H), 1.95 (s, 3H), 1.76 (s, 3H); ^{13}C NMR (100 MHz, $CDCl_3$): δ 172.7, 153.6, 147.0, 141.3, 139.0, 125.9, 117.1, 112.4, 110.6, 50.0, 38.9, 29.5, 20.8, 18.4; IR (ATR): ṽ 2964, 2873, 1743, 1610, 1526, 1467, 1345, 1316, 1146, 1107, 1008, , 859, 808, 752 cm^{-1}; HRMS (EI) Calcd for $C_{16}H_{20}N_3O_3^+$ [M+H]$^+$ 302.1499; found 302.1500.

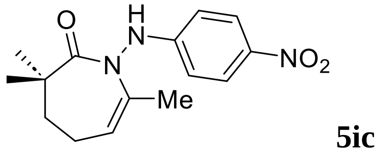

5ic

5ic (50%, EtOAc/nHex = 1:3, R_f = 0.3) as a yellow solid; mp: 66-69 °C; ^{1}H NMR (400 MHz, $CDCl_3$): δ 8.13 (d, *J* = 9.1, 2H), 6.80 (d, *J* = 9.1 Hz, 2H), 6.73 (s, 1H), 5.53 (t, *J* = 7.0 Hz, 1H), 2.40-2.15 (m, 2H), 2.08-1.95 (m, 2H), 1.95 (s, 3H), 1.28 (s, 6H); ^{13}C NMR (100 MHz, $CDCl_3$): δ 178.7, 154.4, 141.5, 138.5, 125.9, 117.2, 112.5, 112.4, 44.5, 42.7, 27.4 (br), 24.5, 19.4; IR (ATR): ṽ 2928, 1713, 1527, 1346, 1275, 1012, 867, 750 cm^{-1}; HRMS (EI) Calcd for $C_{15}H_{20}N_3O_3^+$ [M+H]$^+$ 290.1499; found 290.1499.

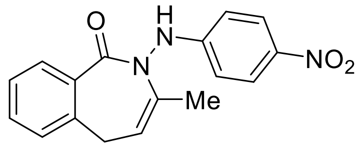

5jc

5jc (63%, EtOAc/nHex = 1:3, R_f = 0.3) as a yellow solid; mp: 66-69 °C; ^{1}H NMR (400 MHz, $CDCl_3$): δ 8.12 (d, *J* = 9.1, 2H), 7.94 (d, *J* = 7.7 Hz, 1H), 7.68 (s, 1H), 7.47 (t, *J* = 7.5 Hz, 1H), 7.34 (t, *J* = 7.5 Hz, 1H), 7.18 (d, *J* = 7.5 Hz, 1H), 6.91 (d, *J* = 9.1 Hz, 2H), 5.67 (t, *J* = 7.4 Hz, 1H), 3.71-3.52 (m, 1H), 3.31-3.11 (m, 1H), 1.93 (s, 3H); ^{13}C NMR (100 MHz, $CDCl_3$): δ 168.2, 153.6, 143.8, 141.8, 137.4, 132.8, 131.7, 130.8, 126.8, 126.6, 125.9, 116.9, 113.0, 31.9, 18.9; IR (ATR): ṽ = 2957, 2922, 1641, 1597, 1503, 1327, 1273, 1110, 839, 751 cm^{-1}; HRMS (EI) Calcd for $C_{17}H_{16}N_3O_3^+$ [M+H]$^+$ 310.1186; found 310.1188.

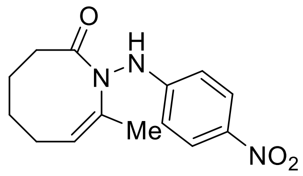

5kc

5kc (78%, EtOAc/nHex = 1:3, R_f = 0.3) as a yellow solid; mp: 133-136 °C; ^{1}H NMR (400 MHz, $CDCl_3$): δ 8.12 (d, *J* = 9.1, 2H), 6.80 (d, *J* = 9.1 Hz, 1H), 6.56 (s, 1H), 5.45 (t, *J* = 8.2 Hz, 1H), 2.63-2.43 (m, 2H), 2.40-2.26 (m, 1H), 2.06-1.83 (m, 3H), 1.93 (s, 3H), 1.74-1.58 (m, 1H), 1.33-1.19 (m, 1H); ^{13}C NMR (100 MHz, $CDCl_3$): δ 173.9, 153.6, 141.4, 135.9, 125.8, 123.7, 112.7, 33.6, 26.0, 24.6, 24.5, 19.3; IR (ATR): ṽ = 3239, 2927, 2859, 1655, 1595, 1503, 1488, 1325, 1276, 1178, 1110, 838, 752 cm^{-1}; HRMS (EI) Calcd for $C_{14}H_{18}N_3O_3^+$ [M+H]$^+$ 276.1343; found 276.1345.

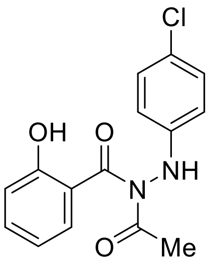

6yb

7zb (61%, EtOAc/nHex = 1:3, R_f = 0.3) as an off-white solid; mp: 139-141 °C; ^{1}H NMR (400 MHz, $CDCl_3$): δ 8.12 (s, 1H), 7.72 (dd, *J* = 7.6, 1.6 Hz, 1H), 7.52 (td, *J* = 8.0, 1.6 Hz, 1H), 7.30 (td, *J* = 7.6, 1.2 Hz, 1H), 7.18-7.13 (m, 1H), 7.16 (d, *J* = 8.4 Hz, 2H), 6.76 (d, *J* = 8.8 Hz, 2H), 2.29 (s, 3H); ^{13}C NMR (100 MHz, $CDCl_3$): δ 169.2, 166.2, 148.3, 146.3, 132.6, 129.6, 129.1, 126.4, 126.0, 125.9, 123.4, 114.9, 21.1; IR (ATR): ṽ = 3296, 1763, 1666, 1606, 1490, 1267, 1200, 827, 738, 705 cm^{-1}; HRMS (EI) Calcd for $C_{15}H_{14}ClN_2O_3^+$ [M+H]$^+$ 305.0687; found 305.0690.

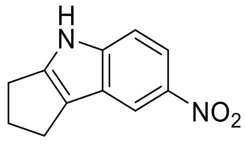

7lc

7lc (43%, EtOAc/nHex = 1:3, R_f = 0.3) as a yellow solid; mp: 146-149 °C; ^{1}H NMR (400 MHz, $CDCl_3$): δ 8.39 (d, *J* = 1.8 Hz, 1H), 8.23 (s, 1H), 8.01 (dd, *J* = 8.9, 2.2 Hz, 1H), 7.30 (d, *J* = 9.0 Hz, 1H), 3.01-2.82 (m, 4H), 2.66-2.52 (m, 2H); ^{13}C NMR (100 MHz, $CDCl_3$): δ 147.1, 143.9, 124.0, 122.1, 116.3, 115.6, 110.9, 28.5, 26.0, 24.3; IR (ATR): ṽ = 3313, 2931, 2855, 1507, 1471, 1308, 1227, 1111, 884, 808, 734 cm^{-1}; HRMS (EI) Calcd for $C_{11}H_{11}N_2O_2^+$ [M+H]$^+$ 203.0815; found 203.0815.

6. Synthetic Applications

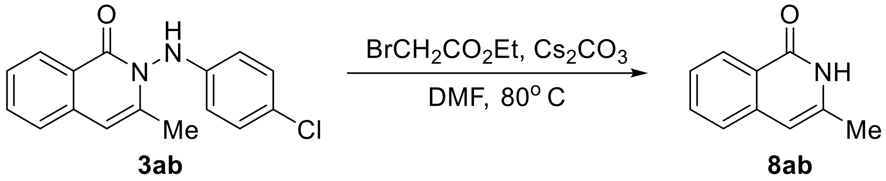

To a suspension of cesium carbonate (0.40 mmol) in DMF (2 mL) was added **3ab** (0.20 mmol) at room temperature. The mixture was stirred for 10 min and then ethyl bromoacetate (0.22 mmol) was added. The mixture was then heated to 80 °C until the completion of the reaction by TLC. Upon completion of the reaction, the mixture was cooled to RT. Water (6 mL) was added and the product was extracted with ethyl acetate (5 mL x 3). The combined organic layers were washed with brine (15 mL), dried (Na_2CO_3), and concentrated. The residue was purified by flash chromatography (EtOAc/Hex) to afford 93% of **8ab**.

8ab[8] (93%, EtOAc/nHex = 1:1, R_f = 0.4) as an off-white solid (^{1}H and ^{13}C NMR spectra matched with the literature data)

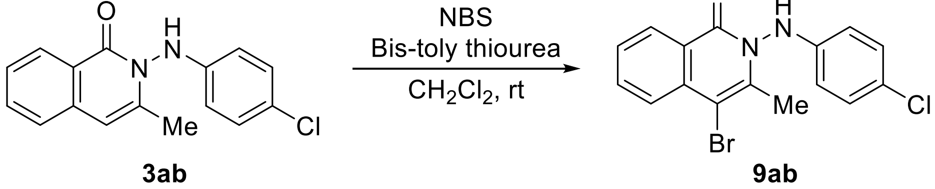

To a solution of **3ab** (0.20 mmol) in CH_2Cl_2 (2 mL) was added 1,3-di(*p*-tolyl)thiourea (0.040 mmol) and N-bromosuccinimide (0.30 mmol) at room temperature. When the TLC indicated complete reaction, the mixture was concentrated in vacuo. The residue was purified by flash

chromatography (EtOAc/Hex) to afford 74% of **9ab**.

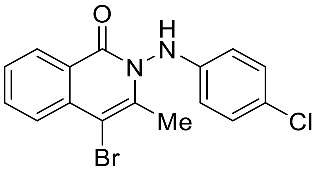

9ab

9ab (74%, EtOAc/nHex = 1:3, R_f = 0.5) as an off-white solid; mp: 204-206 °C; ^{1}H NMR (400 MHz, $CDCl_3$): δ 8.36 (d, *J* = 7.9 Hz, 1H), 7.99 (d, *J* = 8.2 Hz, 1H), 7.78 (t, *J* = 7.3 Hz, 1H), 7.51 (t, *J* = 7.6 Hz, 1H), 7.39 (s, 1H), 7.19 (d, *J* = 8.7 Hz, 2H), 6.64 (d, *J* = 8.7 Hz, 2H), 2.69 (s, 3H); ^{13}C NMR (100 MHz, $CDCl_3$): δ 161.2, 145.4, 140.9, 135.6, 133.7, 129.4, 128.1, 127.3, 127.1, 126.2, 124.3, 115.2, 100.8, 19.3; IR (ATR): ṽ = 3236, 1656, 1609, 1586, 1493, 1264, 738, 701 cm^{-1}; HRMS (EI) Calcd for $C_{16}H_{13}BrClN_2O^+$ $[M+H]^+$ 362.9894; found 362.9898.

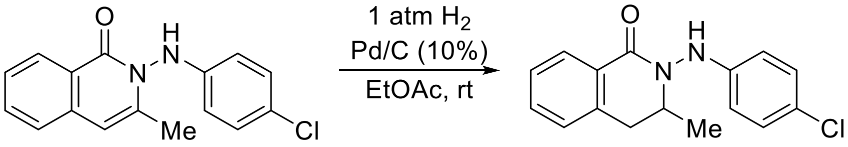

3ab **10ab**

To a solution of **3ab** (0.20 mmol) in ethyl acetate (2 mL) was added Pd/C (10%) at room temperature. The mixture was purged with hydrogen gas and the mixture was stirred at 25 °C under 1 atm hydrogen gas. When the TLC indicated complete reaction, the mixture was filtered through celite. The filtrate was concentrated in vacuo and the residue was purified by flash chromatography (EtOAc/Hex) to afford 80% of **10ab**

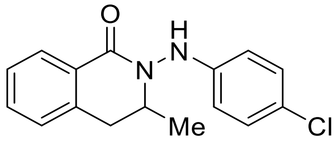

10ab

10ab (80%, EtOAc/nHex = 1:3, R_f = 0.4) as an off-white solid; mp: 161-164 °C; ^{1}H NMR (400 MHz, $CDCl_3$): δ 8.09 (d, *J* = 7.7 Hz, 1H), 7.49 (t, *J* = 7.4 Hz, 1H), 7.37 (t, *J* = 7.6 Hz, 1H), 7.30-7.23 (m, 1H), 7.20 (d, *J* = 8.7 Hz, 2H), 6.86 (d, *J* = 8.7 Hz, 2H), 6.82 (s, 1H), 4.11-3.98 (m, 1H), 3.57 (dd of ABq, *J* = 16.0, 5.8 Hz, 1H), 2.93 (dd of ABq, *J* = 16.0, 3.8 Hz, 1H), 1.30

(d, J = 6.6 Hz, 3H); ^{13}C NMR (100 MHz, $CDCl_3$): δ 164.6, 146.2, 136.4, 132.6, 129.2, 128.3, 128.1, 127.8, 127.1, 126.2, 115.3, 55.5, 34.7, 17.6; IR (ATR): ṽ = 3265, 2971, 1652, 1604, 1488, 1409, 1337, 1276, 1241, 1094, 823, 755, 714 cm^{-1}; HRMS (EI) Calcd for $C_{16}H_{16}ClN_2O^+$ $[M+H]^+$ 287.0946; found 287.0948.

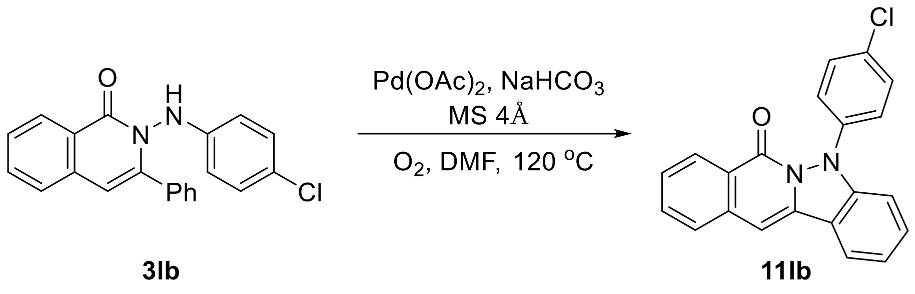

To a solution of **3ab** (0.20 mmol) in DMF (5 mL) was added $Pd(OAc)_2$ (0.010 mmol), $NaHCO_3$ (0.2 mmol) and MS 4Å (60 mg) under an oxygen atmosphere. The mixture was then tightly capped and stirred for 10 min at room temperature and then heated at 120 °C for 48 h. The mixture was then cooled to room temperature, diluted with CH_2Cl_2 (10 mL), and filtered through celite. The filtrate was concentrated in vacuo and the residue was purified by flash chromatography (EtOAc/Hex) to afford 77% of **11lb**.

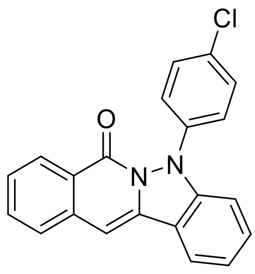

11lb

11lb (77%, EtOAc/nHex = 1:3, R_f = 0.6) as an off-white solid; mp: 218-220 °C; ^{1}H NMR (400 MHz, $CDCl_3$): δ 8.43 (d, J = 8.2 Hz, 1H), 7.91 (d, J = 7.8 Hz, 1H), 7.74 (d, J = 7.8 Hz, 1H), 7.69 (t, J = 6.8 Hz, 1H), 7.47 (t, J = 6.7 Hz, 2H), 7.40 (d, J = 8.8 Hz, 2H), 7.33 (t, J = 7.8 Hz, 1H), 7.28 (d, J = 8.8 Hz, 2H), 7.17 (s, 1H), 7.11 (d, J = 8.2 Hz, 1H); ^{13}C NMR (100 MHz, $CDCl_3$): δ 156.5, 147.3, 142.2, 136.6, 136.5, 133.5, 131.8, 130.9, 129.5, 127.5, 126.1, 125.55, 125.49, 124.2, 123.7, 121.2, 121.0, 112.3, 94.9; IR (ATR): ṽ = 1666, 1626, 1486, 1277, 1260, 754, 701 cm^{-1}; HRMS (EI) Calcd for $C_{21}H_{14}ClN_2O^+$ $[M+H]^+$ 345.0789; found 345.0792.

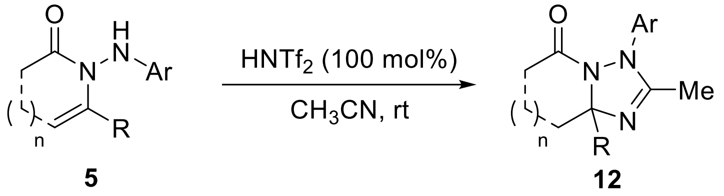

To a solution of $HNTf_2$ (0.1 mmol) in CH_3CM (1 mL) was added corresponding **5** (0.1 mmol) at room temperature. The reaction mixture was stirred at 25 °C and monitored by TLC. Upon completion of the reaction, the mixture was quenched with Et_3N (20 µl) and concentrated in vacuo. The residue was purified by flash chromatography (EtOAc/Hex) to afford 83-86% of **12**.

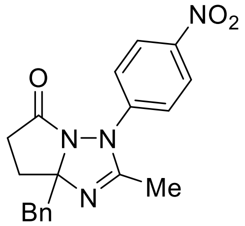

12ac

12ac (86%, EtOAc/nHex = 1:1, R_f = 0.1) as a yellow oil; ^{1}H NMR (400 MHz, $CDCl_3$): δ 8.03 (d, *J* = 9.2 Hz, 2H), 7.11-6.99 (m, 5H), 6.89 (d, *J* = 9.2 Hz, 2H), 2.99 (d of ABq, *J* = 13.8 Hz, 1H), 2.91 (d of ABq, *J* = 13.8 Hz, 1H), 2.69-2.45 (m, 3H), 2.38-2.29 (m, 1H), 2.26 (s, 3H); ^{13}C NMR (100 MHz, $CDCl_3$): δ 184.2, 155.5, 146.5, 143.2, 134.3, 130.3, 127.9, 127.3, 124.7, 118.6, 92.5, 45.6, 34.2, 28.6, 16.9; IR (ATR): ṽ = 3039, 2923, 1737, 1591, 1496, 1306, 1253, 1113, 856, 749, 703 cm^{-1}; HRMS (EI) Calcd for $C_{19}H1_8N_4NaO_3^+$ $[M+Na]^+$ 373.1271; found 373.1273.

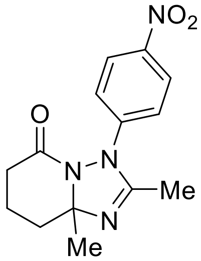

12bc

12bc (84%, EtOAc/nHex = 1:1, R_f = 0.1) as a yellow solid; mp: 163-166 °C; ^{1}H NMR (400

MHz, $CDCl_3$): δ 8.22 (d, *J* = 9.2 Hz, 2H), 7.30 (d, *J* = 9.1 Hz, 2H), 2.74-2.62 (m, 1H), 2.56-2.43 (m, 1H), 2.39-232 (m, 1H), 2.32 (s, 3H), 2.06-2.19 (m, 2H), 1.88-1.75 (m, 1H), 1.48 (s, 3H); ^{13}C NMR (100 MHz, $CDCl_3$): δ 170.0, 155.7, 147.3, 143.9, 125.3, 119.3, 88.0, 34.0, 30.9, 27.0, 16.6, 15.9; IR (ATR): ṽ = 3051, 2979, 1671, 1631, 1589, 1496, 1317, 1264, 1120, 952, 852, 730 cm^{-1}; HRMS (EI) Calcd for $C_{14}H_{16}N_4NaO_3^+$ $[M+Na]^+$ 311.1115; found 311.1117.

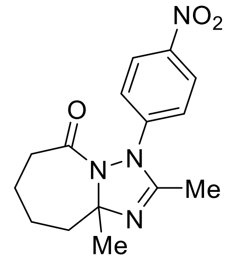

12ec

12ec (83%, EtOAc/nHex = 1:1, R_f = 0.1) as a yellow solid; mp: 163-166 °C; ^1H NMR (400 MHz, $CDCl_3$): δ 8.25 (d, *J* = 9.0 Hz, 2H), 7.35 (d, *J* = 9.0 Hz, 2H), 2.71-2.58 (m, 1H), 2.56-2.45 (m, 1H), 2.38-2.24 (m, 1H), 2.06 (s, 3H), 2.01-1.85 (m, 4H), 1.76 (s, 3H), 1.76-1.64 (m, 1H); ^{13}C NMR (100 MHz, $CDCl_3$): δ 170.8, 156.0, 147.2, 145.4, 125.1, 122.6, 88.9, 39.3, 36.0, 26.2, 23.8, 23.6, 15.0; IR (ATR): ṽ = 3107, 2931, 2871, 1649, 1589, 1516, 1338, 1262, 1191, 1112, 855, 733 cm^{-1}; HRMS (EI) Calcd for $C_{15}H_{18}N_4NaO_3^+$ $[M+Na]^+$ 325.1271; found 325.1273.

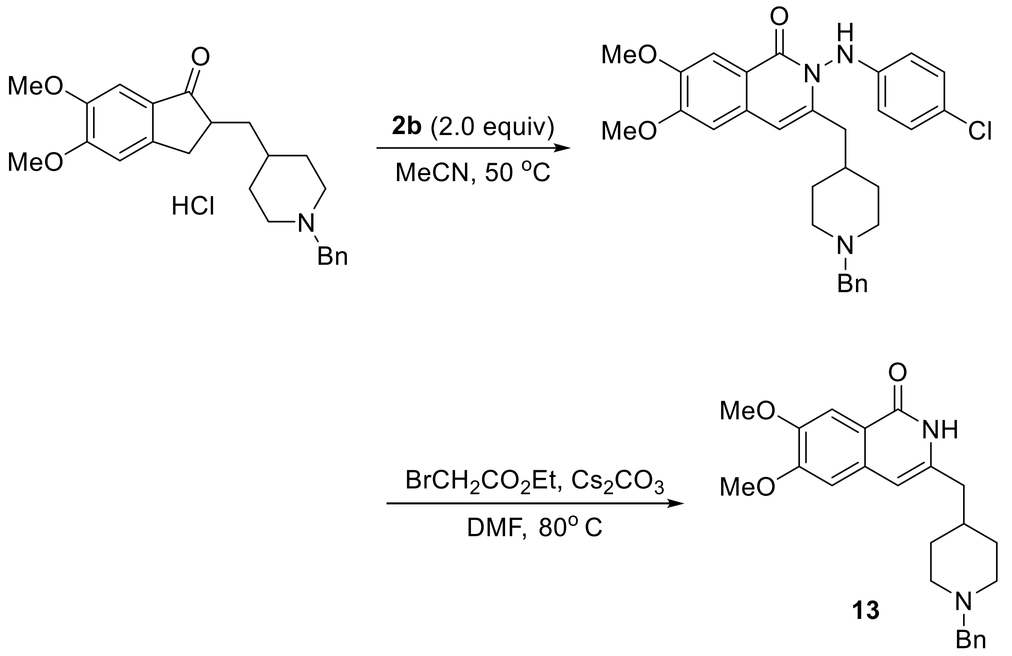

To a suspension of diazonium salt **2b** (0.4 mmol) in CH_3CN (0.5 mL) was added donepezil hydrochloride (0.2 mmol) at room temperature. The mixture was then heated to 50 °C until the completion of the reaction by TLC. Upon completion of the reaction, the mixture was cooled to RT and concentrated in vacuo. The residue was purified by flash chromatography (EtOAc/Hex) 97% of corresponding isoquinolone.

To a suspension of cesium carbonate (0.38 mmol) in DMF (2 mL) was added corresponding isoquinolone (0.19 mmol) at room temperature. The mixture was stirred for 10 min and then ethyl bromoacetate (0.21 mmol) was added. The mixture was then heated to 80 °C until the completion of the reaction by TLC. Upon completion of the reaction, the mixture was cooled to RT. Water (6 mL) was added and the product was extracted with ethyl acetate (5 mL x 3). The combined organic layers were washed with brine (15 mL), dried (Na_2CO_3), and concentrated. The residue was purified by flash chromatography (EtOAc/Hex) to afford 80% of **13**

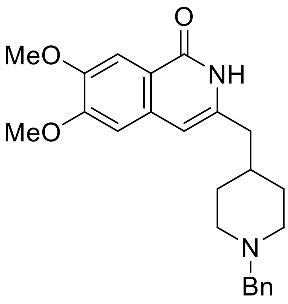

13

13 (76%, MeOH/EtOAc = 1:20, R_f = 0.2) as a off-white solid; mp: 236-239 °C; ^{1}H NMR (400 MHz, $CDCl_3$): δ 10.1 (s, 1H), 7.75 (s, 1H), 7.39-7.17 (m, 5H), 6.83 (s, 1H), 6.20 (s, 1H), 3.99 (s, 6H), 3.45 (s, 2H), 2.85 (d, *J* = 11.1 Hz, 2H), 2.49 (d, *J* = 6.7 Hz, 2H), 1.91 (t, *J* = 11.4 Hz, 2H), 1.70 (d, *J* = 12.3 Hz, 3H), 1.36 (q, *J* = 11.1 Hz, 2H); ^{13}C NMR (100 MHz, $CDCl_3$): δ 163.4, 153.8, 148.7, 138.5, 138.3, 134.1, 129.2, 128.2, 127.0, 118.2, 107.1, 105.8, 104.6, 63.4, 56.2, 56.1, 53.6, 40.7, 35.5, 32.1; IR (ATR): ṽ = 3151, 2933, 2775, 1633, 1507, 1265, 1226, 1086, 882, 743, 699 cm^{-1}; HRMS (EI) Calcd for $C_{24}H_{29}N_2O_3^+$ $[M+H]^+$ 393.2173; found 393.2174.

7. Mechanistic Experiments

In order to probe the ring expansion step, a series of α-diazenyl derivatives were prepared and Hammett plots was constructed.

First, we prepared α-diazenyl intermediates with different substituents at C5-position of indanone and compared their initial rates (k_X) relative to the rate of unsubstituted substrate (k_H). A linear relationship was found between the logarithm of relative rates versus σ^+ constant, which indicated a positive charge developing at the carbonyl carbon in the transition state.

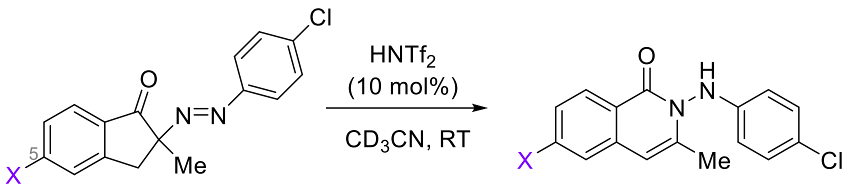

X	σ_p	σ^+	σ^-	k_X (s^{-1})	log(k_X/k_H)
p-OMe	-0.27	**-0.78**	-0.26	8.1×10^{-2}	1.10
p-Me	-0.17	**-0.31**	-0.17	2.7×10^{-2}	0.625
p-F	0.062	**-0.07**	-0.03	9.3×10^{-3}	0.162
p-H	0	**0**	0	6.4×10^{-3}	0
p-Cl	0.23	**0.11**	0.19	5.8×10^{-3}	-0.043

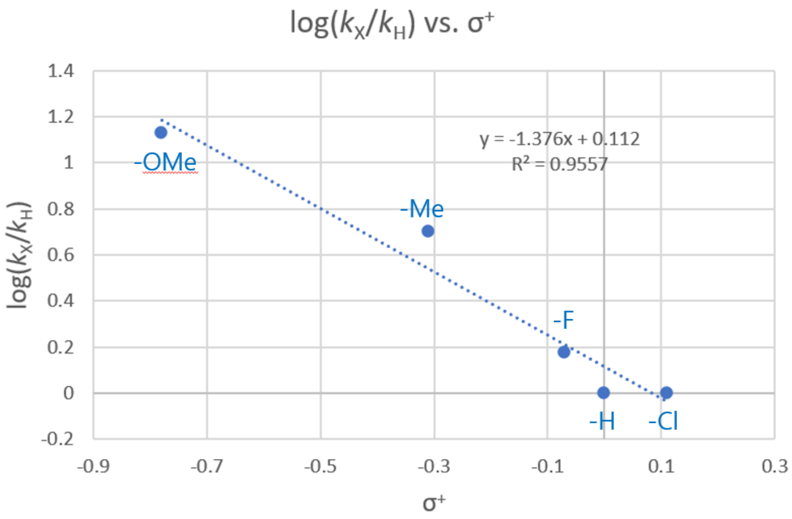

Secondly, we prepared a series of α-diazenyl derivatives employing different arene diazonium

salts, having substituents at para-position. A linear relationship was found between the logarithm of relative rates versus σ^+ constant,

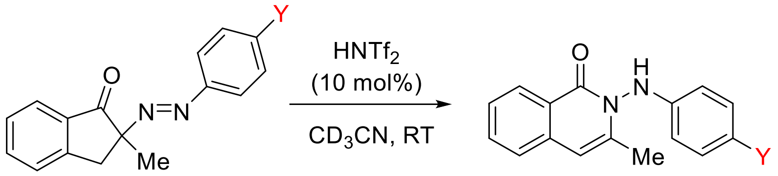

X	σ_p	σ^+	σ^-	k_X (s^{-1})	$\log(k_X/k_H)$
p-Me	-0.17	-0.31	-0.17	1.1 x 10^{-3}	0.4357286
p-F	0.15	-0.07	-0.03	3.7 x 10^{-3}	0.0957503
p-H	0	0	0	3.0 x 10^{-3}	0
p-Cl	0.24	0.11	0.19	6.3 x 10^{-3}	0.3194531

From these Hammett plots, a plausible transition state is proposed as below in which positive partial charge is developing at C1 of indanone and at the same time, negative partial charge is developing at N2 position.

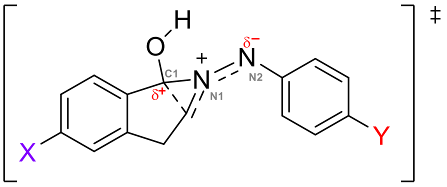

8. X-Ray Crystallographic Data of 3aa

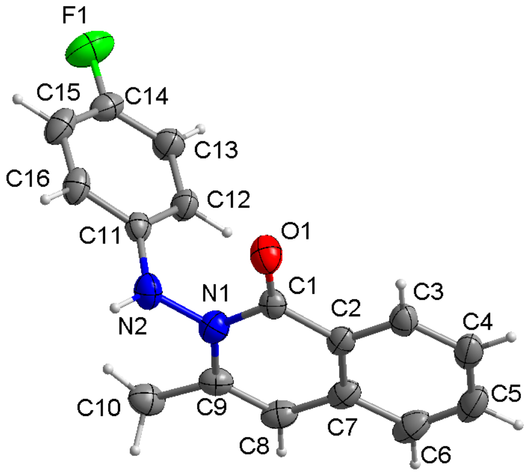

CCDC 2428433; This can be obtained free of charge from The Cambridge Crystallographic Data Center via www.ccdc.cam.ac.uk/data_request/cif.

Table S7. Crystal data and structure refinement for **3aa**.

Identification code	**3aa**	
Empirical formula	C16 H13 F N2 O	
Formula weight	268.28	
Temperature	193(2) K	
Wavelength	0.71073 Å	
Crystal system	Monoclinic	
Space group	$P2_1/c$	
Unit cell dimensions	a = 8.6254(2) Å	α= 90°.
	b = 17.3035(4) Å	β= 95.613(2)°.
	c = 8.9185(2) Å	γ = 90°.
Volume	1324.70(5) $Å^3$	
Z	4	
Density (calculated)	1.345 Mg/m^3	
Absorption coefficient	0.095 mm^{-1}	
F(000)	560	
Crystal size	0.524 x 0.478 x 0.374 mm^3	
Theta range for data collection	2.354 to 28.299°.	
Index ranges	-11<=h<=11, -23<=k<=19, -11<=l<=11	
Reflections collected	13416	
Independent reflections	3283 [R(int) = 0.0317]	
Completeness to theta = 25.242°	99.9 %	
Absorption correction	Semi-empirical from equivalents	
Max. and min. transmission	0.7457 and 0.6969	
Refinement method	Full-matrix least-squares on F^2	
Data / restraints / parameters	3283 / 0 / 181	
Goodness-of-fit on F^2	1.058	
Final R indices [I>2sigma(I)]	R1 = 0.0618, wR2 = 0.1818	
R indices (all data)	R1 = 0.0793, wR2 = 0.2000	
Extinction coefficient	n/a	
Largest diff. peak and hole	0.500 and -0.516 e.$Å^{-3}$	

Table S8. Atomic coordinates (x 10^4) and equivalent isotropic displacement parameters ($Å^2$x 10^3) for **3aa**. U(eq) is defined as one third of the trace of the orthogonalized U^{ij} tensor.

	x	y	z	U(eq)
F(1)	6371(2)	2669(1)	-446(2)	69(1)
O(1)	4210(2)	4196(1)	5896(2)	45(1)
N(1)	2291(2)	4389(1)	4001(2)	36(1)
N(2)	3390(2)	4691(1)	3102(2)	39(1)
C(1)	2823(2)	4139(1)	5437(2)	36(1)
C(2)	1638(2)	3800(1)	6302(2)	38(1)
C(3)	2088(3)	3521(1)	7762(2)	45(1)
C(4)	1010(3)	3165(1)	8559(3)	56(1)
C(5)	-520(3)	3086(1)	7936(3)	63(1)
C(6)	-991(3)	3372(2)	6527(3)	58(1)
C(7)	97(2)	3730(1)	5672(2)	44(1)
C(8)	-322(2)	4019(1)	4180(3)	46(1)
C(9)	749(2)	4342(1)	3370(2)	40(1)
C(10)	393(3)	4651(2)	1811(2)	53(1)
C(11)	4190(2)	4141(1)	2299(2)	36(1)
C(12)	3777(3)	3368(1)	2193(2)	46(1)
C(13)	4533(3)	2868(1)	1285(3)	50(1)
C(14)	5672(2)	3153(1)	492(2)	48(1)
C(15)	6127(3)	3913(2)	580(3)	54(1)
C(16)	5394(3)	4407(1)	1498(2)	47(1)

Table S9. Bond lengths [Å] and angles [°] for **3aa**.

F(1)-C(14)	1.365(2)
O(1)-C(1)	1.230(2)
N(1)-C(1)	1.387(2)
N(1)-C(9)	1.395(2)
N(1)-N(2)	1.402(2)
N(2)-C(11)	1.412(2)
N(2)-H(2A)	0.8800
C(1)-C(2)	1.462(3)
C(2)-C(7)	1.397(3)
C(2)-C(3)	1.407(3)
C(3)-C(4)	1.371(3)
C(3)-H(3A)	0.9500
C(4)-C(5)	1.387(4)
C(4)-H(4A)	0.9500
C(5)-C(6)	1.374(4)
C(5)-H(5A)	0.9500
C(6)-C(7)	1.410(3)
C(6)-H(6A)	0.9500
C(7)-C(8)	1.434(3)
C(8)-C(9)	1.350(3)
C(8)-H(8A)	0.9500
C(9)-C(10)	1.492(3)
C(10)-H(10A)	0.9800
C(10)-H(10B)	0.9800
C(10)-H(10C)	0.9800
C(11)-C(12)	1.384(3)
C(11)-C(16)	1.395(3)
C(12)-C(13)	1.390(3)
C(12)-H(12A)	0.9500
C(13)-C(14)	1.358(3)
C(13)-H(13A)	0.9500
C(14)-C(15)	1.372(4)
C(15)-C(16)	1.380(3)
C(15)-H(15A)	0.9500
C(16)-H(16A)	0.9500

C(1)-N(1)-C(9)	124.84(16)
C(1)-N(1)-N(2)	117.59(15)
C(9)-N(1)-N(2)	117.55(15)
N(1)-N(2)-C(11)	115.45(14)
N(1)-N(2)-H(2A)	122.3
C(11)-N(2)-H(2A)	122.3
O(1)-C(1)-N(1)	120.38(17)
O(1)-C(1)-C(2)	124.30(18)
N(1)-C(1)-C(2)	115.30(17)
C(7)-C(2)-C(3)	120.54(18)
C(7)-C(2)-C(1)	120.57(18)
C(3)-C(2)-C(1)	118.85(18)
C(4)-C(3)-C(2)	119.6(2)
C(4)-C(3)-H(3A)	120.2
C(2)-C(3)-H(3A)	120.2
C(3)-C(4)-C(5)	120.2(2)
C(3)-C(4)-H(4A)	119.9
C(5)-C(4)-H(4A)	119.9
C(6)-C(5)-C(4)	121.0(2)
C(6)-C(5)-H(5A)	119.5
C(4)-C(5)-H(5A)	119.5
C(5)-C(6)-C(7)	120.0(2)
C(5)-C(6)-H(6A)	120.0
C(7)-C(6)-H(6A)	120.0
C(2)-C(7)-C(6)	118.5(2)
C(2)-C(7)-C(8)	119.15(18)
C(6)-C(7)-C(8)	122.3(2)
C(9)-C(8)-C(7)	121.19(19)
C(9)-C(8)-H(8A)	119.4
C(7)-C(8)-H(8A)	119.4
C(8)-C(9)-N(1)	118.92(19)
C(8)-C(9)-C(10)	123.9(2)
N(1)-C(9)-C(10)	117.17(18)
C(9)-C(10)-H(10A)	109.5
C(9)-C(10)-H(10B)	109.5
H(10A)-C(10)-H(10B)	109.5

C(9)-C(10)-H(10C)	109.5
H(10A)-C(10)-H(10C)	109.5
H(10B)-C(10)-H(10C)	109.5
C(12)-C(11)-C(16)	118.95(19)
C(12)-C(11)-N(2)	123.43(17)
C(16)-C(11)-N(2)	117.50(17)
C(11)-C(12)-C(13)	120.58(19)
C(11)-C(12)-H(12A)	119.7
C(13)-C(12)-H(12A)	119.7
C(14)-C(13)-C(12)	118.8(2)
C(14)-C(13)-H(13A)	120.6
C(12)-C(13)-H(13A)	120.6
C(13)-C(14)-F(1)	118.8(2)
C(13)-C(14)-C(15)	122.4(2)
F(1)-C(14)-C(15)	118.85(19)
C(14)-C(15)-C(16)	118.9(2)
C(14)-C(15)-H(15A)	120.6
C(16)-C(15)-H(15A)	120.6
C(15)-C(16)-C(11)	120.4(2)
C(15)-C(16)-H(16A)	119.8
C(11)-C(16)-H(16A)	119.8

Symmetry transformations used to generate equivalent atoms:

Table S10 Anisotropic displacement parameters ($Å^2$x 10^3) for **3aa**. The anisotropic displacement factor exponent takes the form: $-2\pi^2$[h^2 $a^{*2}U^{11}$ + ... + 2 h k a* b* U^{12}]

	U^{11}	U^{22}	U^{33}	U^{23}	U^{13}	U^{12}
F(1)	56(1)	86(1)	69(1)	-17(1)	22(1)	7(1)
O(1)	42(1)	46(1)	46(1)	6(1)	-3(1)	-12(1)
N(1)	36(1)	36(1)	36(1)	-2(1)	4(1)	-3(1)
N(2)	45(1)	32(1)	40(1)	5(1)	7(1)	-7(1)
C(1)	39(1)	30(1)	38(1)	-2(1)	3(1)	-5(1)
C(2)	44(1)	29(1)	42(1)	-6(1)	11(1)	-3(1)
C(3)	57(1)	37(1)	44(1)	1(1)	13(1)	-1(1)
C(4)	76(2)	42(1)	55(1)	3(1)	28(1)	2(1)
C(5)	71(2)	48(1)	76(2)	-1(1)	42(1)	-8(1)
C(6)	48(1)	53(1)	77(2)	-14(1)	23(1)	-11(1)
C(7)	43(1)	37(1)	54(1)	-11(1)	14(1)	-5(1)
C(8)	35(1)	48(1)	56(1)	-14(1)	2(1)	-3(1)
C(9)	38(1)	39(1)	43(1)	-10(1)	-1(1)	1(1)
C(10)	52(1)	60(1)	44(1)	-5(1)	-5(1)	8(1)
C(11)	35(1)	40(1)	34(1)	5(1)	0(1)	-3(1)
C(12)	47(1)	42(1)	50(1)	4(1)	16(1)	-5(1)
C(13)	54(1)	44(1)	55(1)	-2(1)	13(1)	0(1)
C(14)	36(1)	63(1)	46(1)	-5(1)	6(1)	5(1)
C(15)	40(1)	76(2)	47(1)	1(1)	10(1)	-14(1)
C(16)	45(1)	53(1)	43(1)	4(1)	5(1)	-16(1)

Table S11. Hydrogen coordinates (x 10^4) and isotropic displacement parameters ($Å^2$x 10^3) for **3aa**.

	x	y	z	U(eq)
H(2A)	3570	5191	3043	47
H(3A)	3133	3580	8194	54
H(4A)	1311	2971	9542	67
H(5A)	-1253	2830	8492	76
H(6A)	-2050	3328	6130	70
H(8A)	-1373	3982	3755	55
H(10A)	1347	4857	1450	79
H(10B)	-19	4234	1142	79
H(10C)	-384	5064	1818	79
H(12A)	2970	3178	2745	55
H(13A)	4258	2337	1221	60
H(15A)	6933	4095	18	65
H(16A)	5711	4933	1586	57

9. Reference

1. (a) Wu, B.; Wen, X.; Chen, H.; Hu, L. *Org. Chem. Front.* **2021**, *8*, 5124-5129. (b) Segovia, C.; Godemert, J.; Brière, J. -F.; Levacher, V.; Oudeyer, S. *Adv. Synth. Catal.* **2022**, *364*, 3794-3799. (c) Protich, Z.; Lowder, L. L.; Huges, R. P.; Wu, J. *Chem. Sci.* **2023**, *14*, 5196-5203.

2. (a) Zheng, Y. -L.; Newman, S. G. *Angew. Chem. Int. Ed.* **2019**, *58*, 18159-18164. (b) Teng, B.; Chen, W.; Dong, S.; Kee, C. W.; Gandamana, D. A.; Zong, L.; Tan, C. H. *J. Am. Chem. Soc.* **2016**, *138*, 9935-9940. (c) Fillion, E.; Fishlock, D. *Org. Lett.* **2003**, *5*, 4653-4656.

3. (a) Kawatsura, M.; Hartwig, J. F. *J. Am. Chem. Soc.* **1999**, *121*, 1473-1478. (b) Yuan, J.; Liu, C.; Chen, Y.; Zhang, Z.; Yan, D.; Zhang, W. *Tetrahedron* **2019**, *75*, 269-277.

4. Zhou, Z. S.; Li, L.; He, X. H. *Chin. Chem. Lett.* **2012**, *23*, 12123-1216.

5. (a) Banwell, M. G.; Phillis, A. T.; Willis, A. C. *Org. Lett.* **2006**, *23*, 5341-5344. (b) Zhu, Y. -F.; Geng, X. -L.; Guan, Y. -H.l Teng, W.l Fan, X. *Synlett* **2017**, *28*, 1821-1827. (c) Austin, R. N.; Clark, T. J.; Dickson, T. E.; Killian, C. M.; Nile, T. A.; Schabacker, D. L.; McPhail, A. T. *J. Organometal. Chem.* **1995**, *491*, 11-18. (d) Nejrotto, S.; Iannicelli, M.; Jamil, S. S., Arnodo, D.; Blangetti, M.; Prandi, C. *Green Chem.* **2020**, *22*, 110-117.

6. Lin, A.; Ghosh, A.; Yellen, S.; Ball, Z. T.; Kürti, L. *J. Am. Chem. Soc.* **2024**, *146*, 21129–21136.

7. (a) Chen, Q.; Tanaka, S.; Fujita, T.; Chen, L.; Minato, T.; Ishikawa, M.; Chen, M.; Asao, N.; Yamamoto, Y.; Jin, T. *Chem. Commun.* **2014**, *50*, 3344-3346. (b) Pérez, M.; Qu, Z.; Caputo, C. B.; Podgorny, V.; Hounjet, L. J.; Hansen, A.; Dobrovetsky, R.; Grimme, S.; Stephan, D. W. *Chem. Eur. J.* **2015**, *21*, 6491–6500. (c) Han, Y.; Corey, E. J. *Org. Lett.* **2019**, *21*, 283–286. (d) Tuokko, S.; Pihko, P. M. *Org. Process Res. Dev.* **2014**, *18*, 1740–1751. (e) Poisson, T.; Gembus, V.; Dalla, V.; Oudeyer, S.; Levacher, V. *J. Org. Chem.* **2010**, *75*, 7704– 7716.

8. Yang, T.; Fan, X.; Zhao, X.; Yu, W. *Org. Lett.* **2018**, *20*, 1875–1879.

9. (a) Sheng, M.; Frurip, D.; Gorman, D. *J. Loss Prev. Process Ind.* **2015**, *38*, 114-118. (b) Ullrich, R.; Grewer, Th. *Thermochim. Acta,* **1993**, *225*, 201-211.

10. Liu, C. -H.; Wang, Z.; Xiao, L. -Y.; Mukadas; Zhu, D.-S.; Zhao, Y. -L. *Org. Lett.* **2018**, *20*, 4862-4866.

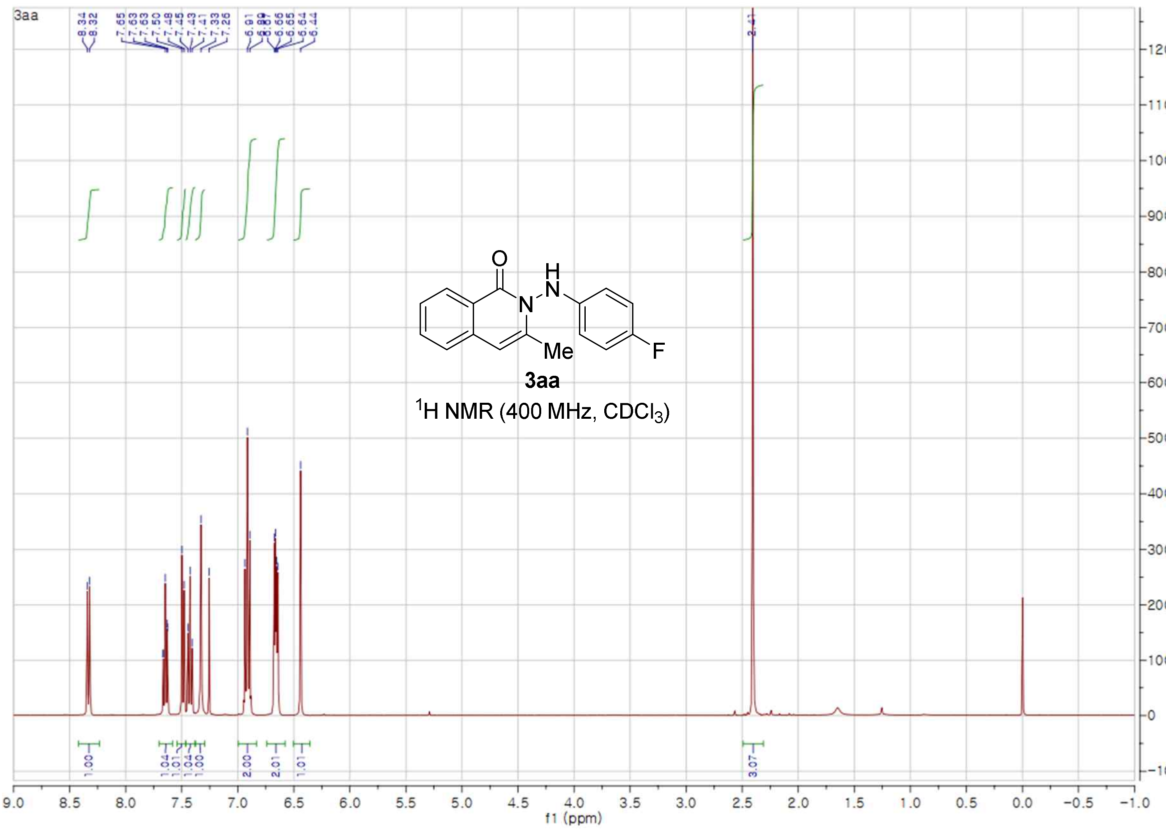
3aa
3aa
¹H NMR (400 MHz, CDCl₃)
f1 (ppm)

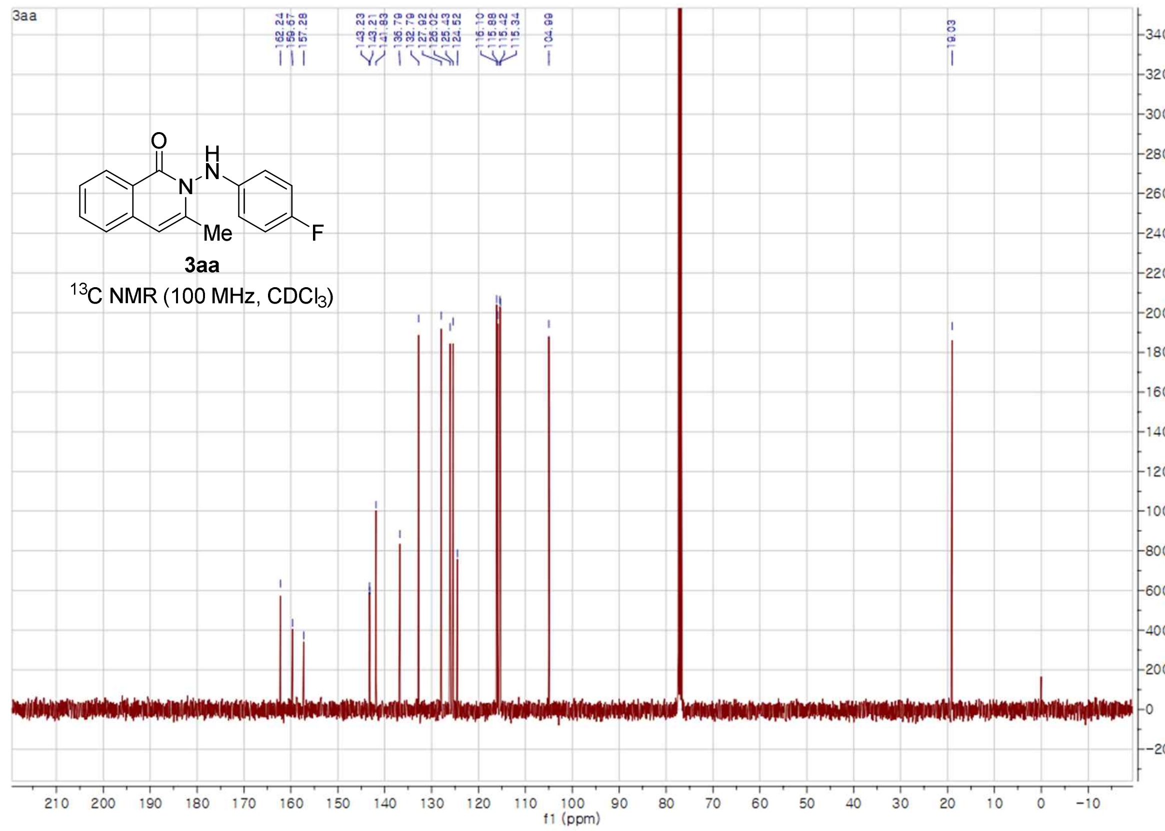
3aa
3aa
¹³C NMR (100 MHz, CDCl₃)
f1 (ppm)

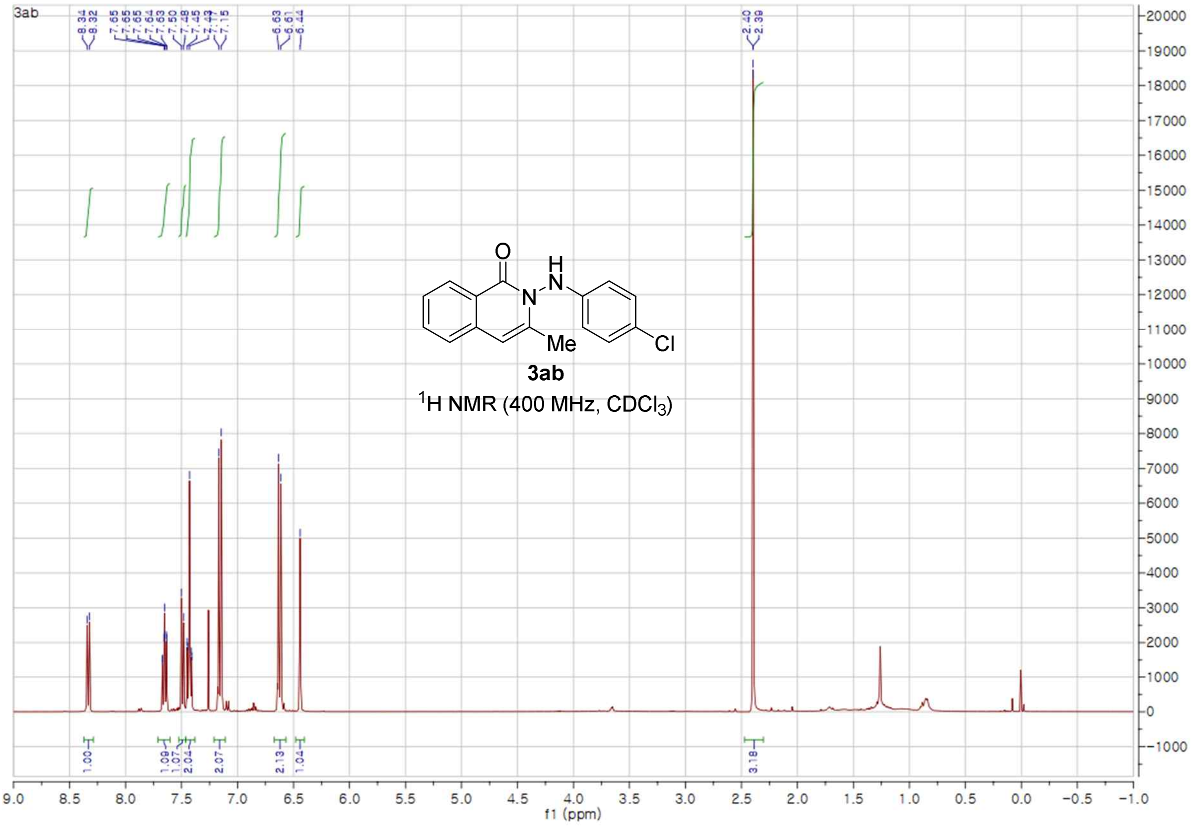
3ab
8.34
8.32
7.65
7.65
7.65
7.64
7.63
7.50
7.48
7.45
7.43
7.17
7.15
6.63
6.61
6.44
2.40
2.39
O
H
N
N
Me
Cl
3ab
¹H NMR (400 MHz, CDCl₃)
1.00
1.09
1.07
2.04
2.07
2.13
1.04
3.18
f1 (ppm)

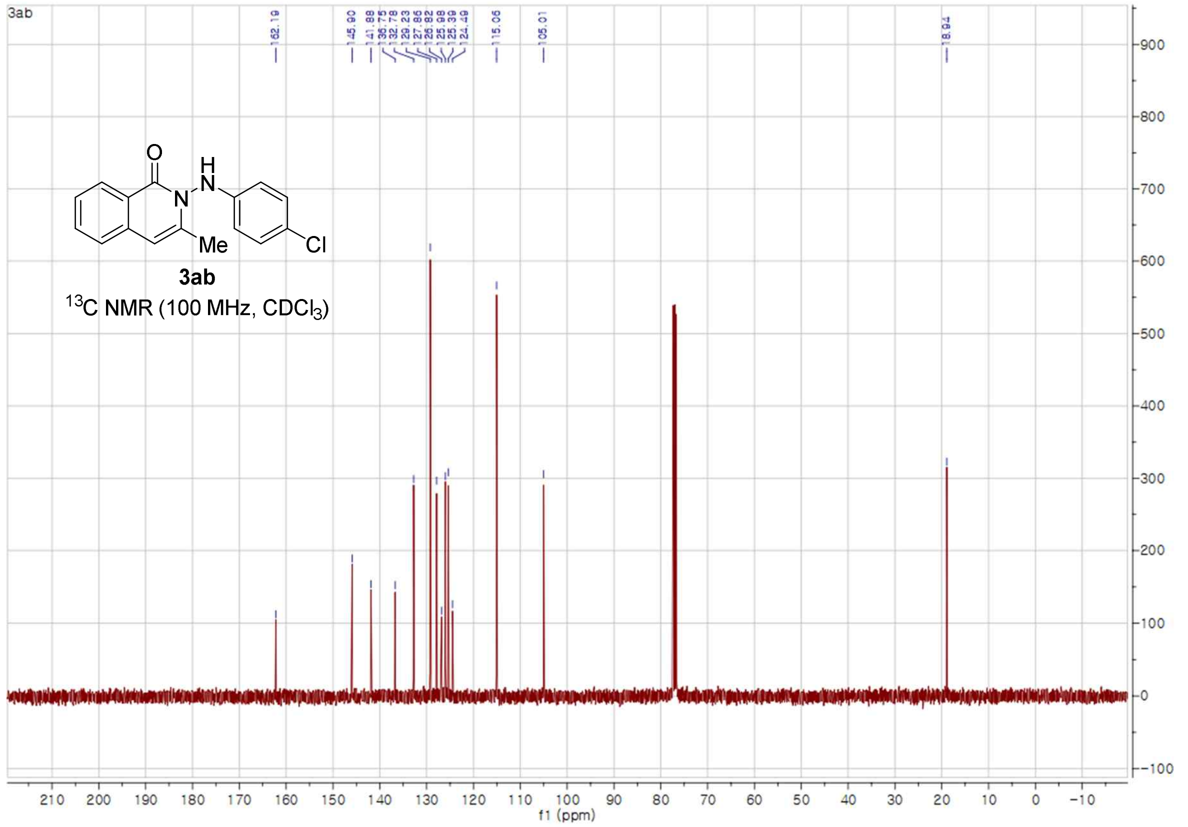
3ab
162.19
145.90
141.88
136.75
132.78
129.23
127.86
126.82
125.98
125.39
124.49
115.06
105.01
18.94
O
H
N
N
Me
Cl
3ab
¹³C NMR (100 MHz, CDCl₃)
f1 (ppm)

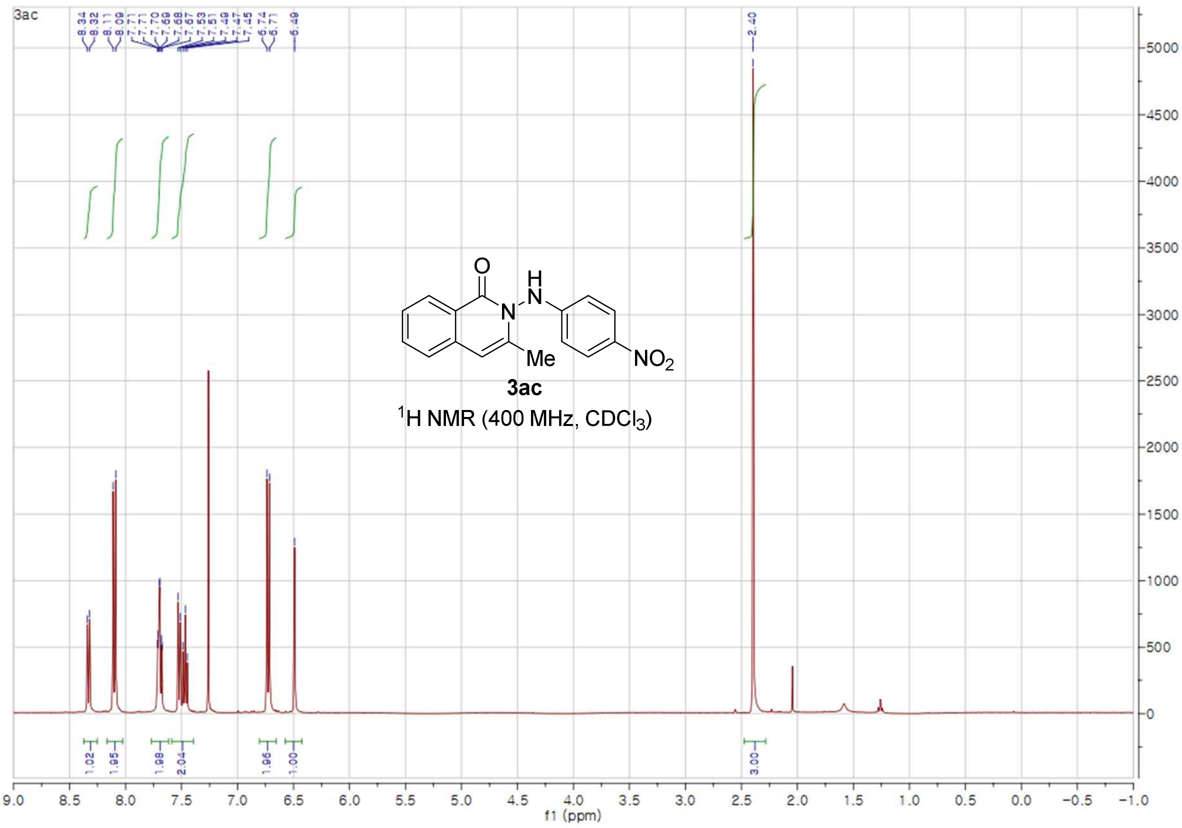
3ac
8.34 8.32 8.11 8.09 7.71 7.70 7.69 7.68 7.67 7.53 7.51 7.49 7.47 7.45 6.74 6.71 6.49
2.40
3ac
^{1}H NMR (400 MHz, CDCl$_3$)
1.02 1.95 1.98 2.04 1.96 1.00 3.00
f1 (ppm)

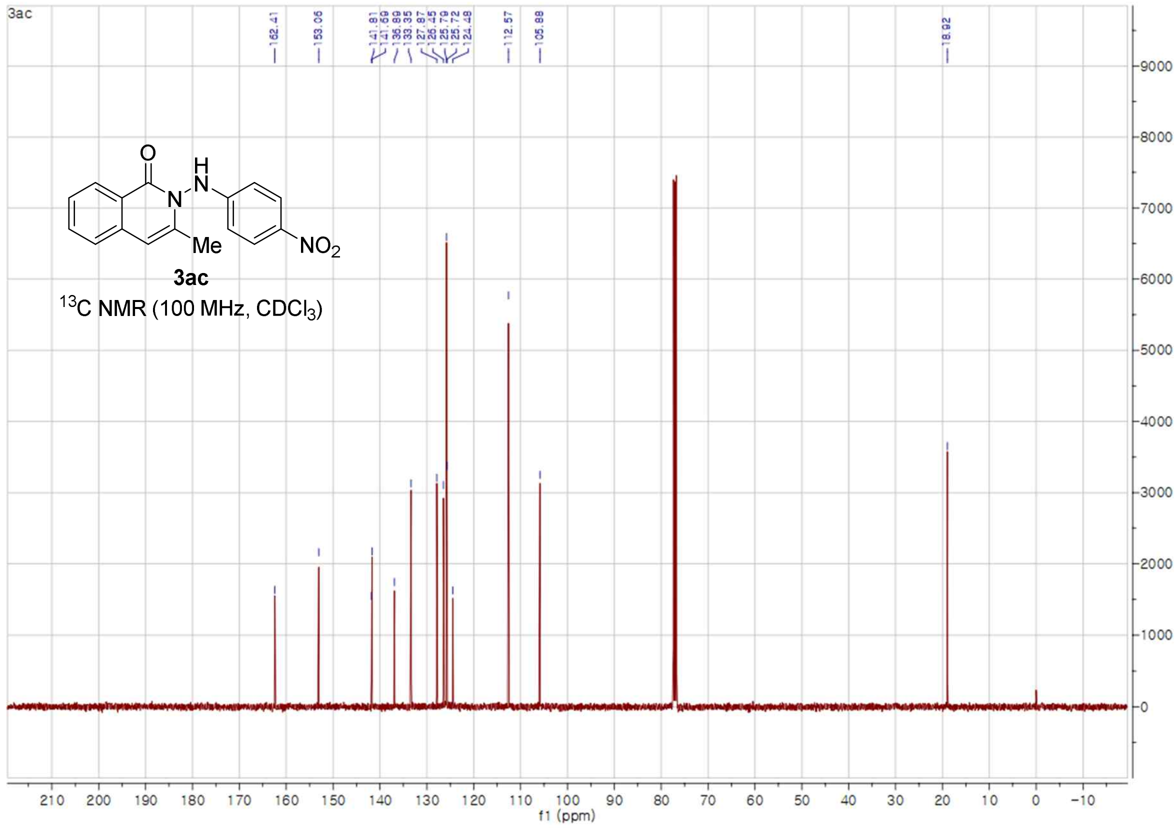
3ac
162.41 153.06 141.81 141.69 136.89 133.35 127.87 126.45 125.79 125.72 124.48 112.57 105.88 18.92
3ac
^{13}C NMR (100 MHz, CDCl$_3$)
f1 (ppm)

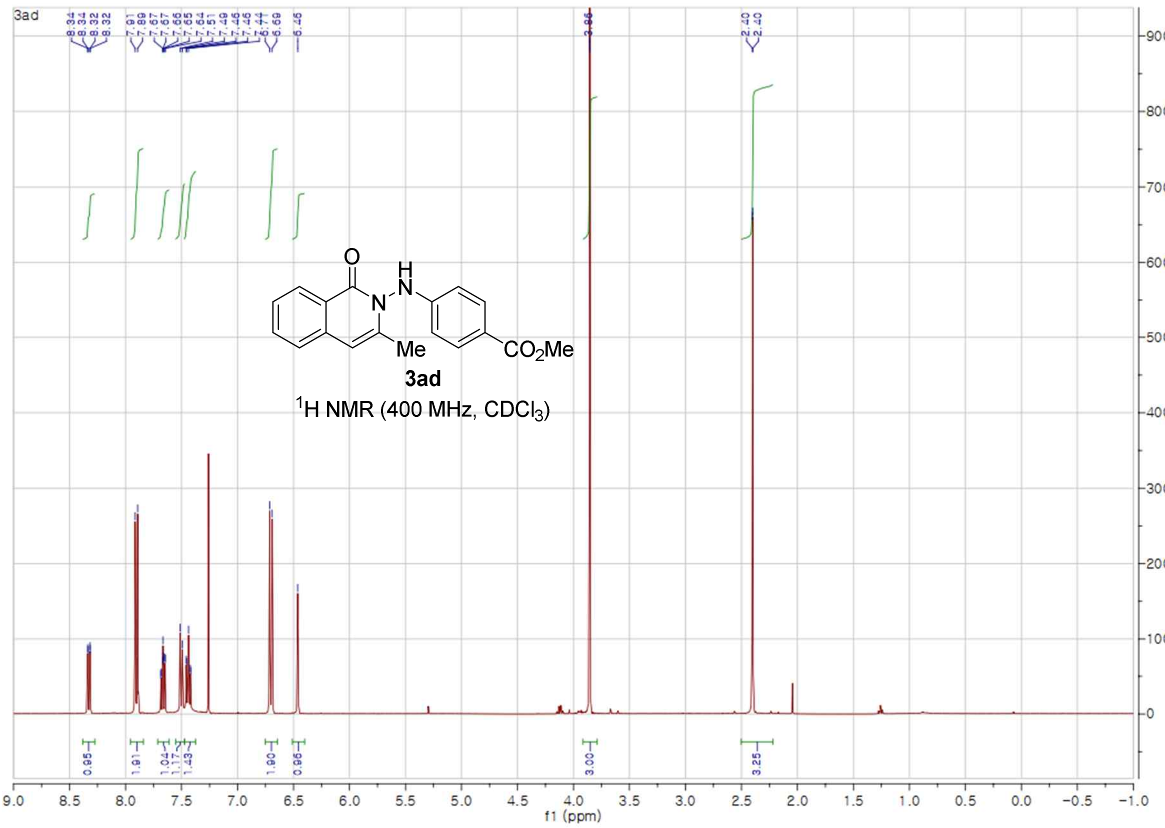
3ad
8.34
8.34
8.32
8.32
7.91
7.89
7.67
7.66
7.65
7.64
7.51
7.49
7.46
7.44
6.71
6.69
6.46
3.86
2.40
2.40
O
H
N
N
Me
CO2Me
3ad
1H NMR (400 MHz, CDCl3)
0.95
1.91
1.04
1.17
1.43
1.90
0.96
3.00
3.25
f1 (ppm)

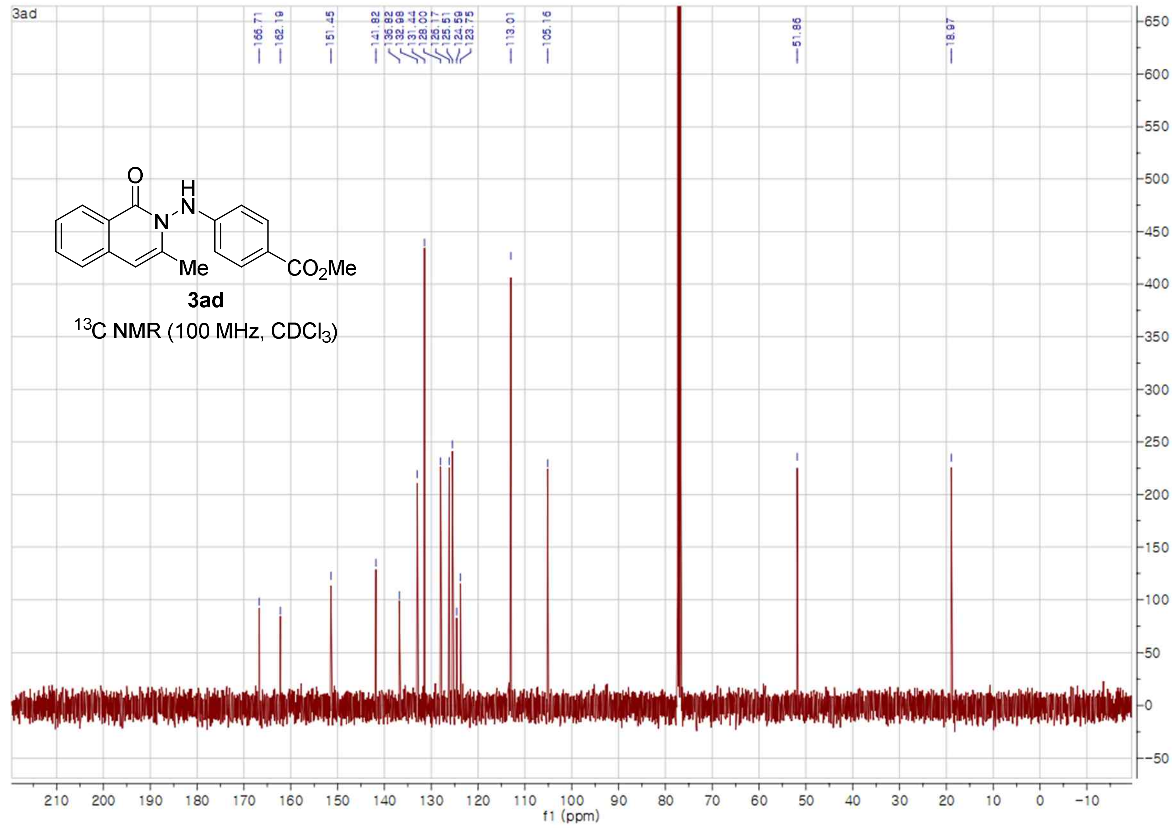
3ad
166.71
162.19
151.45
141.82
135.82
132.98
131.44
128.00
126.17
125.51
124.59
123.75
113.01
105.16
51.86
18.97
O
H
N
N
Me
CO2Me
3ad
13C NMR (100 MHz, CDCl3)
f1 (ppm)

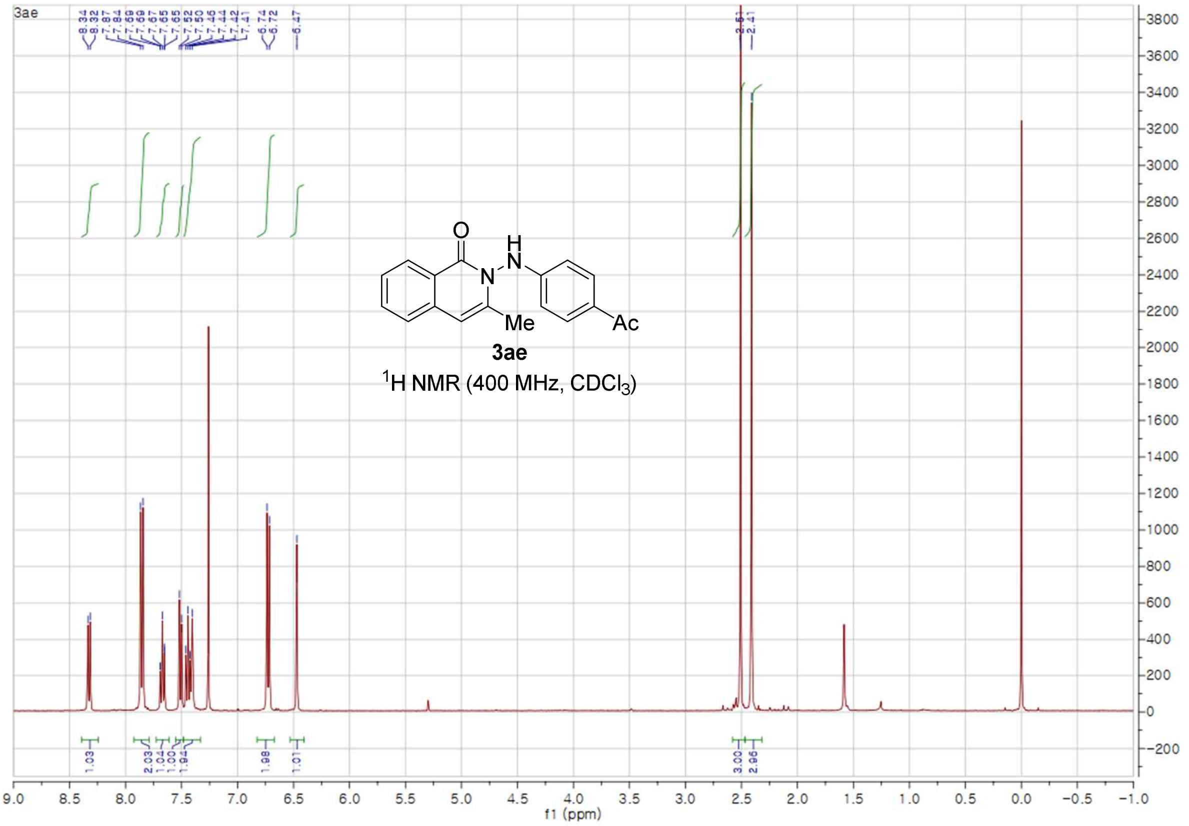
3ae
8.34
8.32
7.87
7.84
7.69
7.69
7.67
7.65
7.65
7.52
7.50
7.46
7.44
7.42
7.41
6.74
6.72
6.47
2.51
2.41
O
H
N
N
Me
Ac
3ae
1H NMR (400 MHz, CDCl3)
1.03
2.03
1.04
1.00
1.94
1.98
1.01
3.00
2.96
f1 (ppm)

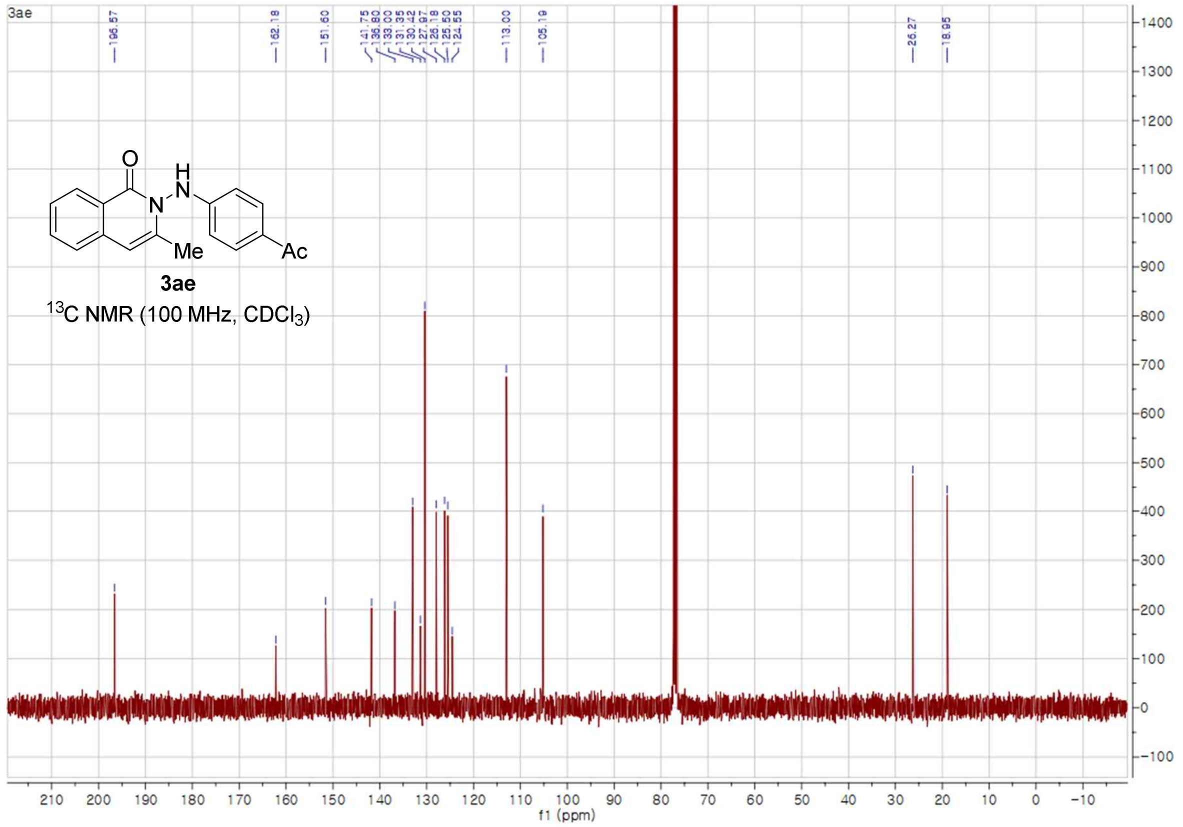
3ae
196.57
162.18
151.60
141.75
136.80
133.00
131.35
130.42
127.97
126.18
125.50
124.55
113.00
105.19
26.27
18.95
O
H
N
N
Me
Ac
3ae
13C NMR (100 MHz, CDCl3)
f1 (ppm)

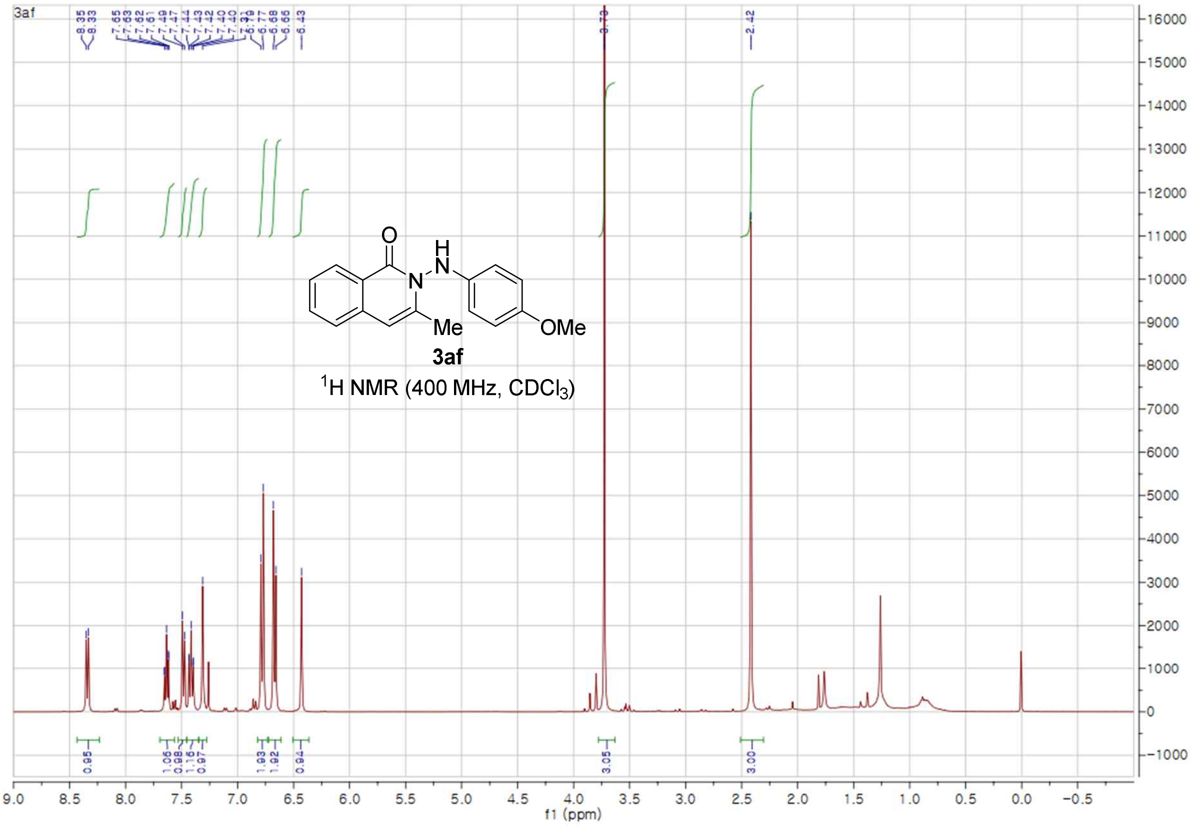
3af
8.35
8.33
7.65
7.63
7.62
7.61
7.49
7.47
7.44
7.43
7.42
7.40
7.31
6.79
6.77
6.68
6.66
6.43
3.73
2.42
0.95
1.05
0.98
1.16
0.97
1.93
1.92
0.94
3.05
3.00
O
H
N
N
Me
OMe
3af
¹H NMR (400 MHz, CDCl₃)
f1 (ppm)

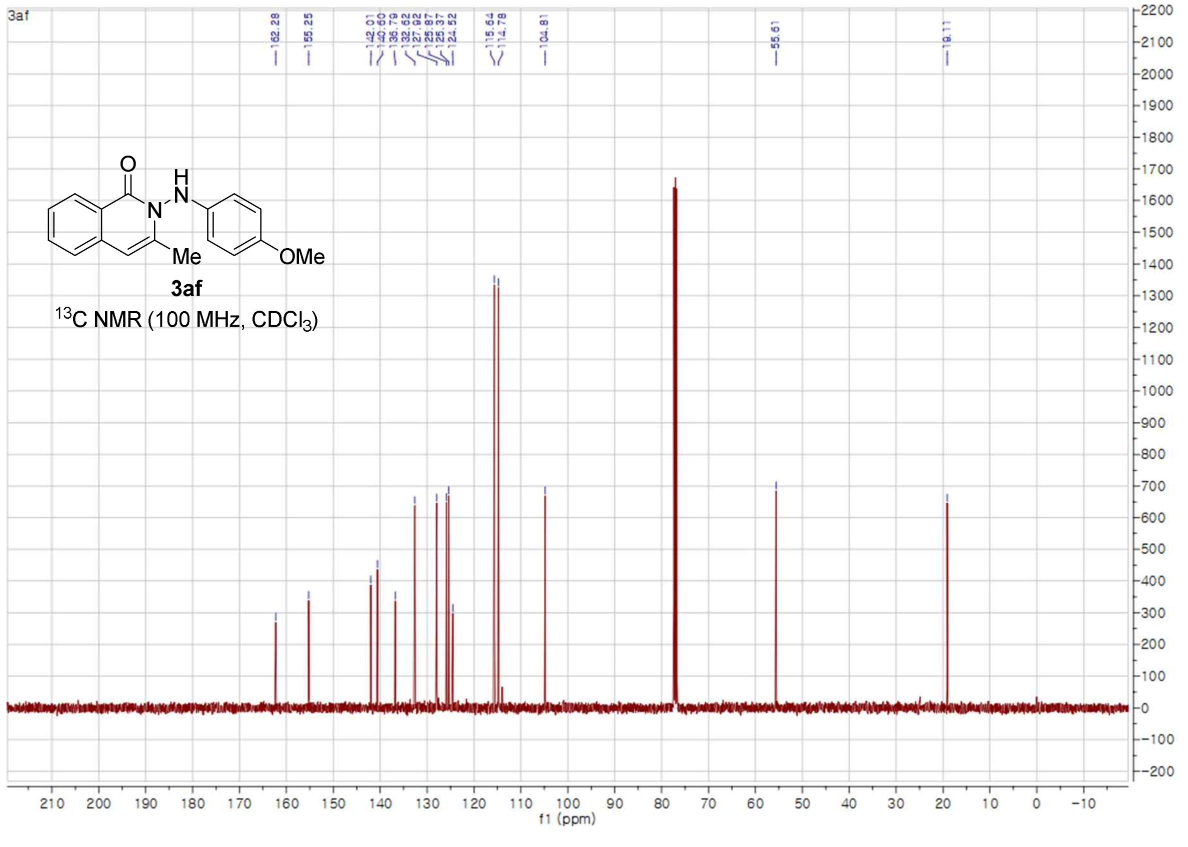
3af
162.28
155.25
142.01
140.60
136.79
132.62
127.92
125.87
125.37
124.52
115.64
114.78
104.81
55.61
19.11
O
H
N
N
Me
OMe
3af
¹³C NMR (100 MHz, CDCl₃)
f1 (ppm)

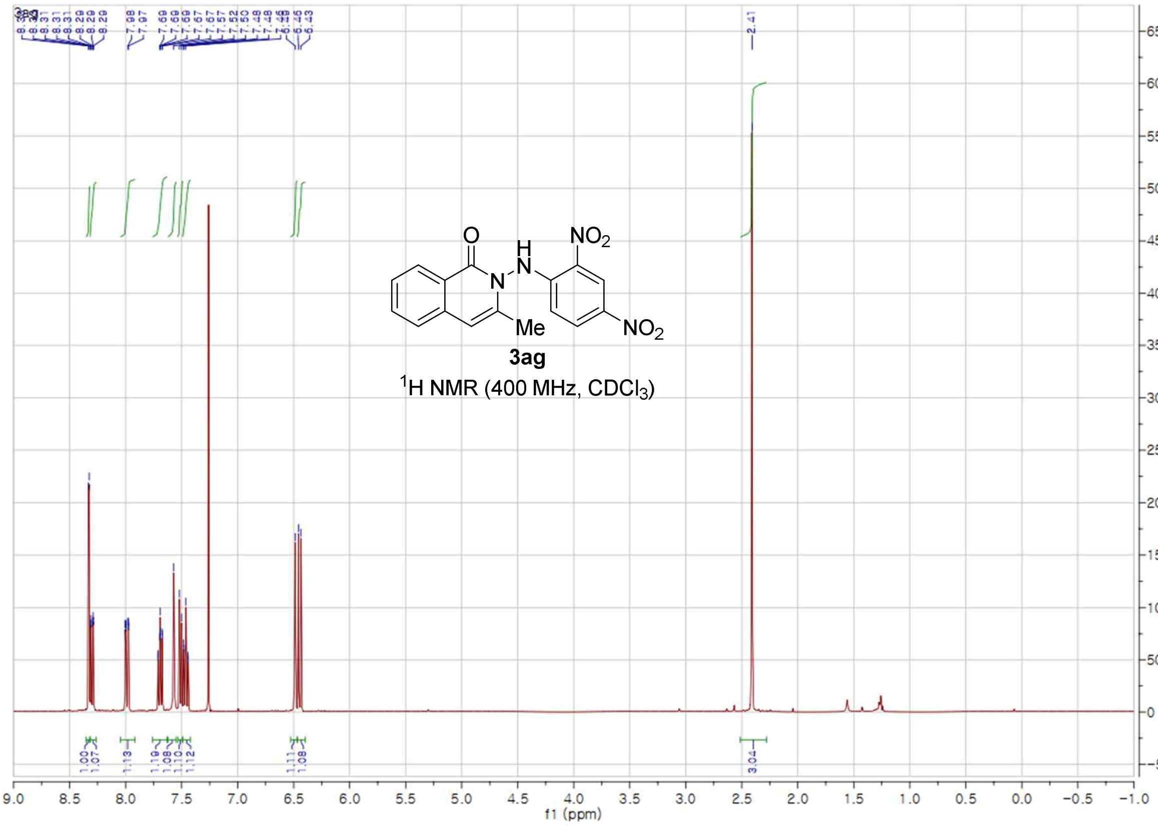

3ag
2.41
O
H
N
N
NO2
Me
NO2
3ag
1H NMR (400 MHz, CDCl3)
f1 (ppm)

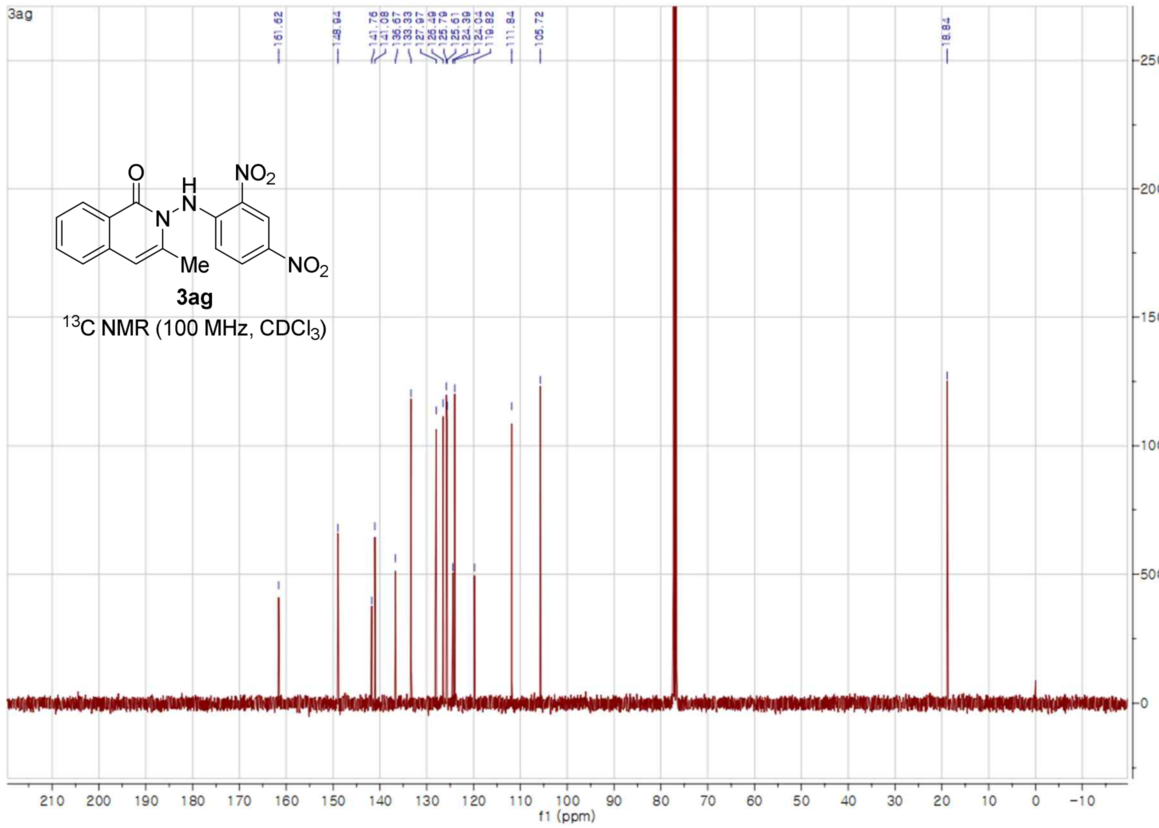

3ag
161.62
148.94
105.72
18.84
O
H
N
N
NO2
Me
NO2
3ag
13C NMR (100 MHz, CDCl3)
f1 (ppm)

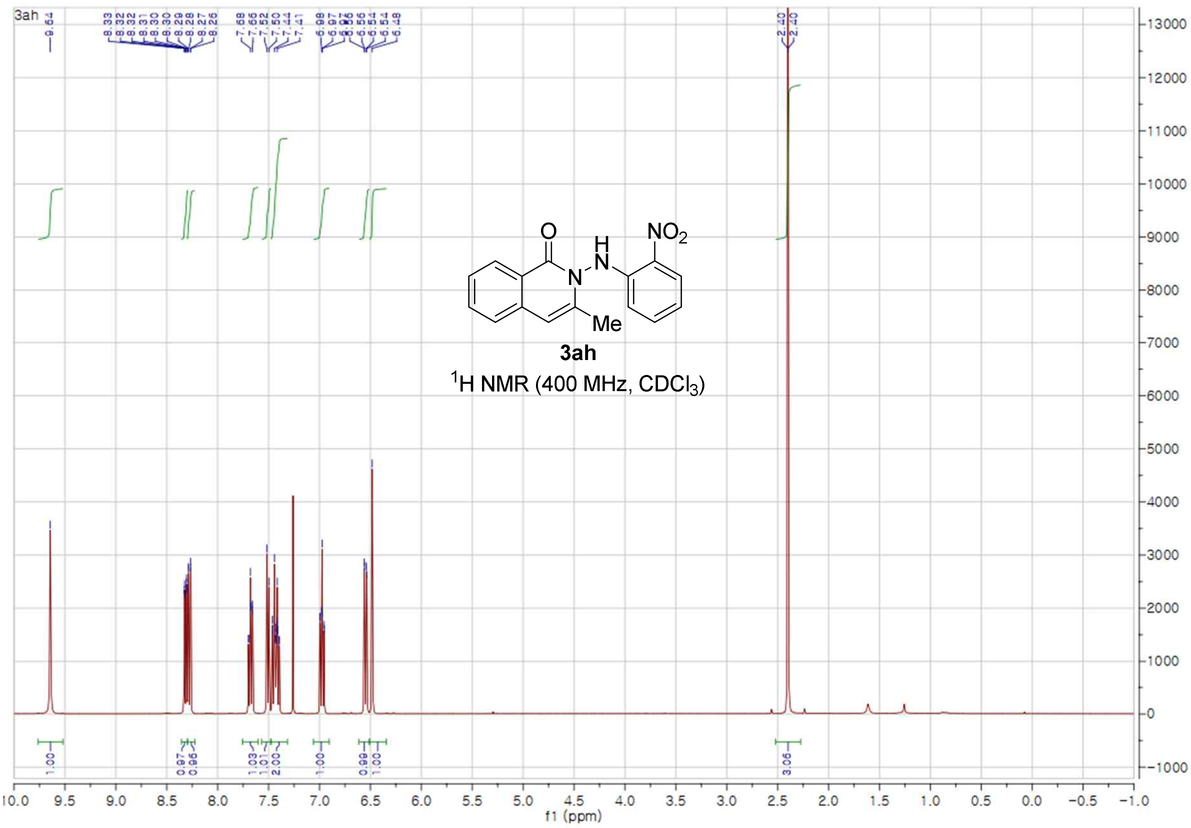
3ah
NO2
Me
3ah
1H NMR (400 MHz, CDCl3)
f1 (ppm)

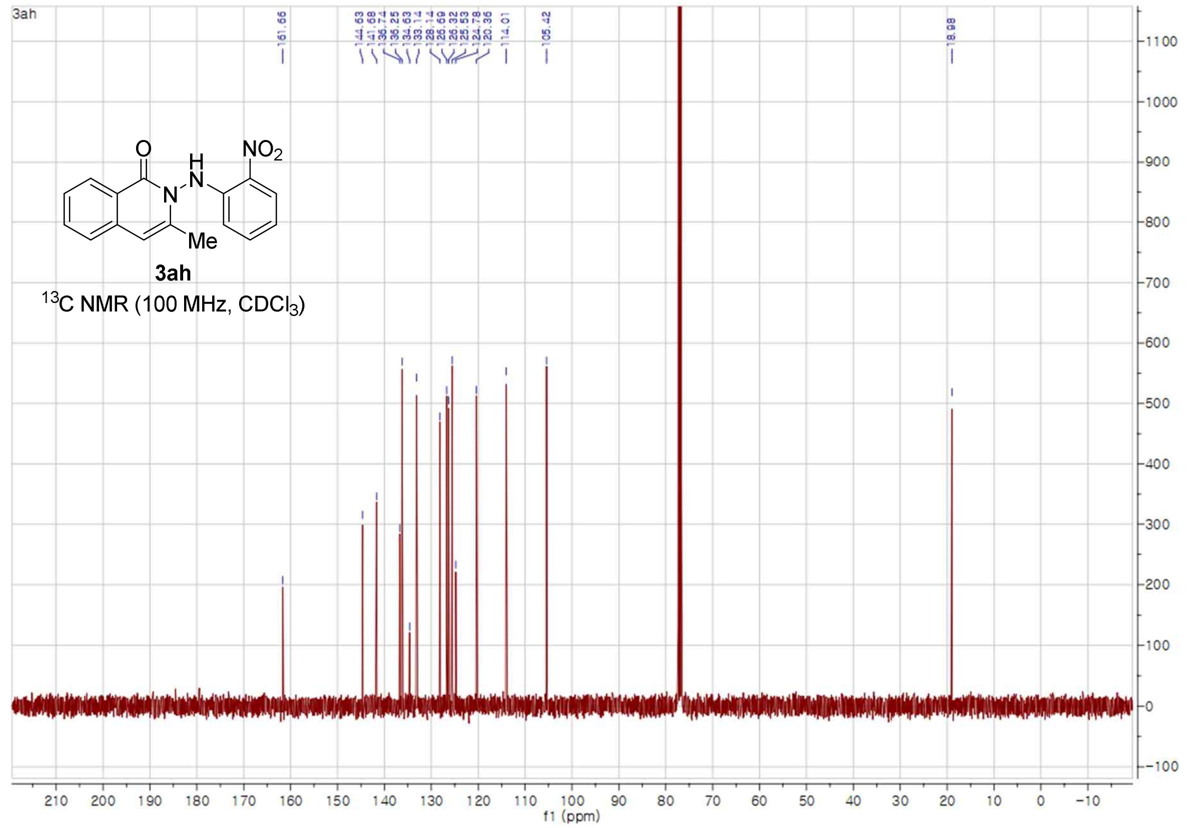
3ah
NO2
Me
3ah
13C NMR (100 MHz, CDCl3)
f1 (ppm)

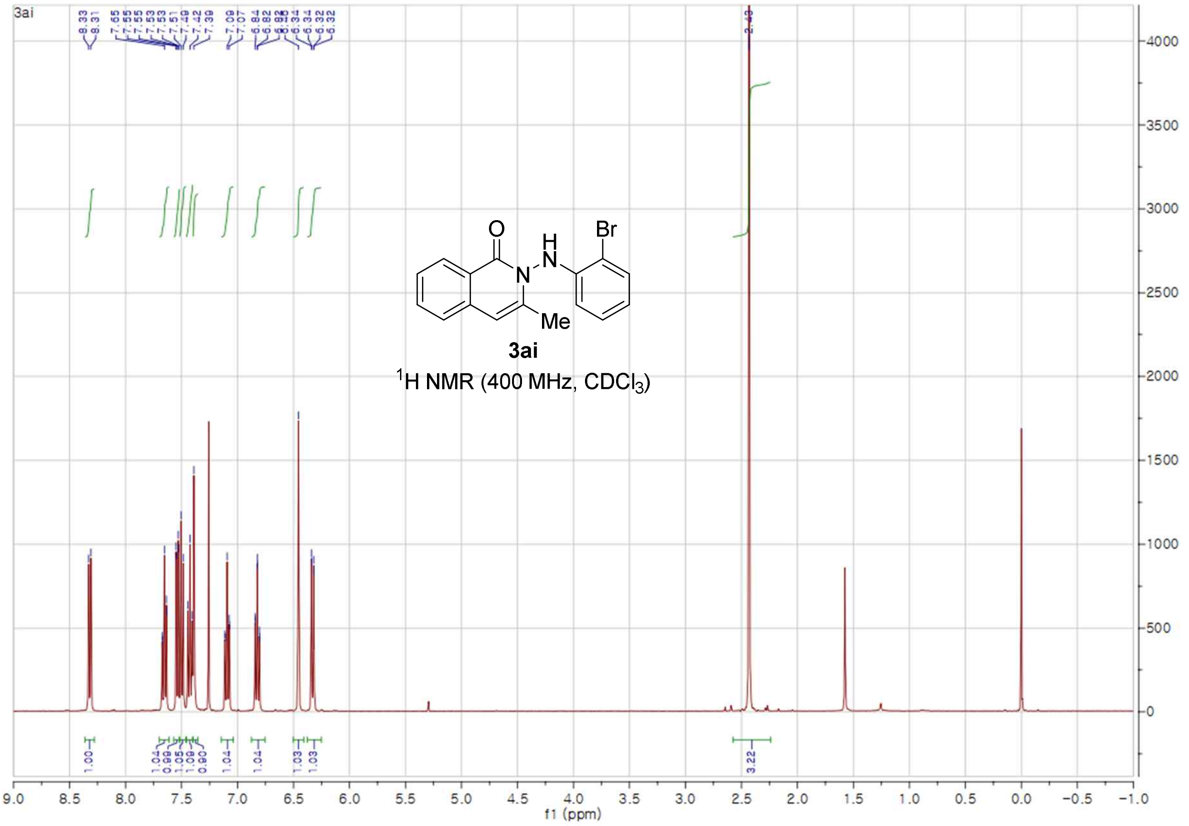
3ai
8.33
8.31
7.65
7.55
7.55
7.53
7.53
7.51
7.49
7.42
7.39
7.09
7.07
6.84
6.82
6.34
6.34
6.32
6.32
O
H
N
N
Br
Me
3ai
1H NMR (400 MHz, CDCl3)
1.00
1.04
0.98
1.05
1.09
0.90
1.04
1.04
1.03
1.03
3.22
f1 (ppm)

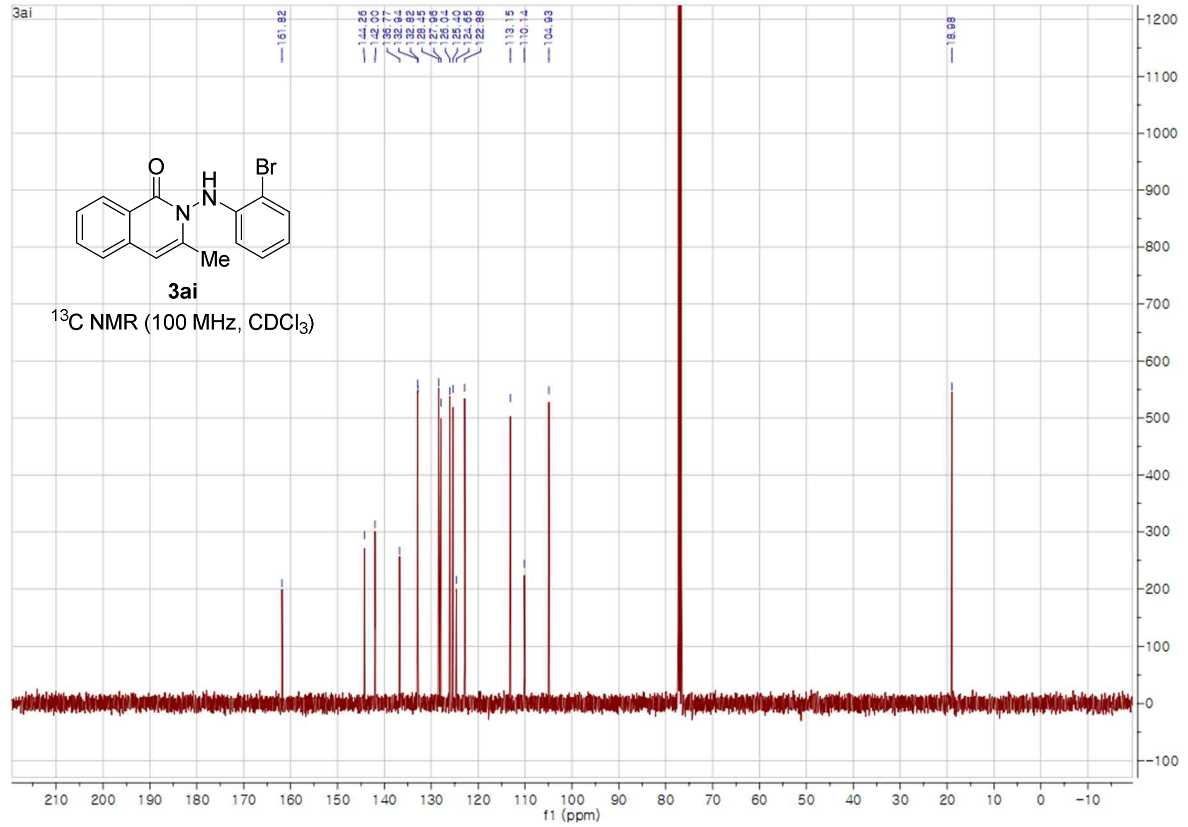
3ai
161.82
144.26
142.00
136.77
132.94
132.82
128.45
127.95
125.04
125.40
124.65
122.88
113.15
110.14
104.93
18.98
O
H
N
N
Br
Me
3ai
13C NMR (100 MHz, CDCl3)
f1 (ppm)

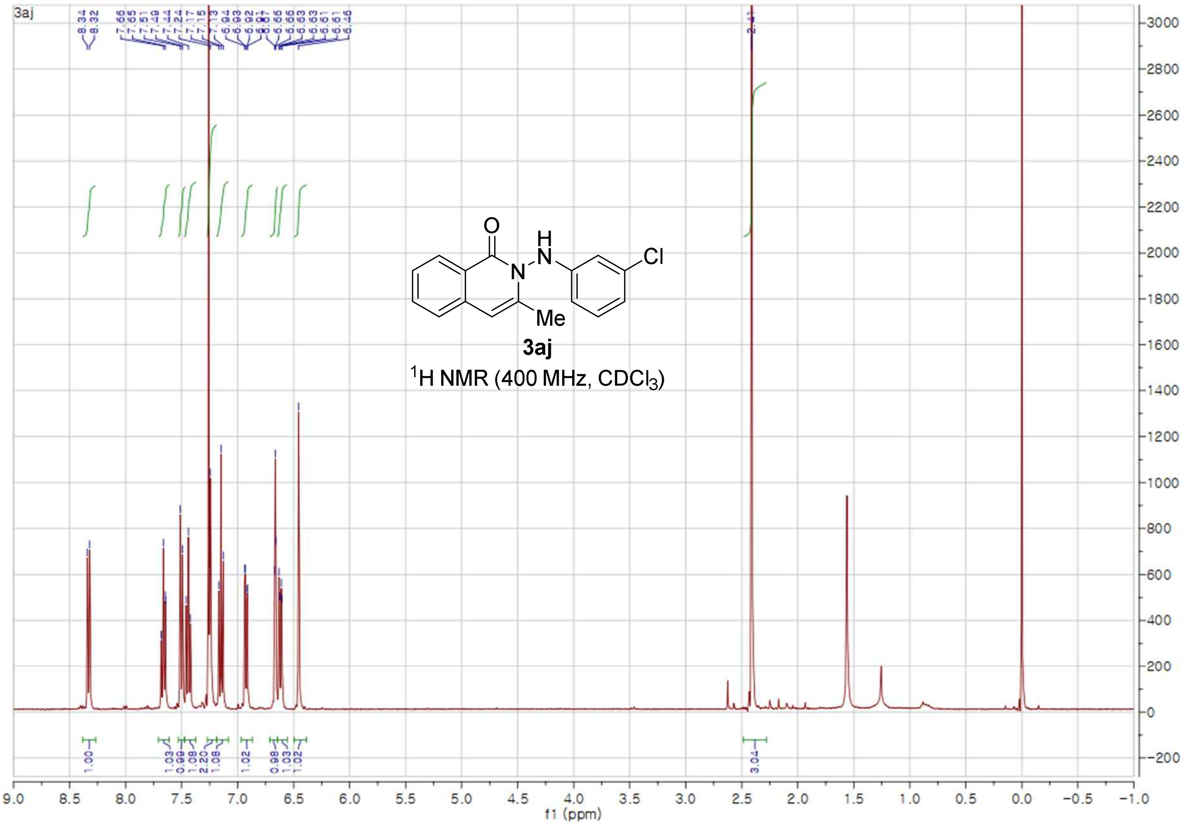
3aj
O
H
N
N
Cl
Me
3aj
^{1}H NMR (400 MHz, CDCl$_3$)
f1 (ppm)

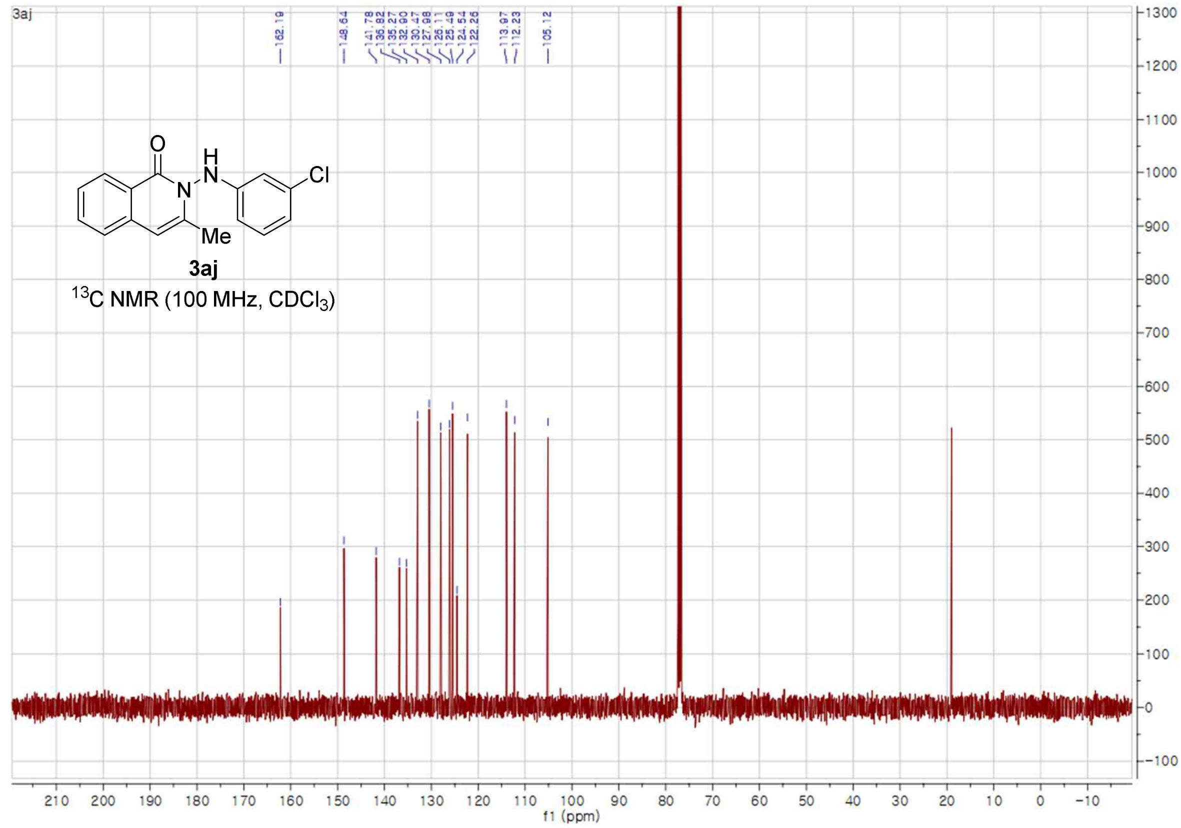
3aj
O
H
N
N
Cl
Me
3aj
^{13}C NMR (100 MHz, CDCl$_3$)
f1 (ppm)

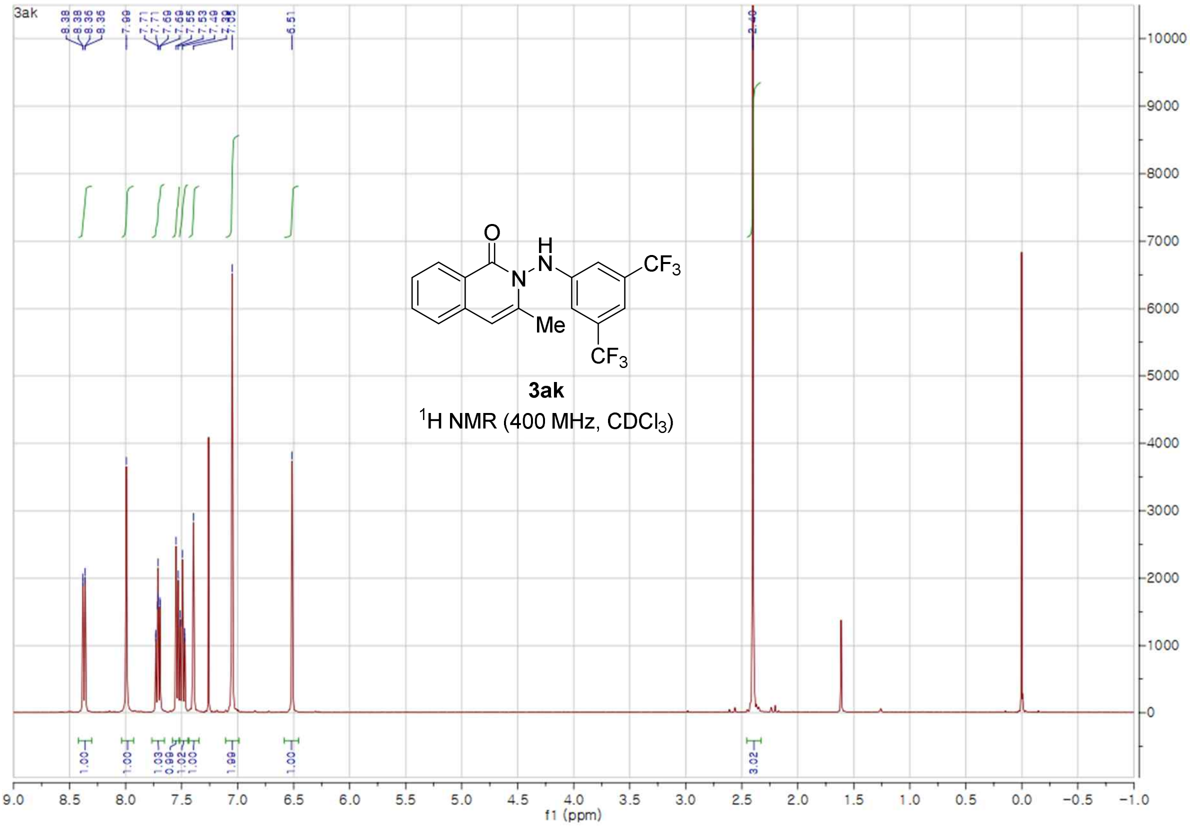
3ak
O
H
N
N
CF3
Me
CF3
3ak
1H NMR (400 MHz, CDCl3)
f1 (ppm)

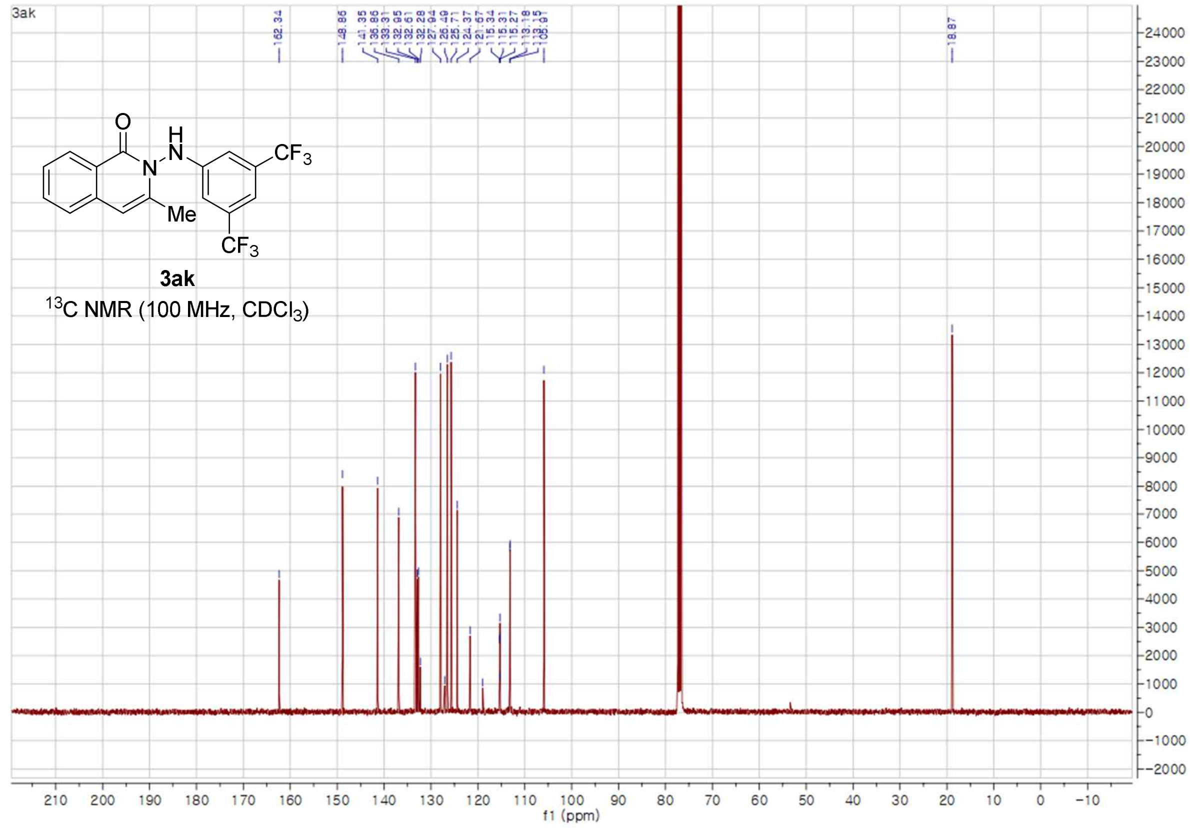
3ak
O
H
N
N
CF3
Me
CF3
3ak
13C NMR (100 MHz, CDCl3)
f1 (ppm)

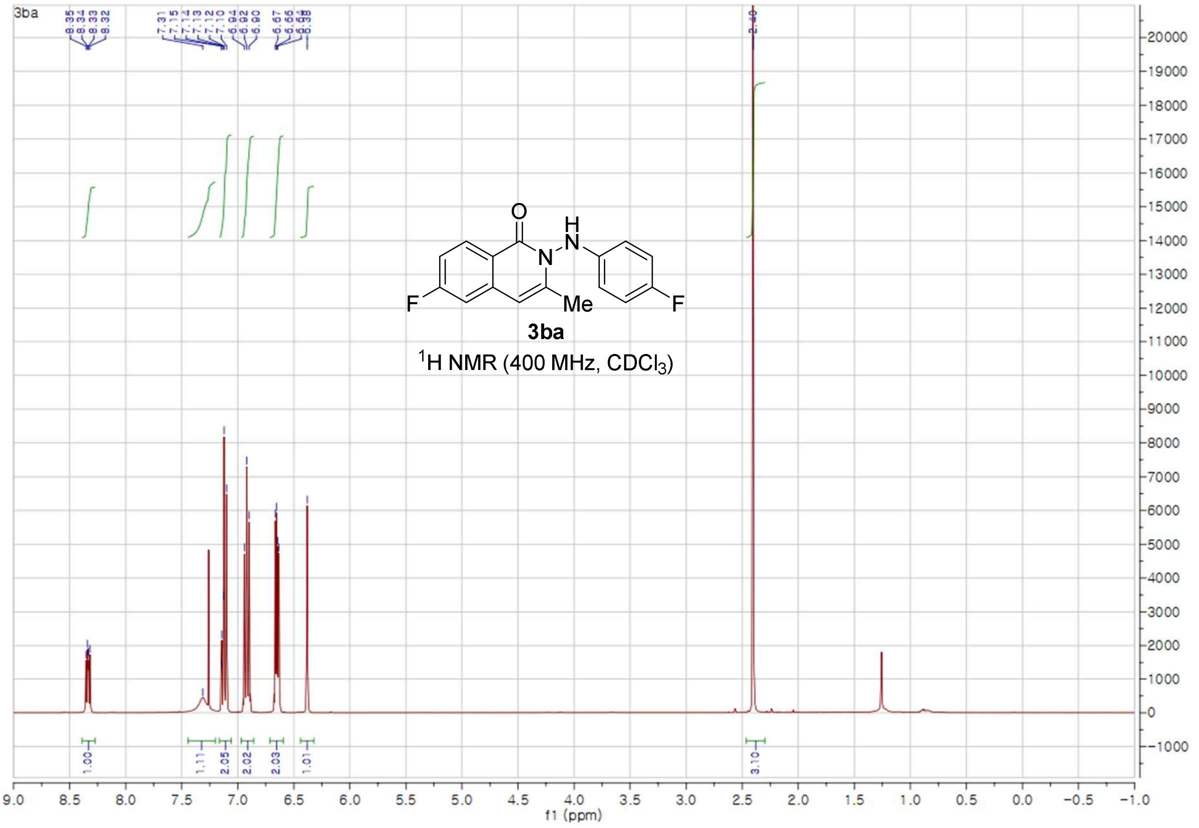
3ba
3ba
1H NMR (400 MHz, CDCl3)
f1 (ppm)

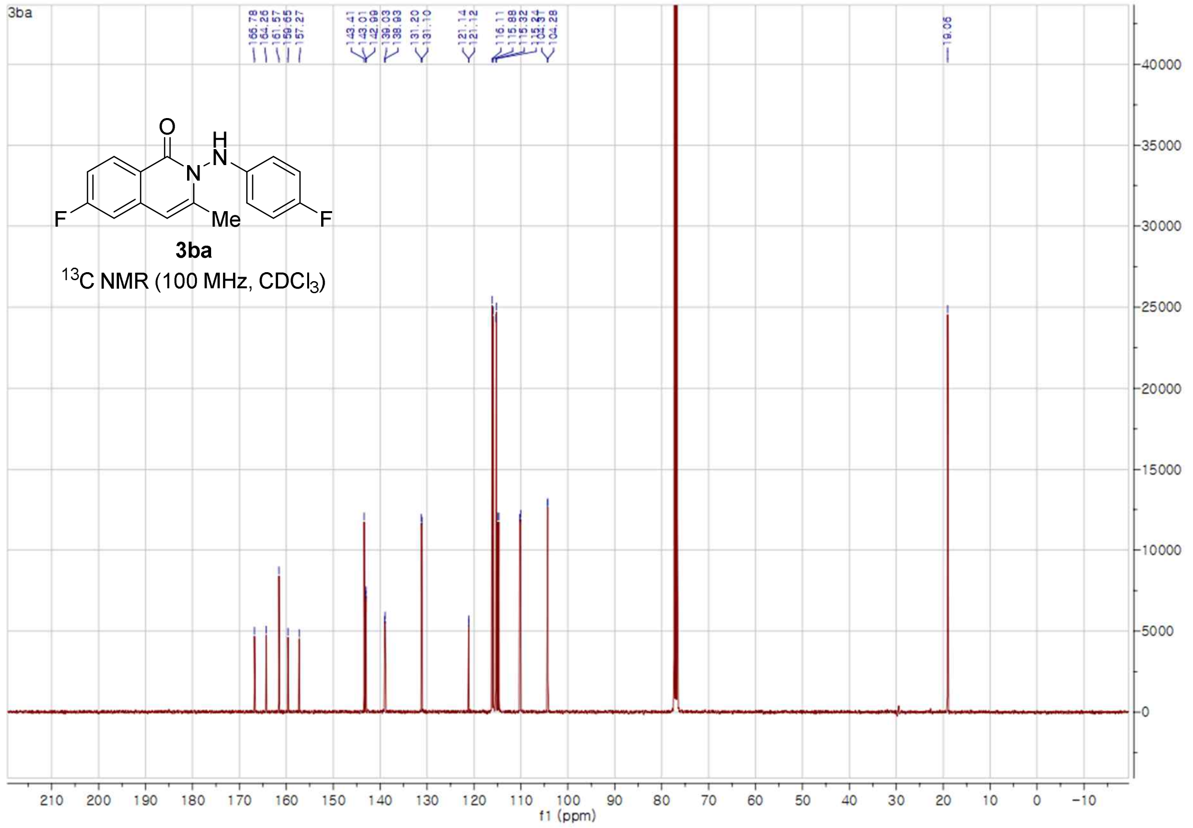
3ba
3ba
13C NMR (100 MHz, CDCl3)
f1 (ppm)

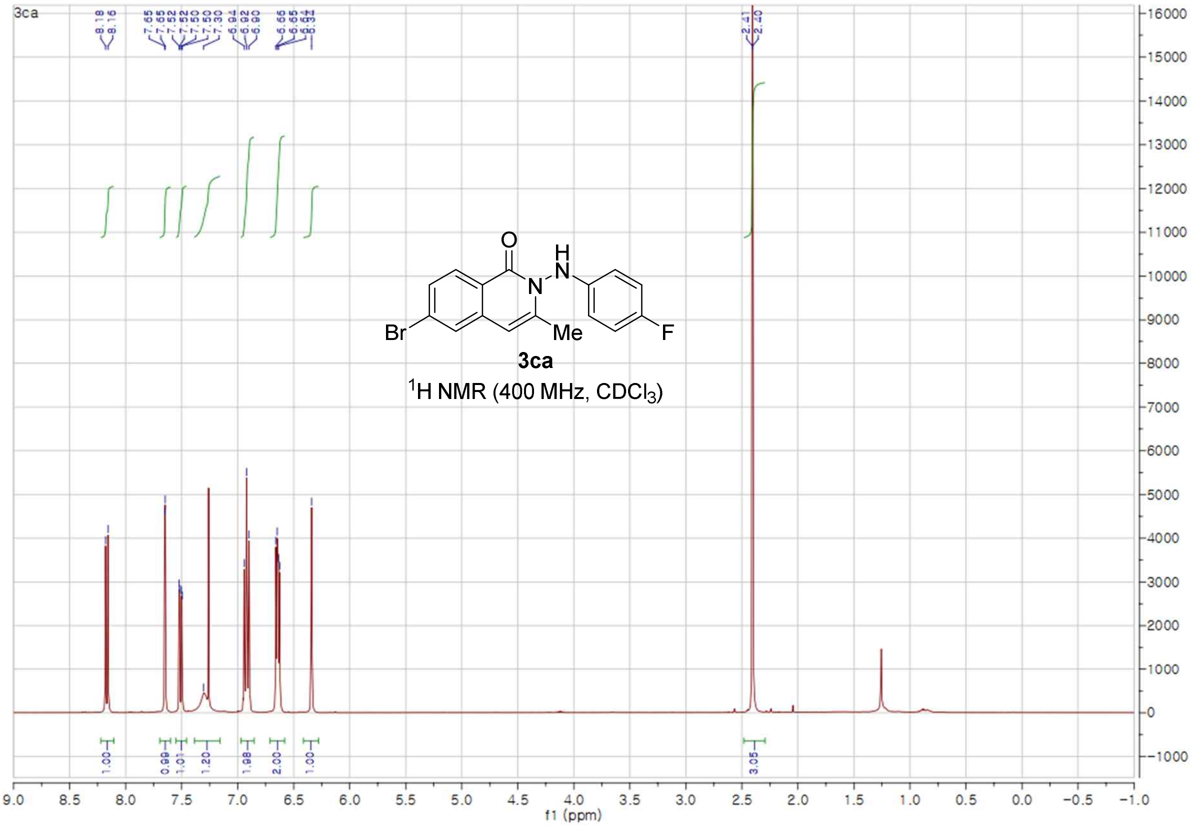
3ca
Br
Me
F
3ca
1H NMR (400 MHz, CDCl3)
f1 (ppm)

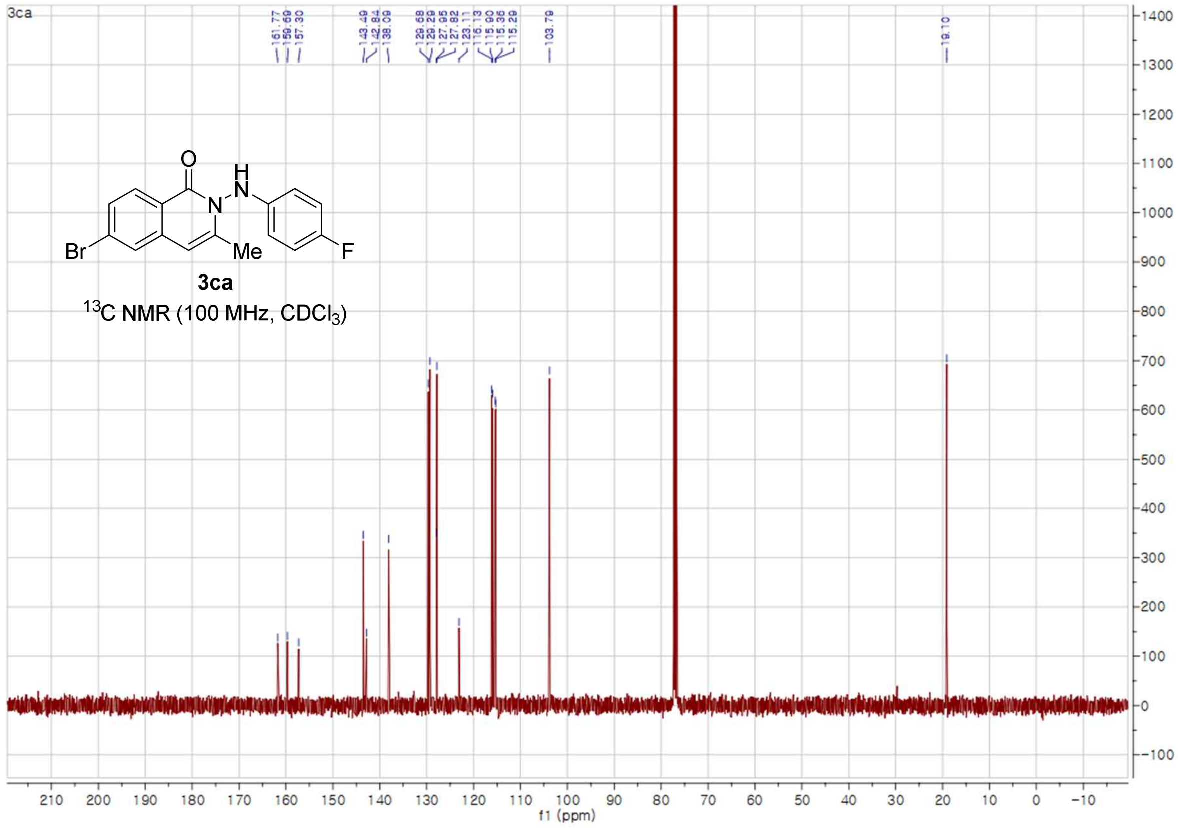
3ca
Br
Me
F
3ca
13C NMR (100 MHz, CDCl3)
f1 (ppm)

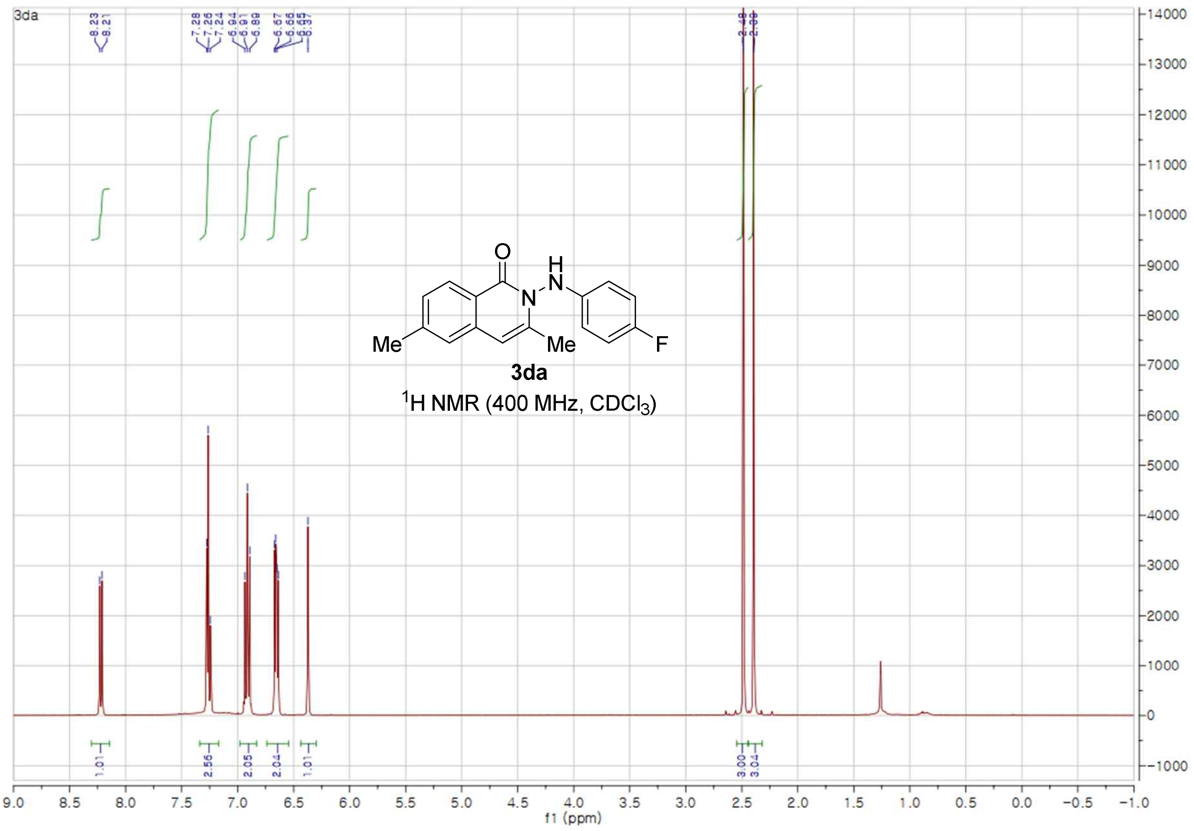

3da
8.23
8.21
7.28
7.26
7.24
6.94
6.91
6.89
6.67
6.66
6.65
6.37
3da
^{1}H NMR (400 MHz, $CDCl_3$)
1.01
2.56
2.05
2.04
1.01
3.00
3.04
f1 (ppm)

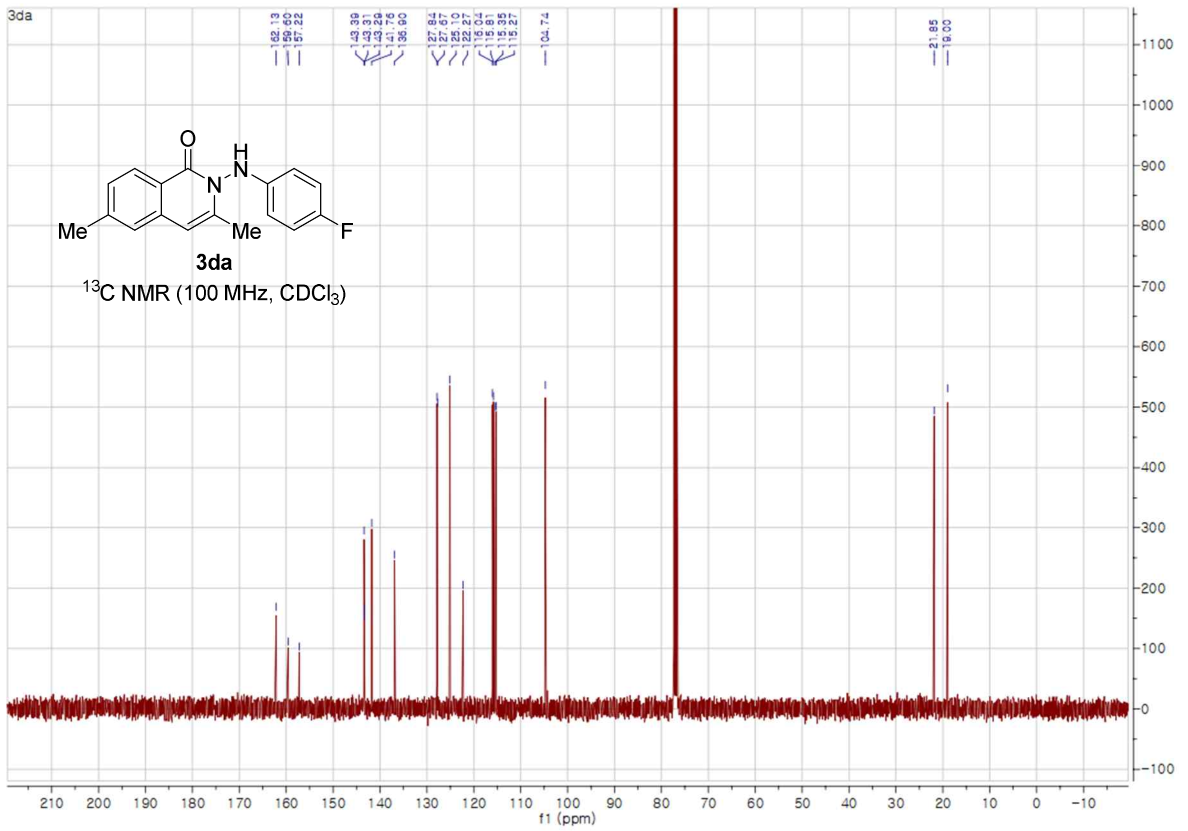

3da
162.13
159.60
157.22
143.39
143.31
143.29
141.76
136.90
127.84
127.57
125.10
122.27
116.04
115.81
115.35
115.27
104.74
21.85
19.00
3da
^{13}C NMR (100 MHz, $CDCl_3$)
f1 (ppm)

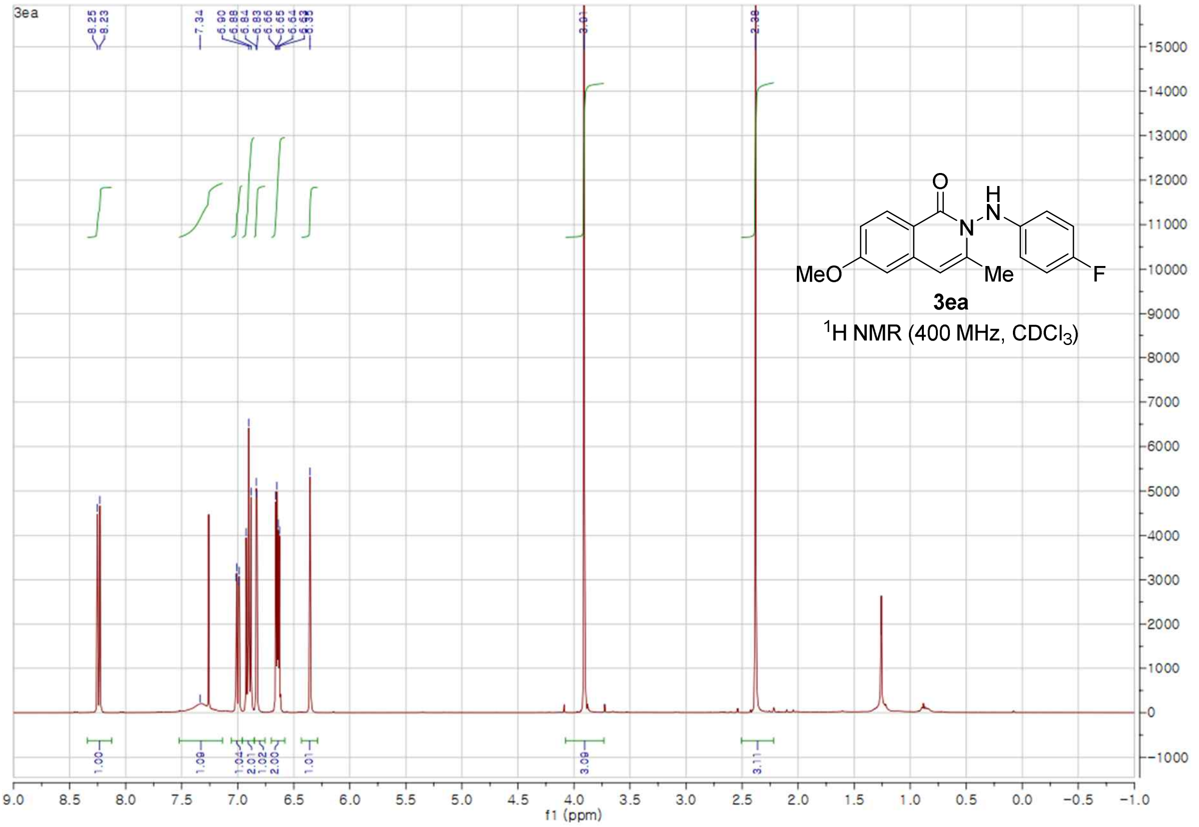

3ea
3ea
¹H NMR (400 MHz, CDCl₃)
f1 (ppm)

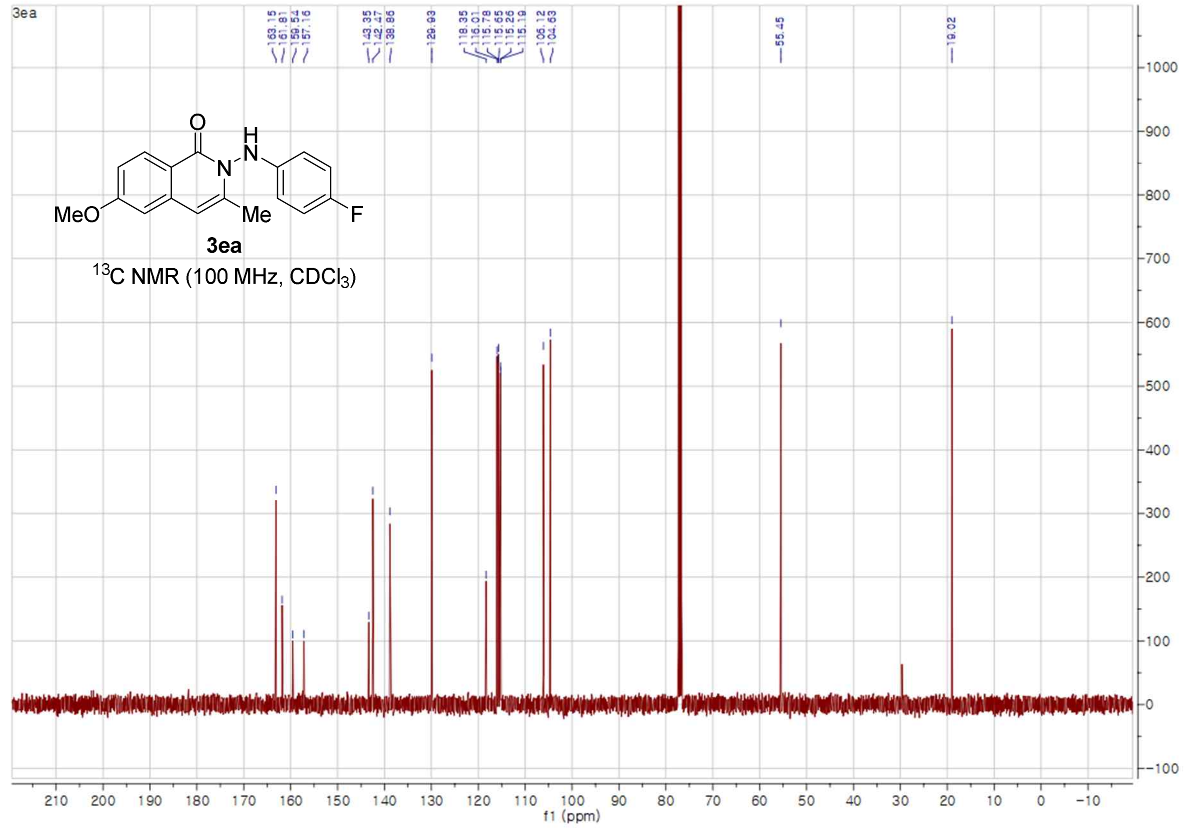

3ea
3ea
¹³C NMR (100 MHz, CDCl₃)
f1 (ppm)

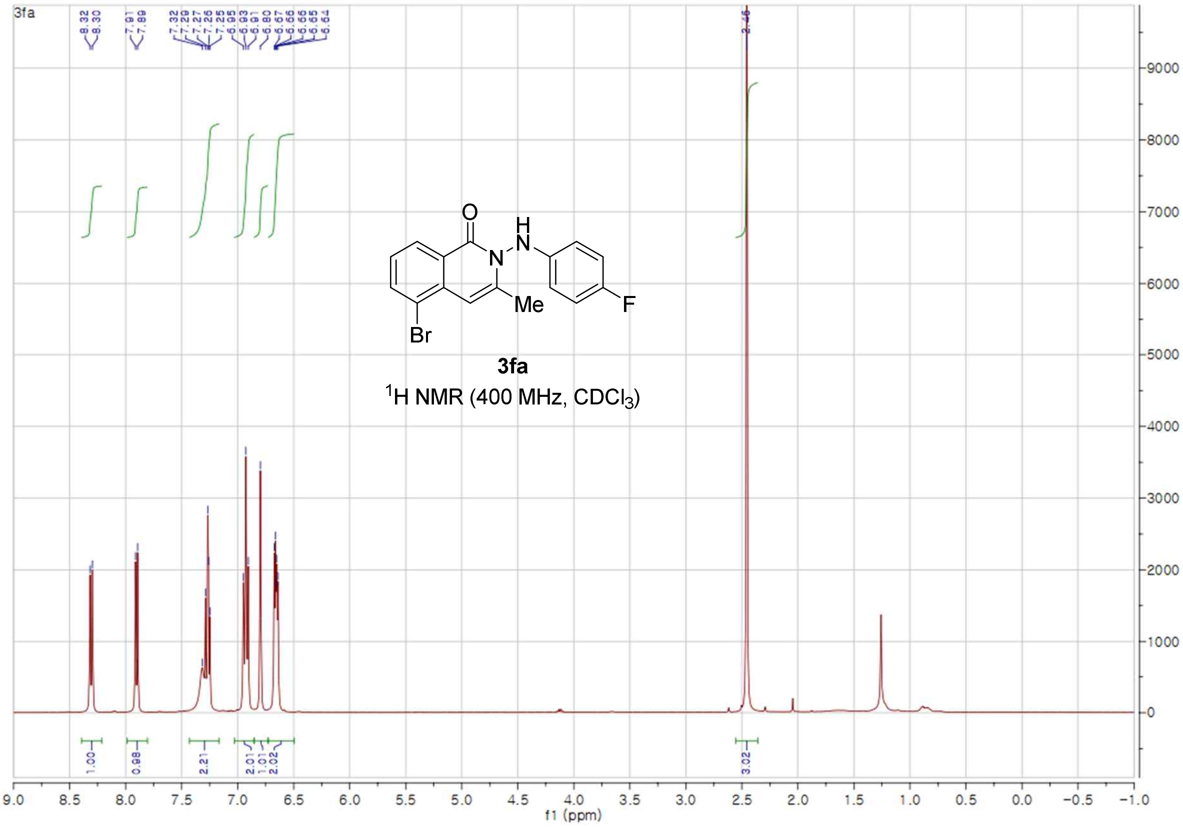
3fa
8.32
8.30
7.91
7.89
7.32
7.29
7.27
7.26
7.25
6.95
6.93
6.91
6.80
6.67
6.66
6.65
6.65
6.64
2.46
O
H
N
N
Me
Br
F
3fa
1H NMR (400 MHz, CDCl3)
1.00
0.98
2.21
2.01
1.01
2.02
3.02
f1 (ppm)

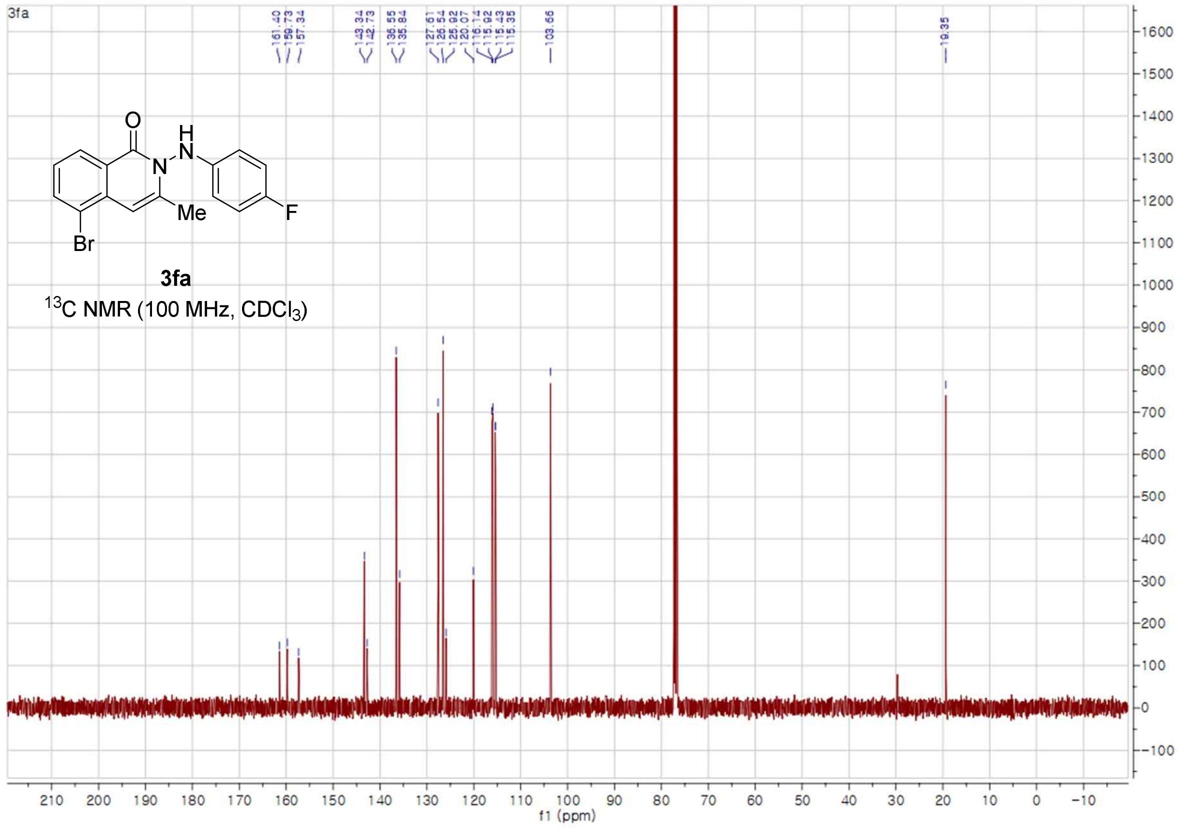
3fa
161.40
159.73
157.34
143.34
142.73
135.55
135.84
127.61
126.54
125.92
120.07
115.92
115.43
115.35
103.66
19.35
O
H
N
N
Me
Br
F
3fa
13C NMR (100 MHz, CDCl3)
f1 (ppm)

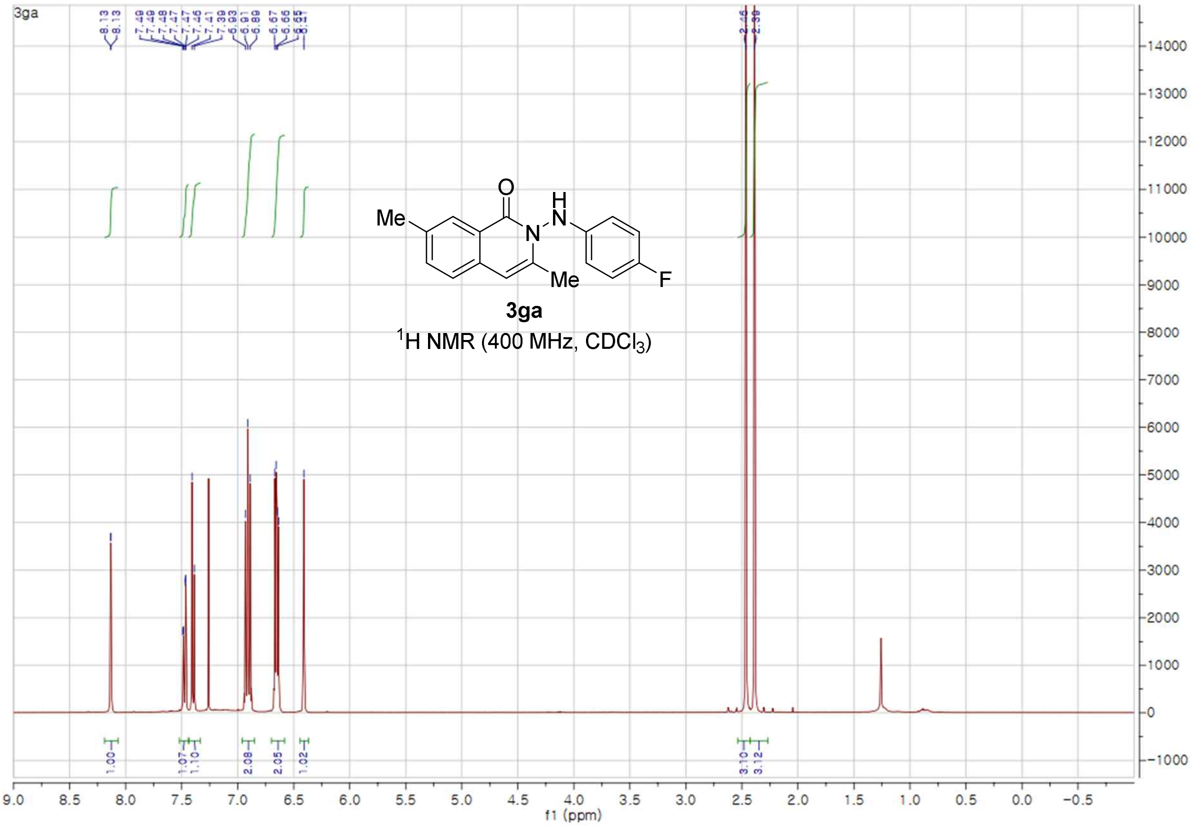
3ga
8.13
8.13
7.49
7.48
7.48
7.47
7.46
7.41
7.39
6.93
6.91
6.89
6.67
6.66
6.65
6.41
2.46
2.39
Me
O
H
N
N
Me
F
3ga
^{1}H NMR (400 MHz, $CDCl_3$)
1.00
1.07
1.10
2.08
2.05
1.02
3.10
3.12
f1 (ppm)

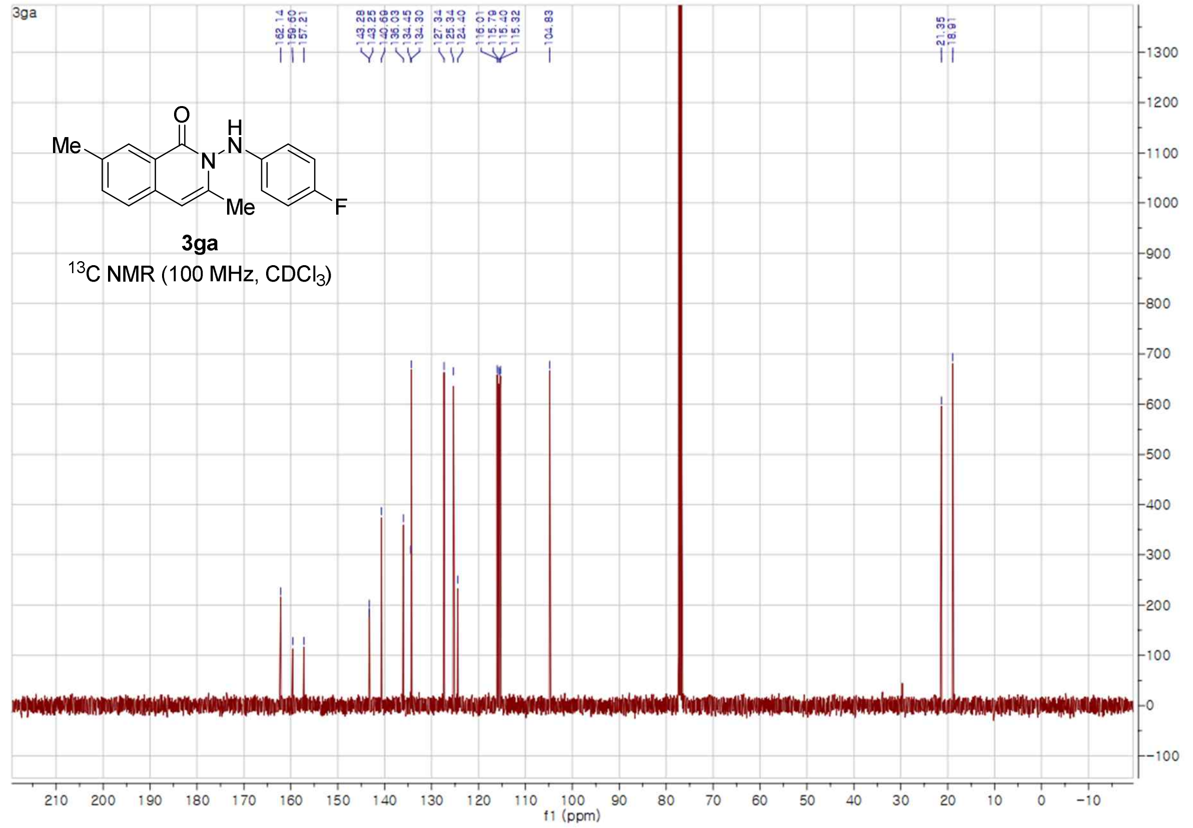
3ga
162.14
159.60
157.21
143.28
143.25
140.69
136.03
134.45
134.30
127.34
125.34
124.40
116.01
115.79
115.40
115.32
104.83
21.35
18.91
Me
O
H
N
N
Me
F
3ga
^{13}C NMR (100 MHz, $CDCl_3$)
f1 (ppm)

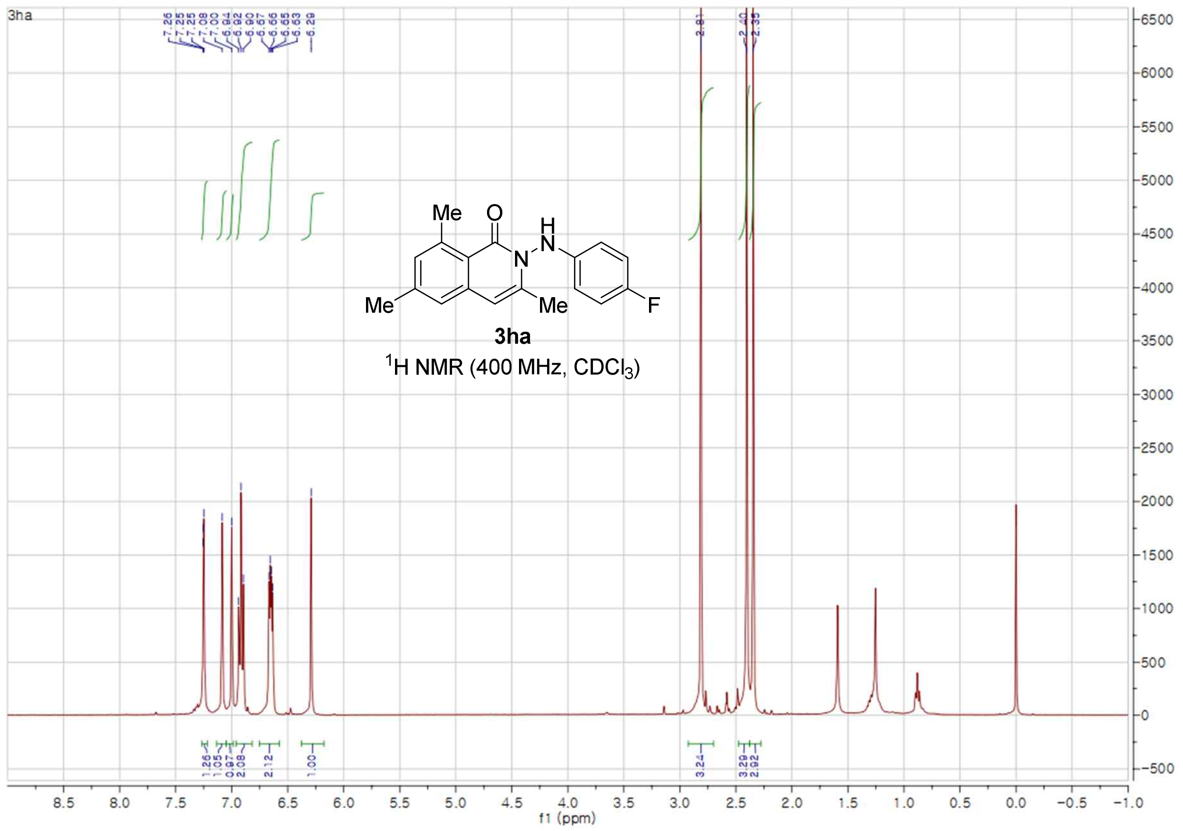
3ha
3ha
¹H NMR (400 MHz, CDCl₃)
f1 (ppm)

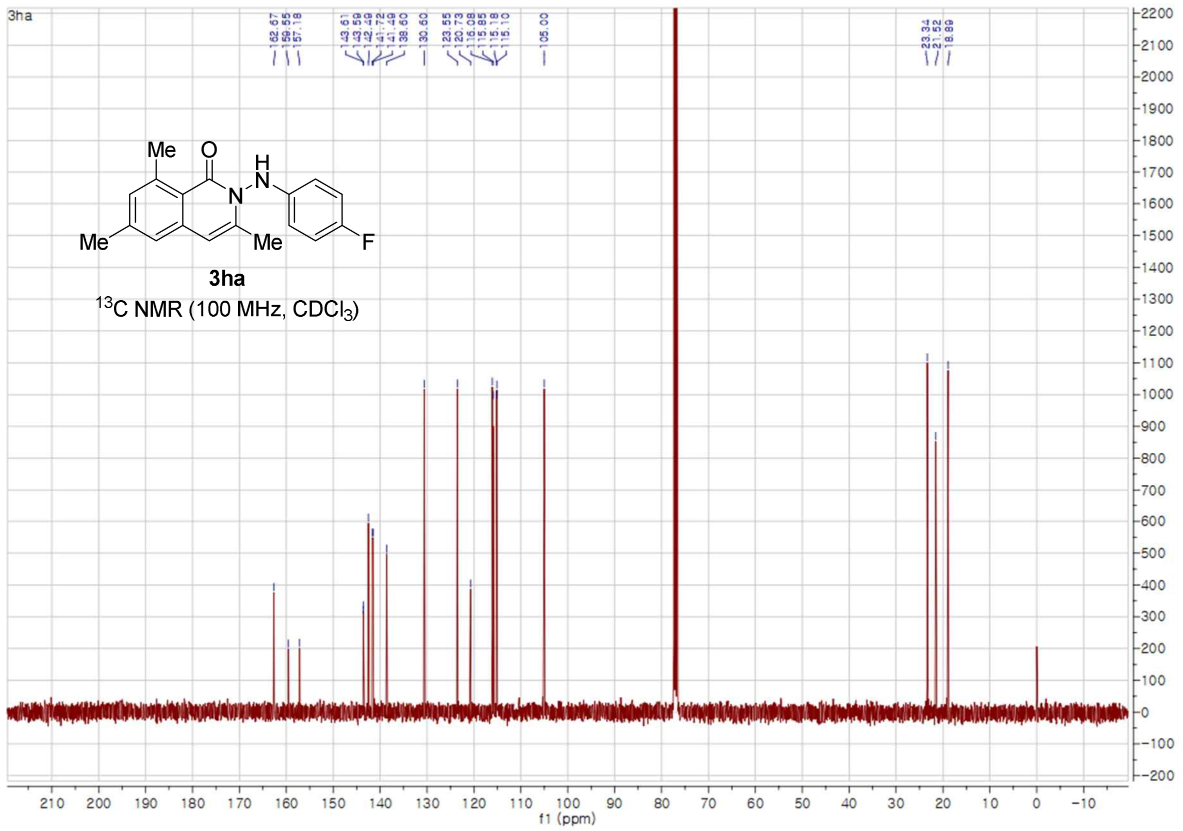
3ha
3ha
¹³C NMR (100 MHz, CDCl₃)
f1 (ppm)

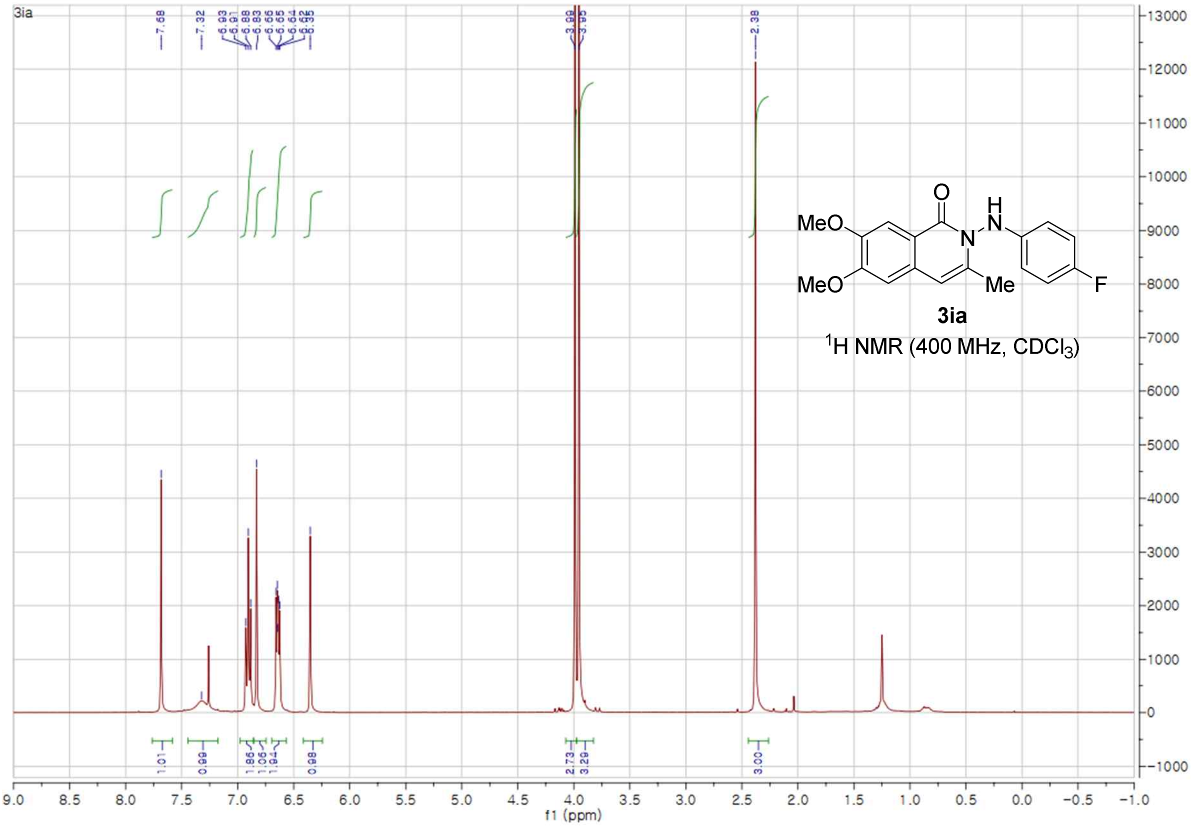
3ia
7.68
7.32
6.93
6.91
6.88
6.83
6.66
6.65
6.64
6.62
6.35
3.99
3.95
2.38
MeO
MeO
O
H
N
N
Me
F
3ia
1H NMR (400 MHz, CDCl3)
1.01
0.99
1.86
1.05
1.94
0.98
2.73
3.29
3.00
f1 (ppm)

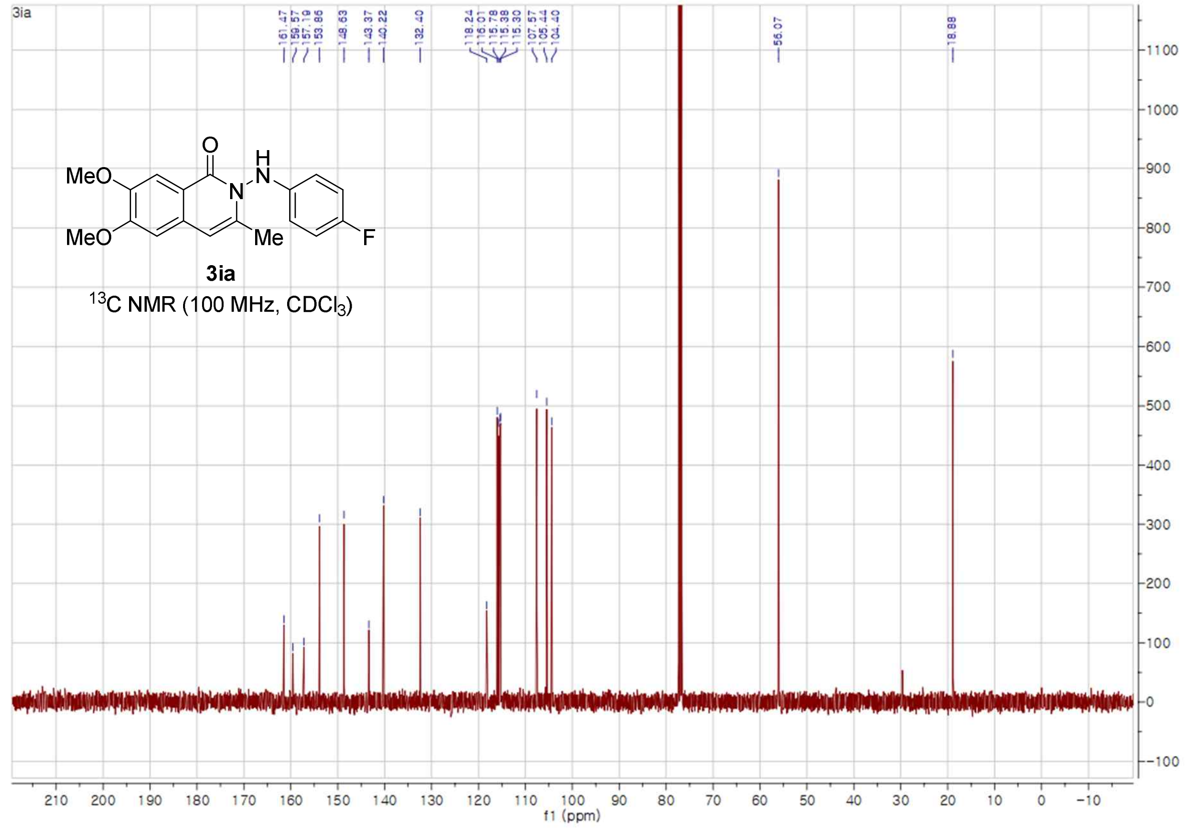
3ia
161.47
159.57
157.19
153.86
148.63
143.37
140.22
132.40
118.24
116.01
115.79
115.38
115.30
107.57
105.44
104.40
56.07
18.88
MeO
MeO
O
H
N
N
Me
F
3ia
13C NMR (100 MHz, CDCl3)
f1 (ppm)

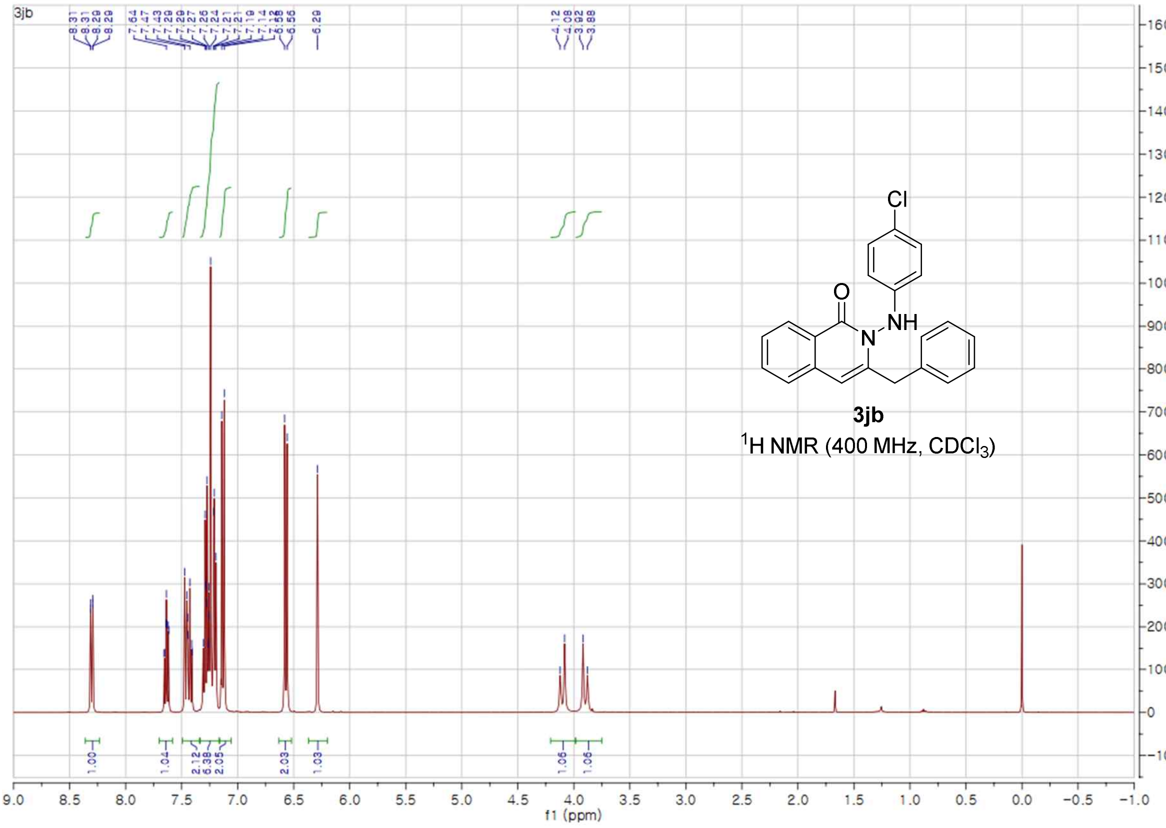
3jb
Cl
O
N
NH
3jb
1H NMR (400 MHz, CDCl3)
f1 (ppm)

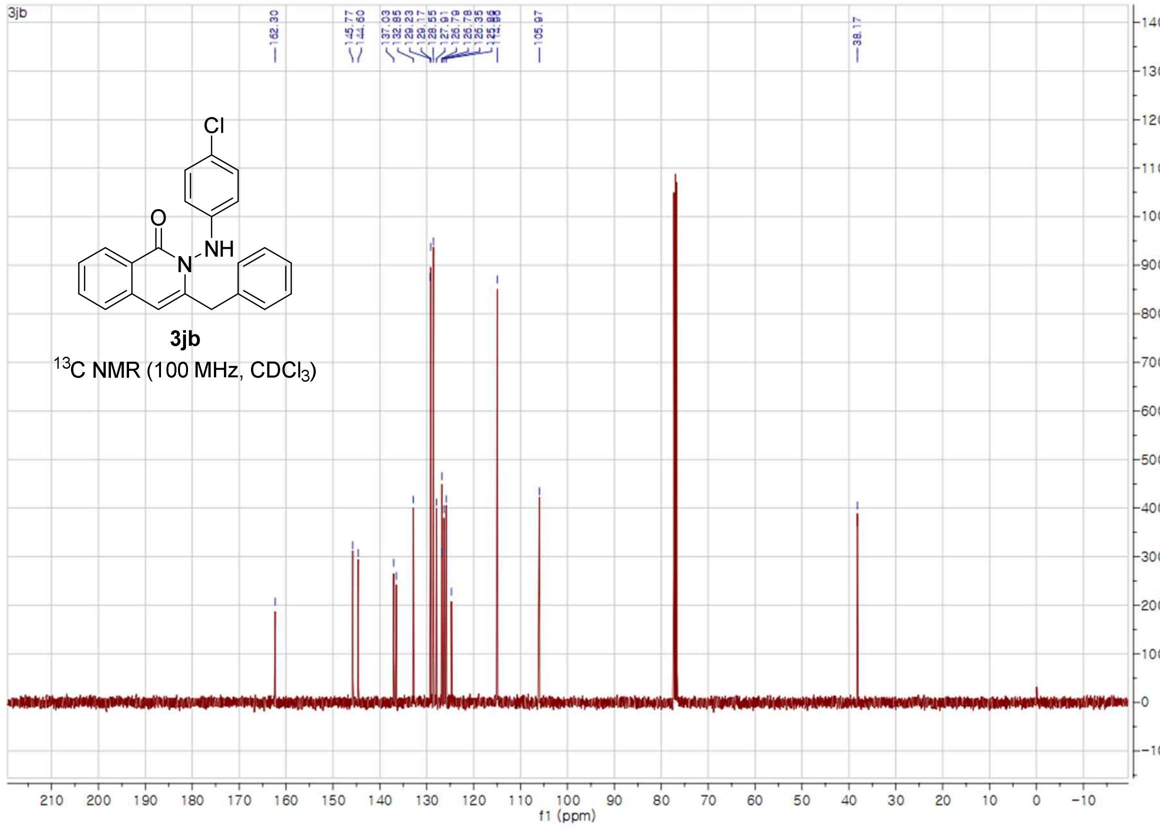
3jb
Cl
O
N
NH
3jb
13C NMR (100 MHz, CDCl3)
f1 (ppm)

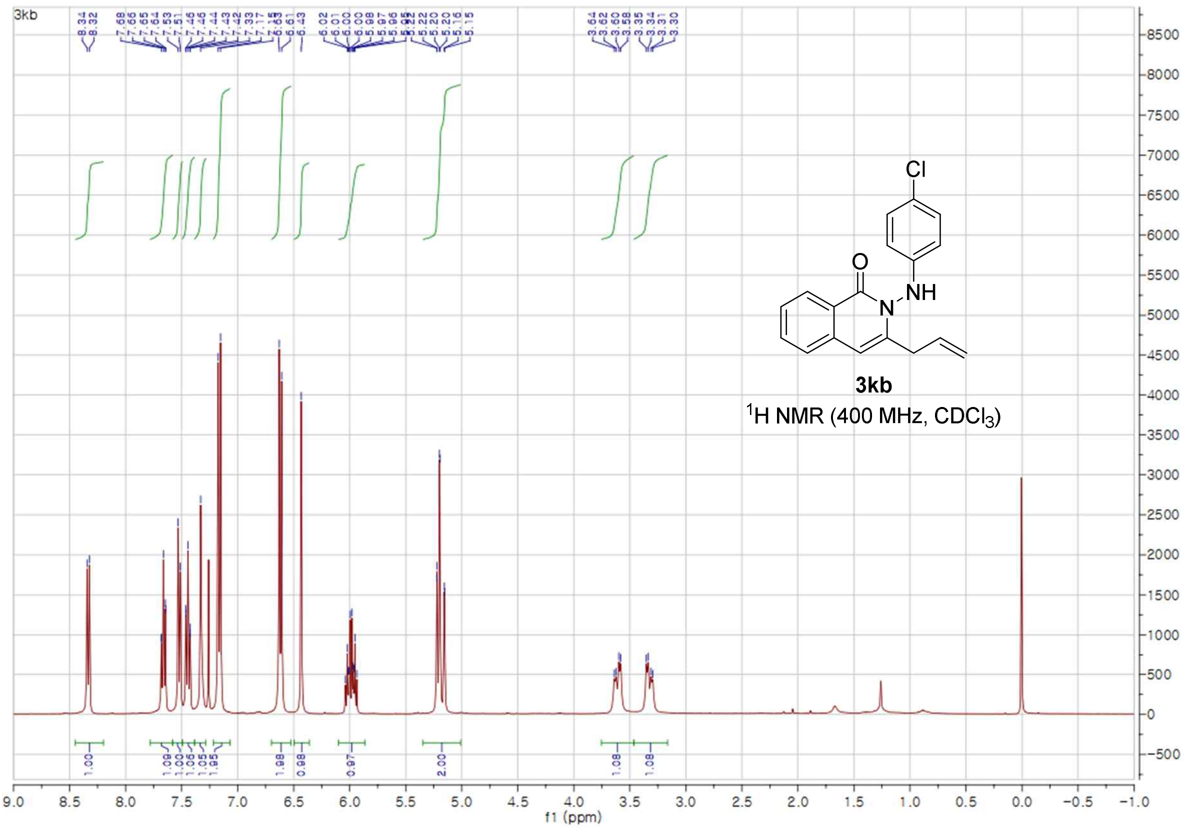
3kb
Cl
O
N
NH
3kb
1H NMR (400 MHz, CDCl3)
f1 (ppm)

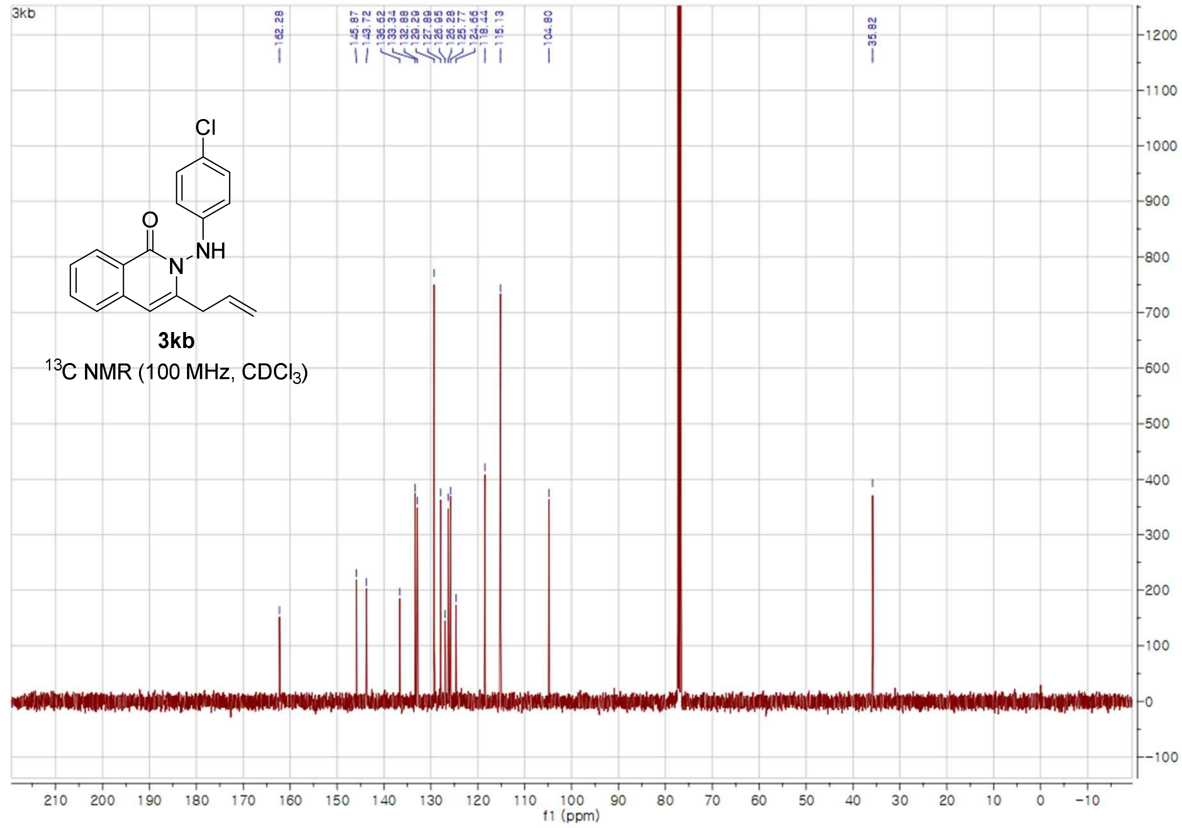
3kb
Cl
O
N
NH
3kb
13C NMR (100 MHz, CDCl3)
f1 (ppm)

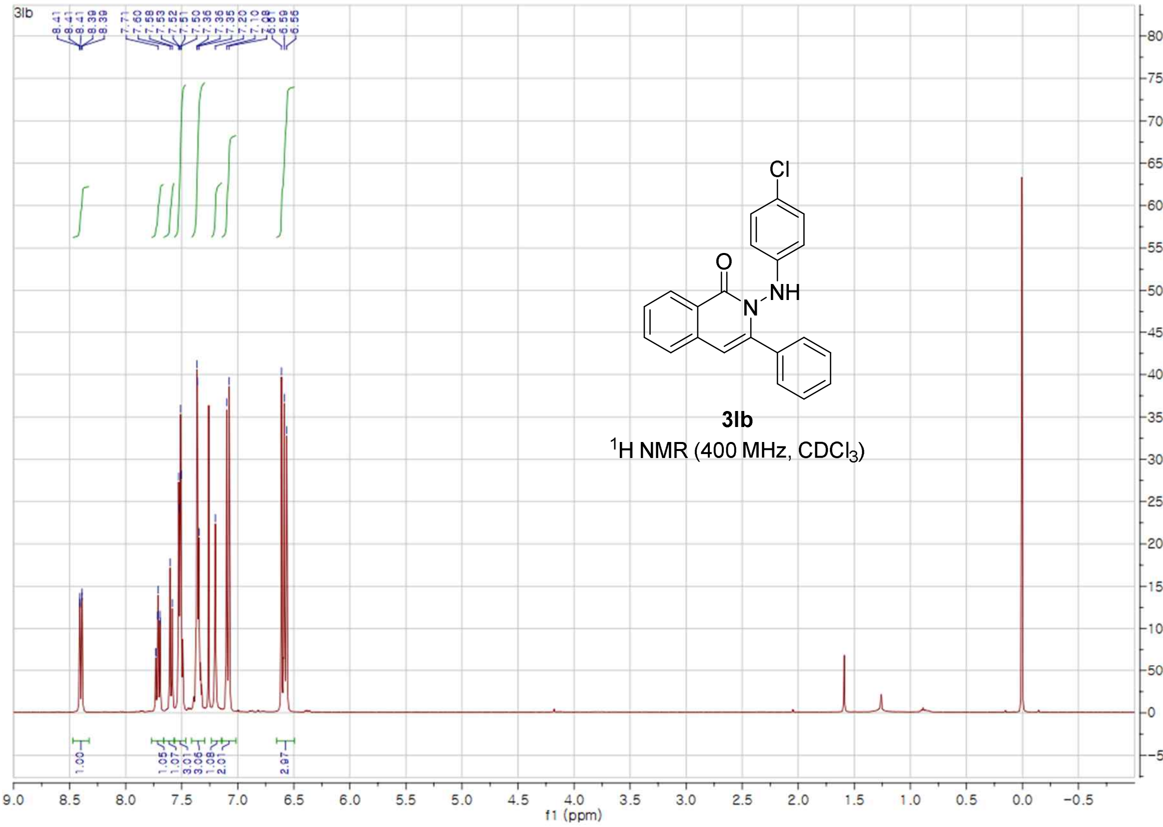
3lb
8.41
8.41
8.39
8.39
7.71
7.60
7.58
7.53
7.52
7.51
7.50
7.36
7.35
7.20
7.10
6.59
6.56
Cl
O
N
NH
3lb
1H NMR (400 MHz, CDCl3)
1.00
1.05
1.07
3.01
3.06
1.08
2.01
2.97
f1 (ppm)

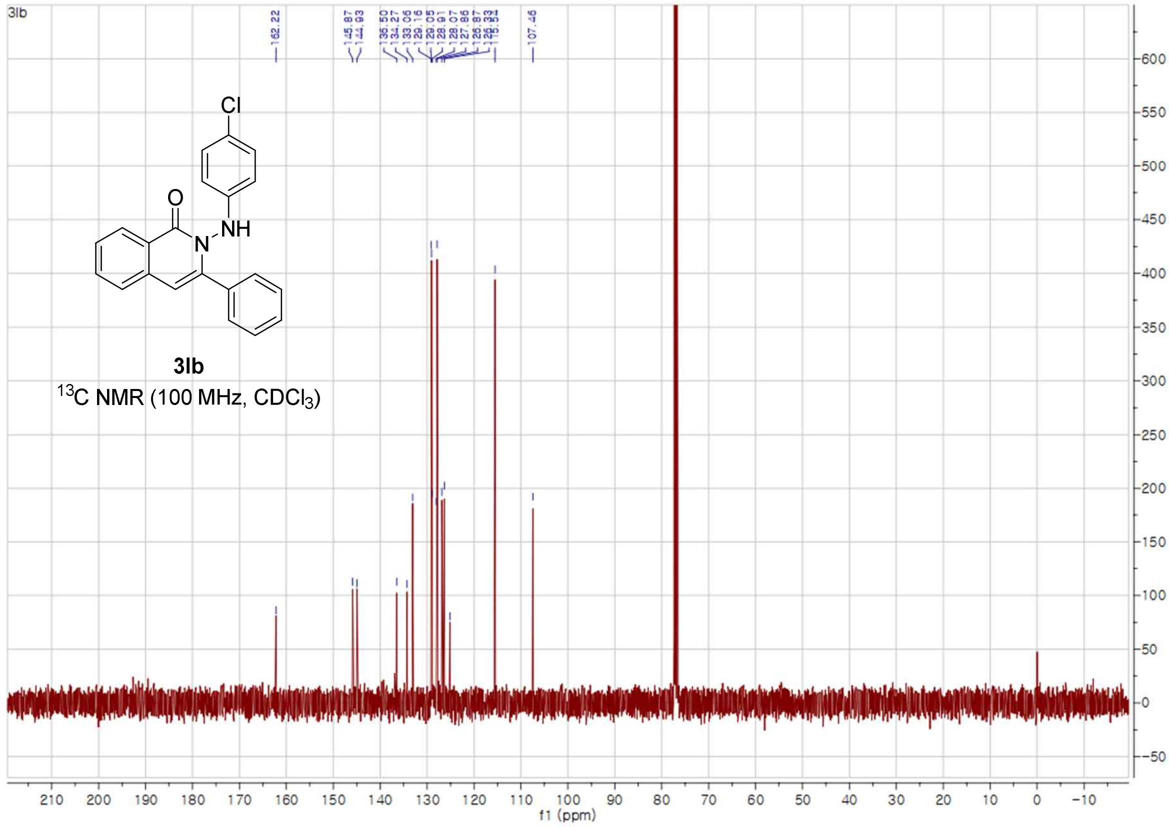
3lb
162.22
145.87
144.93
136.50
134.27
133.06
129.16
129.05
128.91
128.07
127.86
126.87
125.33
115.53
107.46
Cl
O
N
NH
3lb
13C NMR (100 MHz, CDCl3)
f1 (ppm)

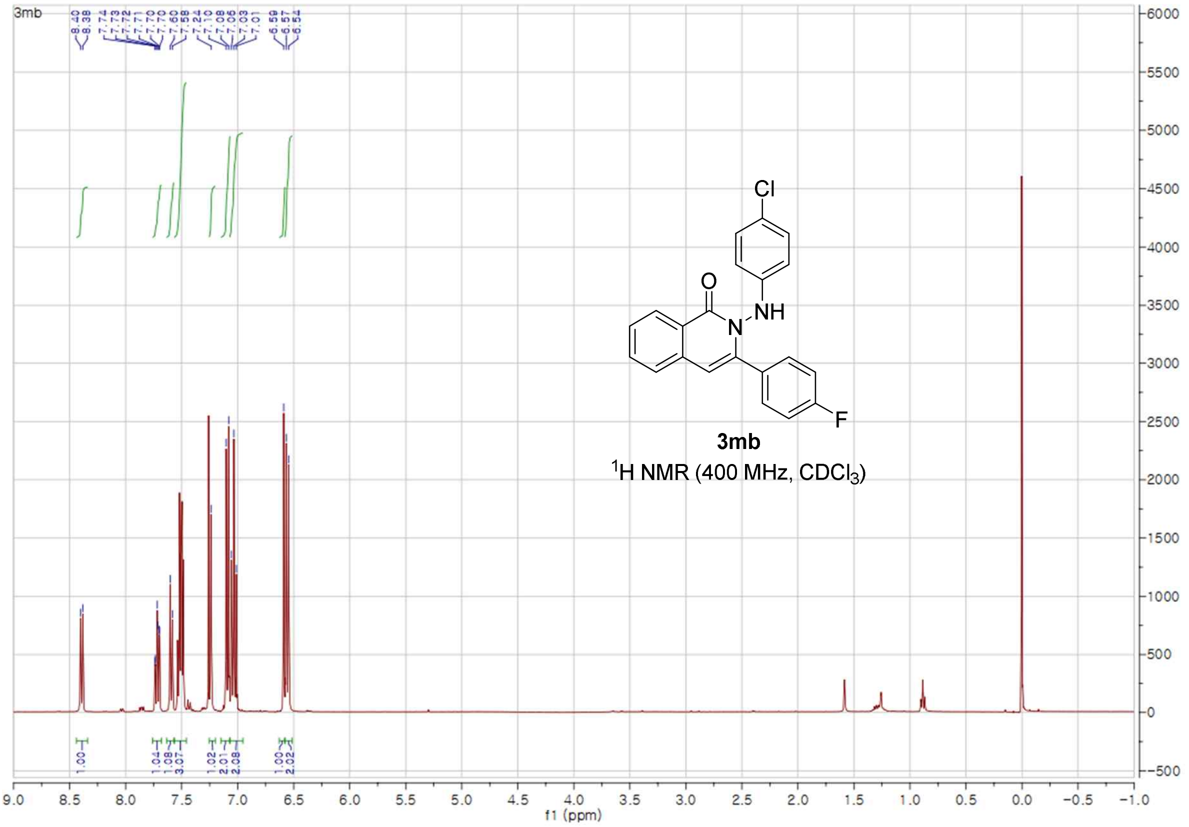

3mb
Cl
O
N
NH
F
3mb
^{1}H NMR (400 MHz, CDCl$_3$)
f1 (ppm)

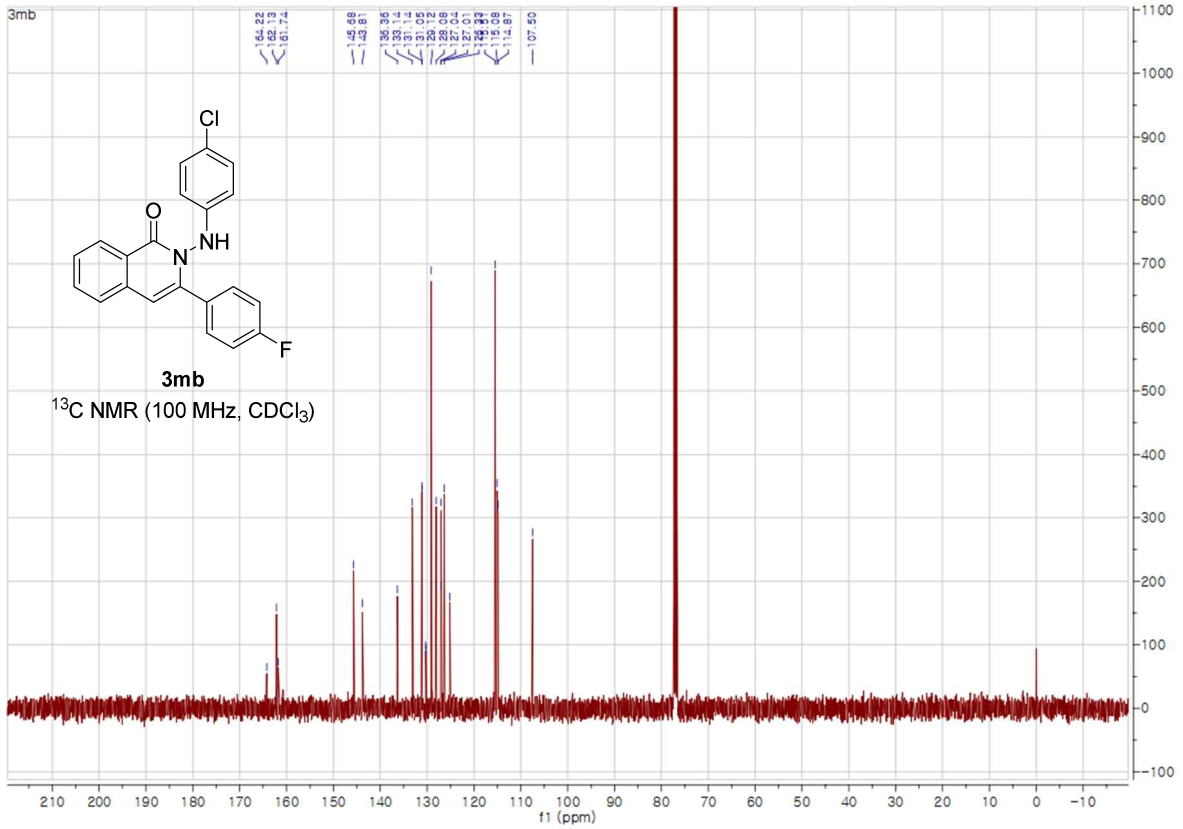

3mb
Cl
O
N
NH
F
3mb
^{13}C NMR (100 MHz, CDCl$_3$)
f1 (ppm)

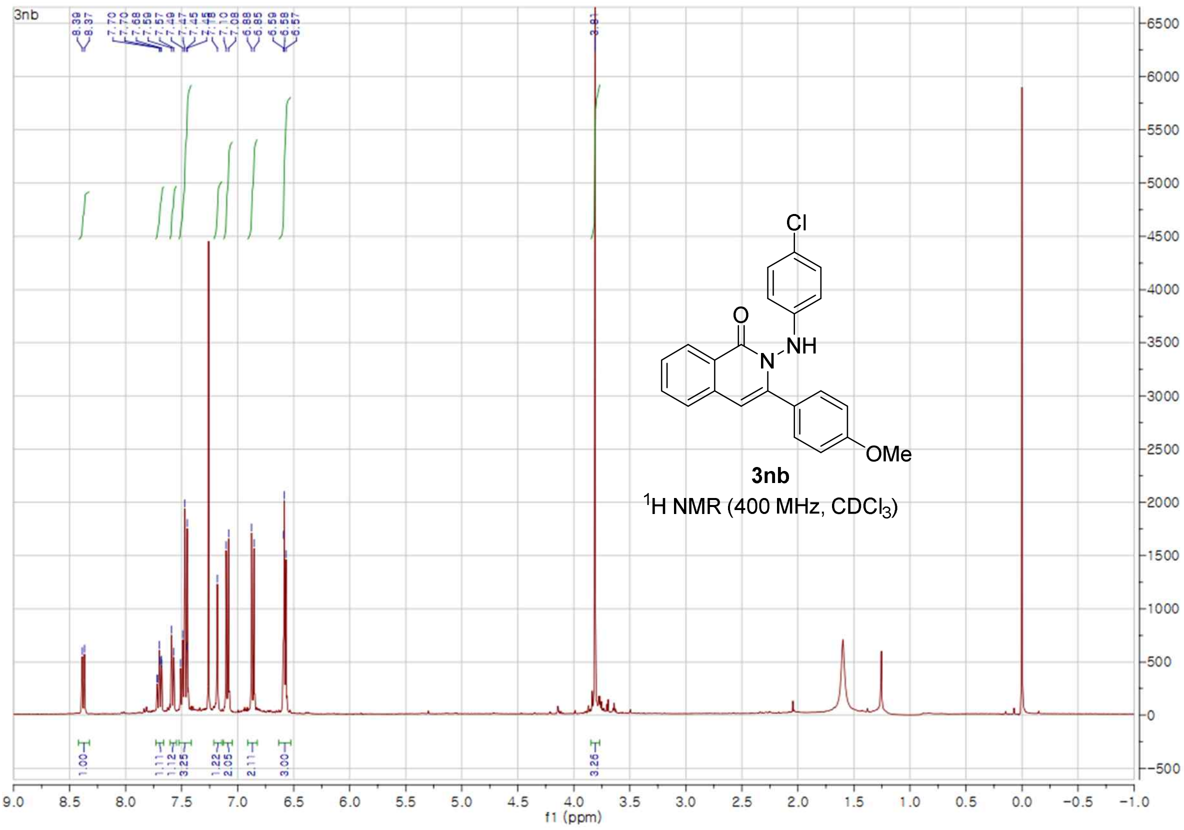

3nb
8.39
8.37
7.70
7.70
7.68
7.59
7.57
7.57
7.49
7.47
7.45
7.18
7.10
7.08
6.88
6.85
6.59
6.58
6.57
3.81
3nb
^{1}H NMR (400 MHz, $CDCl_3$)
1.00
1.11
1.12
3.25
1.22
2.05
2.11
3.00
3.26
f1 (ppm)

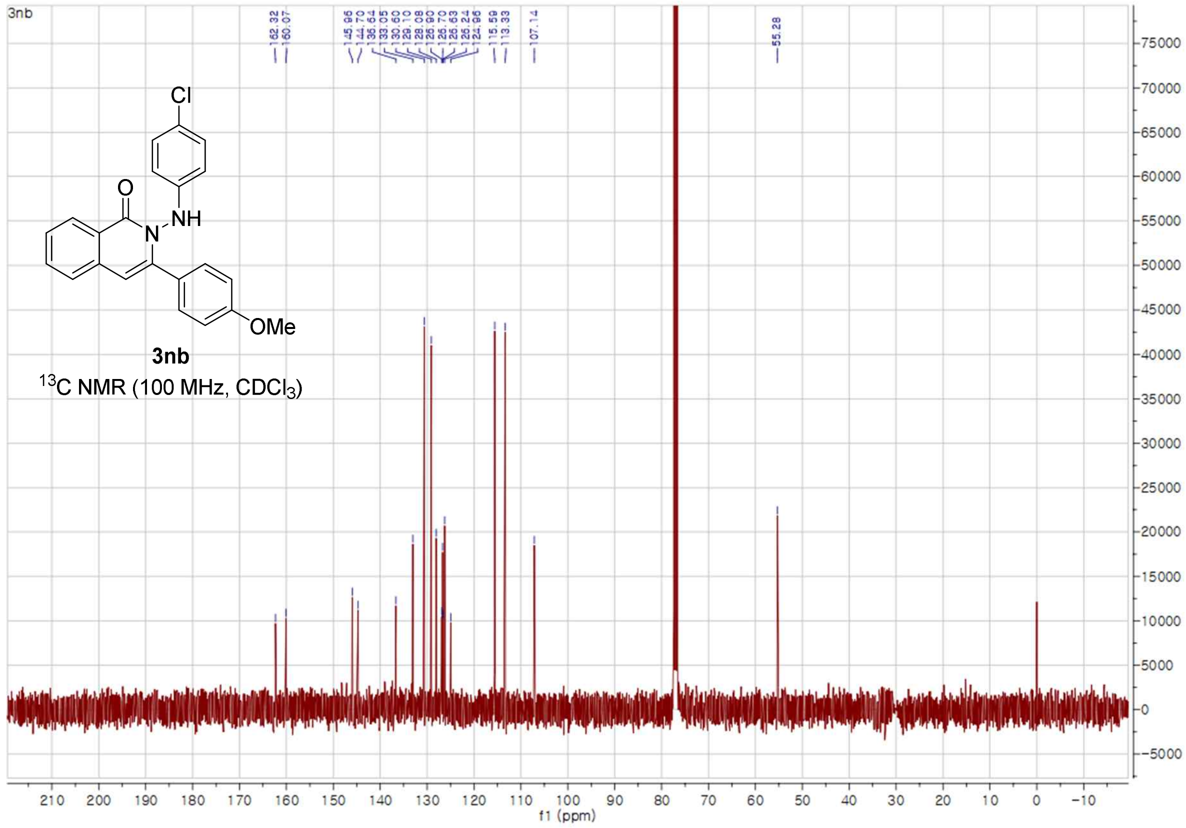

3nb
162.32
160.07
145.96
144.70
136.64
133.05
130.60
129.10
128.08
126.90
126.70
126.63
126.24
124.96
115.59
113.33
107.14
55.28
3nb
^{13}C NMR (100 MHz, $CDCl_3$)
f1 (ppm)

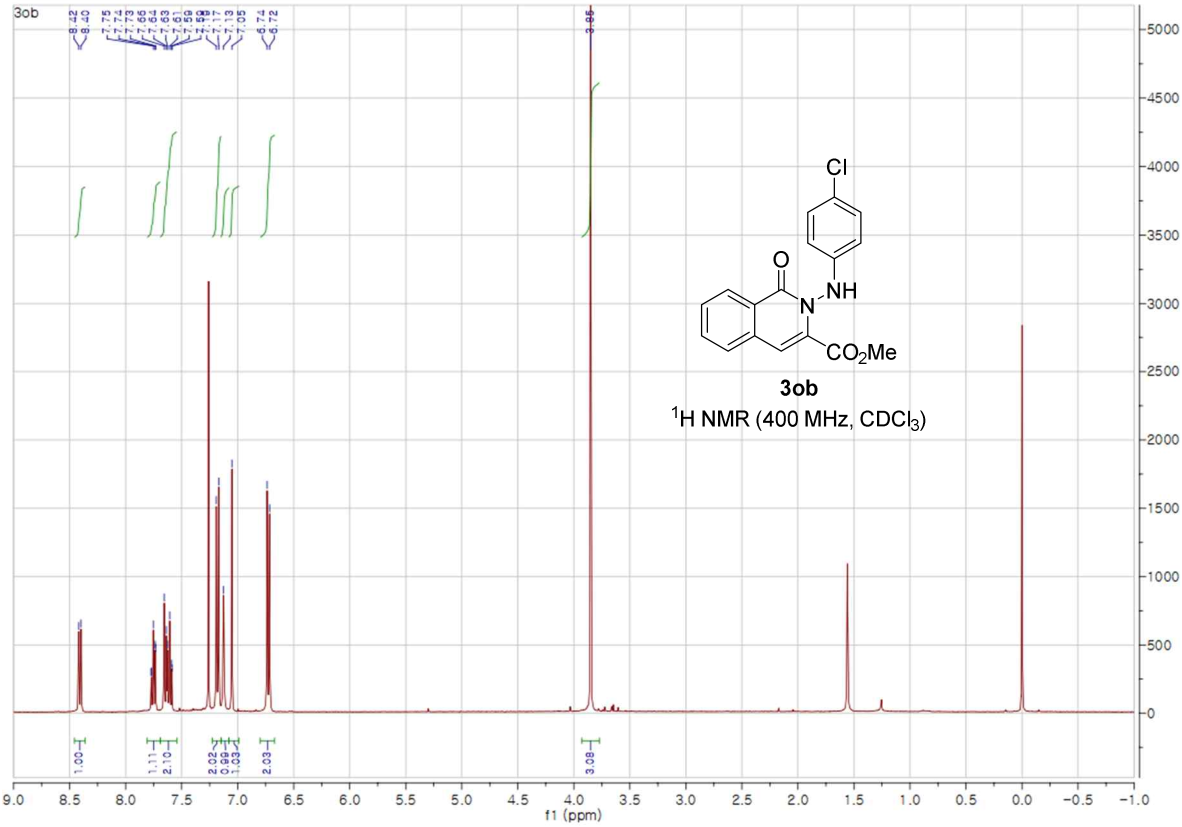
3ob
8.42
8.40
7.75
7.74
7.73
7.66
7.64
7.63
7.61
7.59
7.17
7.13
7.05
6.74
6.72
3.85
Cl
O
N
NH
CO2Me
3ob
1H NMR (400 MHz, CDCl3)
1.00
1.11
2.10
2.02
0.99
1.03
2.03
3.08
f1 (ppm)

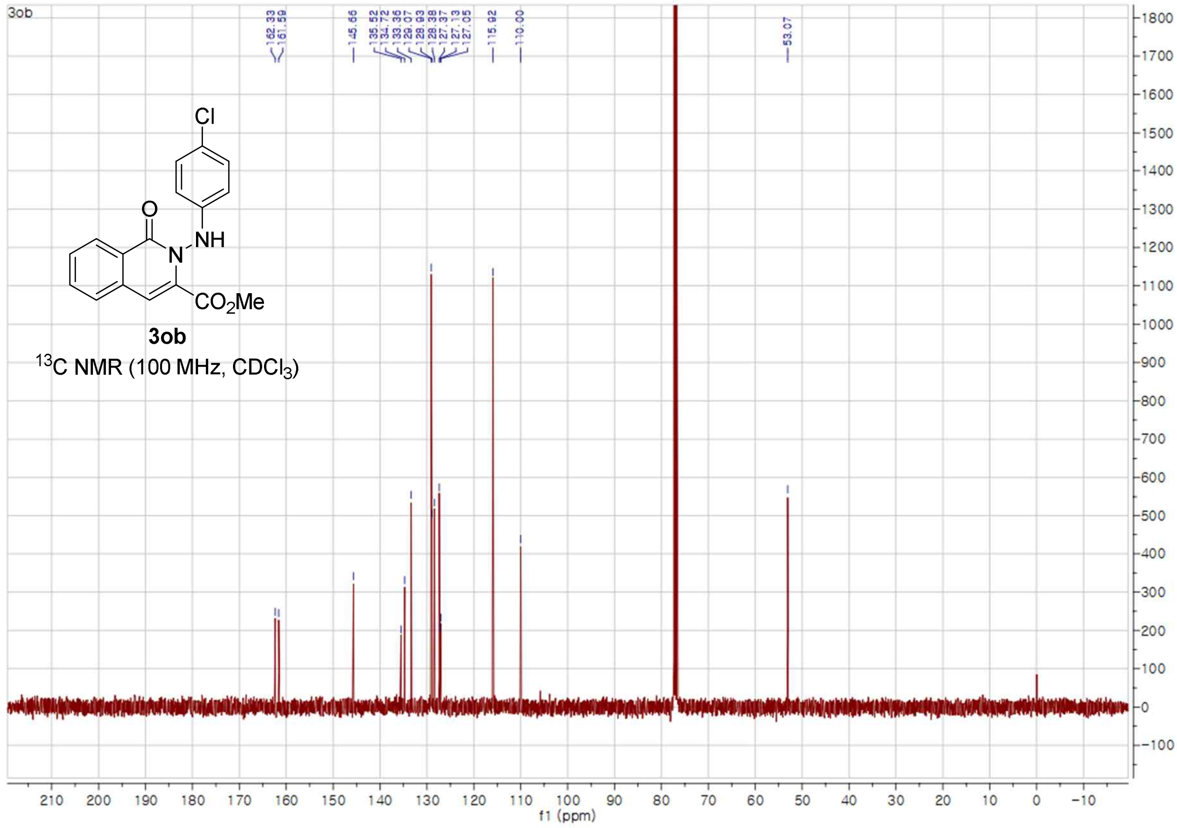
3ob
162.33
161.59
145.66
135.52
134.72
133.35
129.07
128.93
128.38
127.37
127.13
127.05
115.92
110.00
53.07
Cl
O
N
NH
CO2Me
3ob
13C NMR (100 MHz, CDCl3)
f1 (ppm)

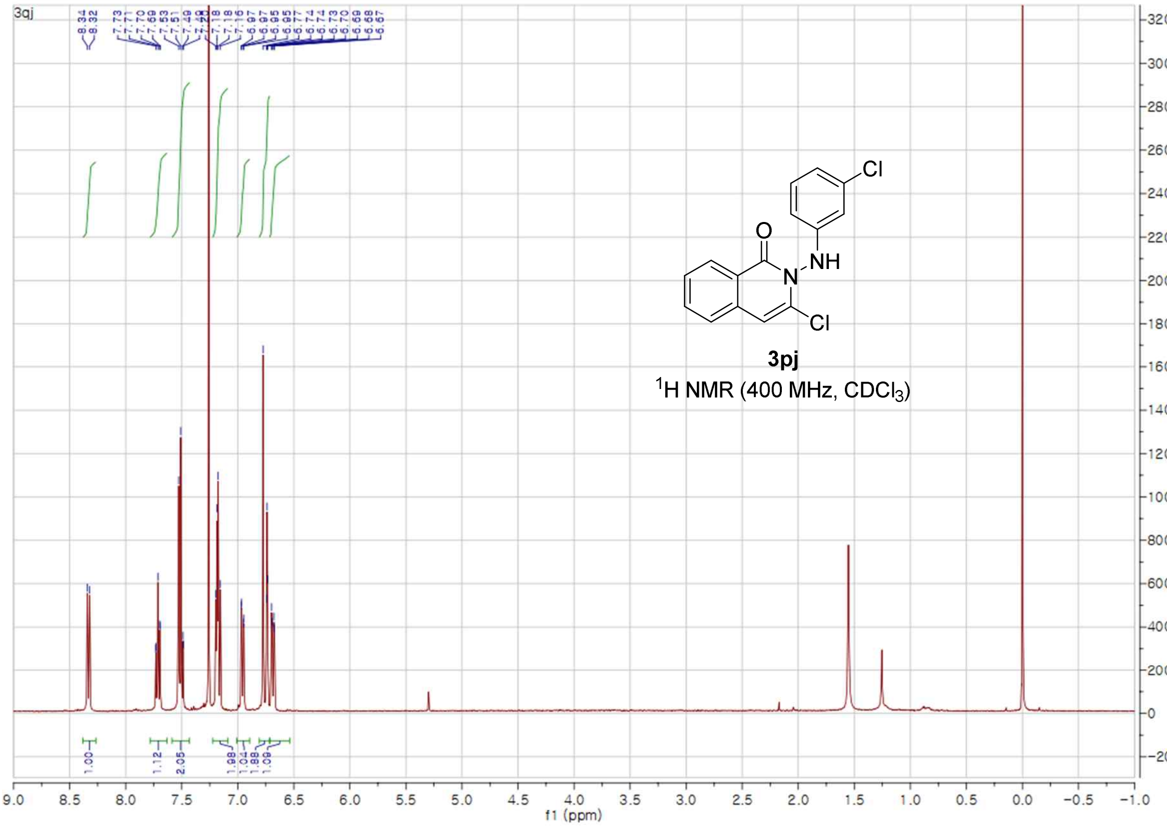
3qj
O
N
NH
Cl
Cl
3pj
^{1}H NMR (400 MHz, $CDCl_3$)
f1 (ppm)

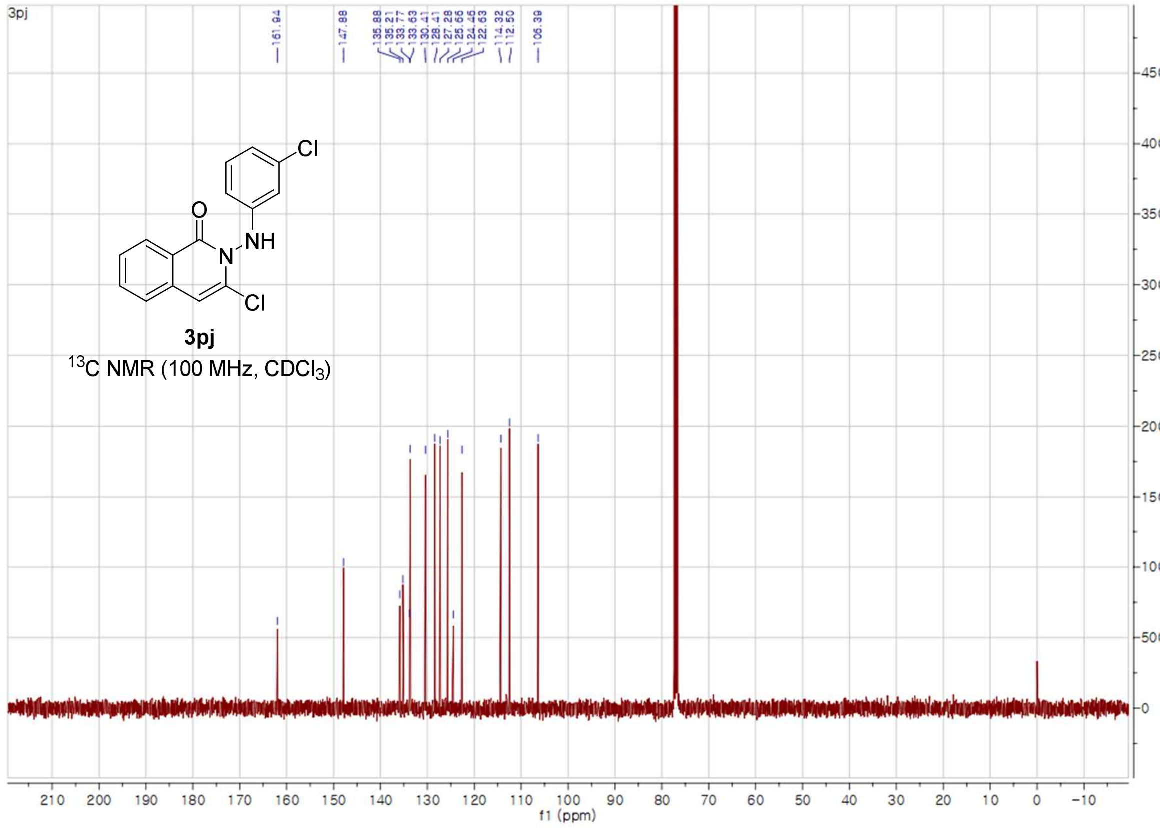
3pj
161.94
147.88
O
N
NH
Cl
Cl
3pj
^{13}C NMR (100 MHz, $CDCl_3$)
f1 (ppm)

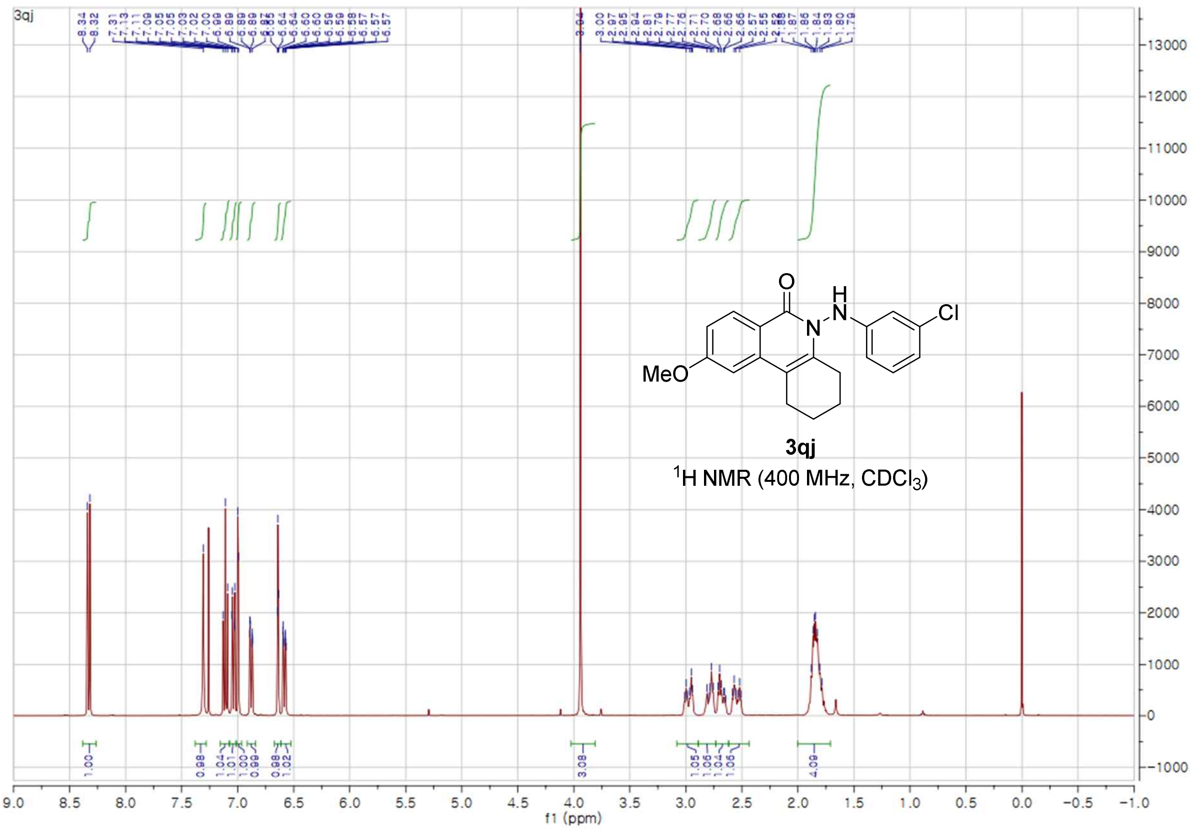
3qj
O
H
N
N
Cl
MeO
3qj
^{1}H NMR (400 MHz, CDCl$_3$)
f1 (ppm)

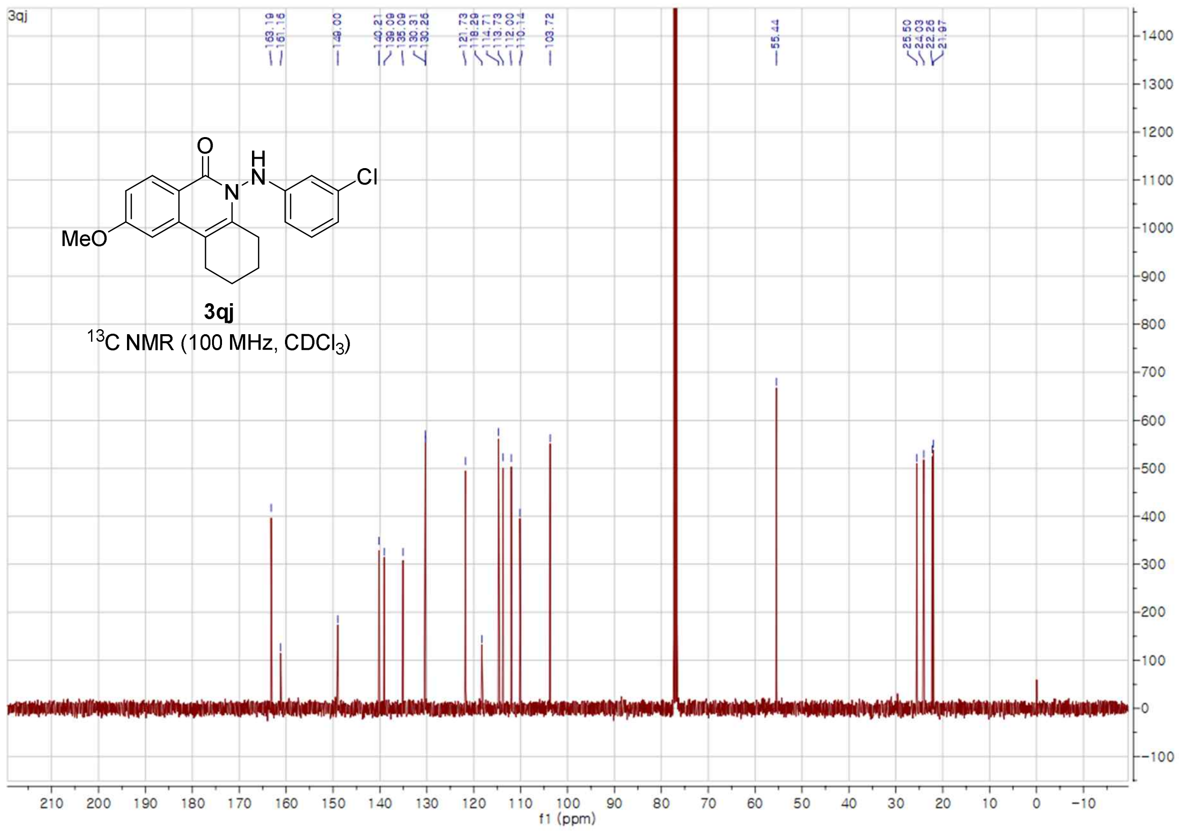
3qj
O
H
N
N
Cl
MeO
3qj
^{13}C NMR (100 MHz, CDCl$_3$)
f1 (ppm)

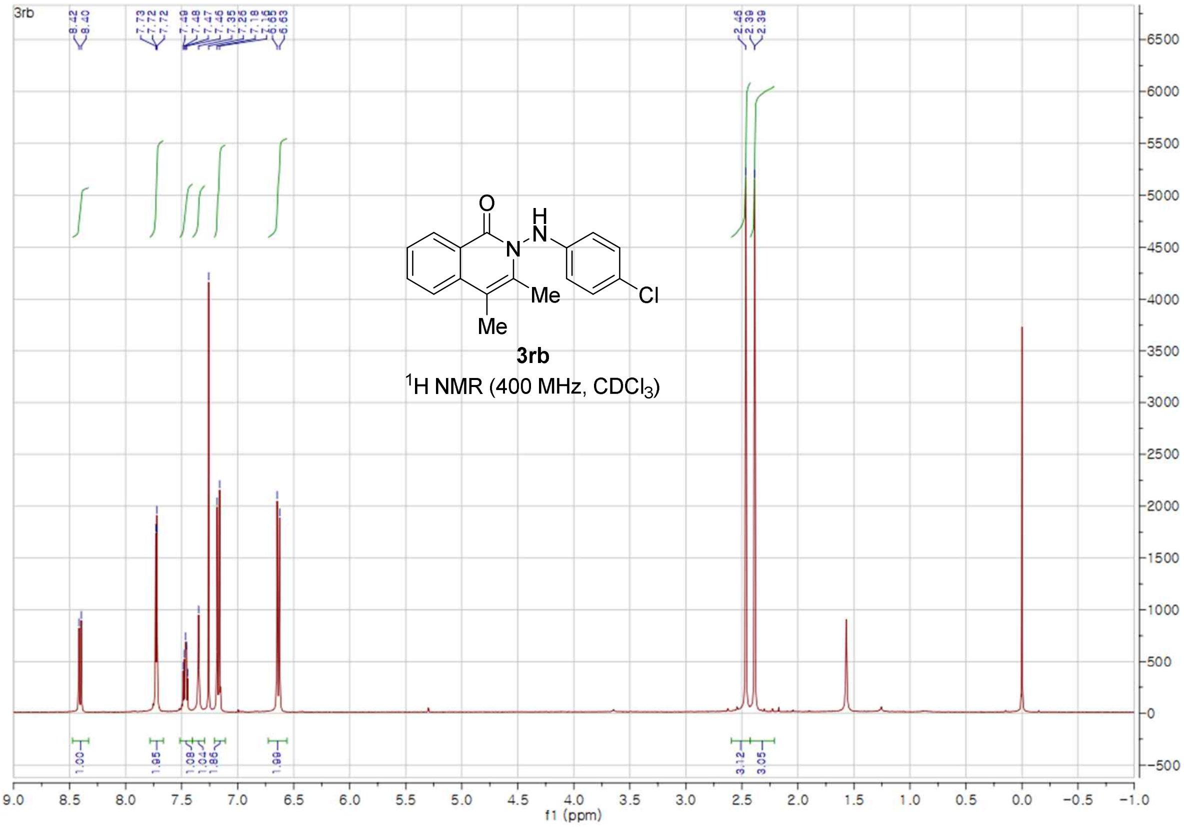
3rb
O
H
N
N
Me
Cl
Me
3rb
1H NMR (400 MHz, CDCl3)
f1 (ppm)

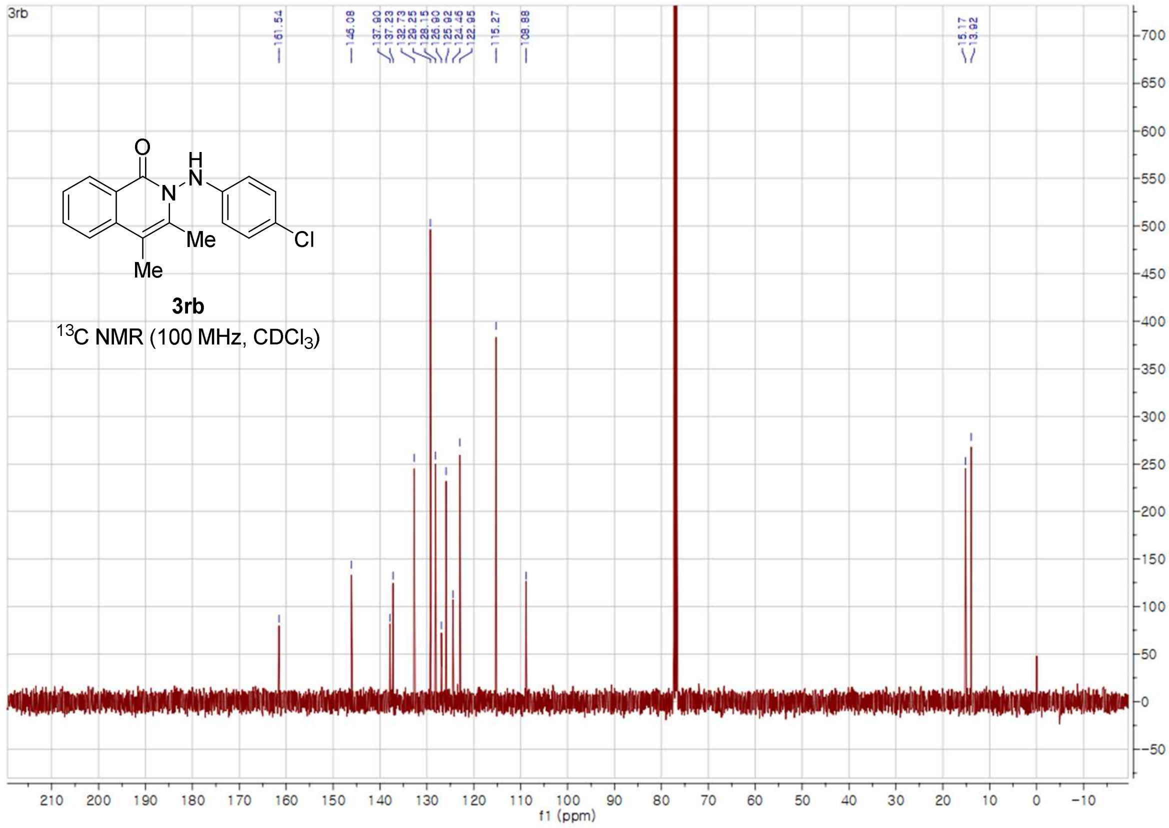
3rb
161.54
145.08
115.27
108.88
15.17
13.92
O
H
N
N
Me
Cl
Me
3rb
13C NMR (100 MHz, CDCl3)
f1 (ppm)

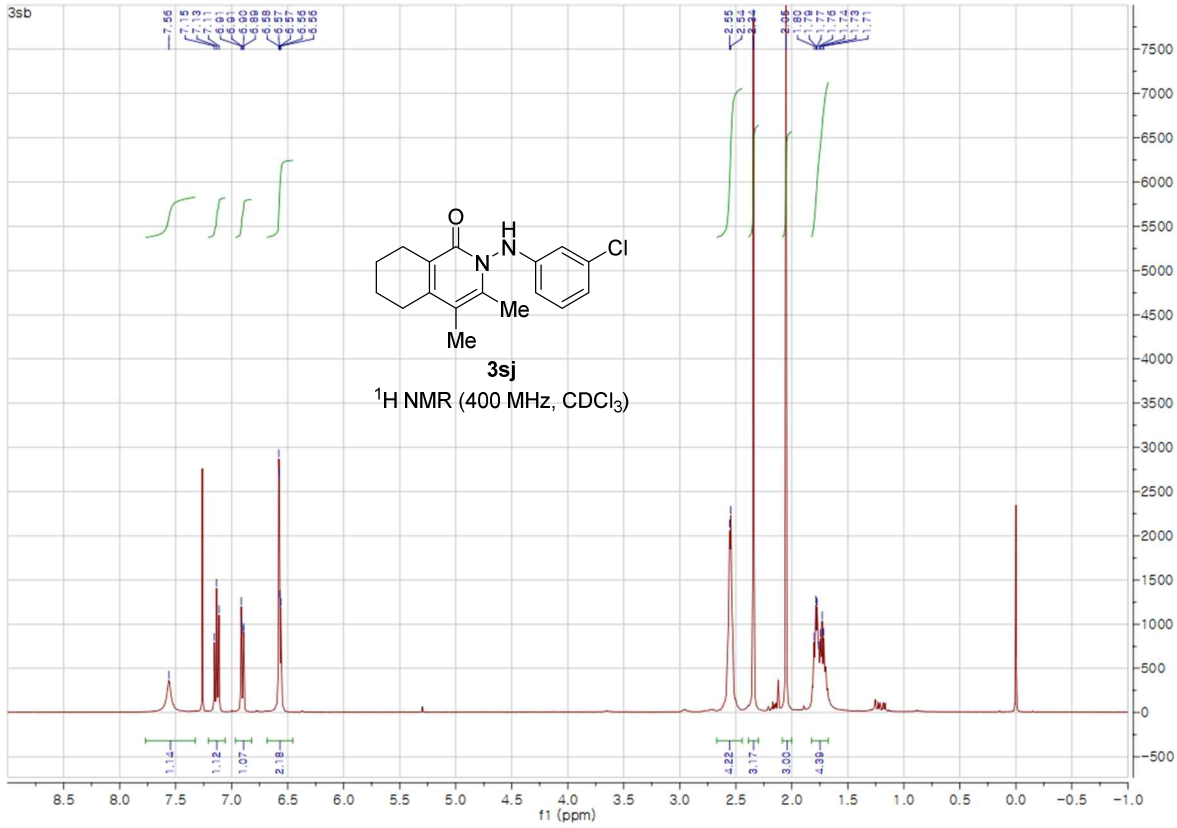
3sb
7.56
7.15
7.13
7.11
6.91
6.91
6.90
6.89
6.58
6.57
6.57
6.56
6.56
2.55
2.54
2.34
2.05
1.80
1.79
1.77
1.76
1.74
1.73
1.71
O
H
N
N
Cl
Me
Me
3sj
1.14
1.12
1.07
2.18
4.22
3.17
3.00
4.39
f1 (ppm)

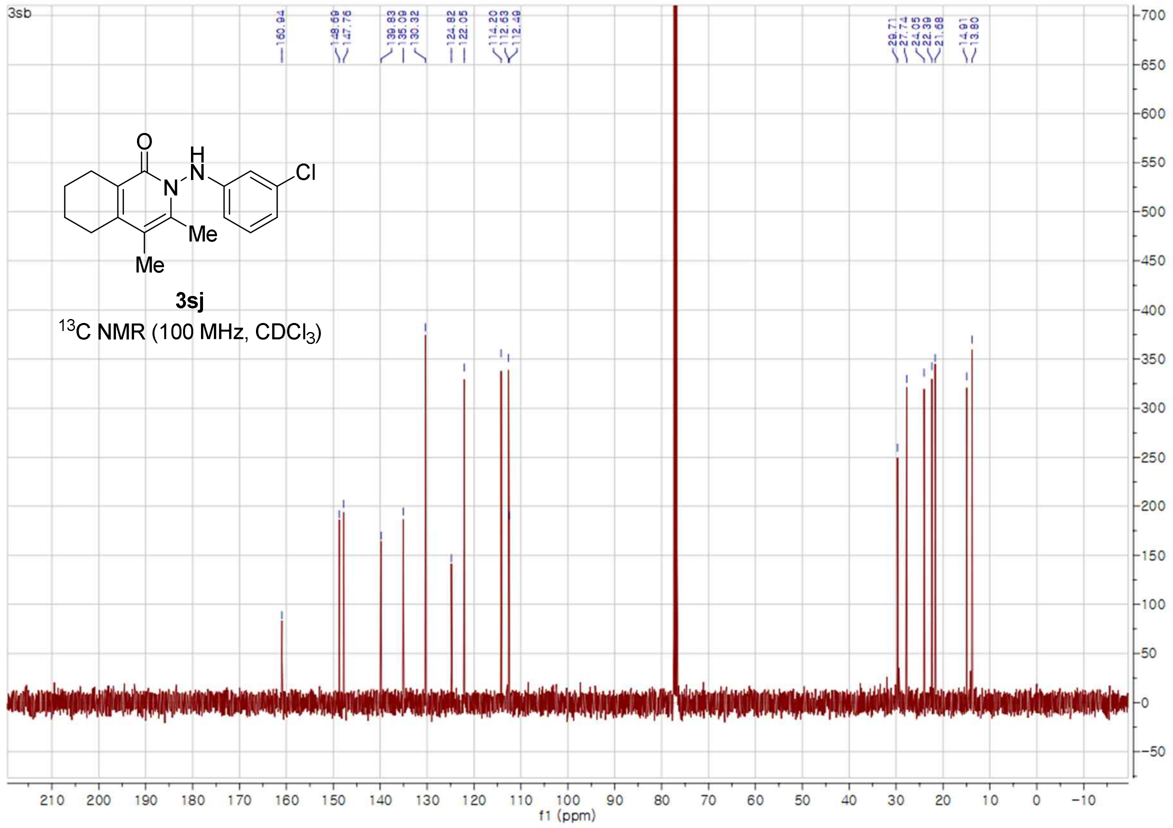
3sb
160.94
148.69
147.76
139.83
135.09
130.32
124.82
122.05
114.20
112.53
112.48
29.71
27.74
24.05
22.39
21.58
14.91
13.80
O
H
N
N
Cl
Me
Me
3sj
f1 (ppm)

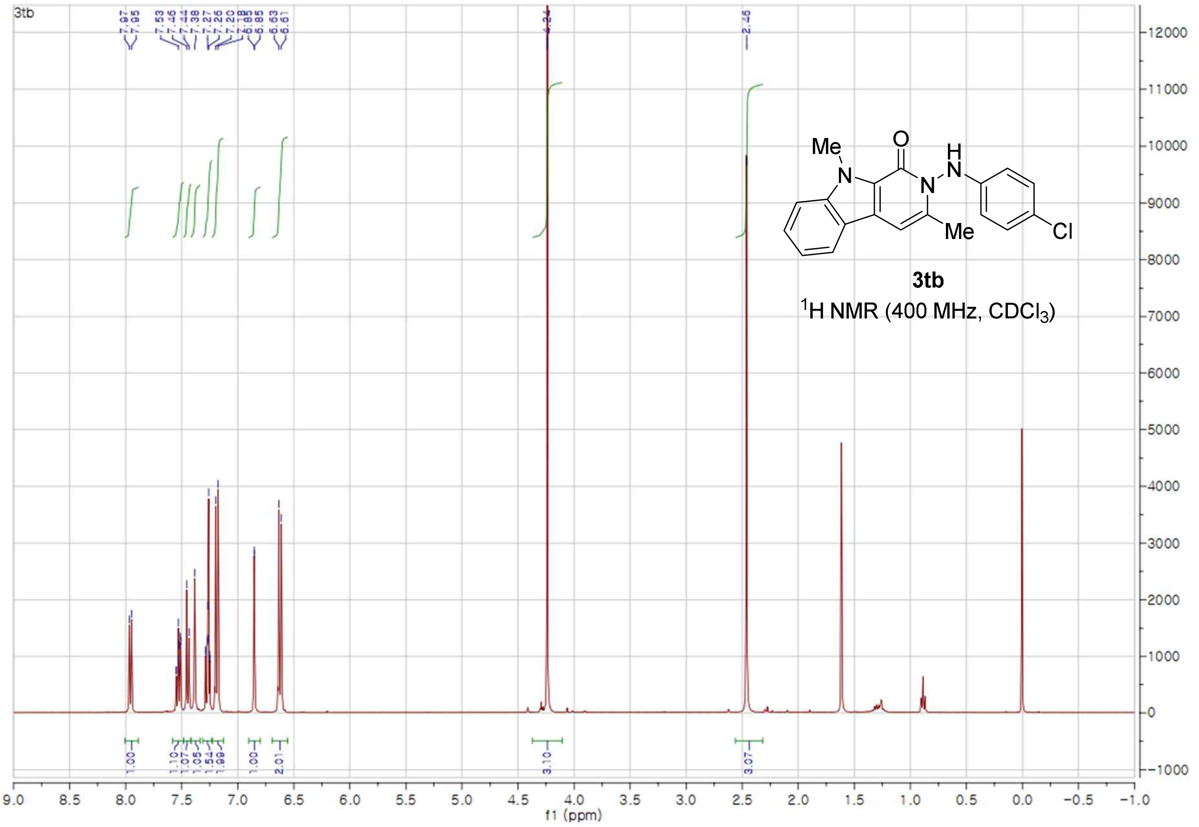
3tb
7.97
7.95
7.53
7.45
7.44
7.38
7.27
7.26
7.20
7.18
6.85
6.85
6.63
6.61
4.24
2.46
Me
N
O
H
N
N
Me
Cl
3tb
^{1}H NMR (400 MHz, CDCl$_3$)
1.00
1.10
1.07
1.05
1.04
1.98
1.00
2.01
3.10
3.07
f1 (ppm)

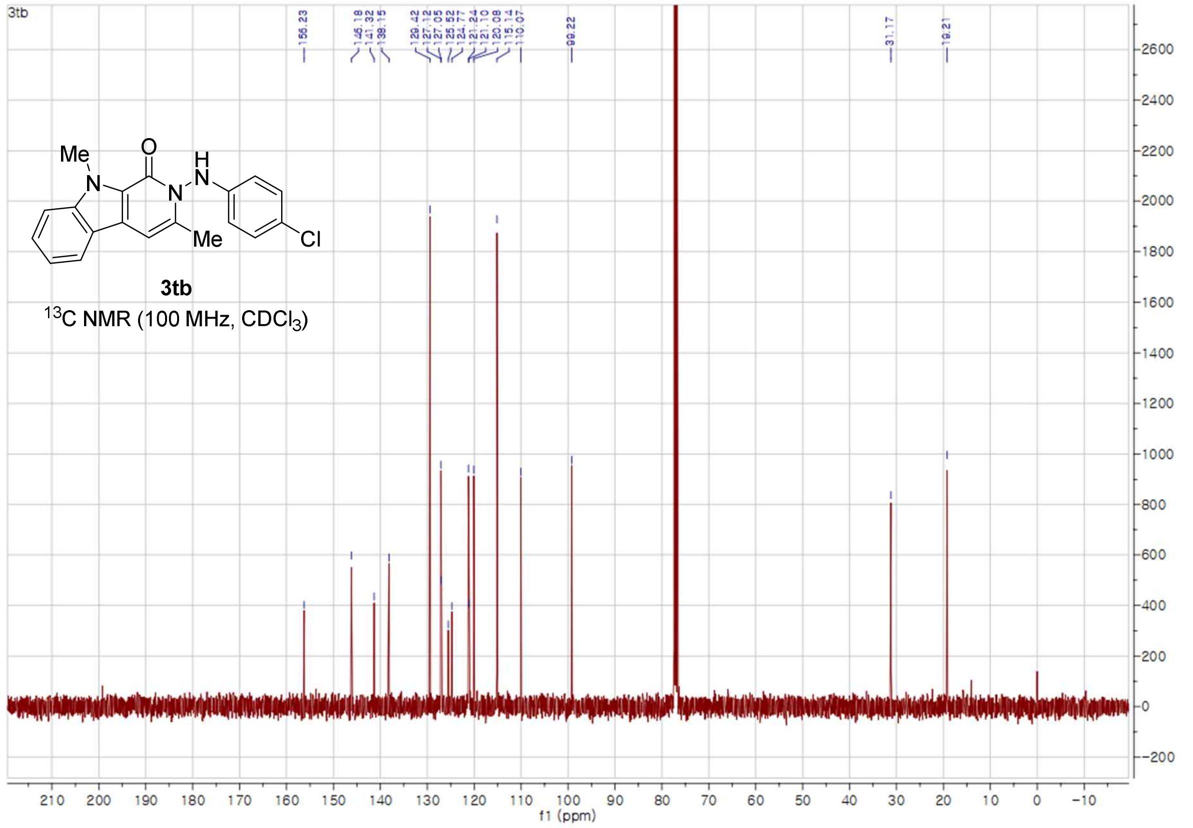
3tb
156.23
145.18
141.32
138.15
129.42
127.12
127.05
125.52
124.77
121.24
121.10
120.08
115.14
110.07
99.22
31.17
19.21
Me
N
O
H
N
N
Me
Cl
3tb
^{13}C NMR (100 MHz, CDCl$_3$)
f1 (ppm)

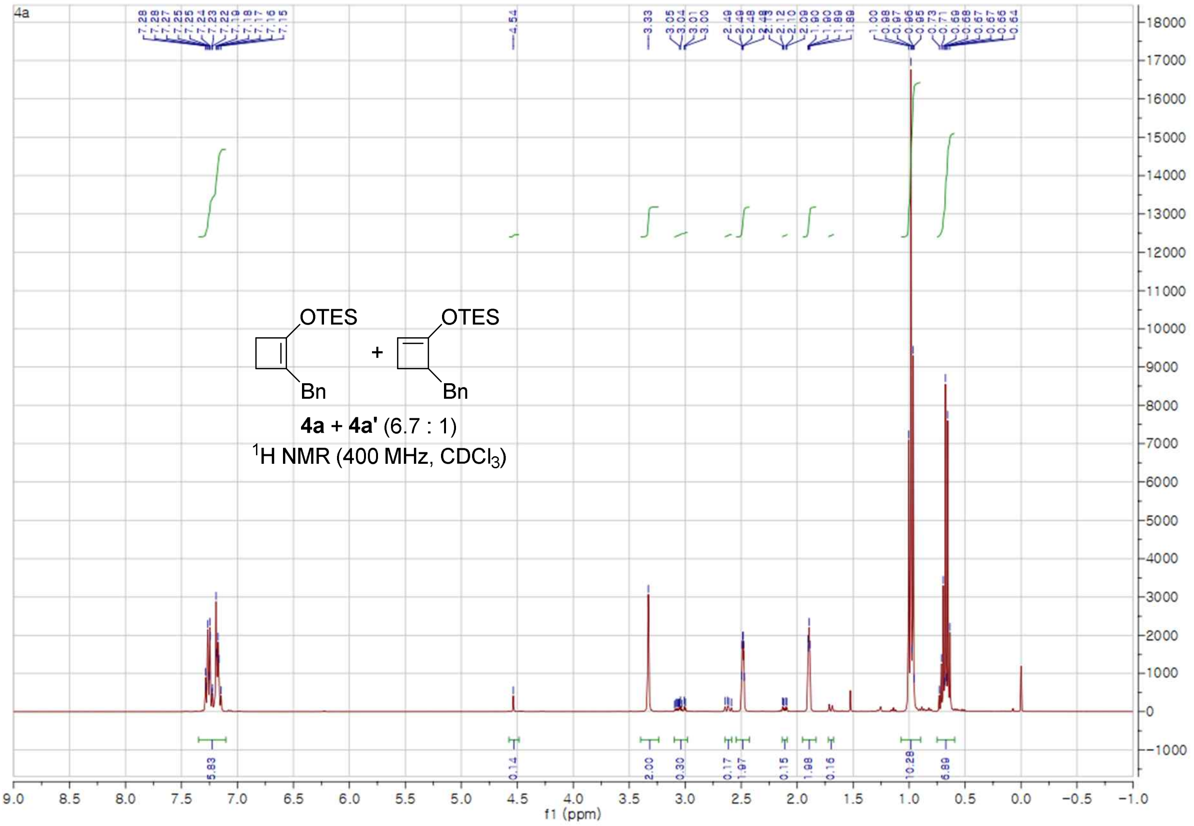
4a
OTES
Bn
+
OTES
Bn
4a + 4a' (6.7 : 1)
1H NMR (400 MHz, CDCl3)
f1 (ppm)

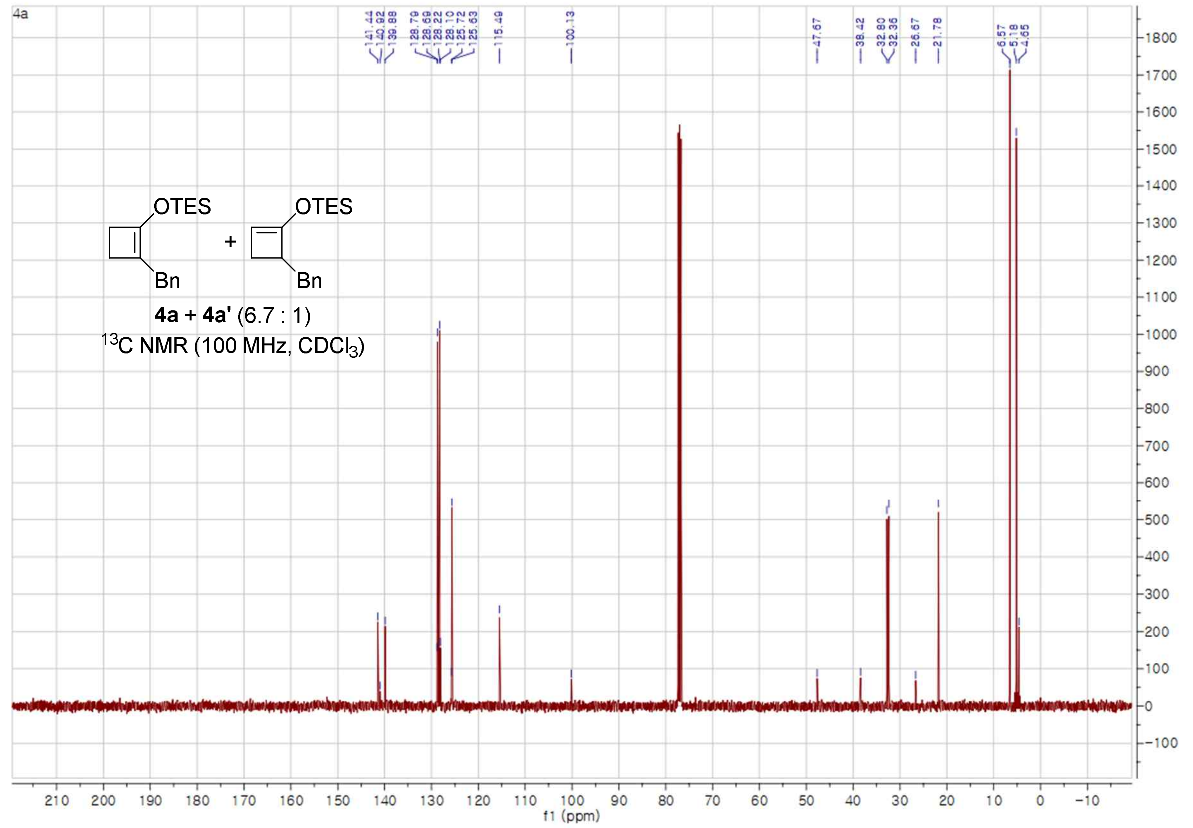
4a
OTES
Bn
+
OTES
Bn
4a + 4a' (6.7 : 1)
13C NMR (100 MHz, CDCl3)
f1 (ppm)

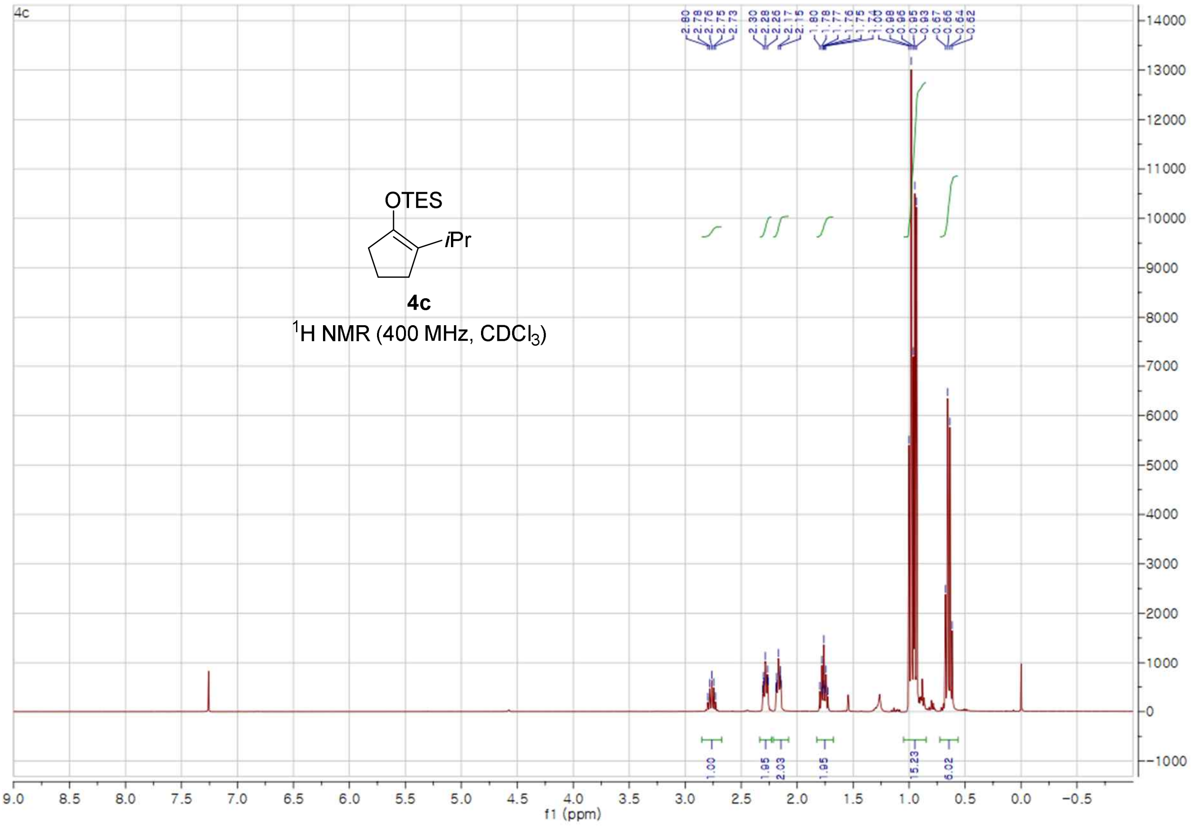
4c
OTES
iPr
4c
1H NMR (400 MHz, CDCl3)
1.00
1.95
2.03
1.95
15.23
6.02
f1 (ppm)

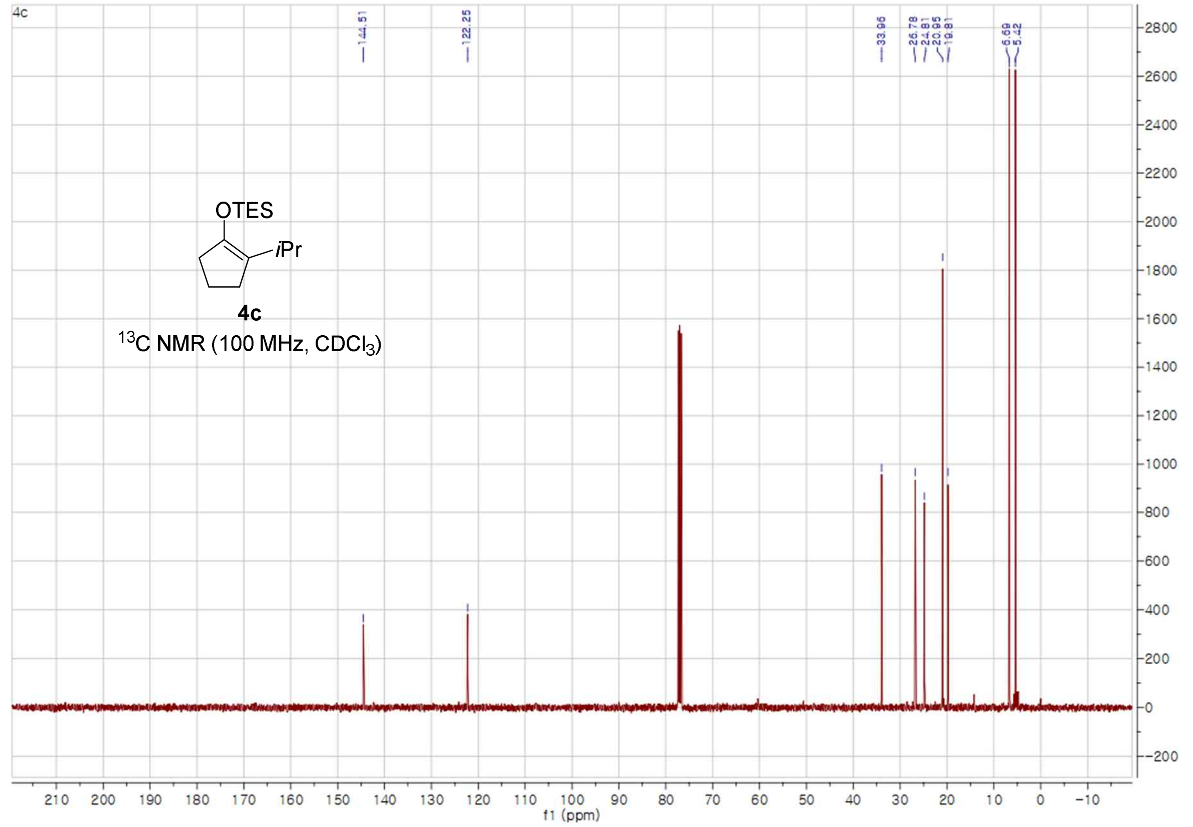
4c
144.51
122.25
33.96
25.78
24.81
20.95
19.81
6.69
5.42
OTES
iPr
4c
13C NMR (100 MHz, CDCl3)
f1 (ppm)

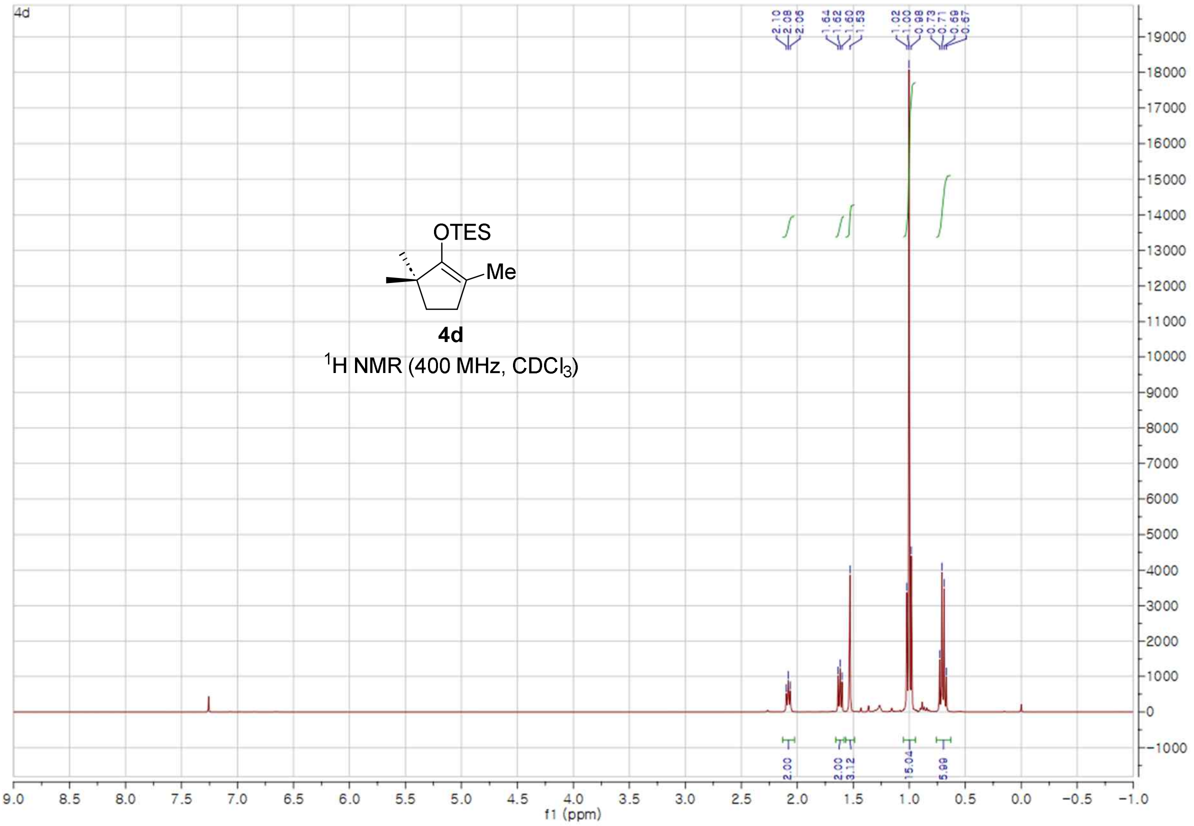

4d
2.10
2.08
2.06
1.64
1.62
1.60
1.53
1.02
1.00
0.98
0.73
0.71
0.69
0.67
OTES
Me
4d
1H NMR (400 MHz, CDCl3)
2.00
2.00
3.12
15.04
5.99
f1 (ppm)

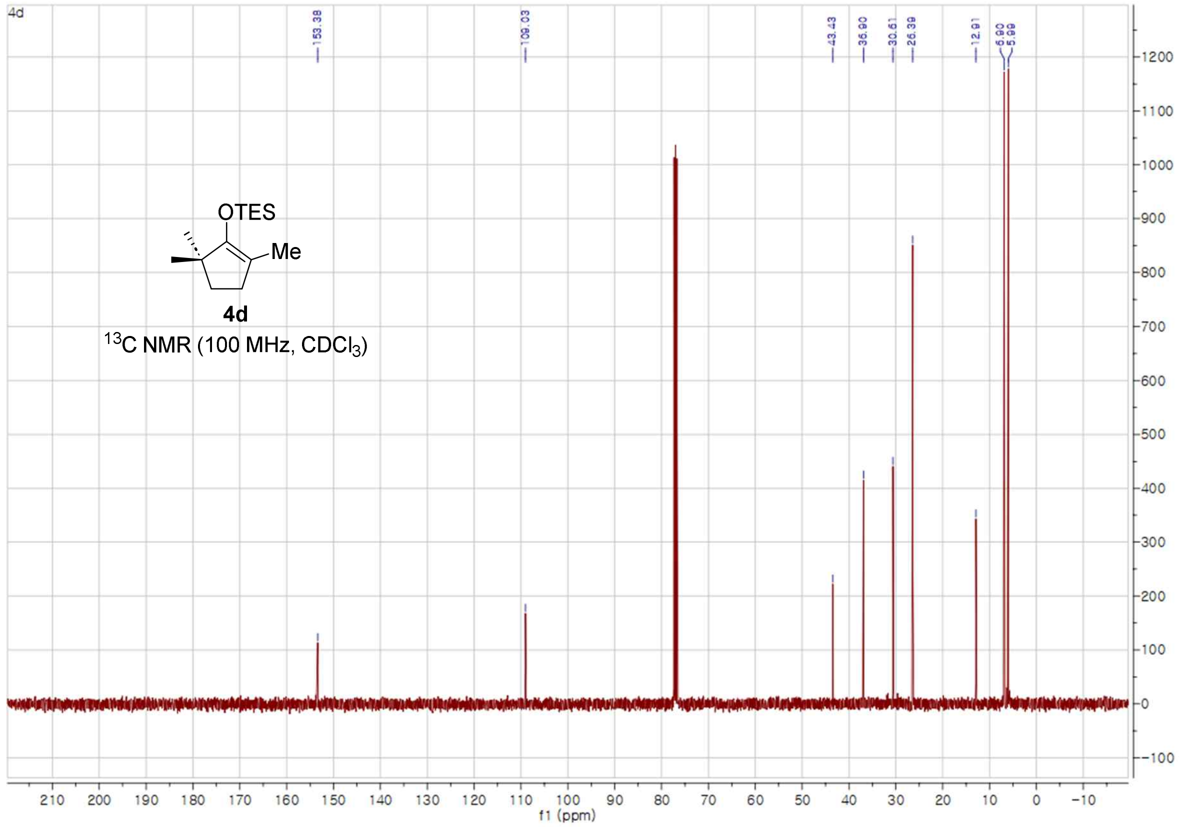

4d
153.38
109.03
43.43
36.90
30.61
26.39
12.91
6.90
5.99
OTES
Me
4d
13C NMR (100 MHz, CDCl3)
f1 (ppm)

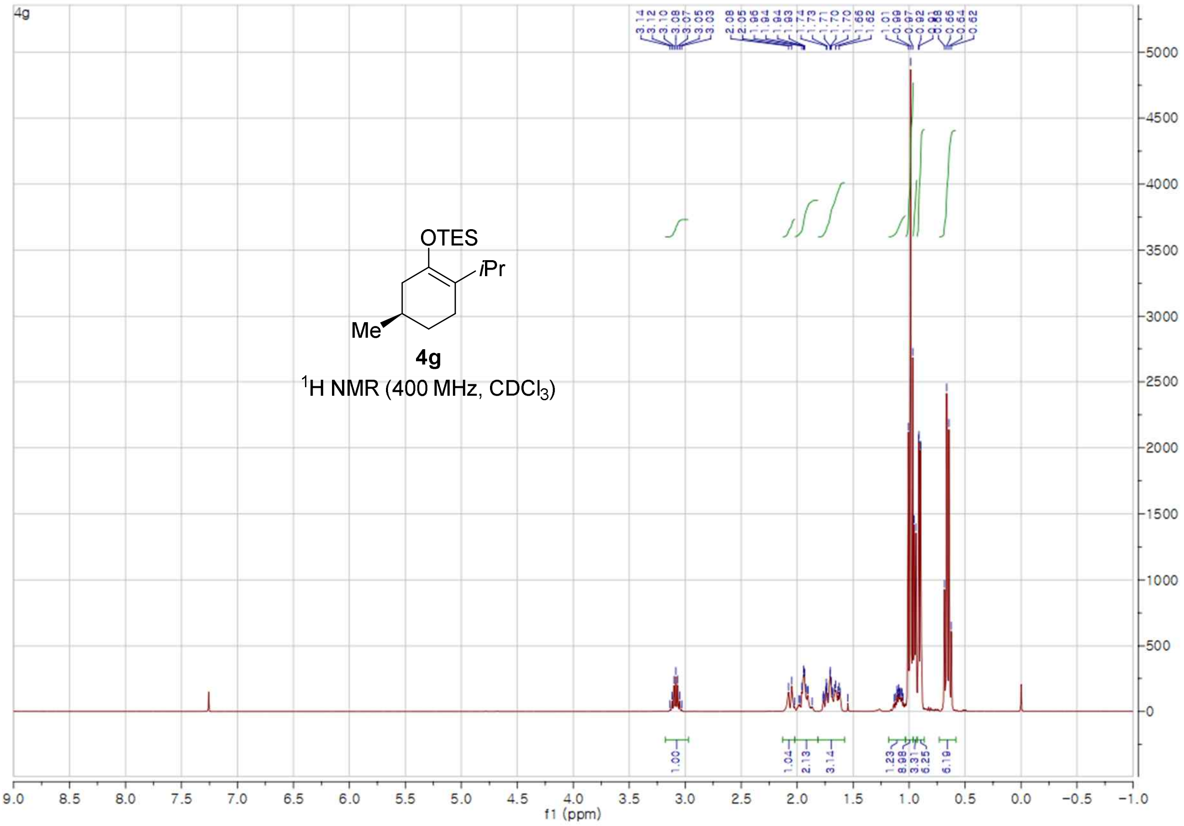
4g
OTES
iPr
Me
4g
1H NMR (400 MHz, CDCl3)
f1 (ppm)

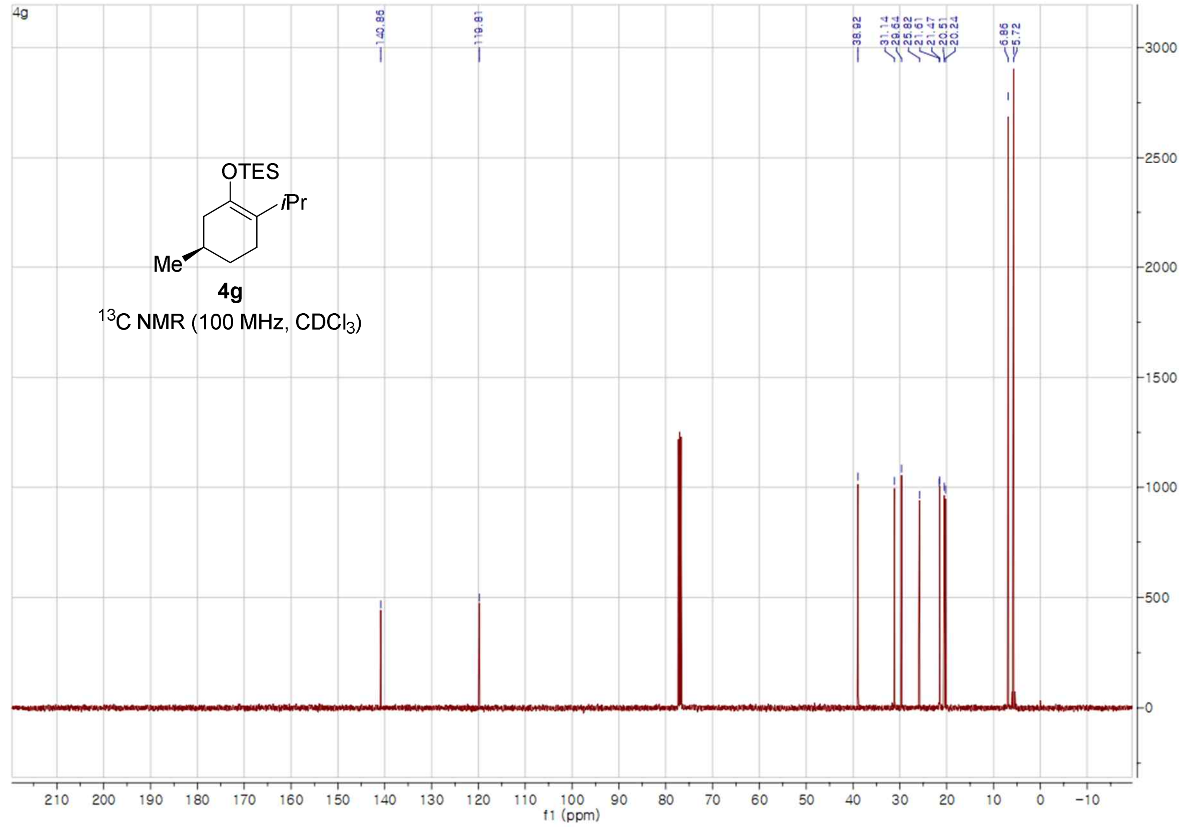
4g
140.86
119.81
38.92
OTES
iPr
Me
4g
13C NMR (100 MHz, CDCl3)
f1 (ppm)

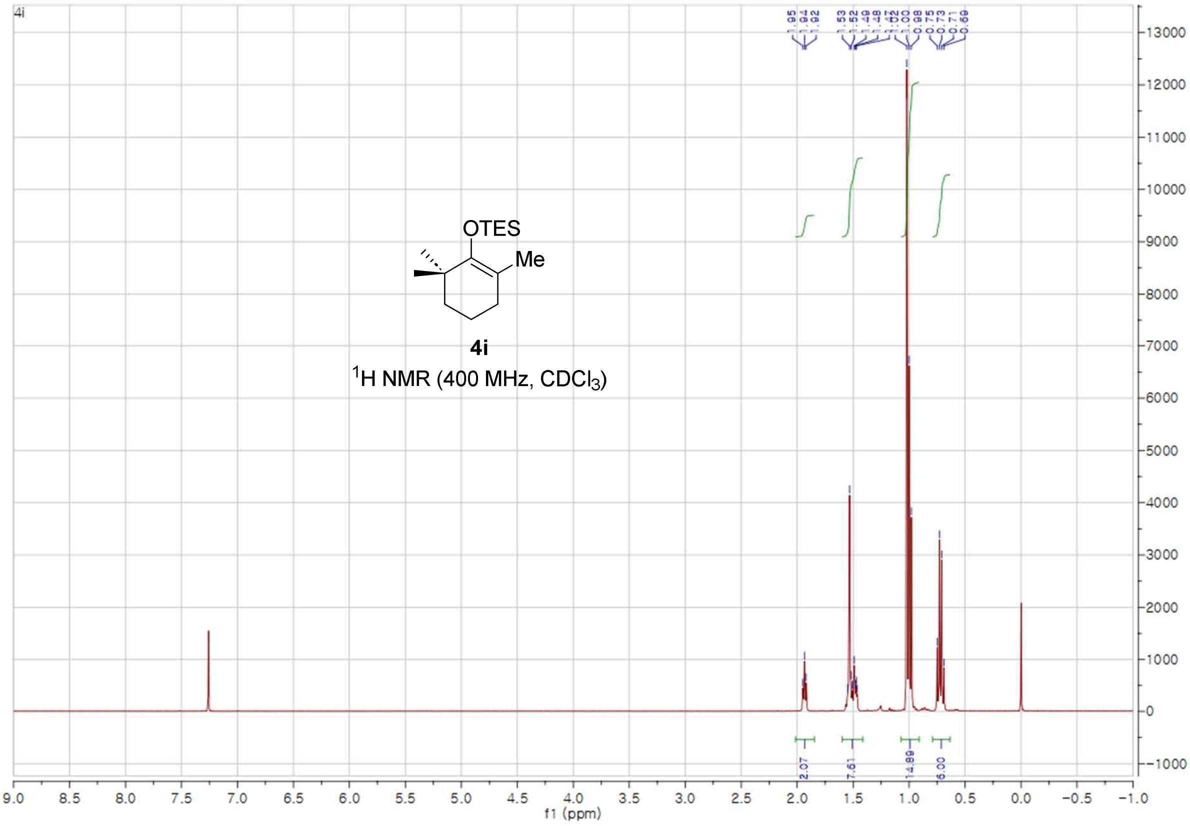
4i
OTES
Me
4i
^{1}H NMR (400 MHz, CDCl$_3$)
1.95
1.94
1.92
1.53
1.52
1.49
1.48
1.47
1.02
1.00
0.98
0.75
0.73
0.71
0.69
2.07
7.61
14.89
6.00
f1 (ppm)

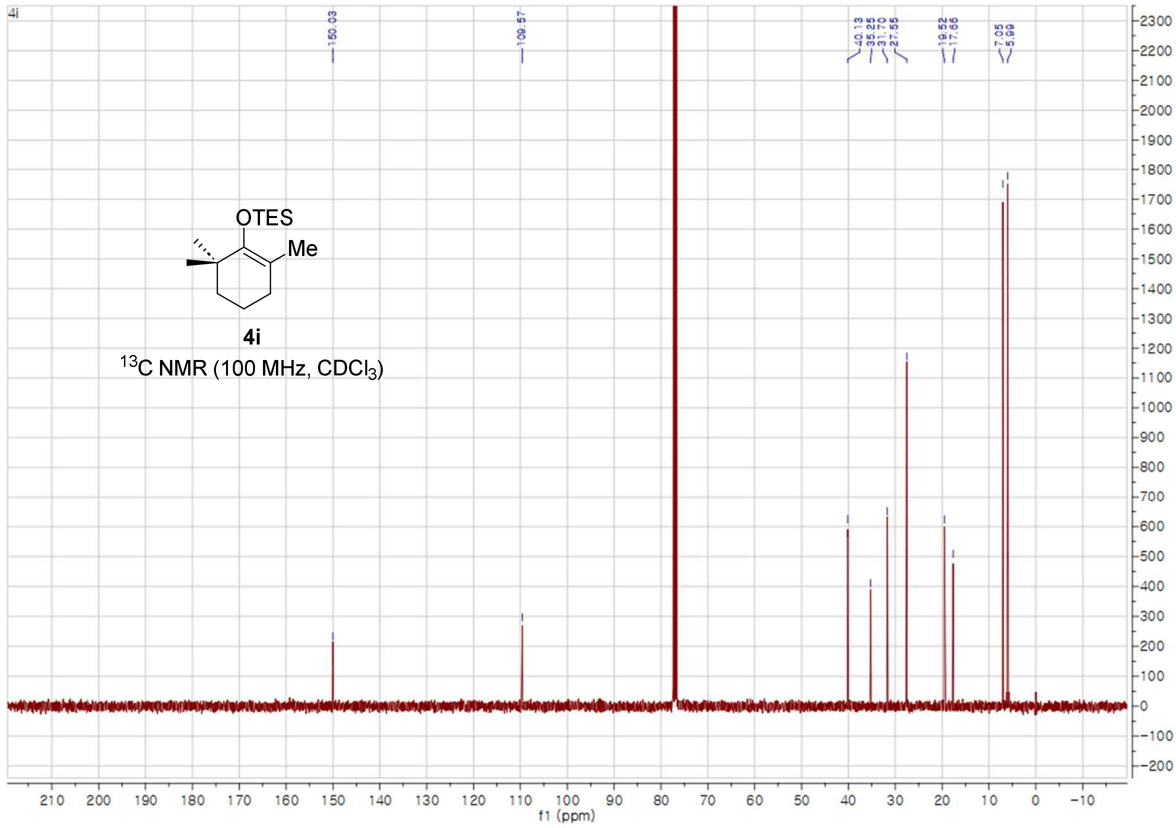
4i
OTES
Me
4i
^{13}C NMR (100 MHz, CDCl$_3$)
150.03
109.57
40.13
35.25
31.70
27.55
19.52
17.56
7.05
5.99
f1 (ppm)

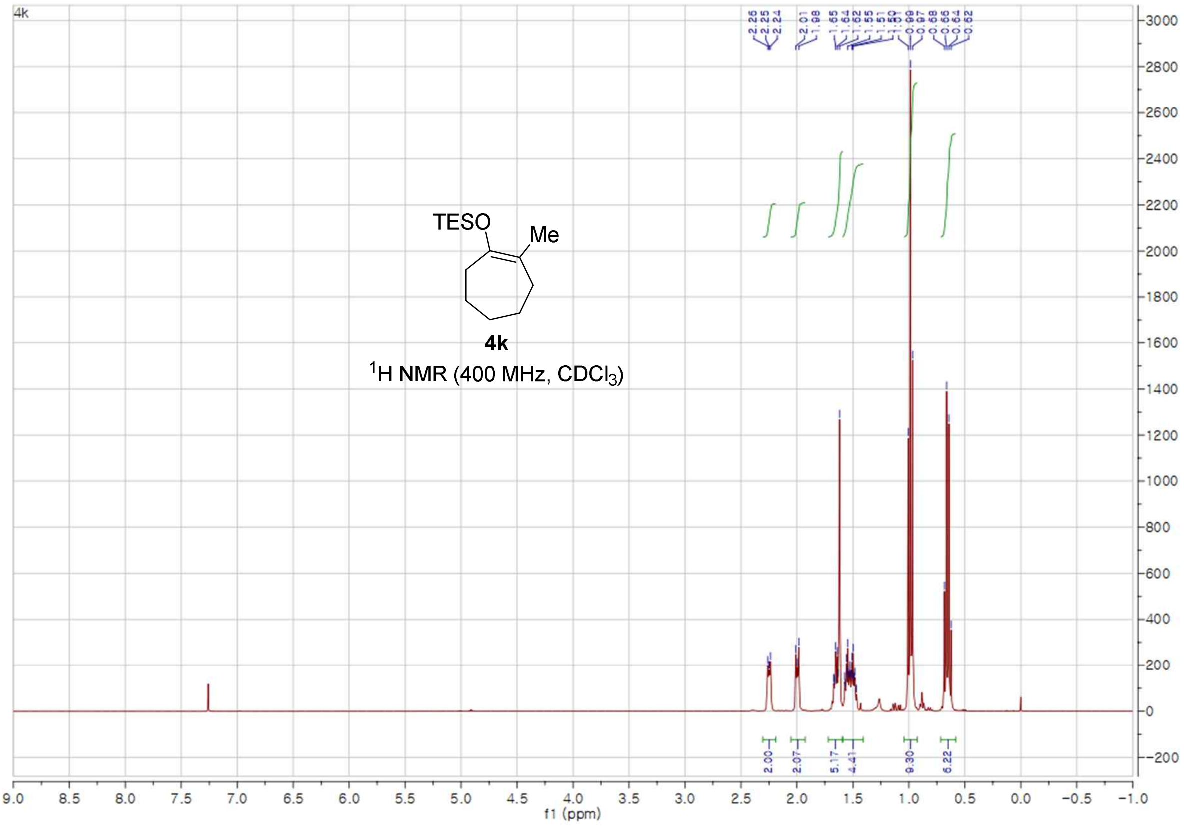
4k
TESO
Me
4k
^{1}H NMR (400 MHz, CDCl$_3$)
2.26
2.25
2.24
2.01
1.98
1.65
1.64
1.62
1.62
1.55
1.51
1.50
0.99
0.97
0.68
0.66
0.64
0.62
2.00
2.07
5.17
4.41
9.30
6.22
f1 (ppm)

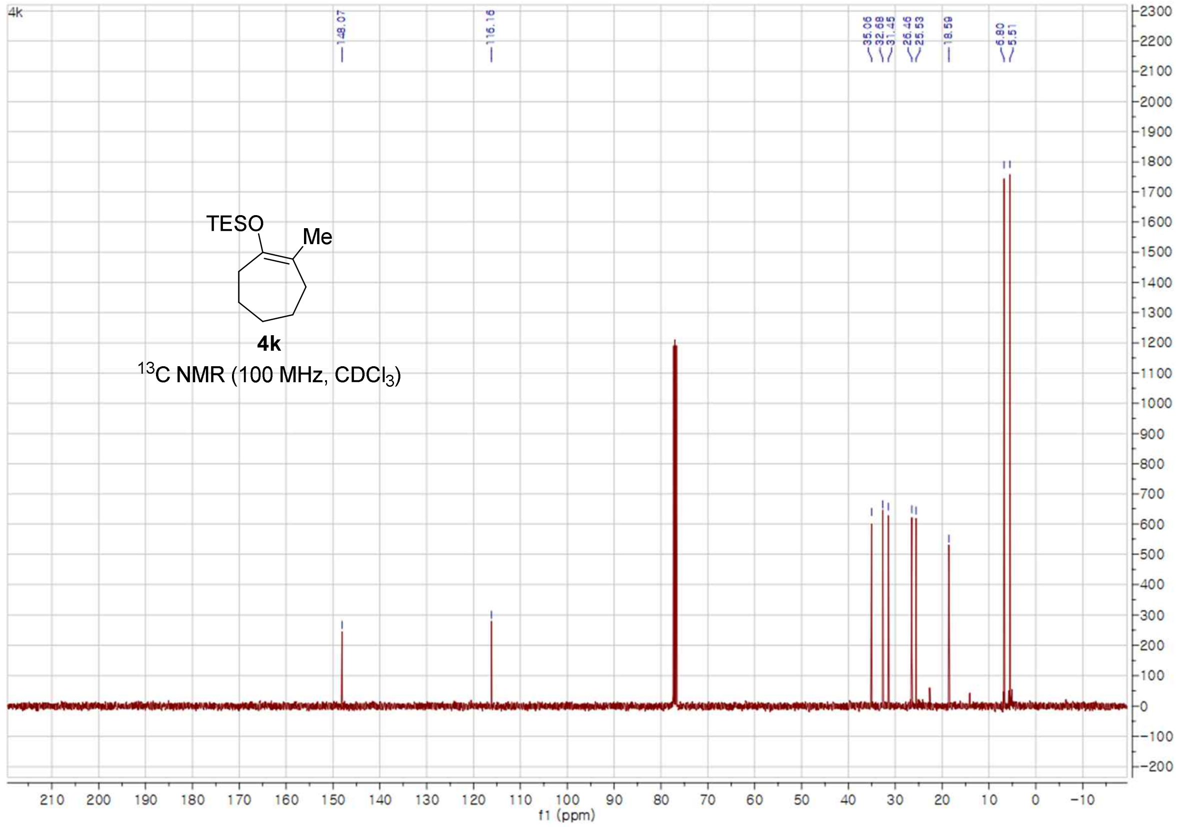
4k
TESO
Me
4k
^{13}C NMR (100 MHz, CDCl$_3$)
148.07
116.16
35.06
32.68
31.45
26.45
25.53
18.59
6.80
5.51
f1 (ppm)

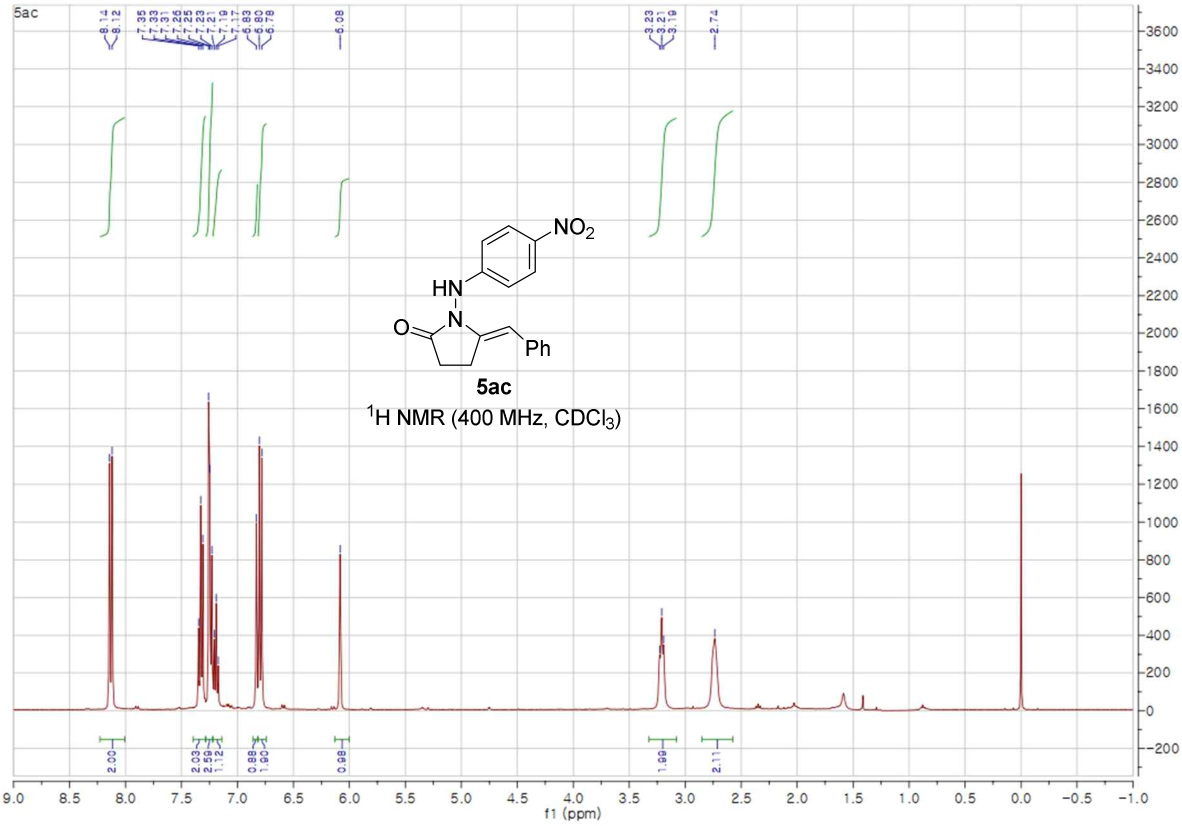
5ac
8.14
8.12
7.35
7.33
7.31
7.26
7.25
7.23
7.21
7.19
7.17
6.83
6.80
6.78
6.08
3.23
3.21
3.19
2.74
NO2
HN
O
N
Ph
5ac
1H NMR (400 MHz, CDCl3)
2.00
2.03
2.59
1.12
0.88
1.90
0.98
1.99
2.11
f1 (ppm)

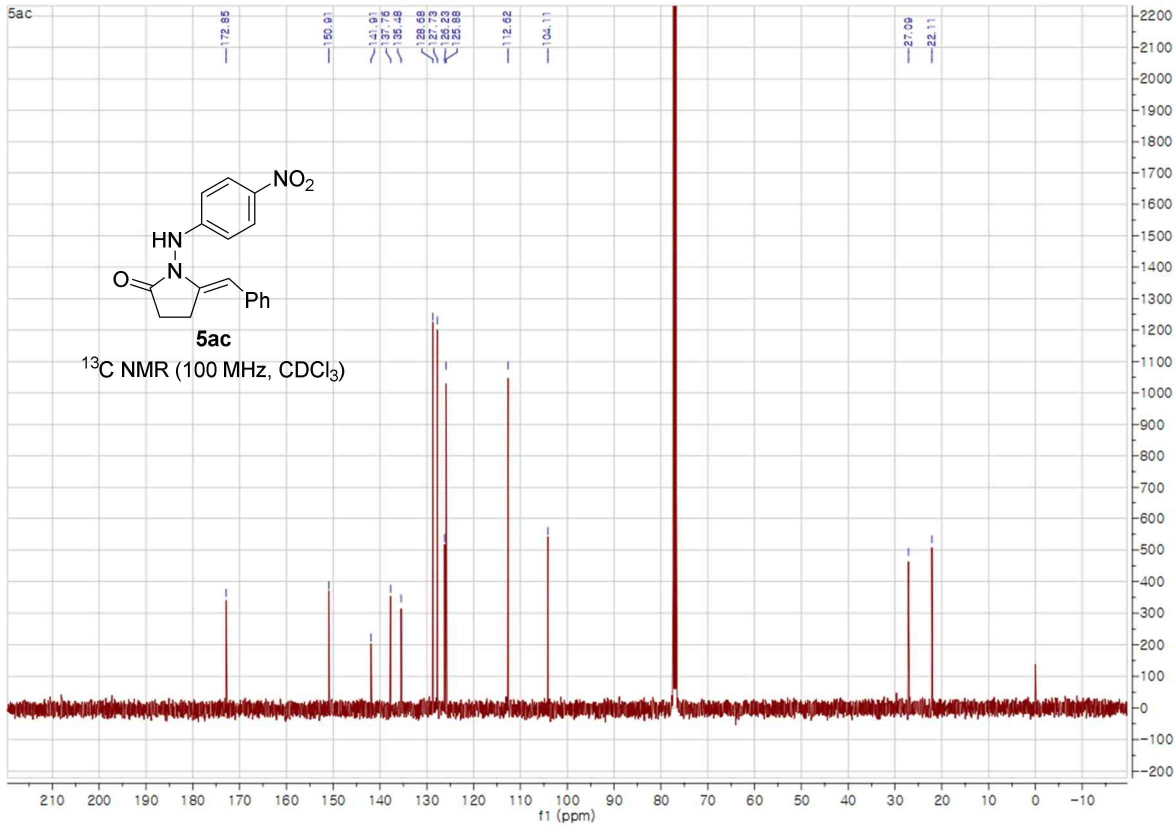
5ac
172.85
150.91
141.91
137.76
135.48
128.68
127.73
126.23
125.88
112.62
104.11
27.09
22.11
NO2
HN
O
N
Ph
5ac
13C NMR (100 MHz, CDCl3)
f1 (ppm)

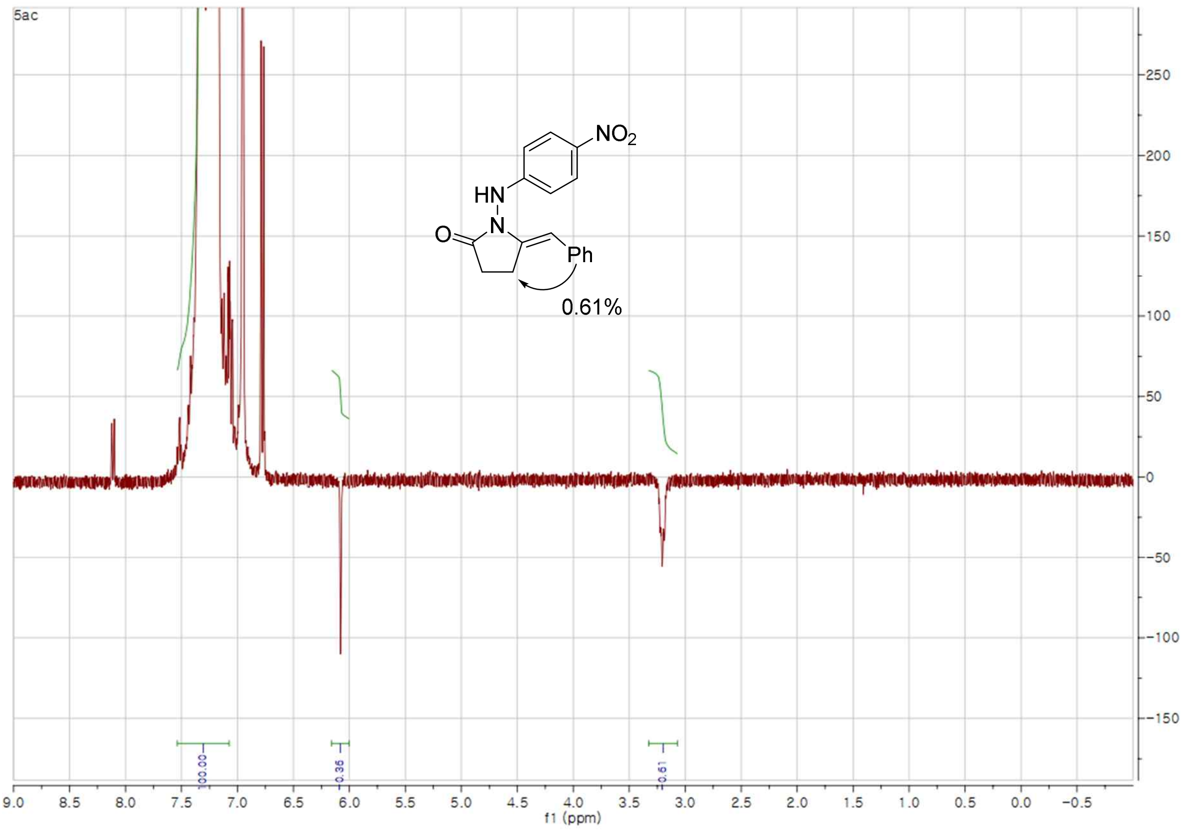
5ac
NO2
HN
N
O
Ph
0.61%
100.00
0.35
0.61
f1 (ppm)

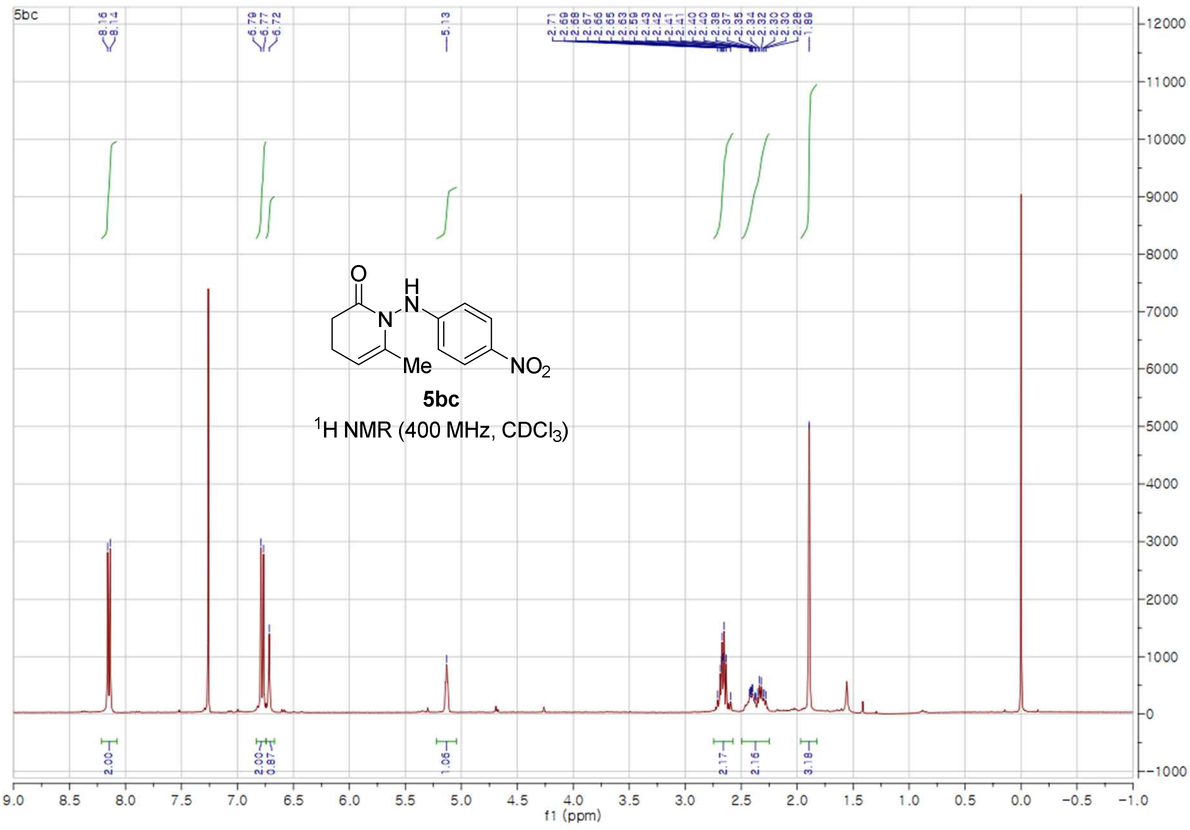
5bc
8.15
8.14
6.79
6.77
6.72
5.13
1.89
5bc
¹H NMR (400 MHz, CDCl₃)
2.00
2.00
0.87
1.05
2.17
2.16
3.18
f1 (ppm)

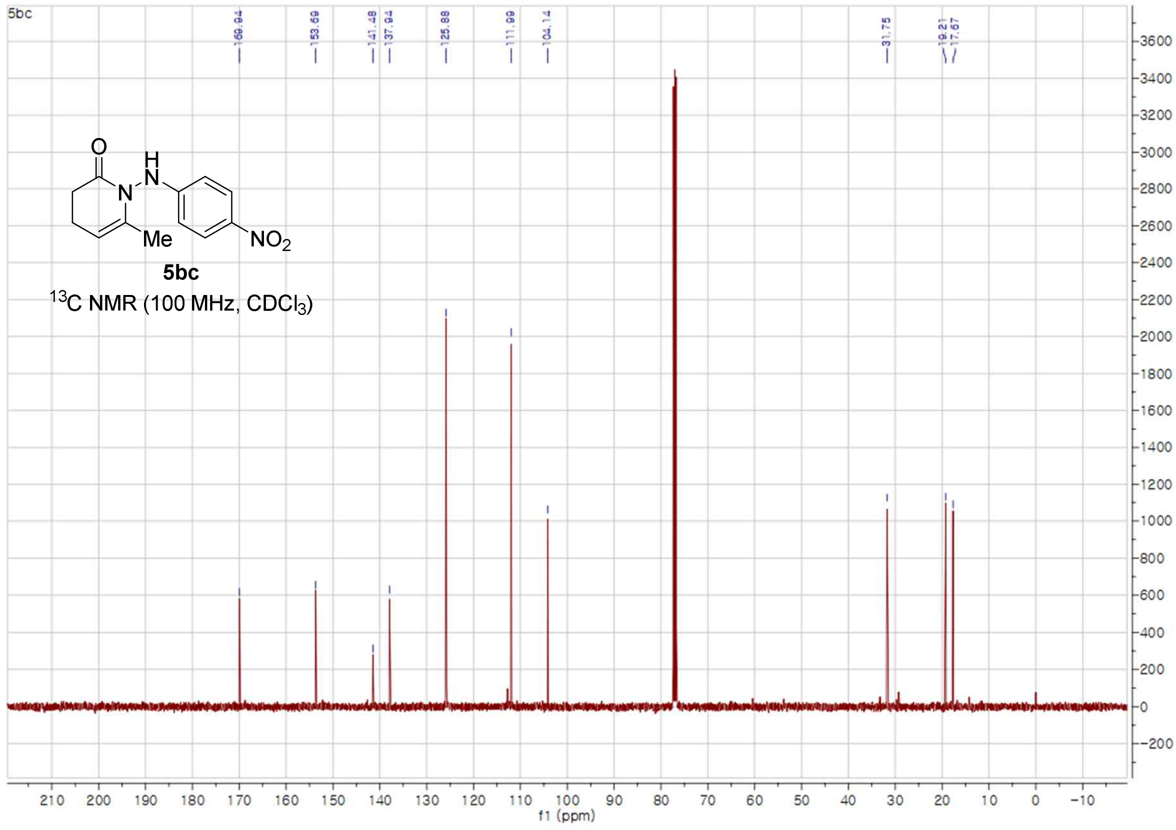
5bc
169.94
153.69
141.48
137.94
125.88
111.99
104.14
31.75
19.21
17.67
5bc
¹³C NMR (100 MHz, CDCl₃)
f1 (ppm)

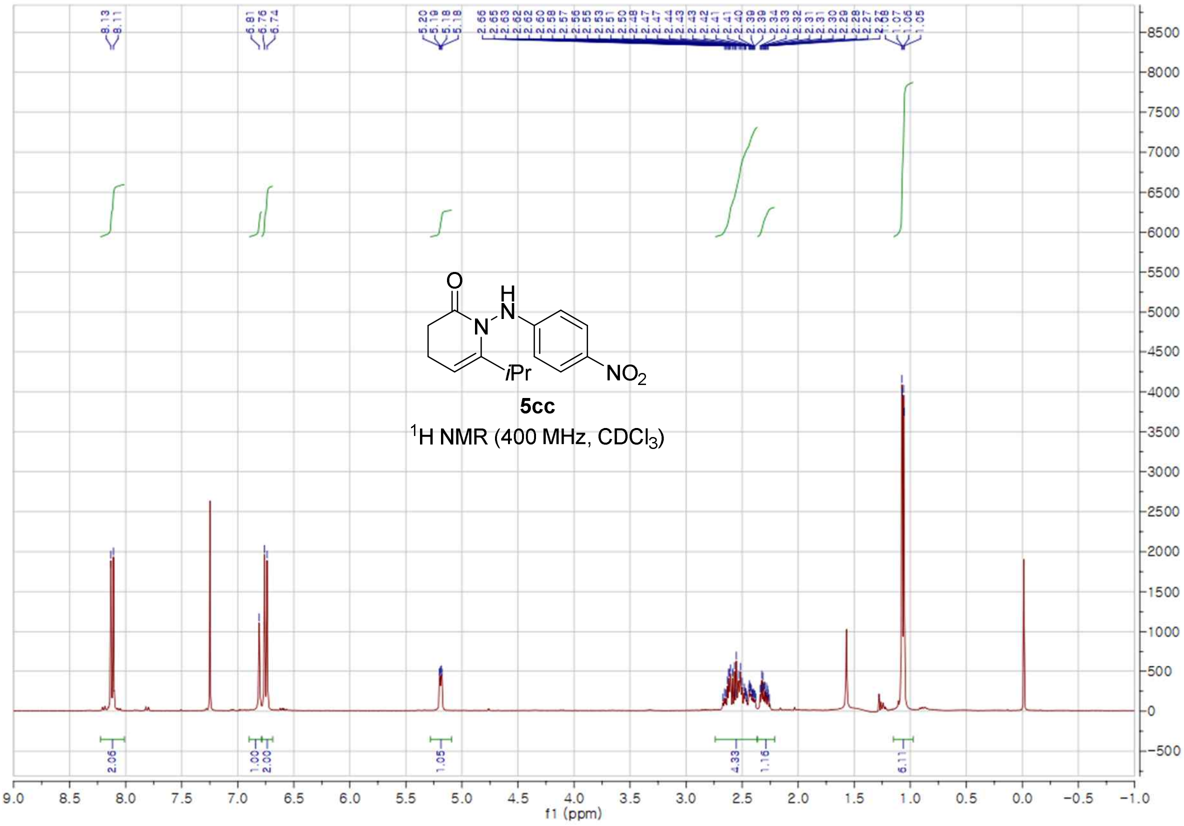
5cc
¹H NMR (400 MHz, CDCl₃)
f1 (ppm)

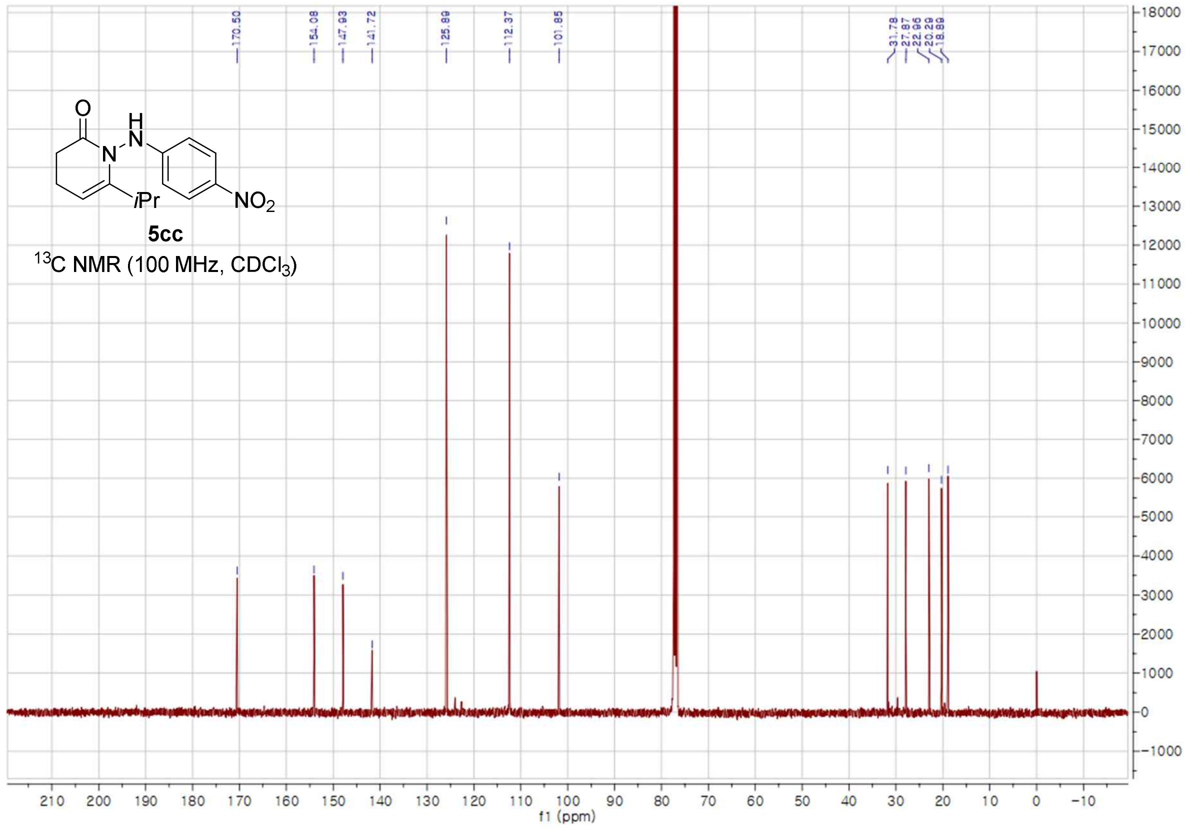
5cc
¹³C NMR (100 MHz, CDCl₃)
f1 (ppm)

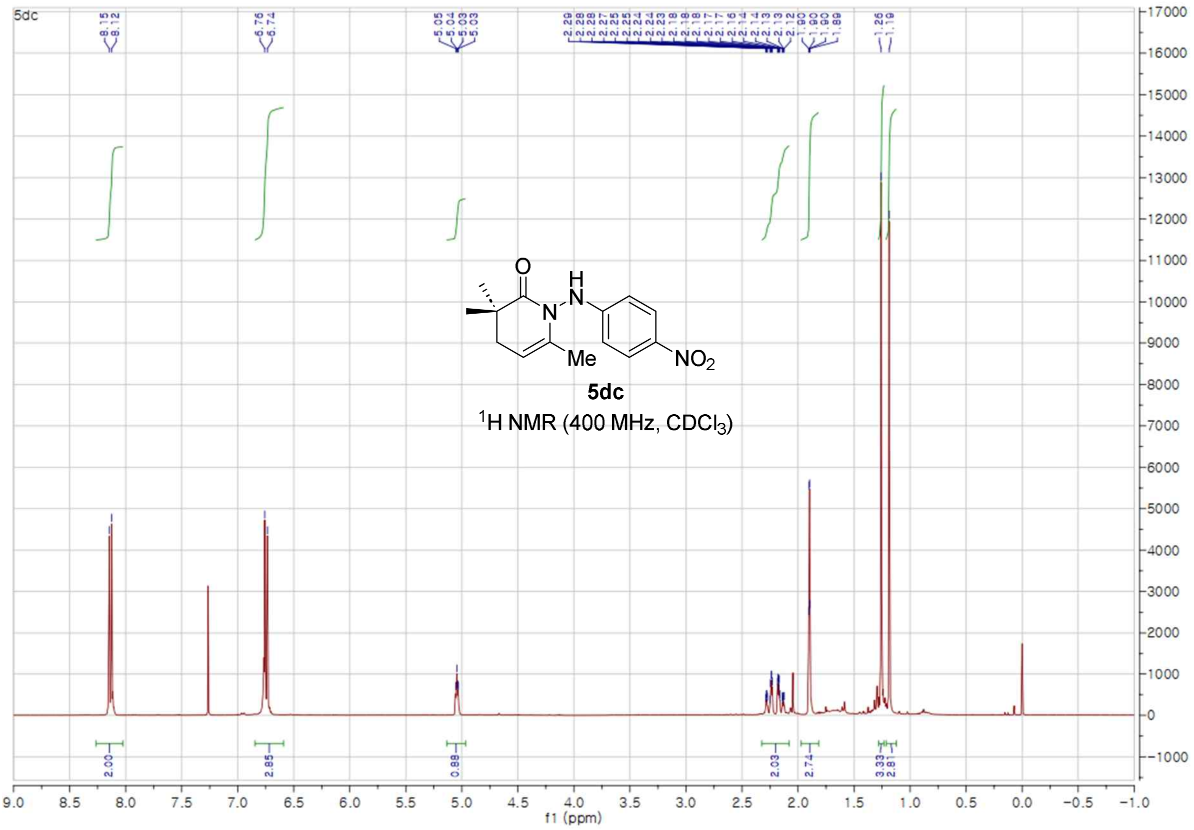
5dc
8.15
8.12
6.76
6.74
5.05
5.04
5.03
5.03
1.90
1.90
1.89
1.26
1.19
2.00
2.85
0.88
2.03
2.74
3.33
2.81
5dc
¹H NMR (400 MHz, CDCl₃)
f1 (ppm)

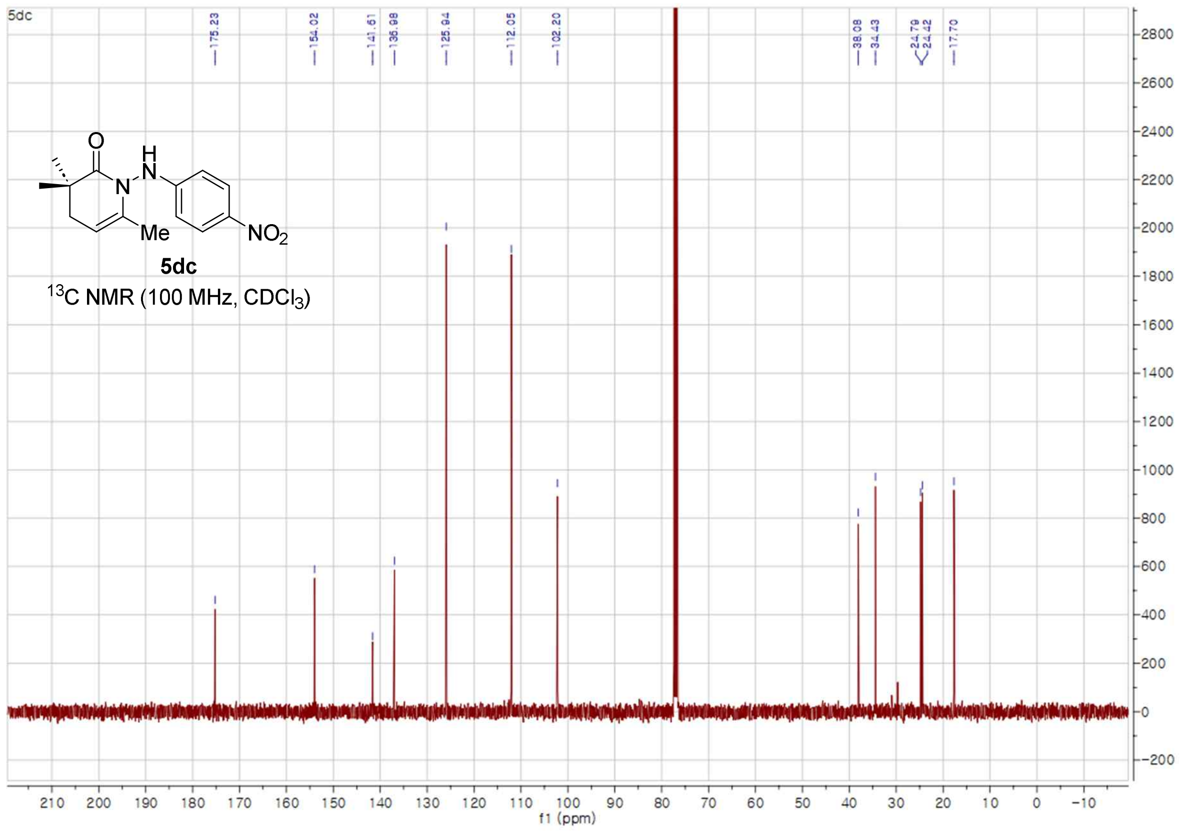
5dc
175.23
154.02
141.61
136.98
125.94
112.05
102.20
38.08
34.43
24.79
24.42
17.70
5dc
¹³C NMR (100 MHz, CDCl₃)
f1 (ppm)

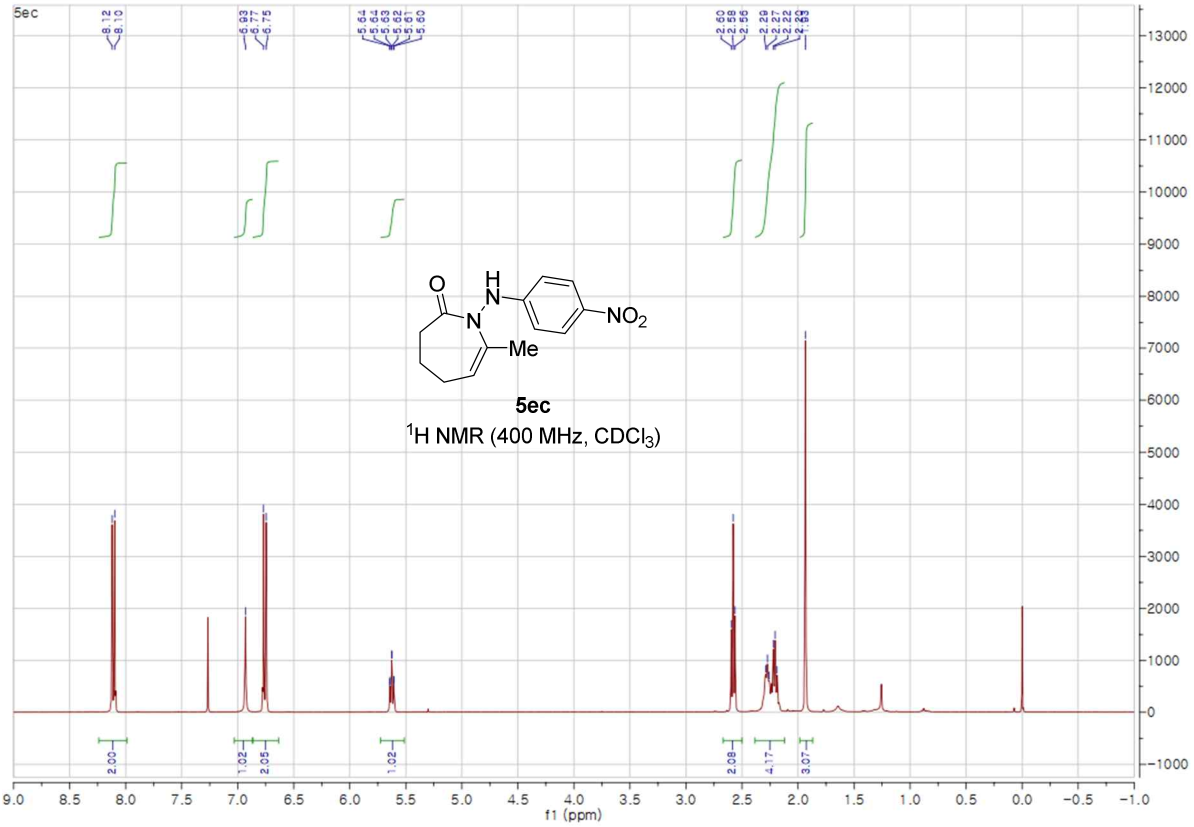
5ec
8.12
8.10
6.93
6.77
6.75
5.64
5.64
5.63
5.62
5.62
5.61
5.60
2.60
2.58
2.56
2.29
2.27
2.22
2.22
2.20
1.93
O
H
N
N
NO_2
Me
5ec
^{1}H NMR (400 MHz, $CDCl_3$)
2.00
1.02
2.05
1.02
2.08
4.17
3.07
f1 (ppm)

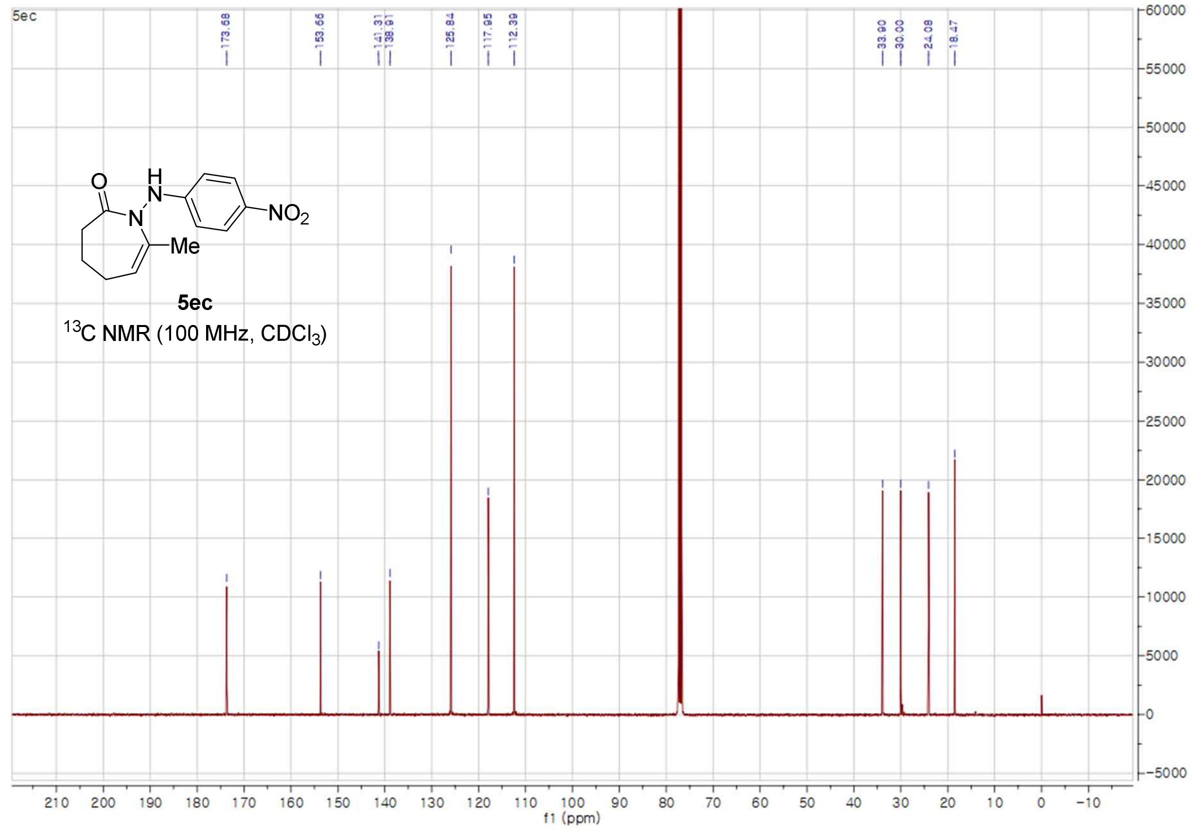
5ec
173.68
153.66
141.31
138.91
125.84
117.95
112.39
33.90
30.00
24.08
18.47
O
H
N
N
NO_2
Me
5ec
^{13}C NMR (100 MHz, $CDCl_3$)
f1 (ppm)

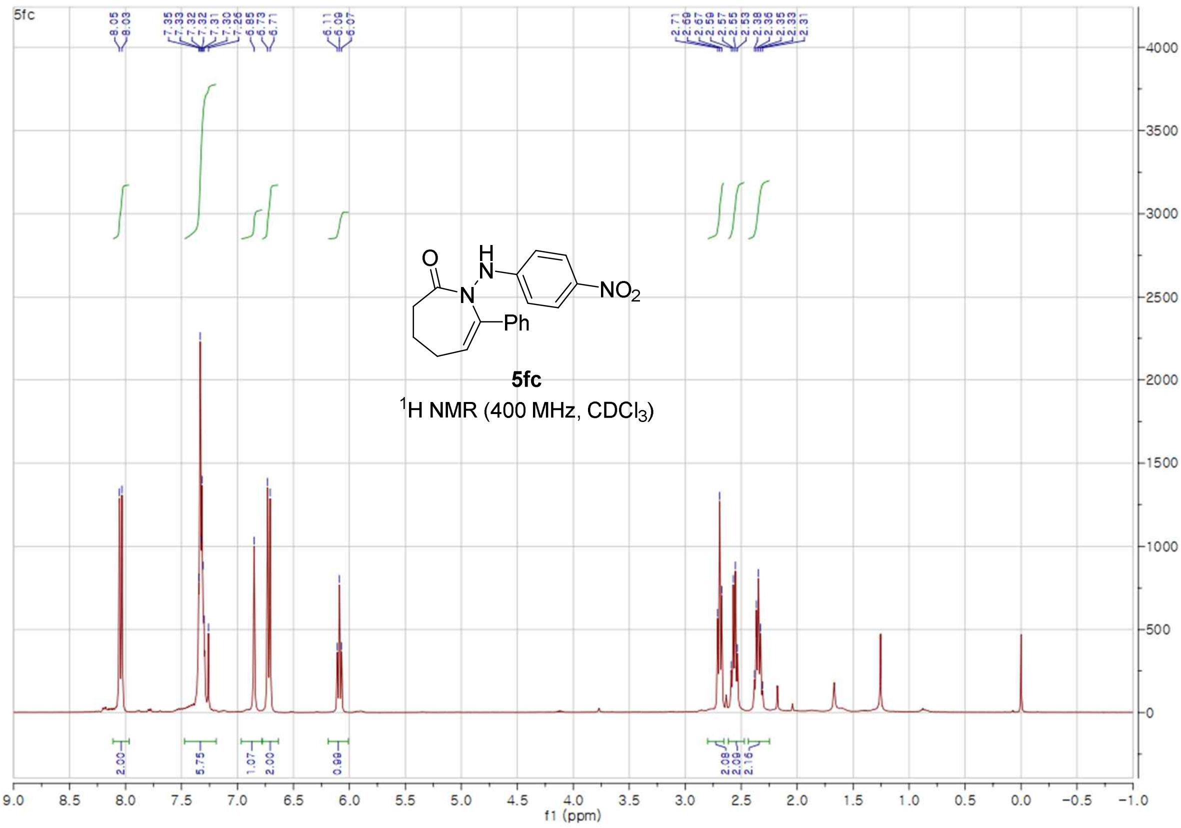
5fc
O
H
N
N
NO2
Ph
5fc
1H NMR (400 MHz, CDCl3)
f1 (ppm)

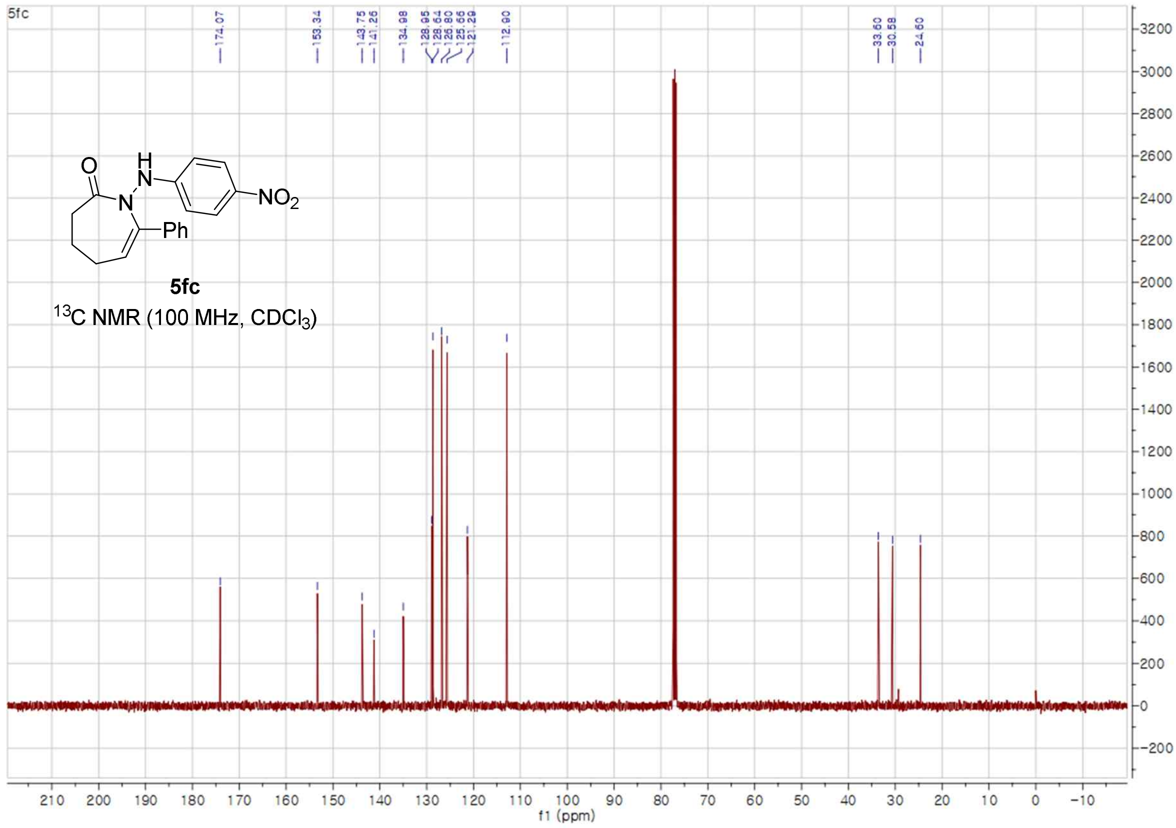
5fc
O
H
N
N
NO2
Ph
5fc
13C NMR (100 MHz, CDCl3)
f1 (ppm)

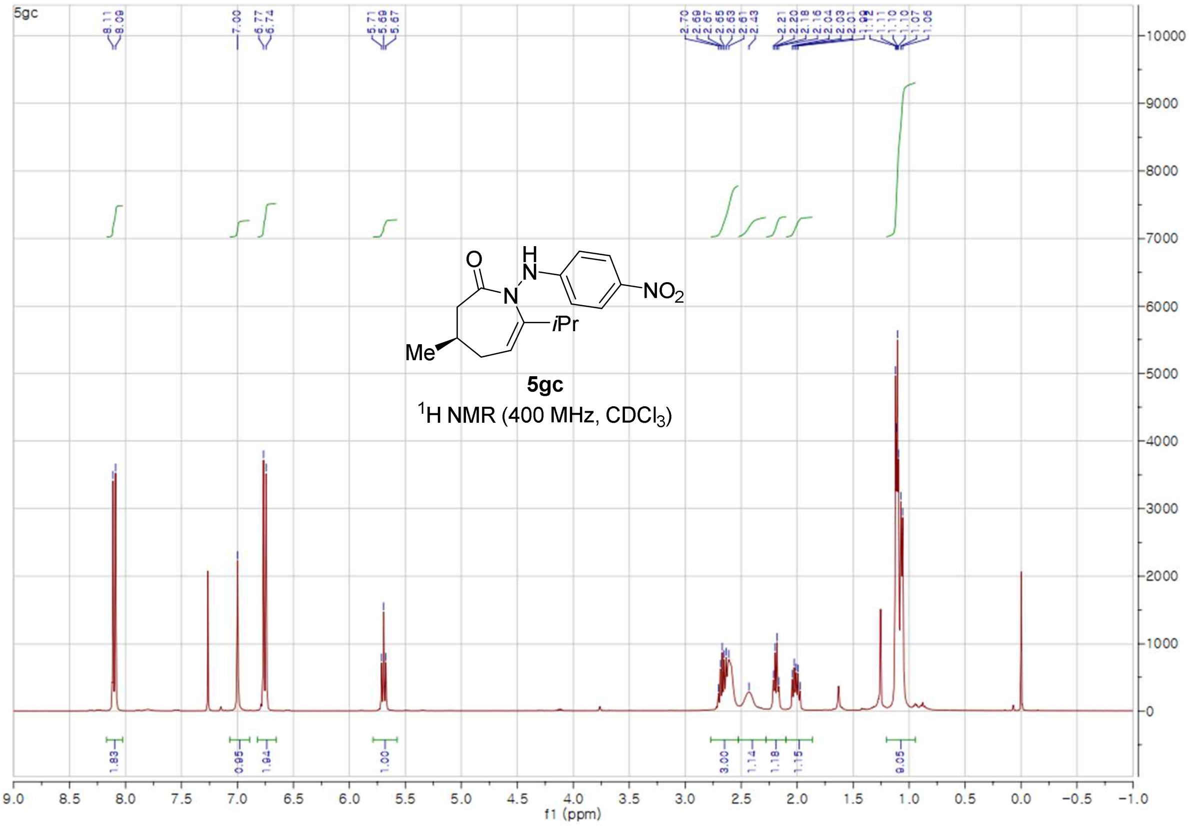
5gc
8.11
8.09
7.00
6.77
6.74
5.71
5.69
5.67
2.70
2.69
2.67
2.65
2.63
2.61
2.43
2.21
2.20
2.18
2.16
2.04
2.03
2.01
1.99
1.12
1.11
1.10
1.07
1.06
5gc
^{1}H NMR (400 MHz, CDCl$_3$)
1.83
0.95
1.94
1.00
3.00
1.14
1.18
1.16
9.05
f1 (ppm)

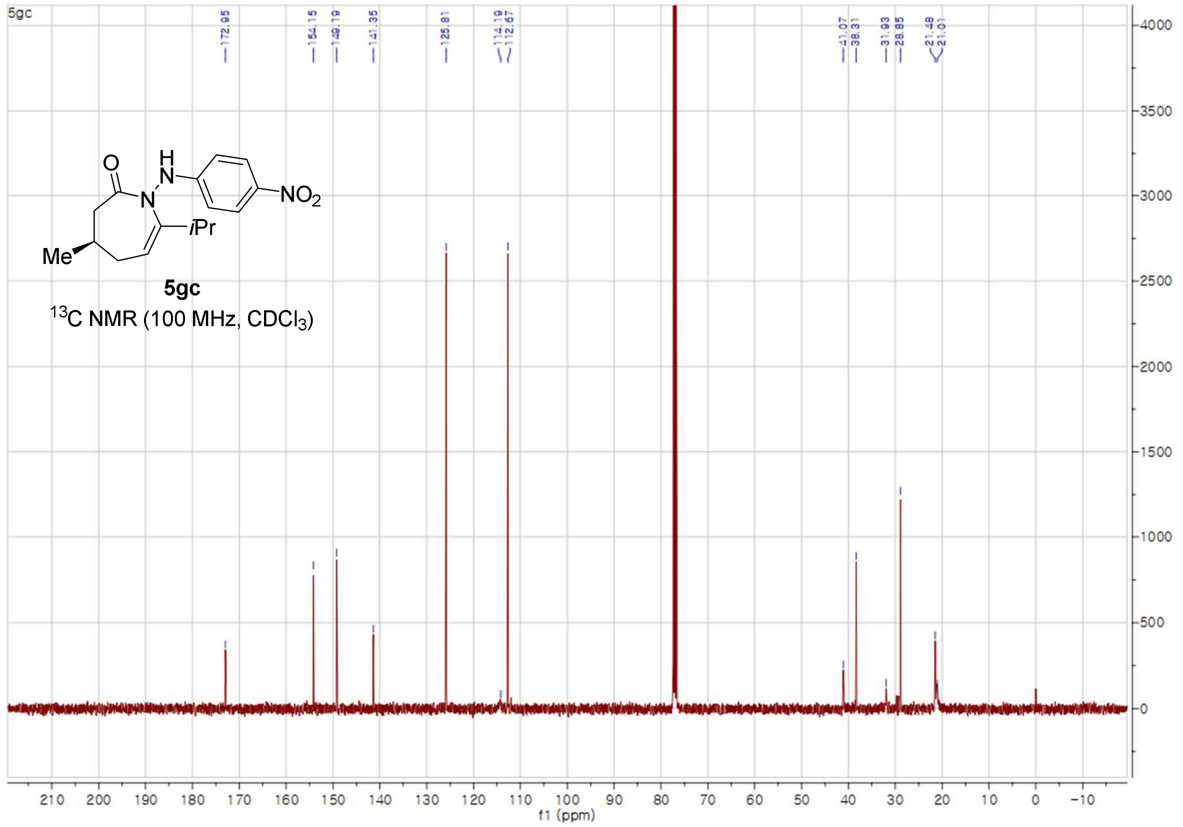
5gc
172.95
154.15
149.19
141.35
125.81
114.19
112.67
41.07
38.31
31.93
28.85
21.48
21.01
5gc
^{13}C NMR (100 MHz, CDCl$_3$)
f1 (ppm)

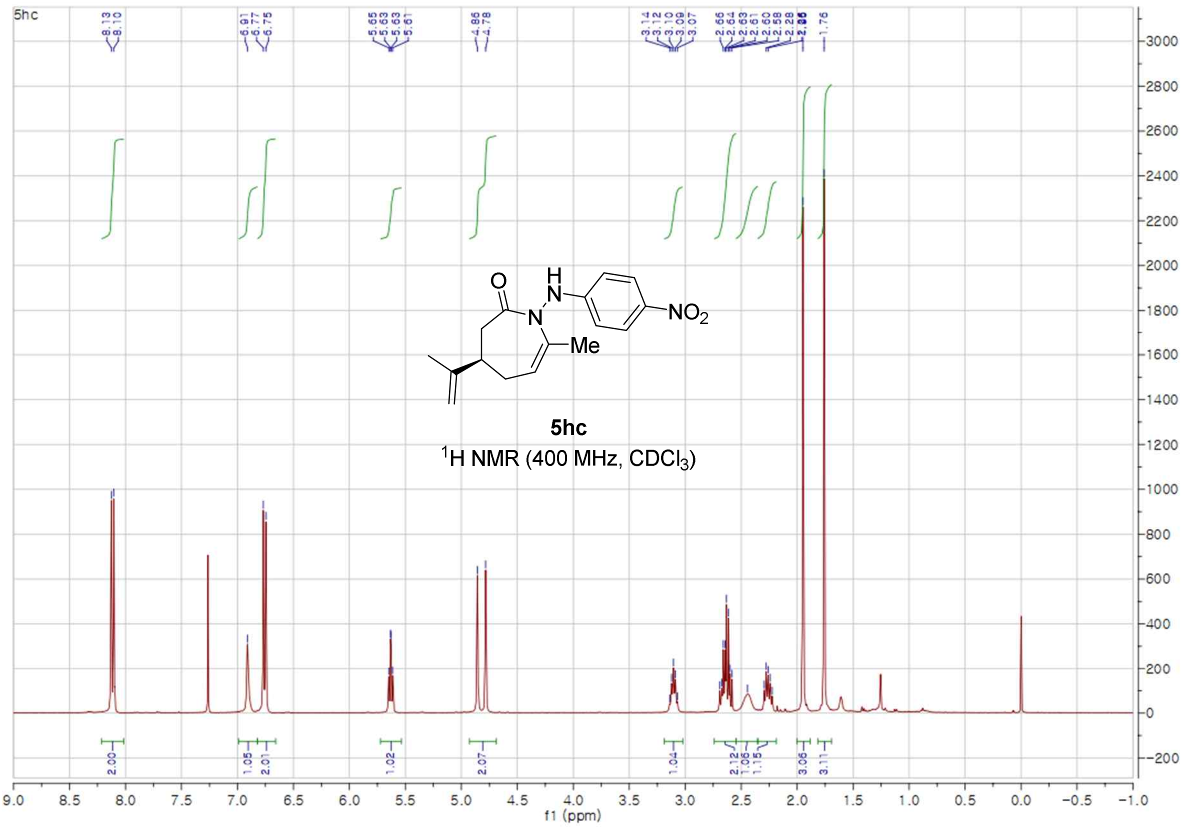

5hc

^{1}H NMR (400 MHz, $CDCl_3$)

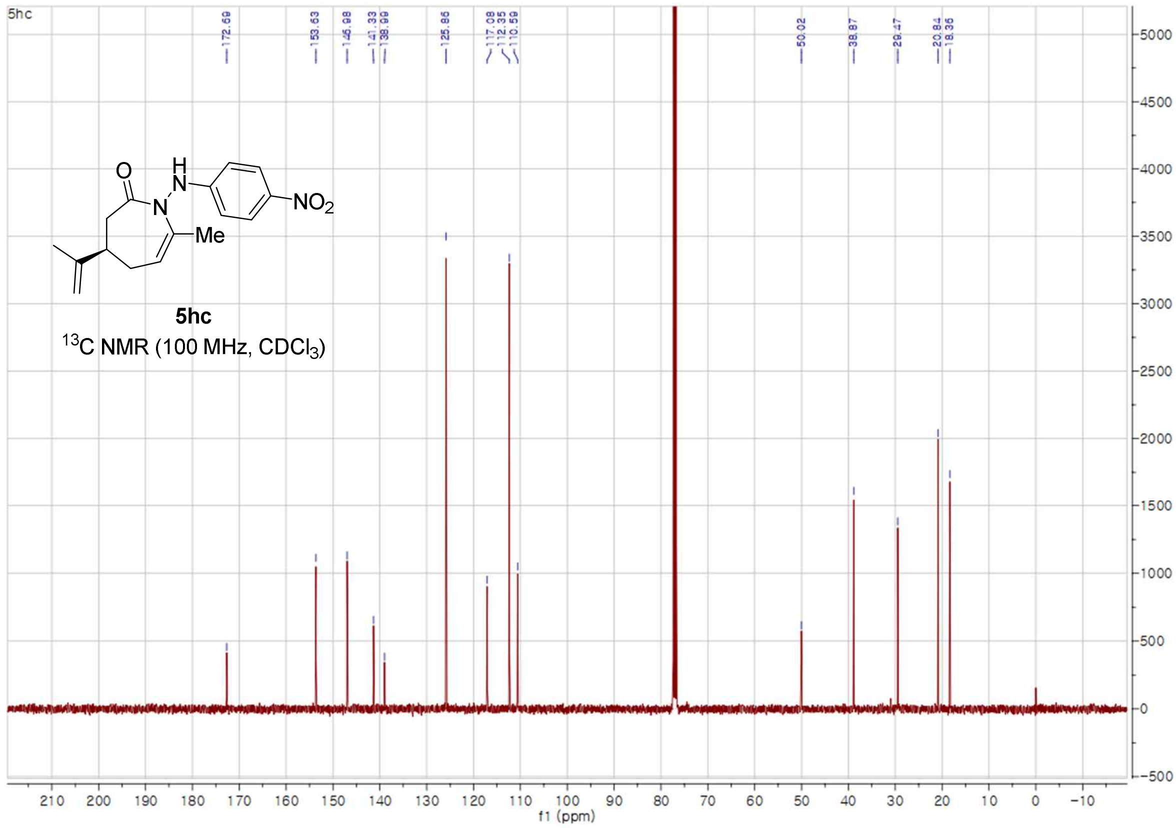

5hc

^{13}C NMR (100 MHz, $CDCl_3$)

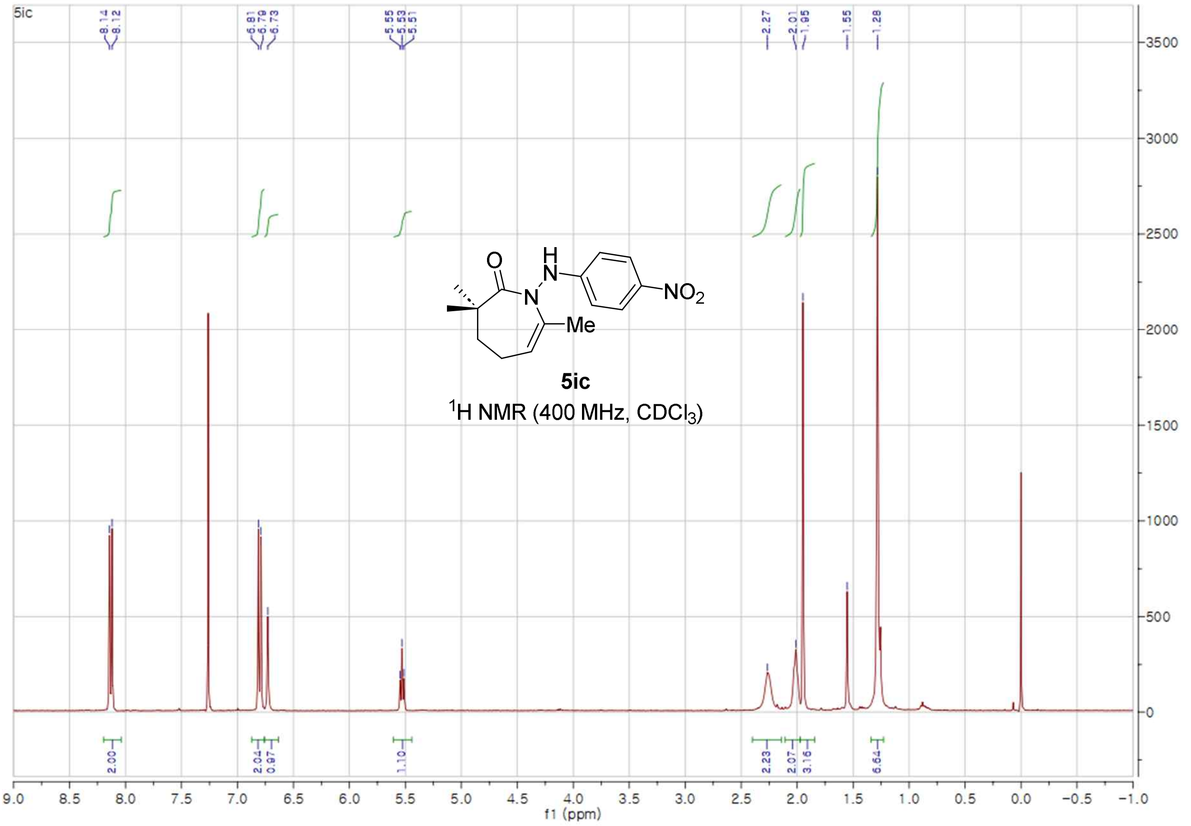
5ic
8.14
8.12
6.81
6.79
6.73
5.55
5.53
5.51
2.27
2.01
1.95
1.55
1.28
O
H
N
N
NO2
Me
5ic
1H NMR (400 MHz, CDCl3)
2.00
2.04
0.97
1.10
2.23
2.07
3.16
6.64
f1 (ppm)

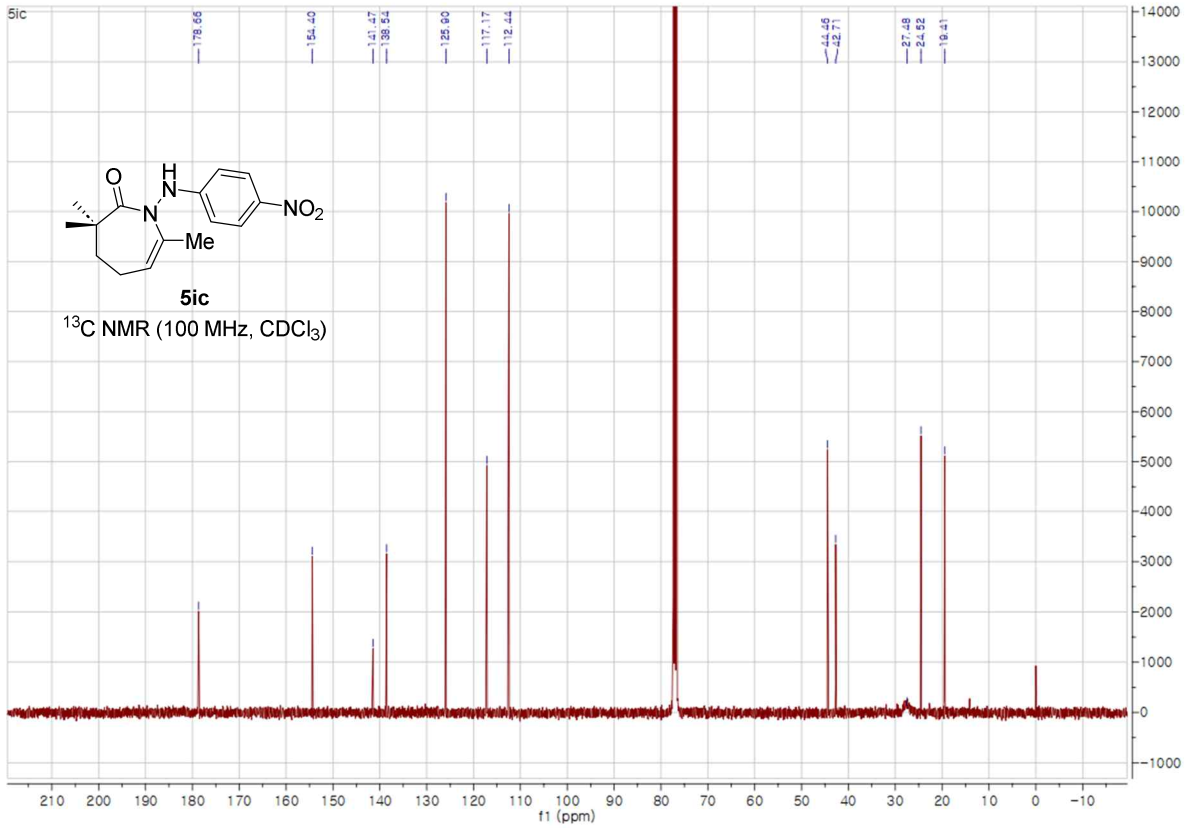
5ic
178.66
154.40
141.47
138.54
125.90
117.17
112.44
44.45
42.71
27.48
24.52
19.41
O
H
N
N
NO2
Me
5ic
13C NMR (100 MHz, CDCl3)
f1 (ppm)

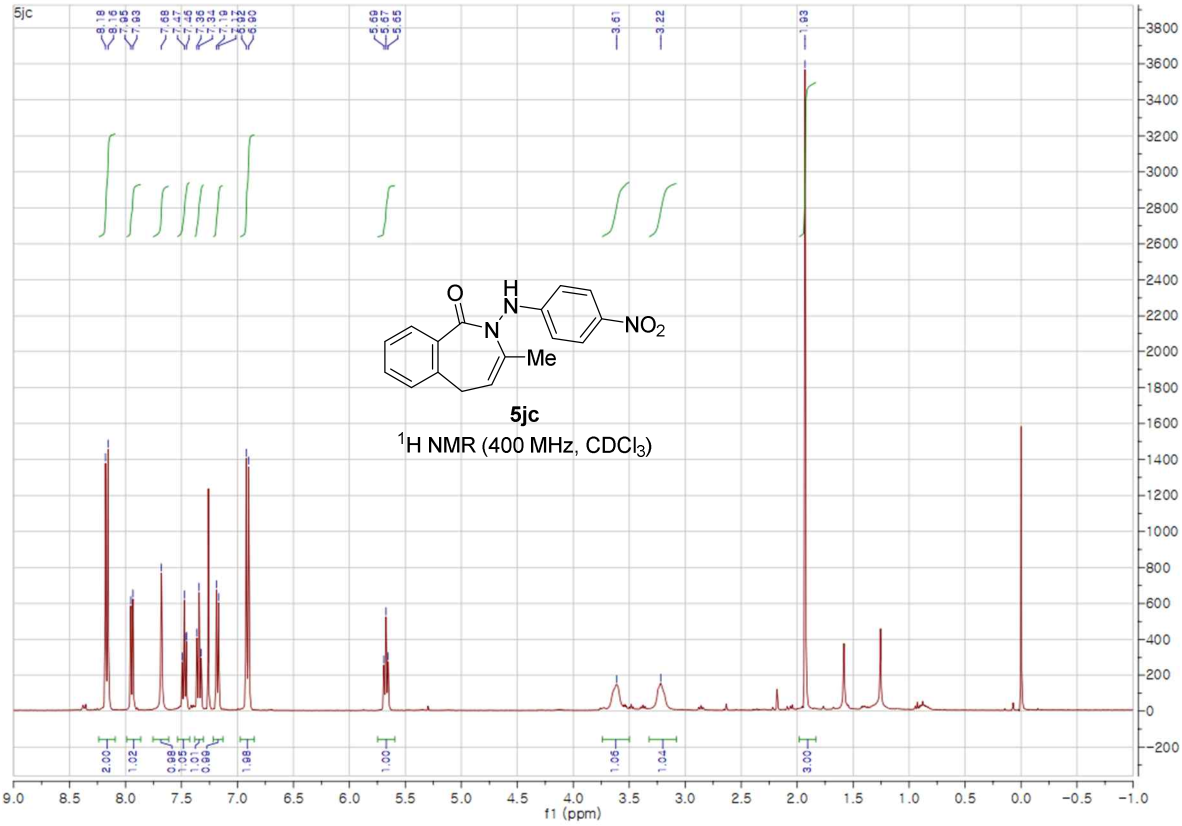
5jc
8.18
8.16
7.95
7.93
7.68
7.47
7.46
7.36
7.34
7.19
7.17
6.92
6.90
5.69
5.67
5.65
3.61
3.22
1.93
NO2
Me
O
N
H
5jc
1H NMR (400 MHz, CDCl3)
2.00
1.02
0.98
1.05
1.01
0.99
1.98
1.00
1.06
1.04
3.00
f1 (ppm)

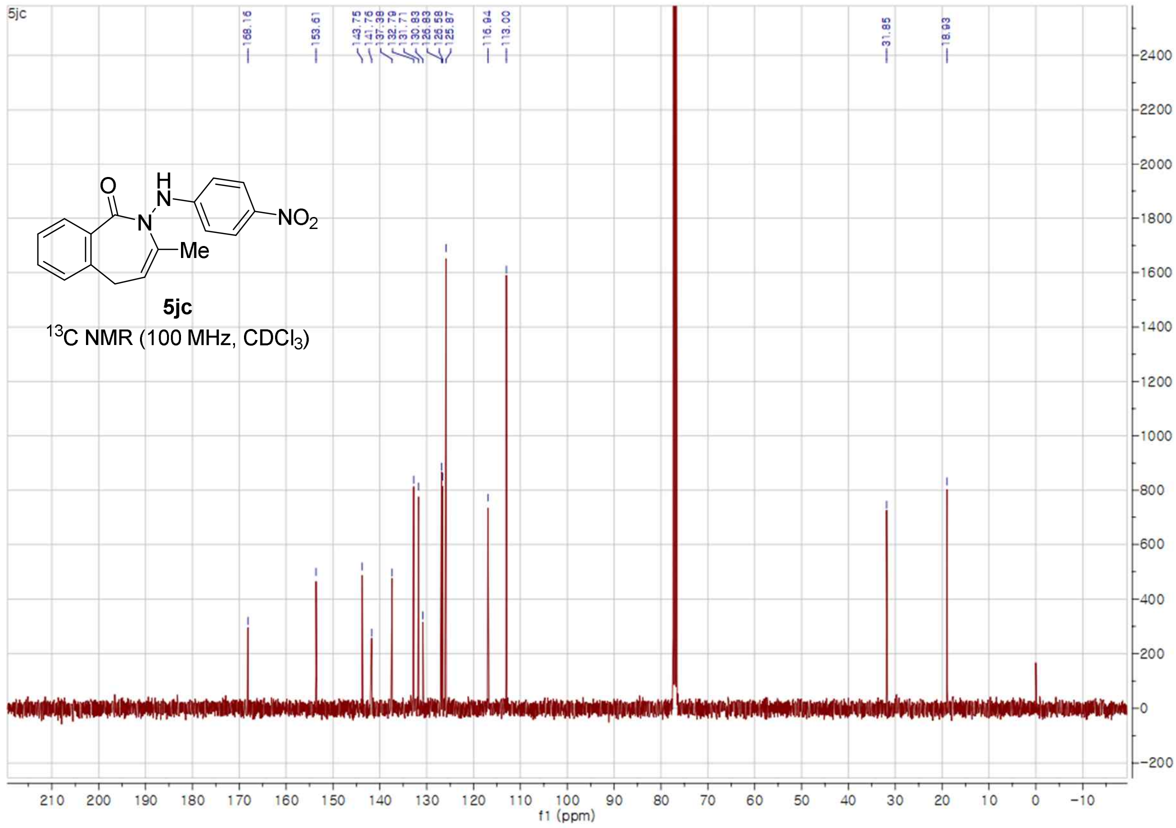
5jc
168.16
153.61
143.75
116.94
113.00
31.85
18.93
NO2
Me
O
N
H
5jc
13C NMR (100 MHz, CDCl3)
f1 (ppm)

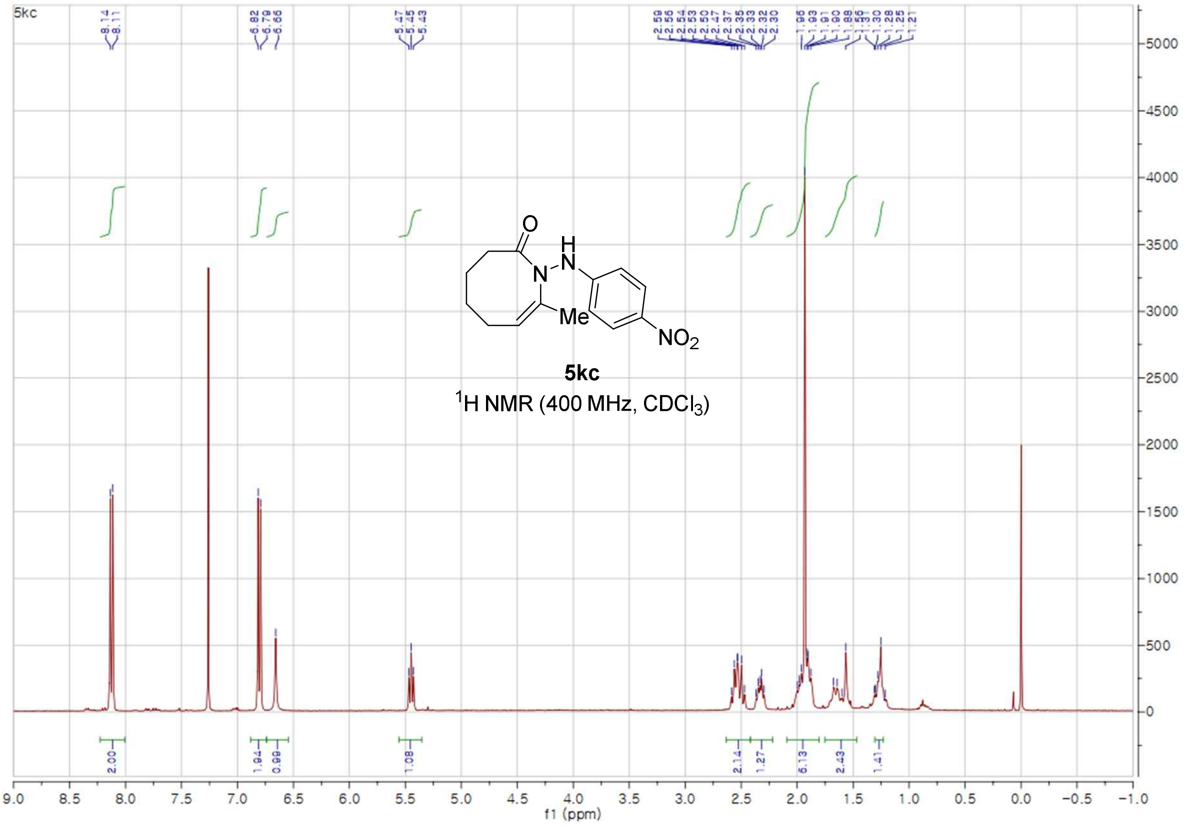
5kc
5kc
1H NMR (400 MHz, CDCl3)
f1 (ppm)

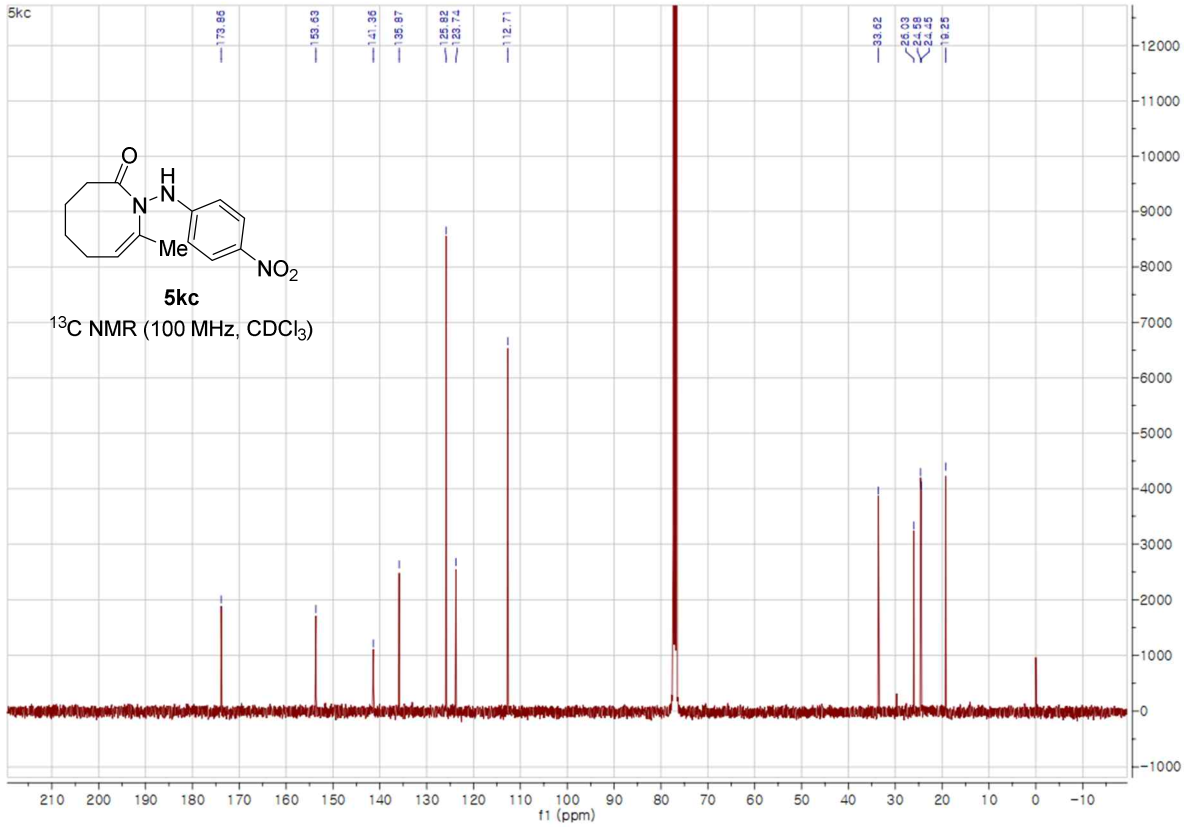
5kc
5kc
13C NMR (100 MHz, CDCl3)
173.86
153.63
141.36
135.87
125.82
123.74
112.71
33.62
26.03
24.58
24.45
19.25
f1 (ppm)

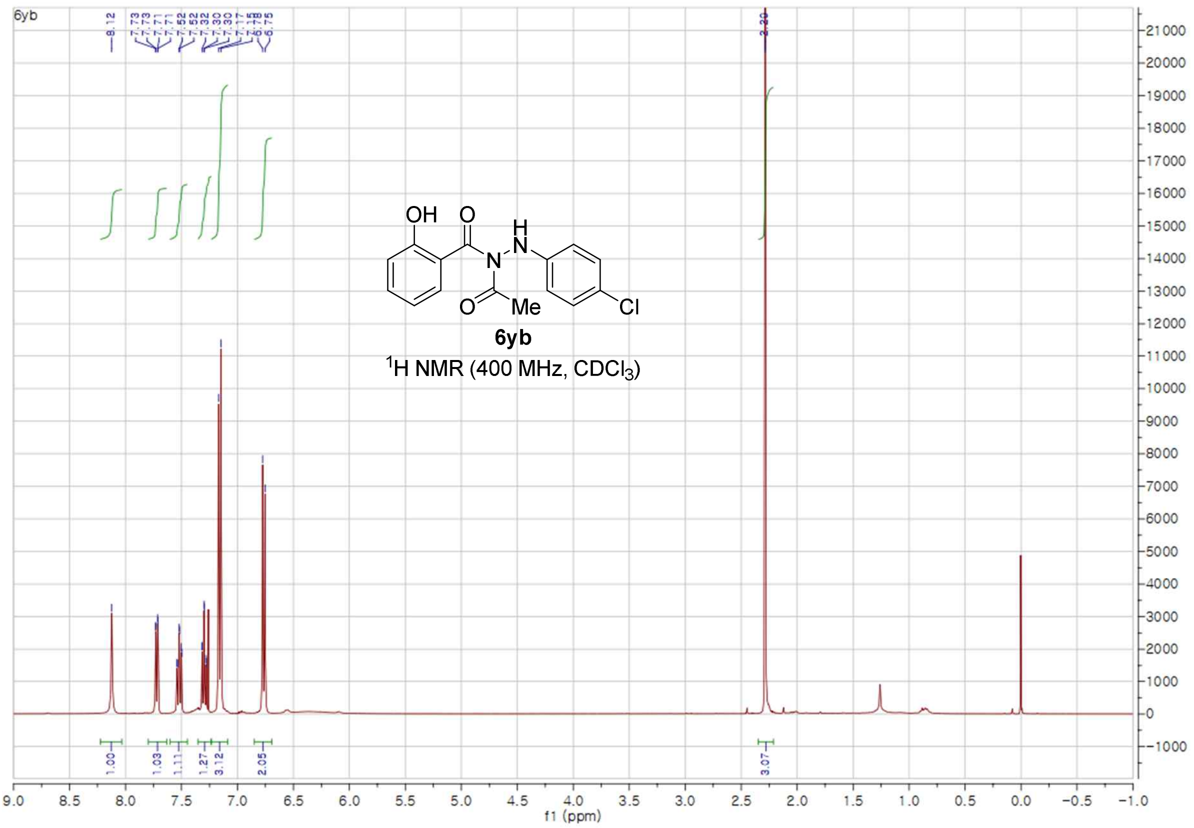
6yb
1H NMR (400 MHz, CDCl3)
f1 (ppm)

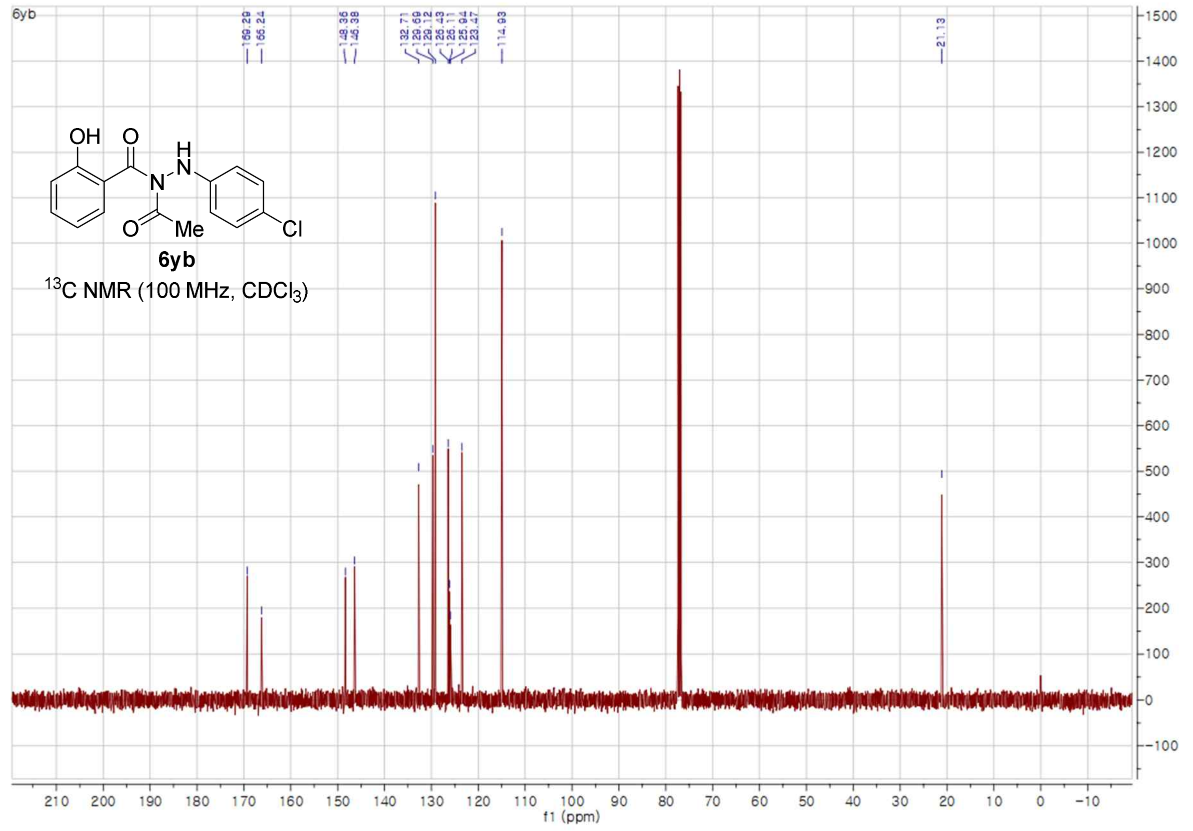
6yb
13C NMR (100 MHz, CDCl3)
f1 (ppm)

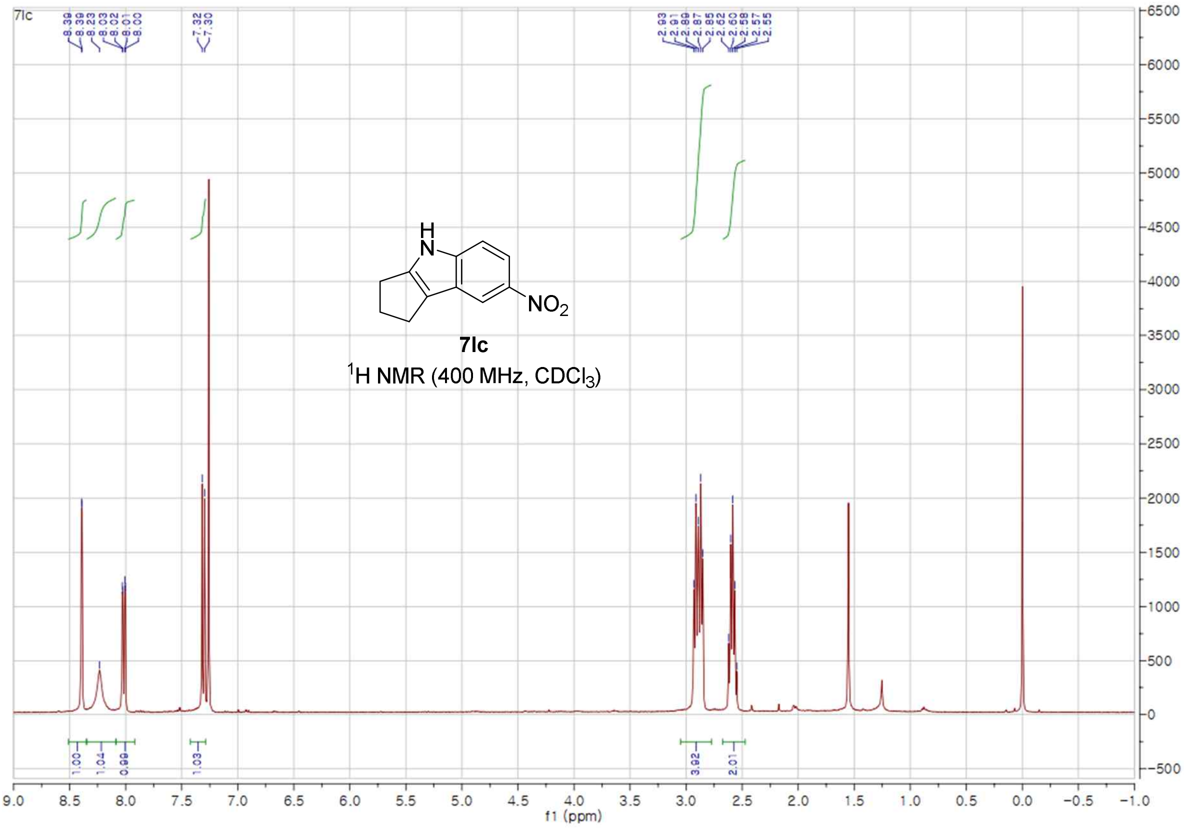
7lc
8.39
8.39
8.23
8.03
8.02
8.01
8.00
7.32
7.30
2.93
2.91
2.89
2.87
2.85
2.62
2.60
2.58
2.57
2.55
H
N
NO_2
7lc
^{1}H NMR (400 MHz, $CDCl_3$)
1.00
1.04
0.99
1.03
3.92
2.01
f1 (ppm)

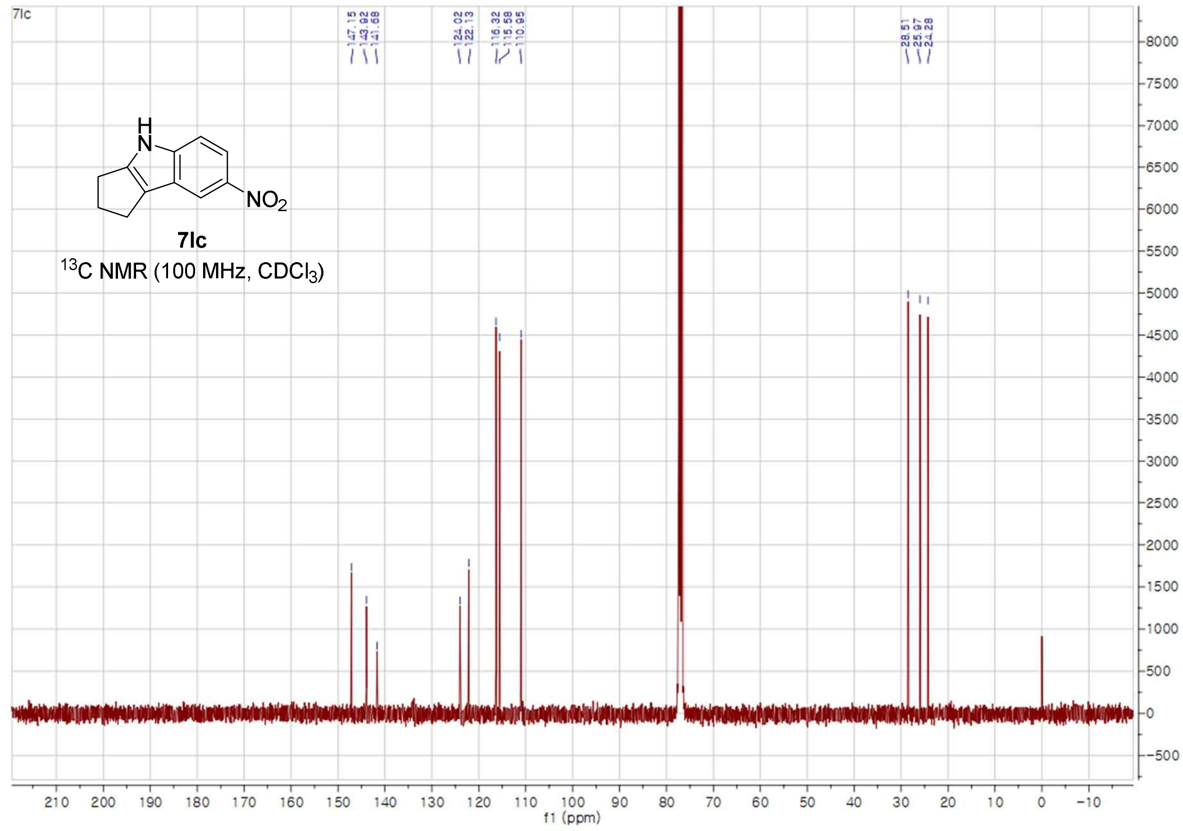
7lc
147.15
143.92
141.68
124.02
122.13
116.32
115.58
110.95
28.51
25.97
24.28
H
N
NO_2
7lc
^{13}C NMR (100 MHz, $CDCl_3$)
f1 (ppm)

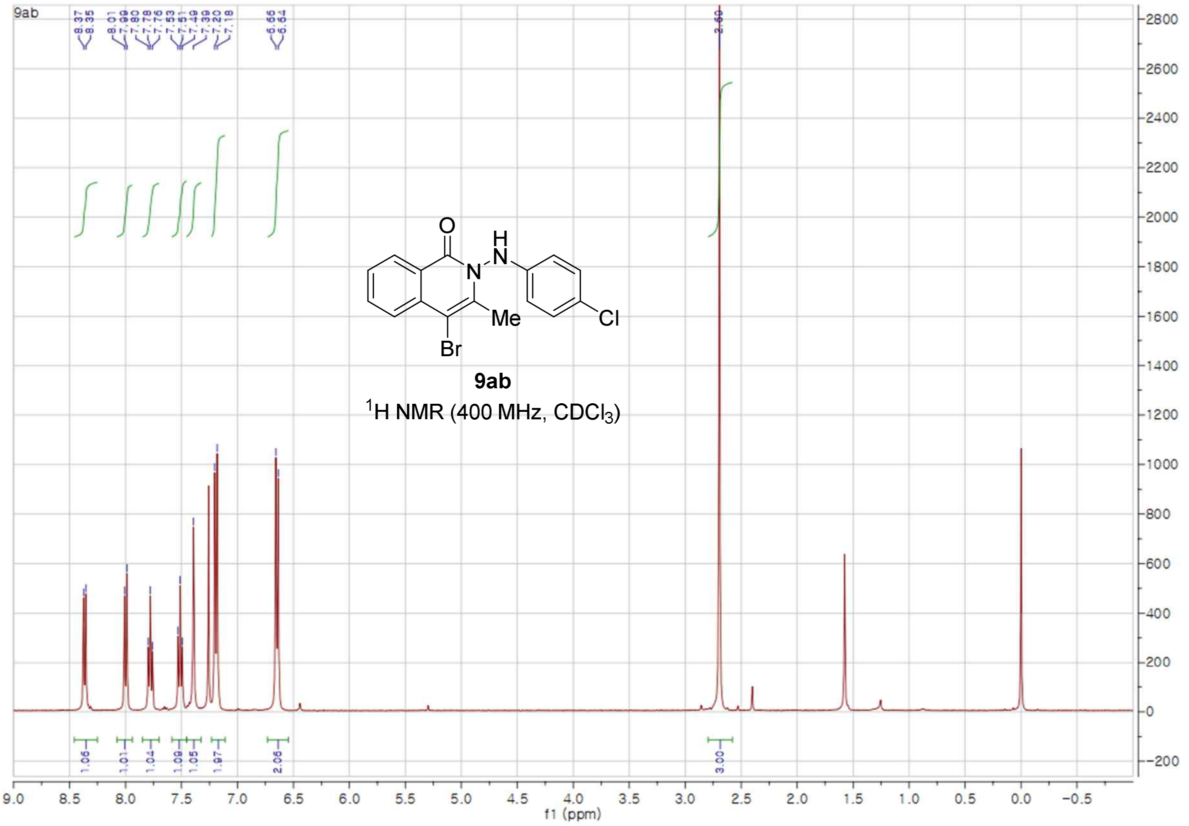
9ab
8.37
8.35
8.01
7.99
7.80
7.78
7.76
7.53
7.51
7.49
7.39
7.20
7.18
6.65
6.64
2.69
O
H
N
N
Me
Cl
Br
9ab
1H NMR (400 MHz, CDCl3)
1.06
1.01
1.04
1.09
1.05
1.97
2.06
3.00
f1 (ppm)

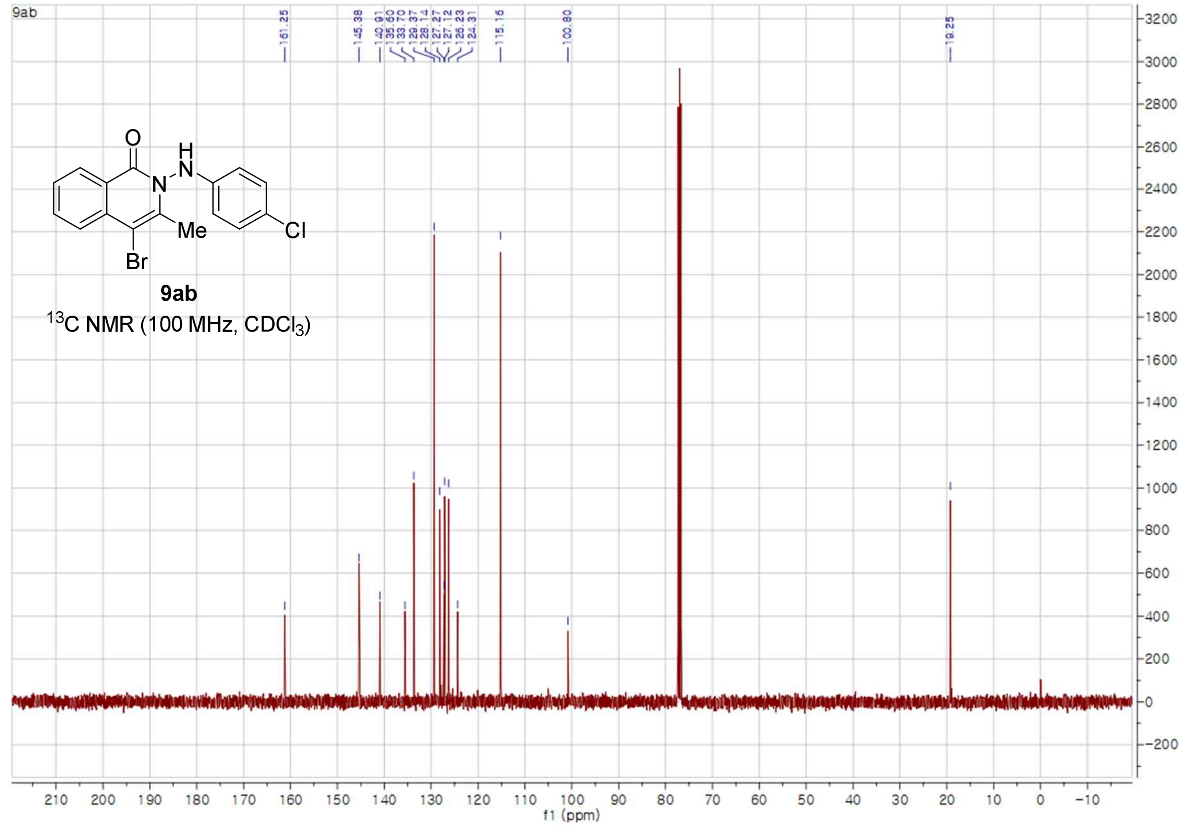
9ab
161.25
145.38
140.91
135.60
133.70
129.37
128.14
127.27
127.12
126.23
124.31
115.16
100.80
19.25
O
H
N
N
Me
Cl
Br
9ab
13C NMR (100 MHz, CDCl3)
f1 (ppm)

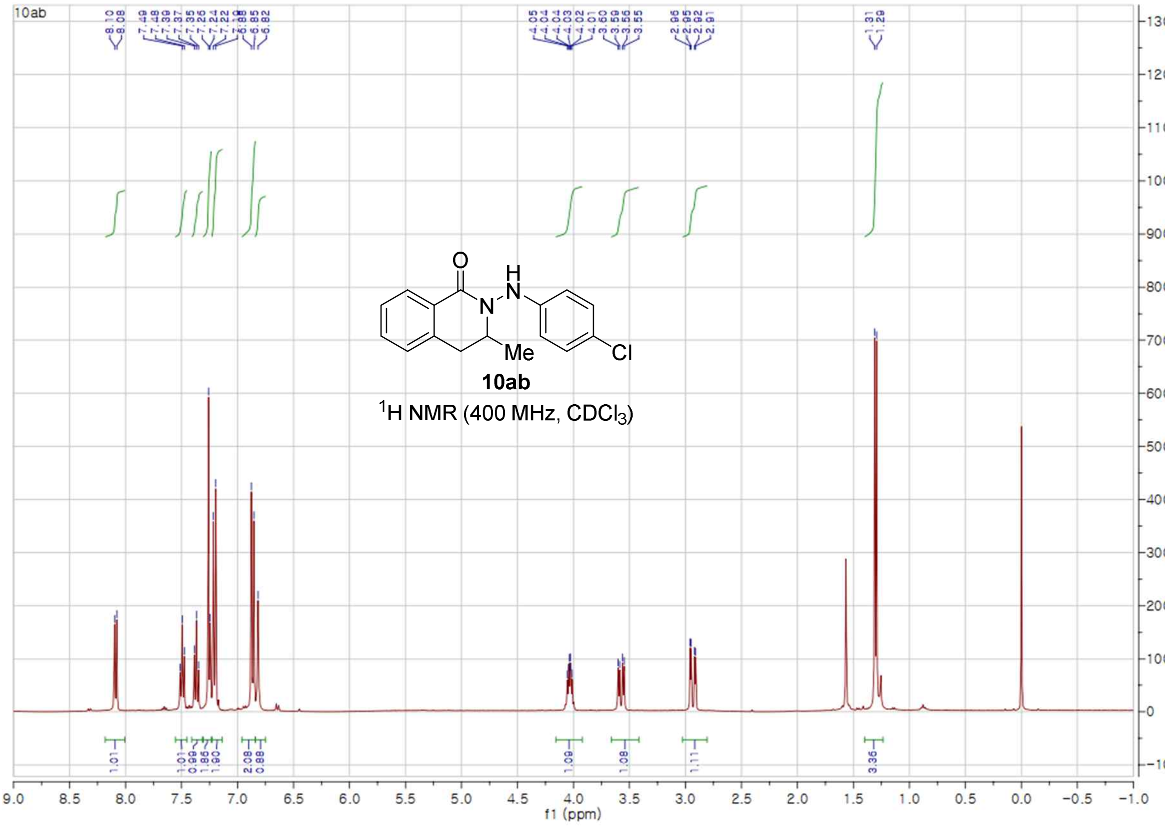
10ab
Me
Cl
10ab
1H NMR (400 MHz, CDCl3)
f1 (ppm)

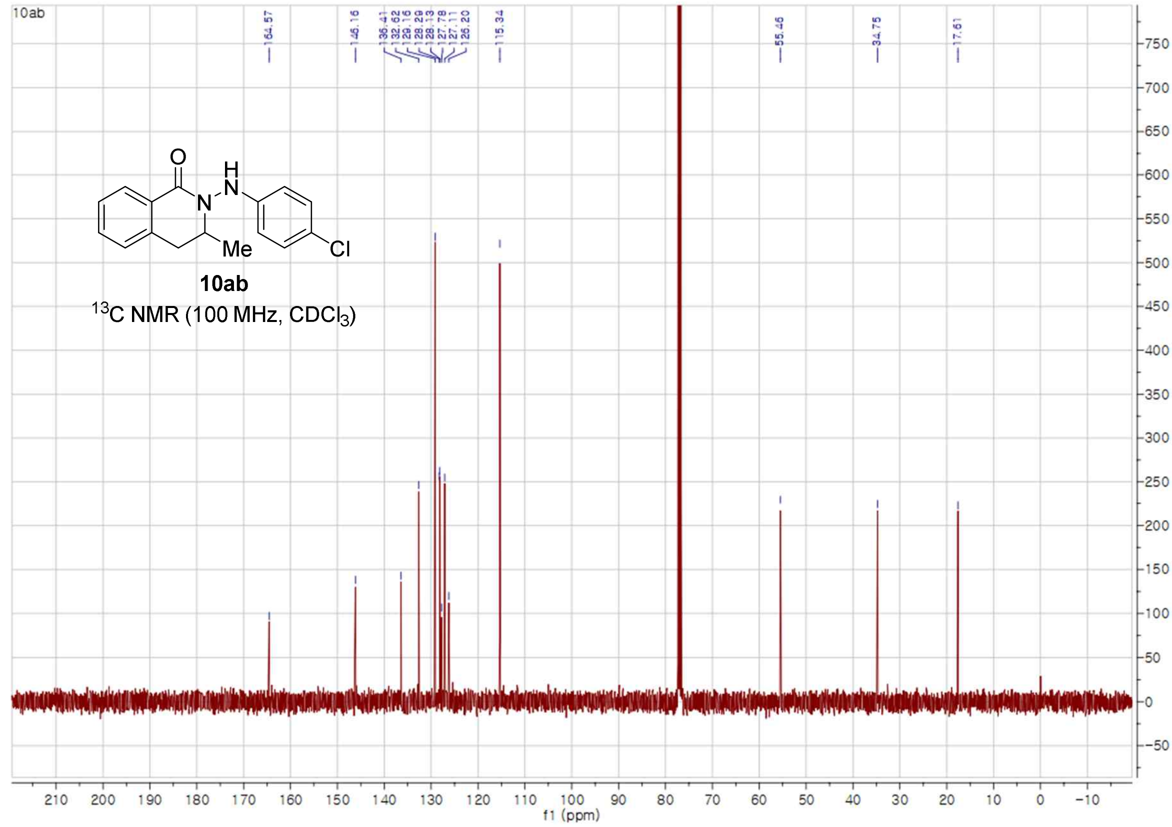
10ab
Me
Cl
10ab
13C NMR (100 MHz, CDCl3)
f1 (ppm)

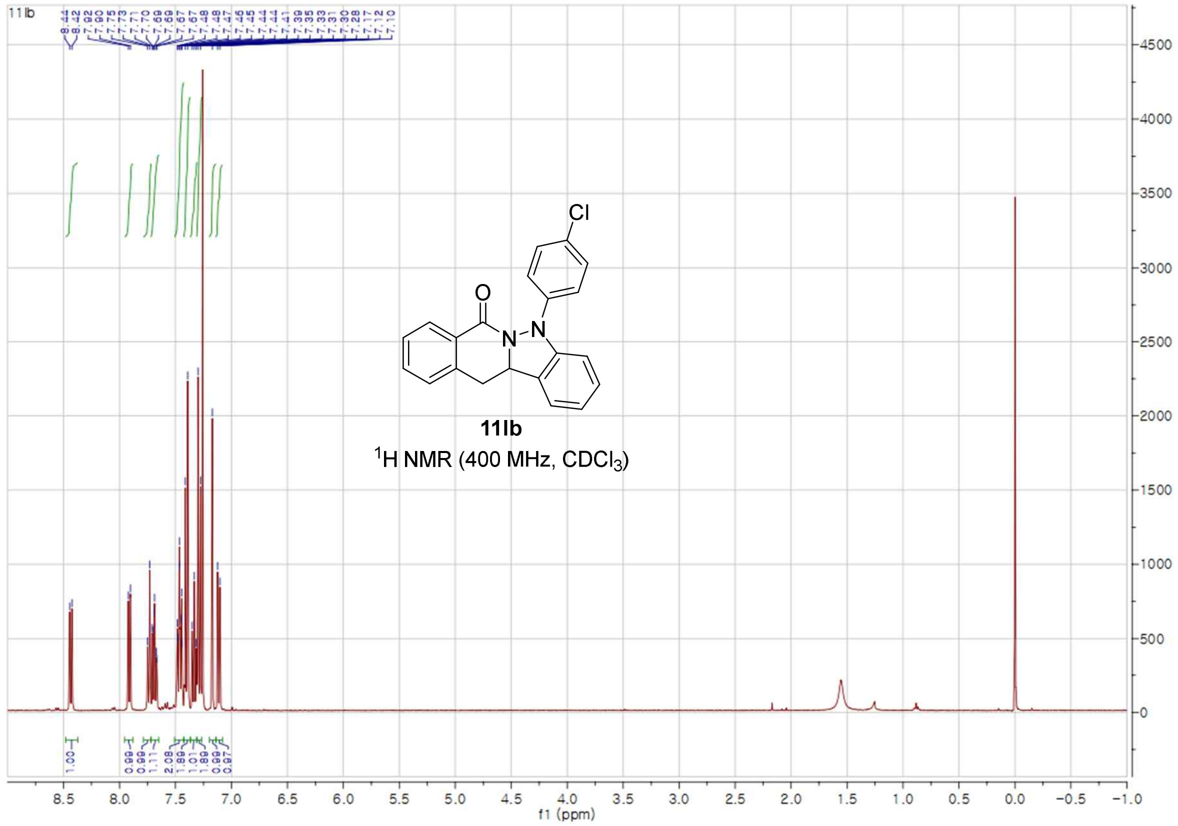

11lb
Cl
O
N
N
11lb
^{1}H NMR (400 MHz, CDCl$_3$)
f1 (ppm)

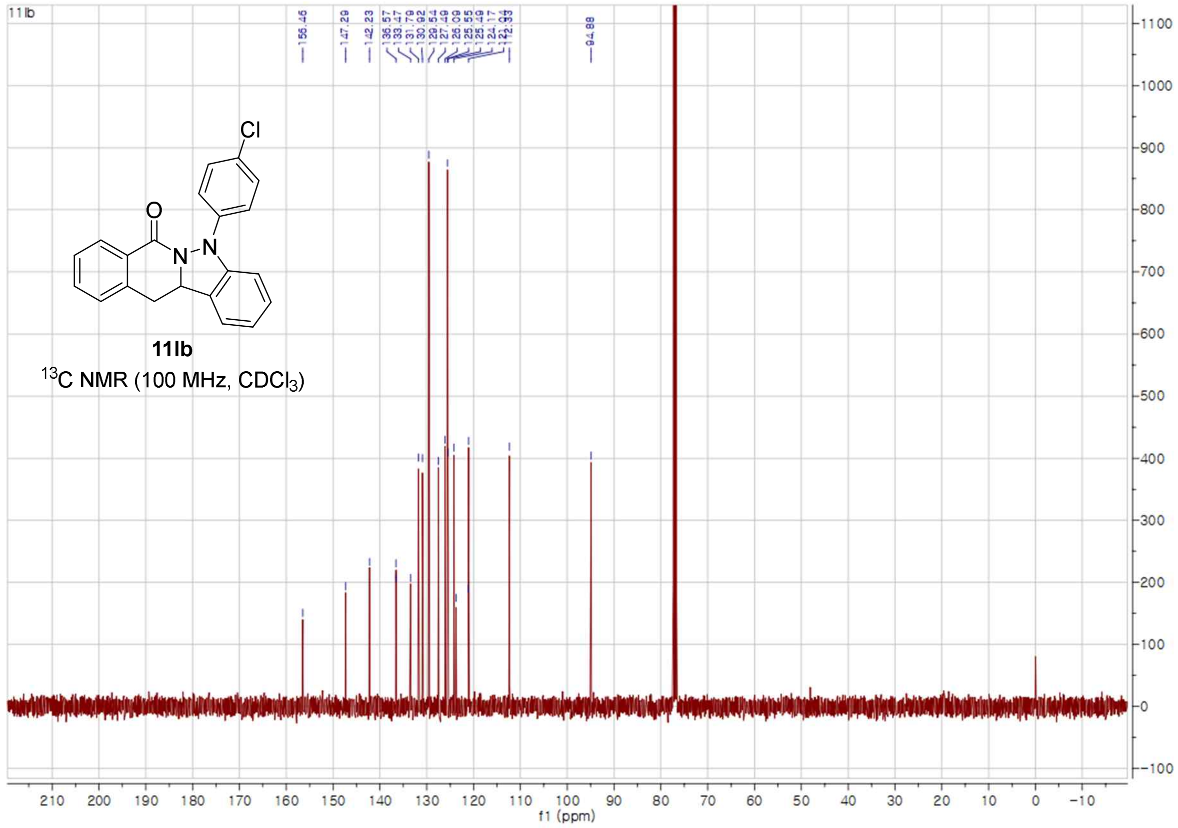

11lb
Cl
O
N
N
11lb
^{13}C NMR (100 MHz, CDCl$_3$)
f1 (ppm)

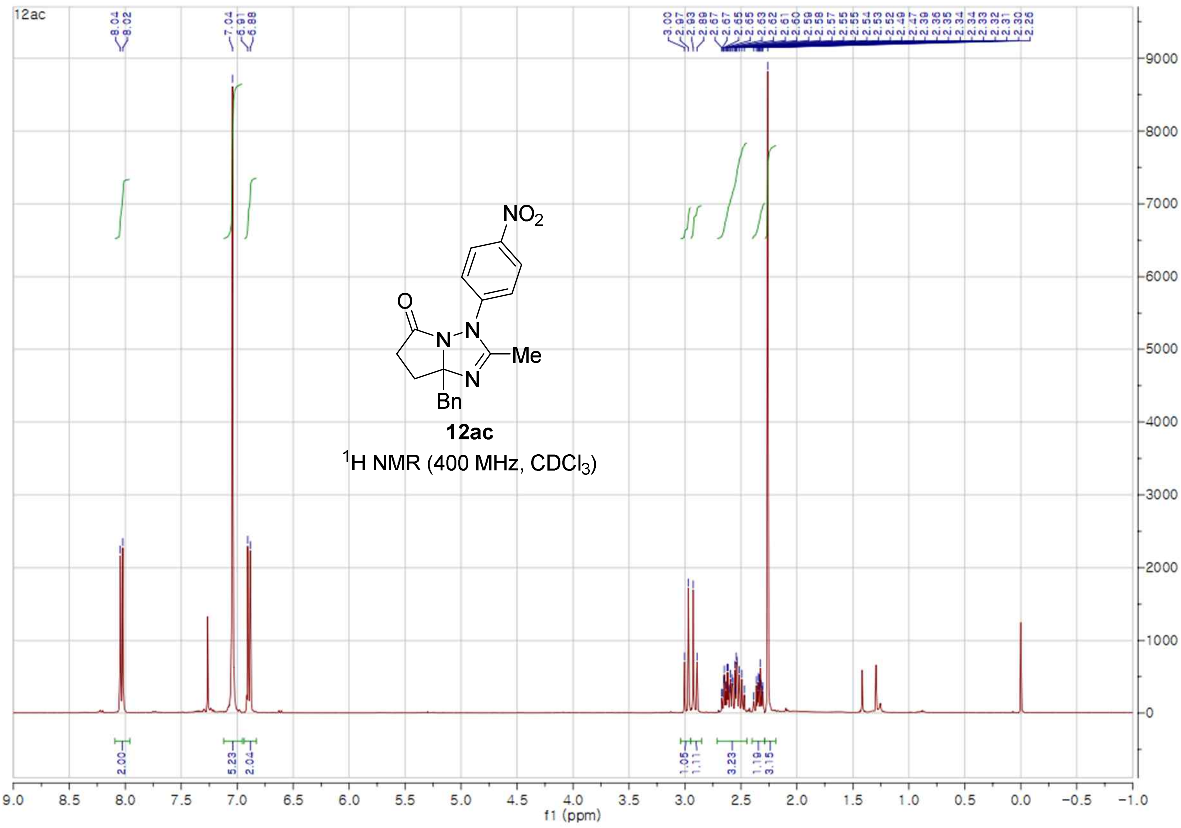
12ac
8.04
8.02
7.04
6.91
6.89
3.00
2.97
2.93
2.89
NO_2
O
N
N
Me
N
Bn
12ac
^1H NMR (400 MHz, $CDCl_3$)
2.00
5.23
2.04
1.05
1.11
3.23
1.19
3.15
f1 (ppm)

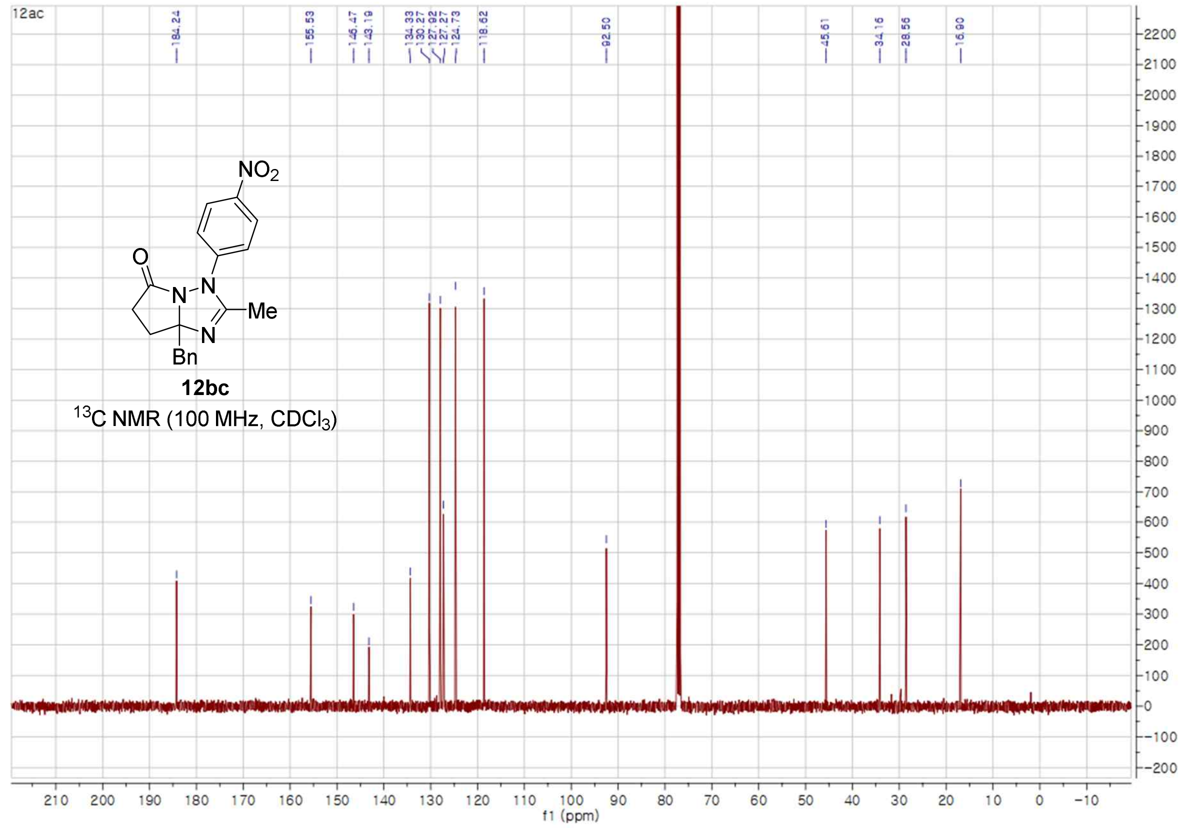
12ac
184.24
155.53
146.47
143.19
134.33
130.27
127.92
127.27
124.73
118.62
92.50
45.61
34.16
28.56
16.90
NO_2
O
N
N
Me
N
Bn
12bc
^{13}C NMR (100 MHz, $CDCl_3$)
f1 (ppm)

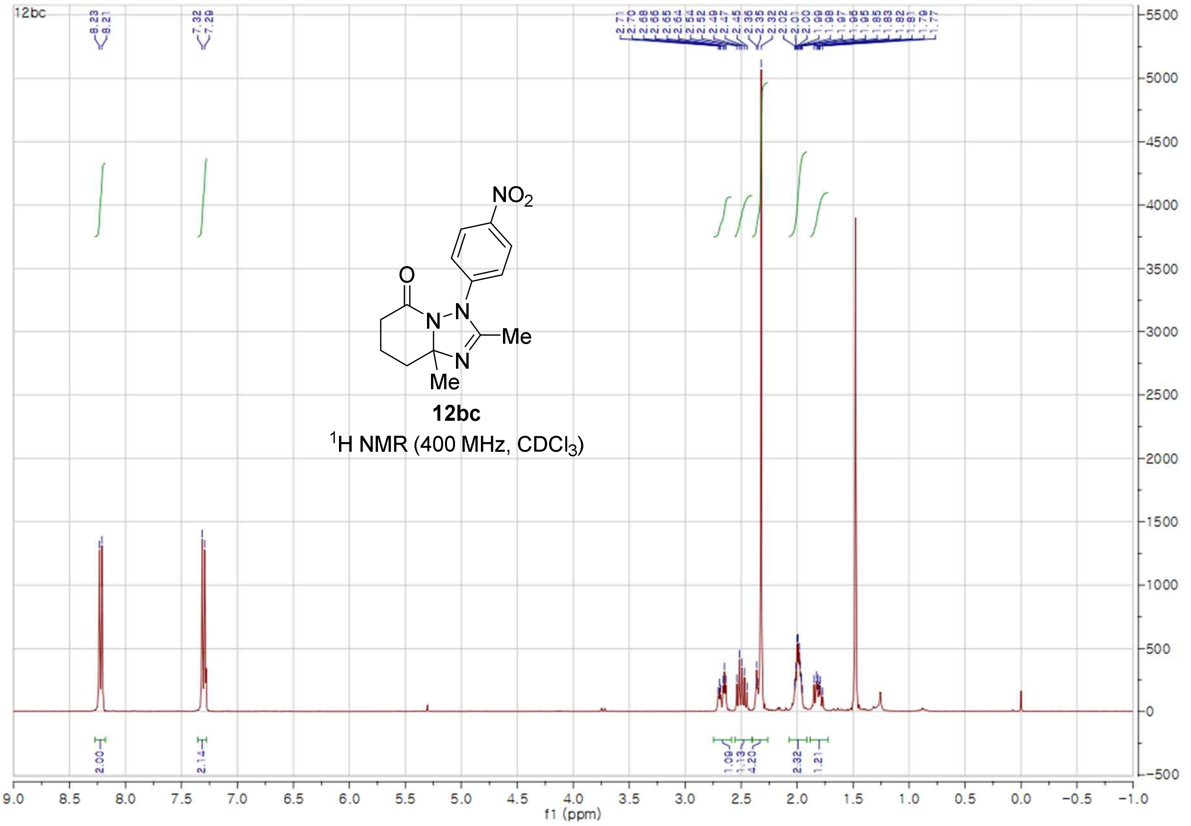

12bc
NO2
O
N
N
Me
N
Me
12bc
1H NMR (400 MHz, CDCl3)
8.23
8.21
7.32
7.29
2.00
2.14
1.09
1.18
4.20
2.32
1.21
f1 (ppm)

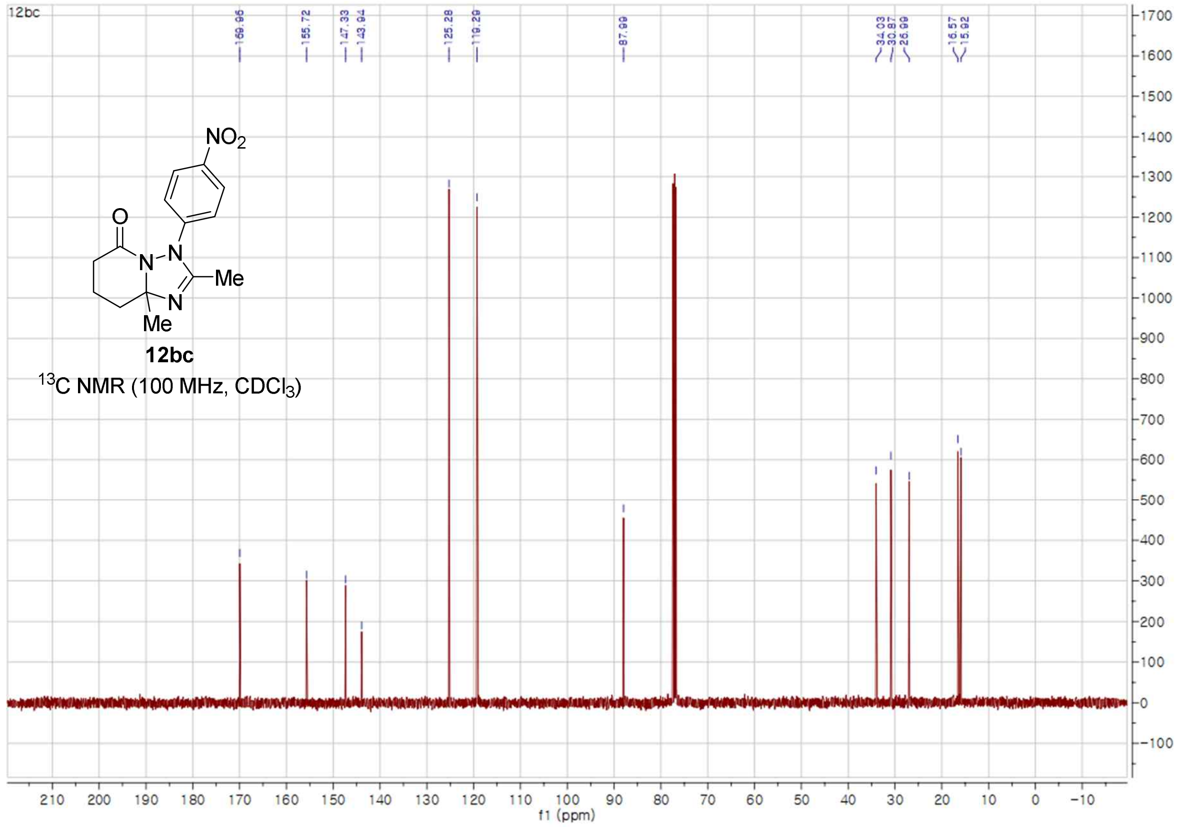

12bc
169.95
155.72
147.33
143.94
125.28
119.29
87.99
34.03
30.87
26.99
16.57
15.92
NO2
O
N
N
Me
N
Me
12bc
13C NMR (100 MHz, CDCl3)
f1 (ppm)

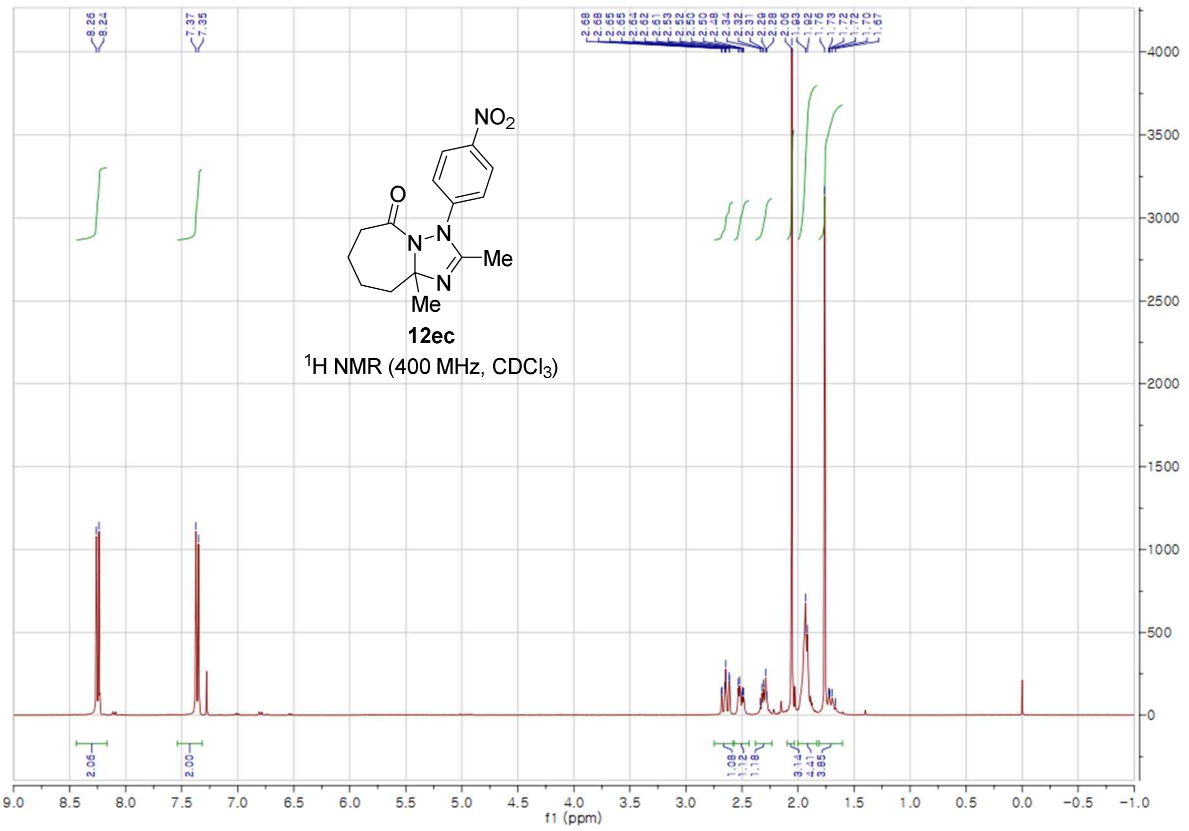

NO2
O
N
N
Me
N
Me
12ec
1H NMR (400 MHz, CDCl3)
8.26
8.24
7.37
7.35
2.06
2.00
1.08
1.12
1.18
3.14
4.41
3.85
f1 (ppm)

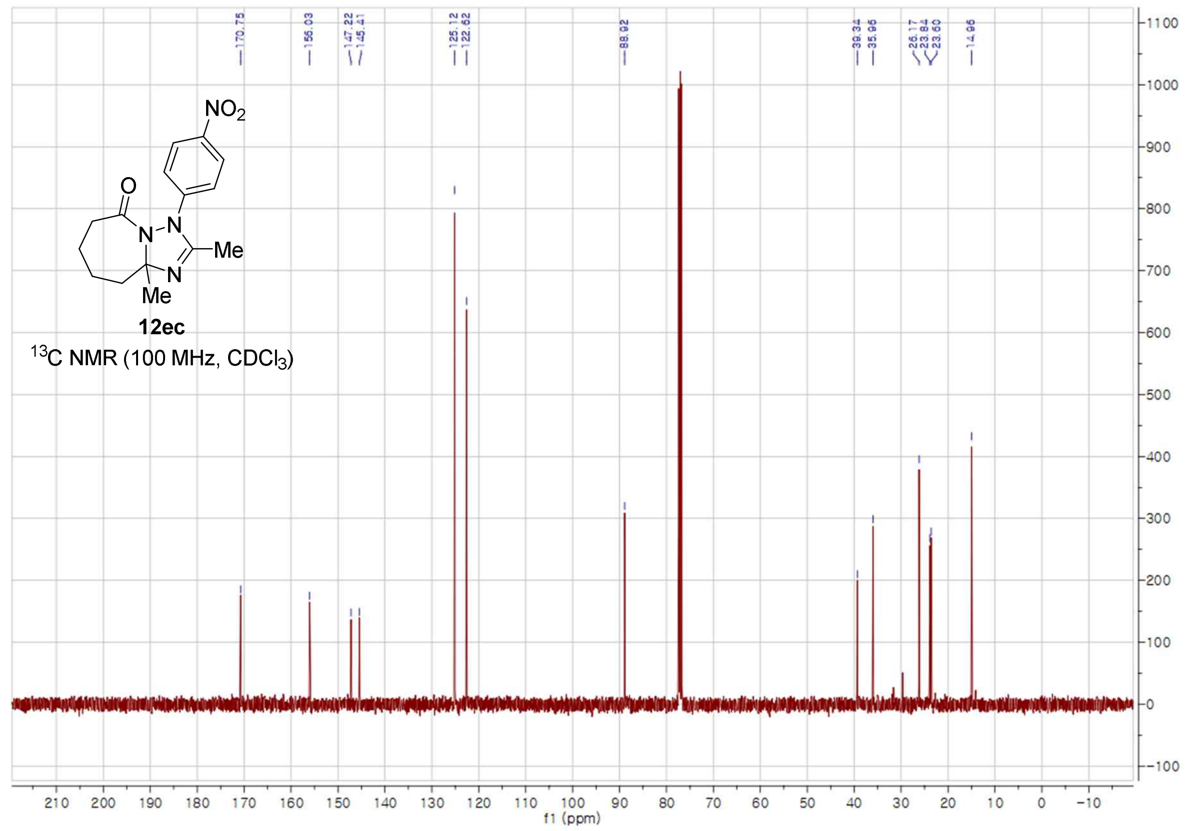

NO2
O
N
N
Me
N
Me
12ec
13C NMR (100 MHz, CDCl3)
170.75
156.03
147.22
145.41
125.12
122.62
88.92
39.34
35.96
26.17
23.84
23.60
14.96
f1 (ppm)

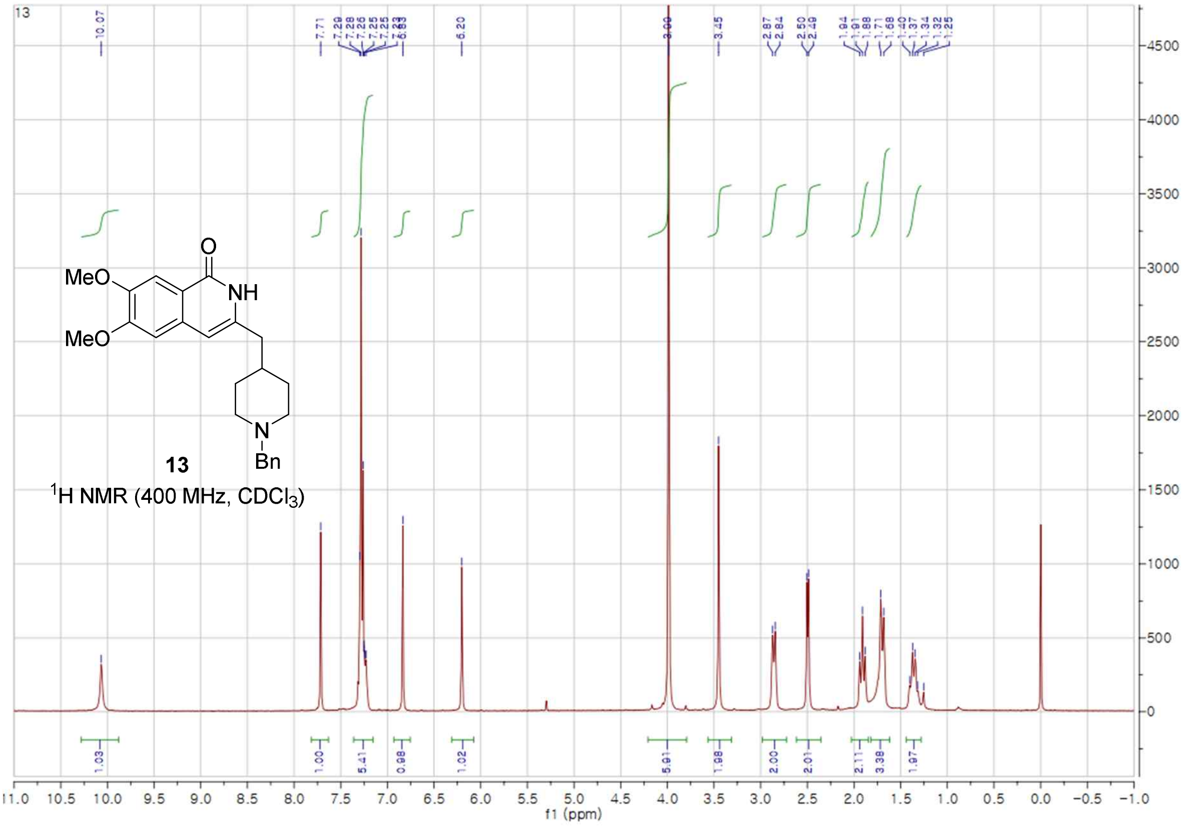

13

^{1}H NMR (400 MHz, $CDCl_3$)

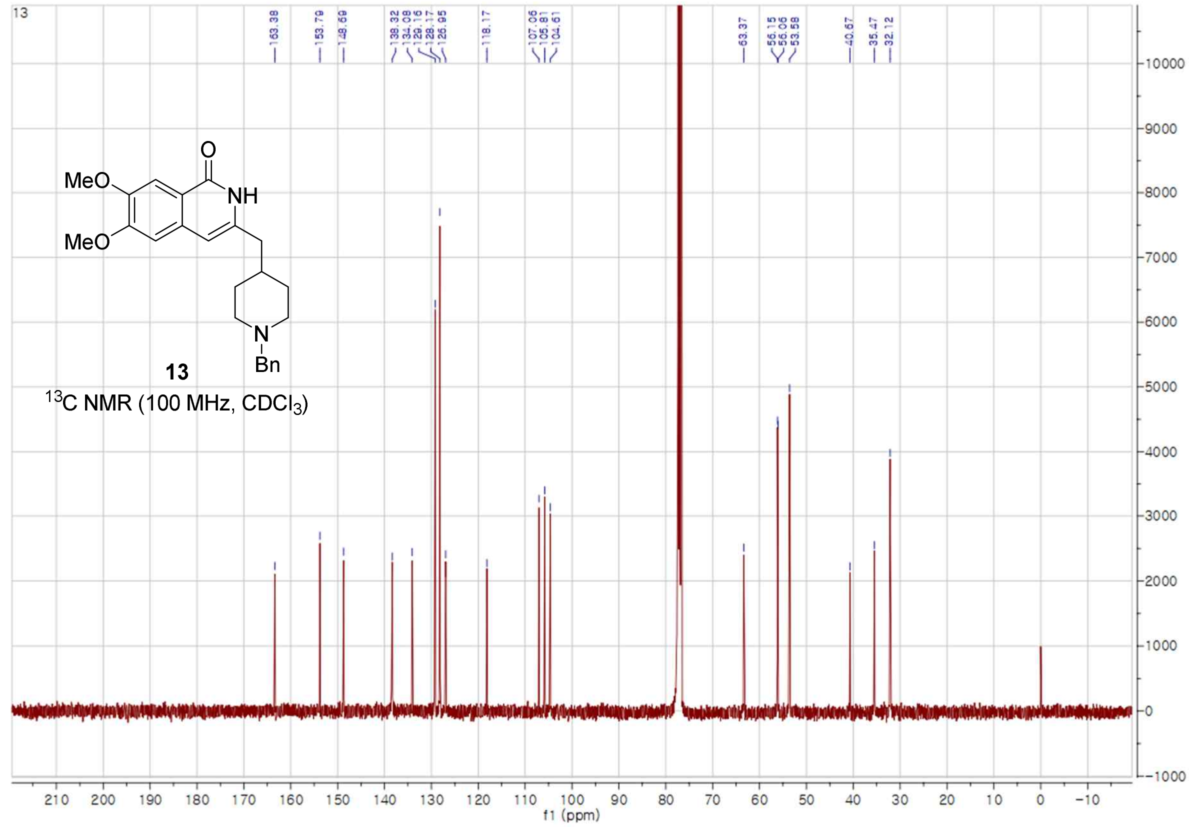

13

^{13}C NMR (100 MHz, $CDCl_3$)
